# Risk assessment models for venous thromboembolism in hospitalised adult patients: a systematic review

**DOI:** 10.1136/bmjopen-2020-045672

**Published:** 2021-07-29

**Authors:** Abdullah Pandor, Michael Tonkins, Steve Goodacre, Katie Sworn, Mark Clowes, Xavier L Griffin, Mark Holland, Beverley J Hunt, Kerstin de Wit, Daniel Horner

**Affiliations:** 1 ScHARR, The University of Sheffield, Sheffield, UK; 2 Barts and The London School of Medicine and Dentistry, Queen Mary University of London, London, UK; 3 Department of Clinical and Biomedical Sciences, University of Bolton, Bolton, UK; 4 Department of Haematology, Guy's and St Thomas' NHS Foundation Trust, London, UK; 5 Department of Medicine, McMaster University, Hamilton, Ontario, Canada; 6 Emergency Department, Salford Royal NHS Foundation Trust, Salford, UK

**Keywords:** Vascular Medicine, Haematology, Anticoagulation, Quality in health care

## Abstract

**Introduction:**

Hospital-acquired thrombosis accounts for a large proportion of all venous thromboembolism (VTE), with significant morbidity and mortality. This subset of VTE can be reduced through accurate risk assessment and tailored pharmacological thromboprophylaxis. This systematic review aimed to determine the comparative accuracy of risk assessment models (RAMs) for predicting VTE in patients admitted to hospital.

**Methods:**

A systematic search was performed across five electronic databases (including MEDLINE, EMBASE and the Cochrane Library) from inception to February 2021. All primary validation studies were eligible if they examined the accuracy of a multivariable RAM (or scoring system) for predicting the risk of developing VTE in hospitalised inpatients. Two or more reviewers independently undertook study selection, data extraction and risk of bias assessments using the PROBAST (Prediction model Risk Of Bias ASsessment Tool) tool. We used narrative synthesis to summarise the findings.

**Results:**

Among 6355 records, we included 51 studies, comprising 24 unique validated RAMs. The majority of studies included hospital inpatients who required medical care (21 studies), were undergoing surgery (15 studies) or receiving care for trauma (4 studies). The most widely evaluated RAMs were the Caprini RAM (22 studies), Padua prediction score (16 studies), IMPROVE models (8 studies), the Geneva risk score (4 studies) and the Kucher score (4 studies). C-statistics varied markedly between studies and between models, with no one RAM performing obviously better than other models. Across all models, C-statistics were often weak (<0.7), sometimes good (0.7–0.8) and a few were excellent (>0.8). Similarly, estimates for sensitivity and specificity were highly variable. Sensitivity estimates ranged from 12.0% to 100% and specificity estimates ranged from 7.2% to 100%.

**Conclusion:**

Available data suggest that RAMs have generally weak predictive accuracy for VTE. There is insufficient evidence and too much heterogeneity to recommend the use of any particular RAM.

**PROSPERO registration number:**

Steve Goodacre, Abdullah Pandor, Katie Sworn, Daniel Horner, Mark Clowes. A systematic review of venous thromboembolism RAMs for hospital inpatients. PROSPERO 2020 CRD42020165778. Available from https://www.crd.york.ac.uk/prospero/display_record.php?RecordID=165778https://www.crd.york.ac.uk/prospero/display_record.php?RecordID=165778

**Strengths and limitations of this study**

- This systematic review provides an up-to-date comprehensive review of risk assessment models for predicting venous thromboembolism in patients admitted to hospital.
- The newly developed PROBAST (Prediction model Risk Of Bias ASsessment Tool) tool was used to evaluate the risk of bias and applicability of the available evidence.
- Heterogeneity in the included studies (participants, inclusion criteria, clinical condition, outcome definition and measurement) and variable reporting of items precluded meta-analysis.
- Limitations of the existing evidence and areas of future research are highlighted.

## Introduction

Venous thromboembolism (VTE) is an important and life-threatening complication of hospitalisation and illness, and is associated with significant morbidity and mortality.^1 2^ Globally, an estimated 10 million VTE episodes are diagnosed each year; over half of these episodes are associated with hospital inpatients stays and result in significant loss of disability-adjusted life years.^3 4^ Consequently, there has been a substantial and sustained focus on VTE prevention over the last three decades, with good evidence indicating a reduction in morbidity with primary thromboprophylaxis in hospitalised patients.^5–8^ Despite this evidence, thromboprophylaxis remains either underused or inappropriately applied.^9^

Risk assessment models (RAMs) have been developed to help stratify the risk of VTE among hospitalised patients.^10^ These models use clinical information from the patient’s history and examination to identify those with an increased risk of developing VTE who are most likely to benefit from pharmacological prophylaxis. Inappropriate use of VTE prophylaxis may not reduce VTE rates and may cause unnecessary harm.^11^ While RAMs could improve the ratio of benefit to risk and benefit to cost, it is unclear which VTE RAM should be applied to guide decision-making for prophylaxis in clinical practice and thereby optimise patient care.

The current review extends and updates three broadly overlapping existing reviews.^10 12 13^ While these reviews identified the use of various (derived and validated) RAMs for VTE in hospitalised patients, they did not find any evidence to suggest which RAM was superior. The aim of this systematic review was to identify primary validation studies (as derivation studies may give an overoptimistic assessment of model performance measures) and determine the accuracy of individual RAMs for predicting the risk of developing VTE in hospital inpatients.

## Methods

A systematic review was undertaken in accordance with the general principles recommended in the Preferred Reporting Items for Systematic Reviews and Meta-Analyses (PRISMA) statement.^14^ This review was part of a larger project on VTE RAMs for hospital inpatients^15^ and was registered on the International Prospective Register of Systematic Reviews (PROSPERO) database (CRD42020165778).

### Eligibility criteria

We sought studies evaluating RAMs which could be applied to a general inpatient population (medical, surgical or trauma) rather than disease-specific models. All primary validation studies that evaluated the accuracy (eg, sensitivity, specificity, C-statistic) of a multivariable RAM (or scoring system) for predicting the risk of developing VTE were eligible for inclusion. We selected studies that included validation of the model in a group of patients that were not involved in model derivation. This involved either splitting the study cohort (internal) or using a new cohort (external). The study could have reported derivation of the model but we only used the validation data to estimate accuracy. The study population consisted of hospital inpatients including those who required medical care, undergoing any surgery (excluding day surgery) or received care following an injury. Studies that primarily focused on children (aged under 16 years), women admitted to hospital for pregnancy-related reasons and any patient admitted to a level 2 or above critical care environment (eg, patients requiring more detailed observation or intervention including support for a single failing organ system or postoperative care and those ‘stepping down’ from higher levels of care) were excluded. These patient groups have VTE risk profiles that differ markedly from the general inpatient population, making the use of a generic model inappropriate.

### Data sources and searches

Potentially relevant studies were identified through searches of five electronic databases including MEDLINE (with MEDLINE In-process and Epub Ahead of Print), EMBASE and the Cochrane Library. The search strategy used free text and thesaurus terms and combined synonyms relating to the condition (eg, VTE in medical inpatients) with risk prediction modelling terms. No language restrictions were used. However, as the current review updated three previous systematic reviews,^10 12 13^ searches were limited by date from 2017 (last search date from earlier reviews)^10^ to February 2021. Searches were supplemented by hand-searching the reference lists of all relevant studies (including existing systematic reviews); forward citation searching of included studies; contacting key experts in the field; and undertaking targeted searches of the World Wide Web using the Google search engine. Further details on the search strategy can be found in online supplemental appendix S1.

10.1136/bmjopen-2020-045672.supp1 Supplementary data

### Study selection

All titles were examined for inclusion by one reviewer (KS) and any citations that clearly did not meet the inclusion criteria (eg, non-human, unrelated to VTE inpatients) were excluded. All abstracts and full-text articles were then examined independently by two reviewers (KS and AP). Any disagreements in the selection process were resolved through discussion or if necessary, arbitration by a third reviewer (SG) and included by consensus.

### Data extraction and quality assessment

Data relating to study design, methodological quality and outcomes were extracted by one reviewer (KS) into a standardised data extraction form and independently checked for accuracy by a second (AP or MT). Any discrepancies were resolved through discussion to achieve agreement. Where differences were unresolved, a third reviewer’s opinion was sought (SG). Where multiple publications of the same study were identified, data were extracted and reported as a single study.

The methodological quality of each included study was assessed using PROBAST (Prediction model Risk Of Bias ASsessment Tool).^16 17^ This instrument evaluates four key domains: patient selection, predictors, outcome and analysis. Each domain is assessed in terms of risk of bias and the concern regarding applicability to the review (first three domains only). To guide the overall domain-level judgement about whether a study is at high, low or an unclear (in the event of insufficient data in the publication to answer the corresponding question) risk of bias, subdomains within each domain include a number of signalling questions to help judge with bias and applicability concerns. An overall risk of bias for each individual study was defined as low risk when all domains were judged as low; and high risk of bias when one or more domains were considered as high. Studies were assigned an unclear risk of bias if one or more domains were unclear and all other domains were low.

### Data synthesis and analysis

We were unable to perform meta-analysis due to significant levels of heterogeneity between studies (participants, inclusion criteria, clinical condition) and variable reporting of items. As a result, a prespecified narrative synthesis approach^18 19^ was undertaken, with data being summarised in tables with accompanying narrative summaries that included a description of the included variables, statistical methods and performance measures (eg, sensitivity, specificity and C-statistic (a value between 0.7 and 0.8 and >0.8 indicated good and excellent discrimination, respectively; and values <0.7 were considered weak^20^), where applicable. All analyses were conducted using Microsoft Excel V.2010 (Microsoft Corporation, Redmond, Washington, USA).

### Patient and public involvement

Patients and the public were not involved in the design or conduct of this systematic review.

## Results

### Study flow

**Figure 1:**
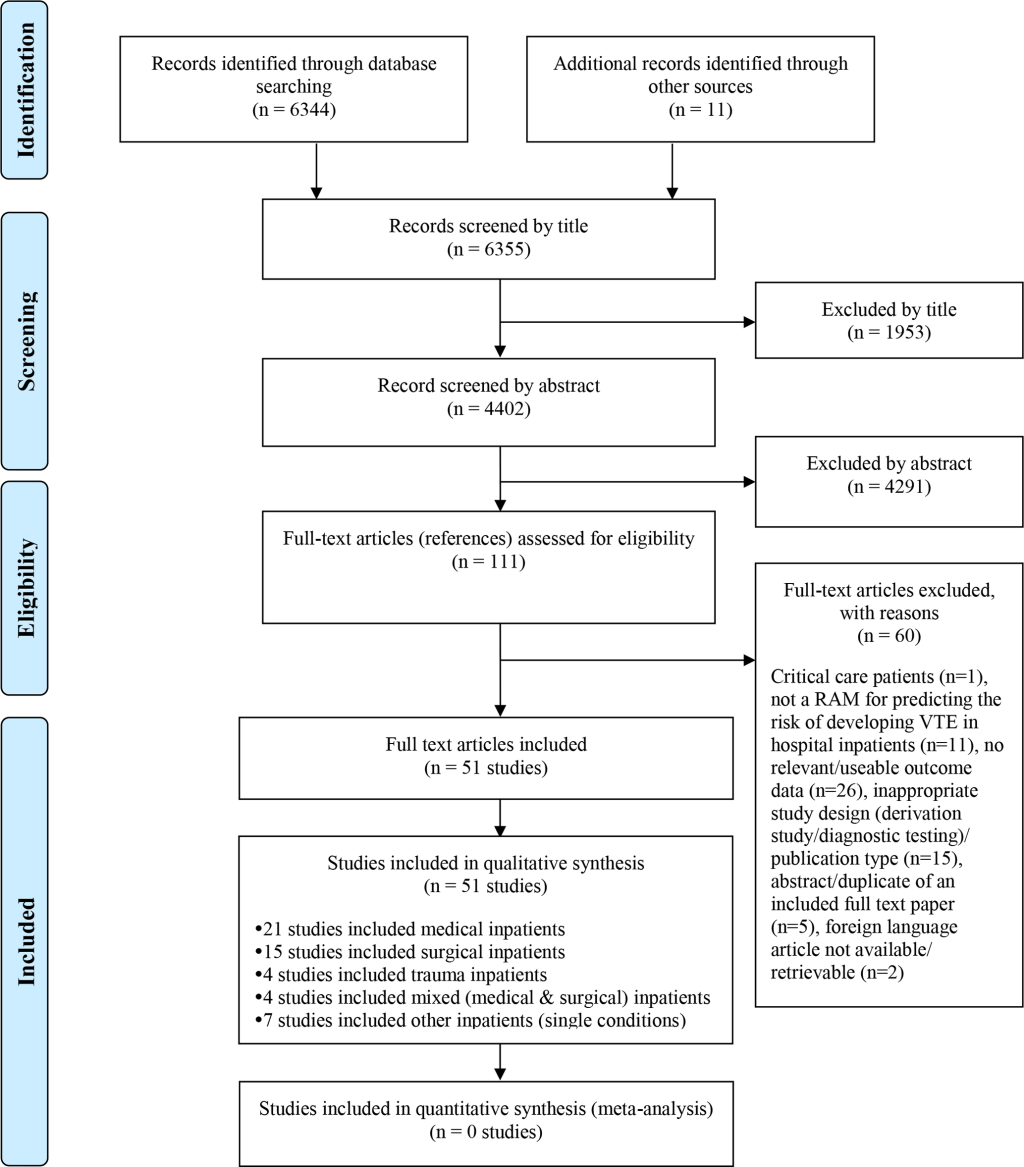
Study flowchart. RAM, risk assessment model; VTE, venous thromboembolism.

Figure 1 summarises the process of identifying and selecting relevant literature. Of the 6355 citations identified, 51 studies investigating 24 unique RAMs met the inclusion criteria. The majority of the articles were excluded primarily for not using a RAM for predicting the risk of developing VTE, having no useable or relevant outcome data or an inappropriate study design (eg, derivation study, reviews, commentaries or editorials). A full list of excluded studies with reasons for exclusion is provided in online supplemental appendix S2.

### Study and patient characteristics

The design and participant characteristics of the 51 included studies^21–71^ are summarised in table 1. All studies were published between 2003 and 2020 and were undertaken in North America (n=24),^23 25 33–40 43 47 48 52–59 65 68 69^ Asia (n=13),^29 30 42 44–46 60–63 67 70 71^ Europe (n=9),^22 24 26–28 31 49 51 66^ the Middle-East (n=2),^21 64^ South America (n=1),^32^ Australia (n=1)^41^ and one study was intercontinental.^50^ Sample sizes ranged from 70^40^ to 1 099 093^43^ patients in 37 observational cohort studies (11 prospective^21 22 24 28 29 32 44 51 52 56 64^ (5 of which were multicentre) and 26 retrospective^23 25–27 33 34 36–41 43 46 49 50 53–55 58 59 62 63 65 68 69^ (16 of which were multicentre) in design). Sample sizes in 14 case–control studies^30 31 35 42 45 47 48 57 60 61 66 67 70 71^ (4 of which were multicentre) ranged from 148^61^ to 19 217^57^ patients.

**Table 1:** Study and population characteristics

Author, year	Country	Design	Single/ Multicentre	Sample size	Population	Mean age (years)	Male	VTE prophylaxis	RAMs	Target condition (risk period)	Incidence	Validation methodology
Autar, 2003^22^	UK	P, CS	Single	148	Hospitalised patients from orthopaedic, medical and surgical specialties	NR	NR	50%	Novel (Autar, 2003)	DVT, not defined (90 days)	18.9%	External
Rogers *et al*, 2007^56^	USA	P, CS	Multi	91 308	Hospitalised surgical patients (undergoing vascular and general surgery)	NR	NR	NR	Novel (Rogers *et al*, 2007)	VTE (30 days)	0.6%	Internal: split (half)
Abdel-Razeq *et al*, 2010^21^	Jordan	P, CS	Single	606	Hospitalised (>24 hours) cancer patients aged ≥18 years	51	51%	55%	Caprini (modified)	VTE, symptomatic (60 days)	3.5%	External
Bahl *et al*, 2010^23^	USA	R, CS	Multi	8216	Hospitalised surgical patients (undergoing general, vascular and urologic surgery)	NR	NR	NR	Caprini	VTE (30 days)	1.4%	External
Barbar *et al*, 2010^24^	Italy	P, CS	Single	1180	Hospitalised medical patients	NR	47%	16%	Padua	VTE, symptomatic (90 days)	3.1%	External
Rothberg *et al*, 2011^58^	USA	R, CS	Multi	48 540	Hospitalised (≥3 days) medical patients aged ≥18 years	NR	NR	30%	Novel (Rothberg *et al,* 2011)	VTE, hospital associated (NR)	0.5%	Internal: split (20%)
Woller *et al*, 2011^69^	USA	R, CS	Multi	46 856	Hospitalised medical patients aged ≥18 years	61	46%	NR	Intermountain Kucher	VTE, defined by ICD-9 codes (90 days)	4.5%	Internal: split (25%)
Pannucci *et al*, 2012^53^	USA and Canada	R, CS	Multi	5761	Hospitalised (>2 days) patients with a burn injury aged ≥18 years	46	69%	NR	Novel (Panunucci *et al,* 2012)	VTE, not defined (NR)	1.0%	Internal: split (25%)
Rogers *et al*, 2012^55^	USA	R, CS	Multi	234 032	Hospitalised trauma patients	NR	NR	NR	TESS	VTE (NR)	NR	Internal: split
Bilimoria *et al*, 2013^25^	USA	R, CS	Multi	88 053	Hospitalised surgical patients (undergoing colorectal surgery)	NR	NR	NR	ACS NSQIP—Colon specific ACS NSQIP—Universal	DVT, not defined (30 days)	2.3%	External: split (by year)
Hegsted *et al*, 2013^39^	USA	R, CS	Single	2281	Hospitalised (≥2 days) trauma patients aged ≥13 years	45	70%	NR	RAP	DVT, not defined or PE (NR)	DVT: 10.5% PE: 1.5%	External
Vardi *et al*, 2013^64^	Israel	P, CS	Single	1080	Hospitalised (≥2 days) sepsis patients aged >18 years	75	52%	18%	Padua	VTE, hospital associated (NR)	1.3%	External
Ho *et al*, 2014^41^	Australia	R, CS	Single	357	Hospitalised major trauma patients	NR	75%	NR	TESS	VTE, symptomatic (NR)	20.7%	External
Liu *et al*, 2014^44^	China	P, CS	Single	287	Hospitalised acute stroke patients aged >18 years	NR	63%	22%	Post-stroke DVT prediction system	DVT (14±3 days)	10.5%	Internal: split (33%)
Mahan *et al*, 2014^47^	USA	CC	Multi	417	Hospitalised (≥3 days) medical patients aged ≥18 years	NR	49%	NR	IMPROVE (7-factor)	VTE, hospital associated (92 days)	NA	External
Nendaz *et al*, 2014^51^	Switzerland	P, CS	Multi	1478	Hospitalised (>24 hours) medical patients aged ≥18 years	65	53%	57%	Geneva Padua	VTE, symptomatic including PE or DVT (90 days)	2.0%	External
Pannucci *et al*, 2014^52^	USA	P, CS	Multi	3576	Hospitalised surgical patients aged ≥18 years	NR	NR	66%	Novel (Panunucci *et al,* 2014)	VTE (90 days)	1.4%	Internal: split (35%)
Rosenberg *et al*, 2014^57^	USA	CC	Multi	19 217	Hospitalised (≥3 days) medical patients aged ≥18 years	NR	47%	43%	IMPROVE (7-factor)	VTE, defined by ICD-9 codes (90 days)	NA	External
Zhou *et al,* 2014^71^	China	CC	Single	998	Hospitalised (≥2 days) medical patients aged >18 years	NR	58%	15%	Caprini Padua	VTE, defined by ICD-10 codes (NR)	NA	External
Hewes *et al*, 2015^40^	USA	R, CS	Single	70	Hospitalised cancer patients (undergoing oesophagectomy)	NR	83%	96%	Caprini (modified)	VTE (60 days)	14.3%	External
de Bastos *et al*, 2016^32^	Brazil	P, CS	Single	11 091	Hospitalised medical patients aged >18 years	50	61%	0%	Caprini	VTE, symptomatic (NR)	0.3%	External
Grant *et al*, 2016^36^	USA	R, CS	Multi	63 548	Hospitalised (≥2 days) medical patients aged ≥18 years	66	45%	61%	Caprini	VTE, hospital associated (90 days)	1.1%	External
Greene *et al*, 2016^37^	USA	R, CS	Multi	63 548	Acutely ill, hospitalised (≥2 days) medical patients aged ≥18 years	66	45%	61%	IMPROVE (4-factor) Intermountain Kucher Padua	VTE, hospital associated (90 days)	1.1%	External
Hachey *et al*, 2016^38^	USA	R, CS	Single	232	Hospitalised surgical patients (undergoing segmenectomy, lobectomy or pneumonectomy for lung cancer)	NR	43%	92%	Caprini	VTE (60 days)	5.2%	External
Lui *et al*, 2016^45^	China	CC	Single	640	Hospitalised (>2 days) medical patients aged ≥18 years	NR	52%	NR	Caprini Padua	VTE (NR)	N/A	External
Lobastov *et al*, 2016^46^	Russia	R, CS*	Multi	140	Hospitalised high-risk emergency surgery patients (undergoing general and neurosurgery)	69	49%	100%	Caprini	DVT or PE, new (NR)	27.9%	External
Shaikh *et al*, 2016^59^	USA	R, CS	Multi	1598	Hospitalised surgical patients (undergoing plastic surgery)	50	19%	34%	Caprini	VTE, not defined (30 days)	1.5%	External
Elias *et al*, 2017^34^	USA	R, CS	Single	30 726	Hospitalised (>2 days) medical and surgical patients	NR	44%	21%	Padua (automated)	VTE, defined by ICD-9 codes (NR)	0.8%	External
Frankel *et al*, 2017 (abstract)^35^	USA	CC	NR	149	Hospitalised surgical patients aged ≥18 years (undergoing robotic-assisted laparoscopic prostatectomy)	NR	NR	NR	Caprini	VTE, not defined (90 days)	NA	External
Krasnow *et al*, 2017 (abstract)^43^	USA	R, CS	Multi	1 099 093	Hospitalised surgical patients (major urological cancer surgery)	NR	NR	NR	Caprini	VTE, symptomatic (90 days)	1.2%	External
Patell *et al*, 2017^54^	USA	R, CS	Single	2780	Hospitalised (>24 hours) cancer patients aged >18 years	62 (median)	56%	65%	Khorana	VTE, defined by ICD-9 codes (NR)	3.8%	External
Winoker *et al*, 2017^68^	USA	R, CS	Multi	300	Hospitalised surgical patients (undergoing urological surgery using robot-assisted partial nephrectomy)	61 (median)	62%	NR	ACS NSQIP—Universal	VTE, not defined (NR)	0.3%	External
Blondon *et al*, 2018^28^	Switzerland	P, CS	Multi	1478	Hospitalised (>24 hour) medical patients aged ≥18 years	65	53%	59%	IMPROVE (7-factor) Geneva † Padua †	VTE, symptomatic including PE or DVT (90 days)	2.0%	External
Chen *et al*, 2018^30^	China	CC	Single	390	Hospitalised (>2 days) patients aged ≥18 years with and without DVT	NR	51%	41%	Caprini Padua	DVT (NR)	NA	External
Dornbus *et al*, 2018 (abstract)^33^	USA	R, CS	NR	2830	Hospitalised surgical patients (undergoing neurosurgery)	NR	NR	NR	Caprini	VTE, not defined (NR)	NR	External
Vaziri *et al*, 2018^65^	USA	R, CS	Single	1006	Hospitalised surgical patients (undergoing neurosurgery)	NR	46%	NR	ACS NSQIP- Universal	VTE, not defined (NR)	1.3%	External
Vincentelli *et al*, 2018^66^	Italy	CC	Multi	1215	Acutely ill, hospitalised medical patients aged >18 years	NR	44%	NR	Chopard Kucher Padua	VTE (NR)	NA	External
Zhou *et al*, 2018^70^	China	CC	Single	1804	Hospitalised (≥2 days) medical patients aged >18 years	NR	59%	5%	Caprini Padua	VTE, defined by ICD-10 codes (NR)	NA	External
Blondon *et al*, 2019a^26^	Italy	R, CS*	Single	1180	Hospitalised medical patients	72	47%	20%	Geneva (simplified)	VTE, symptomatic (90 days)	3.1%	External
Blondon *et al*, 2019b (abstract)^27^	Switzerland	R, CS *	Multi	991	Hospitalised elderly medical patients	75	55%	NR	Geneva (simplified) IMPROVE (NR) Padua	VTE, symptomatic (NR)	15.0%	External
Cobben *et al*, 2019^31^	Netherlands	CC	Multi	556	Hospitalised (>24 hours) medical patients	NR	52%	NR	Caprini Geneva IMPROVE (4-factor) IMPROVE (7-factor) Intermountain Kucher Lecumberri NAVAL NICE Guideline Padua PRETEMED guideline Zakai *et al* (model 2)	VTE (NR)	NA	External
Tachino *et al*, 2019^62^	Japan	R, CS	Multi	859	Hospitalised (>24 hours) trauma patients aged ≥18 years	NR	64%	NR	RAP Quick RAP	VTE (NR)	3.0%	External (RAP)/internal (qRAP)
Tian *et al*, 2019^63^	China	R, CS	Single	533	Hospitalised surgical patients (undergoing thoracic surgery)	53	53%	0%	Caprini Khorana Padua Novel (Rogers *et al*, 2007)	VTE (NR)	8.4%	External
Bo *et al*, 2020^29^	China	P, CS	Multi	24 524	Hospitalised (≥2 days) patients from medical and surgical specialties aged ≥18 years	57	57	NR	Caprini	DVT (NR)	0.9%	External
Hu *et al*, 2020^42^	China	CC	Single	442	Hospitalised (≥2 days) cancer patients aged ≥18 years	NR	62	3.8	Caprini Khorana	VTE, defined by ICD-10 codes (NR)	NA	External
Mlaver *et al*, 2020^48^	USA	CC	Single	189	Hospitalised surgical patients (undergoing hepatobiliary, colorectal, endocrine, plastic, transplant or general surgery)	NR	NR	NR	Caprini Padua	VTE, not defined (NR)	NA	External
Moumneh *et al*, 2020^49^	France	R, CS *	Multi	14 660	Acutely ill, hospitalised (≥2 days) medical patients aged ≥40 years	73	50	46.1	Caprini Padua IMPROVE (7 factor)	VTE, symptomatic including PE or DVT (90 days)	1.8%	External
Nafee *et al*, 2020^50^	35 countries	R, CS *	Multi	6459	Hospitalised medical patients	76	45	100	IMPROVE (NR) Novel (Nafee *et al*, 2020a) Novel (Nafee *et al*, 2020b)	VTE (77 days)	6.3%	External
Shang *et al*, 2020^60^	China	CC	Single	2878	Hospitalised (≥2 days) cancer patients aged ≥18 years	56	47	NR	Caprini (2009) Caprini (2013)	VTE, (NR)	NA	External
Shen *et al*, 2020^61^	China	CC	Single	148	Hospitalised (≥2 days) medical patients aged ≥18 years	NR	NR	0	Novel (Shen *et al,* 2020)	VTE, not defined (NR)	NA	Internal: split (by time, months)
Wang *et al*, 2020^67^	China	CC	Single	1579	Hospitalised (≥3 days) medical patients aged ≥18 years	53	57	NR	Padua	VTE, (NR)	NA	Internal: split (by year, months)

*Prospective cohort study with retrospective analysis, thus classified as retrospective cohort study.

†Data overlap with Nendaz *et al*.^51^

ACS NSQIP, American College of Surgeons National Surgical Quality Improvement Program; CC, case-control; CS, cohort study; DVT, deep vein thrombosis; NA, not applicable; NR, not reported; P, prospective; PE, pulmonary embolism; R, retrospective; RAMs, risk assessment models; RAP, Risk Assessment Profile; TESS, Trauma Embolic Scoring System; VTE, venous thromboembolism.

The vast majority of studies evaluated VTE risk assessment in hospital inpatients who required medical care (n=21),^24 26–28 31 32 36 37 45 47 49–51 57 58 61 66 67 69–71^ were undergoing surgery (n=15)^23 25 33 35 38 40 43 46 48 52 56 59 63 65 68^ or were a mixed medical and surgical cohort (n=4).^22 29 30 34^ The remaining studies focused on patients receiving care for trauma (n=4),^39 41 55 62^ cancer (n=4),^21 42 54 60^ stroke (n=1),^44^ burn injuries (n=1)^53^ and sepsis (n=1).^64^ The mean age ranged from 45 years^39^ to 76 years^50^ (not reported in 29 studies)^22–25 30 31 33–35 38 40–45 47 48 52 55–58 61 62 65 66 70 71^ and the proportion of female subjects ranged from 17%^40^ to 81%^59^ (not reported in 12 studies).^22 23 25 33 35 43 48 52 55 56 58 61^

### VTE definition and case ascertainment

The majority of studies (n=37)^21 23 24 26–32 36–38 40–47 49–52 55–58 60 62–64 66 67 70 71^ defined the VTE endpoint (DVT and or PE) as being objectively confirmed. Of the remainder, 3 studies^34 54 69^ had no objective confirmation of VTE and 11 studies^22 25 33 35 39 48 53 59 61 65 68^ did not report the methods for diagnosis confirmation. In terms of VTE risk period, half of the studies (n=23)^21–26 28 35–38 40 43 44 47 49–52 56 57 59 69^ used the RAMs to predict the occurrence of VTE within 3 months of the index hospitalisation. The remaining studies did not report the VTE risk period. The reported incidence of VTE ranged widely from 0.3%^32 68^ to 27.9%,^46^ depending on definition, study design and study participants (eg, medical, surgical or trauma).

### RAMs

The studies included in this review evaluated 24 validated unique RAMs. The most widely evaluated models were the Caprini RAM (22 studies),^21 23 29–33 35 36 38 40 42 43 45 46 48 49 59 60 63 70 71^ Padua prediction score (16 studies),^24 27 28 30 31 34 37 45 48 49 63 64 66 67 70 71^ IMPROVE models (8 studies),^27 28 31 37 47 49 50 57^ the Geneva risk score (4 studies)^26–28 31^ and the Kucher score (4 studies).^31 37 66 69^ A summary of their associated characteristics and composite clinical variables is provided in online supplemental appendix S3.

### Statistical methods

Statistical methods varied significantly between studies. Most studies reported the discrimination of the RAMs using a combination of the C-statistic and sensitivity or specificity. A minority reported calibration measures, such as the Hosmer-Lemeshow test.^23 40 41 50^

### Risk of bias and applicability assessment

**Figure 2:**
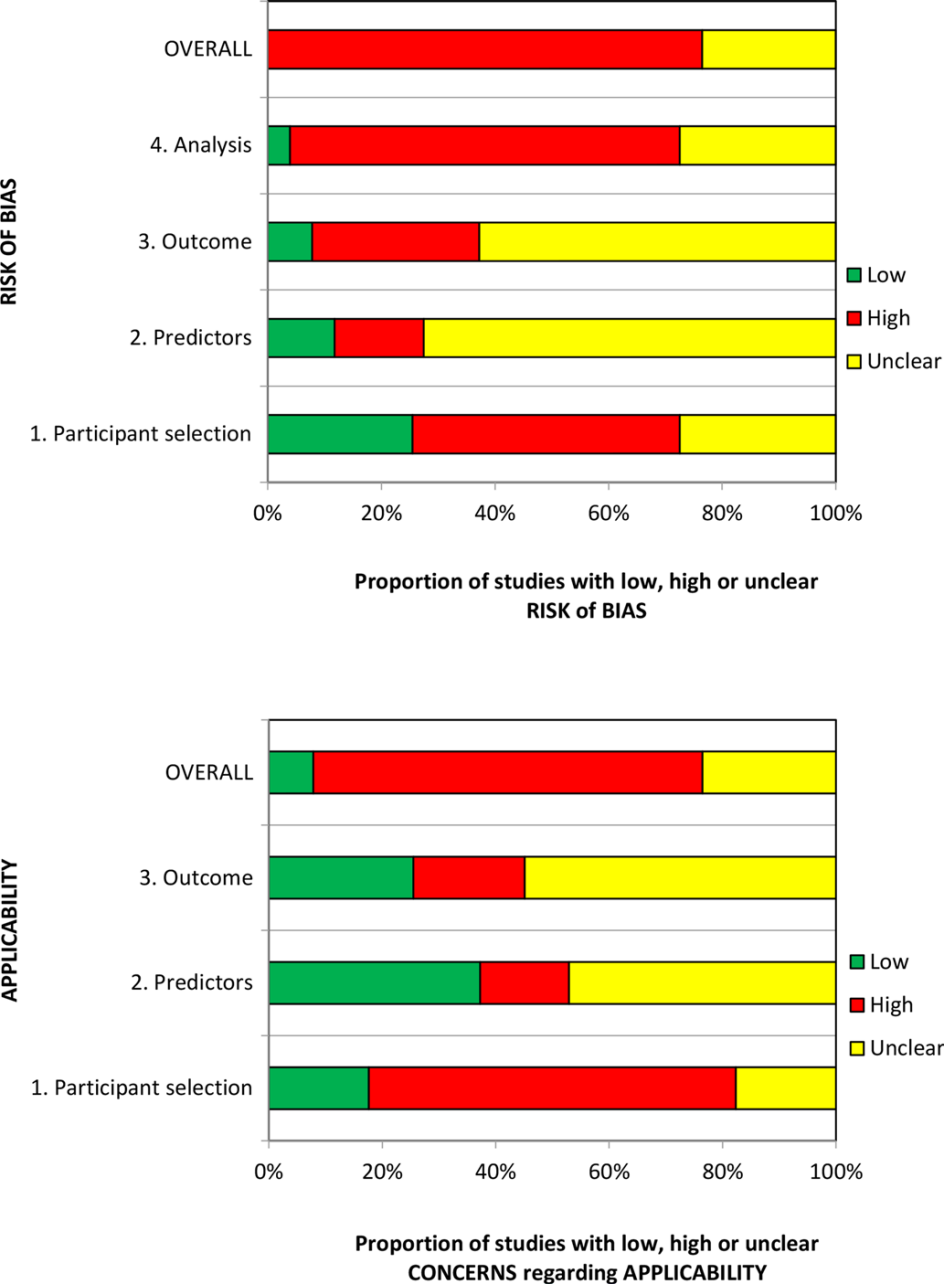
PROBAST (Prediction model Risk Of Bias ASsessment Tool) assessment summary graph—review authors’ judgements.

The overall methodological quality of the 51 included studies^21–71^ is summarised in table 2 and figure 2. The methodological quality of the included studies was variable, with most studies having high or unclear risk of bias in at least one item of the PROBAST tool. The main sources of potential bias were related to the following domains:

1. Patient selection factors, such as retrospective data collection, incomplete patient enrolment or unclear criteria for patients receiving VTE prophylaxis.
2. Predictor and outcome bias arising from inappropriate inclusion of predictors within RAMs, unclear methods of outcome definition, low event rates and missing predictor or outcome data.
3. Analysis factors, such as small sample sizes, inappropriate handling of missing data and failure in reporting relevant performance measures such as calibration.

**Table 2:** Summary of each study’s risk of bias and applicability concern using the PROBAST (Prediction model Risk Of Bias ASsessment Tool) tool—review authors’ judgements

Author, year	Risk of bias				Concern regarding applicability			Overall	Overall
	1. Participant selection	2. Predictors	3. Outcome	4. Analysis	1. Participant selection	2. Predictors	3. Outcomes	Risk of bias	Applicability
Abdel-Razeq *et al*, 2010^21^	High	High	High	High	High	High	High	High	High
Autar, 2003^22^	High	High	High	High	High	High	High	High	High
Bahl *et al*, 2010^23^	High	High	High	High	Unclear	Unclear	Unclear	High	Unclear
Barbar *et al*, 2010^24^	Low	Unclear	Unclear	High	Low	Unclear	Unclear	High	Unclear
Bilimoria *et al*, 2013^25^	Low	Low	Low	High	Low	Low	Low	High	Low
Blondon *et al*, 2019a^26^	Low	Unclear	High	High	Low	Low	Low	High	Low
Blondon *et al*, 2019b (abstract)^27^	Unclear	Unclear	Unclear	Unclear	Unclear	Unclear	Unclear	Unclear	Unclear
Blondon *et al*, 2018^28^	Low	Unclear	Unclear	High	Unclear	Low	Unclear	High	Unclear
Bo *et al*, 2020^29^	Low	Unclear	Unclear	Unclear	High	Low	Low	Unclear	High
Chen *et al*, 2018^30^	High	High	High	High	Unclear	High	High	High	High
Cobben *et al*, 2019^31^	Unclear	Unclear	High	High	Unclear	Low	Unclear	High	Unclear
de Bastos *et al*, 2016^32^	High	Low	High	High	High	Low	Low	High	High
Dornbus *et al*, 2018 (abstract)^33^	High	Unclear	High	Unclear	Unclear	Unclear	Unclear	High	Unclear
Elias *et al*, 2017^34^	High	Unclear	High	High	Low	Low	High	High	High
Frankel *et al*, 2017 (abstract)^35^	High	Unclear	Unclear	High	High	Unclear	Unclear	High	High
Grant *et al*, 2016^36^	High	Unclear	Unclear	Unclear	Low	Low	Low	High	Low
Greene *et al*, 2016^37^	Unclear	Unclear	Unclear	Unclear	Low	Low	Low	Unclear	Low
Hachey *et al*, 2016^38^	High	Unclear	Unclear	High	High	Low	High	High	High
Hegsted *et al*, 2013^39^	High	Unclear	High	High	High	Low	Unclear	High	High
Hewes *et al*, 2015^40^	High	Unclear	Unclear	High	High	Unclear	Low	High	High
Ho *et al*, 2014^41^	Unclear	Unclear	Unclear	High	High	Unclear	Unclear	High	High
Hu *et al*, 2020^42^	Unclear	Unclear	Unclear	Unclear	High	Unclear	Unclear	Unclear	High
Krasnow *et al*, 2017 (abstract)^43^	Unclear	Unclear	Unclear	Unclear	High	Unclear	Unclear	Unclear	High
Liu *et al*, 2014^44^	Low	Low	Unclear	Unclear	High	High	High	Unclear	High
Liu *et al*, 2016^45^	High	Unclear	High	High	High	Low	Low	High	High
Lobastov *et al*, 2016^46^	Unclear	Unclear	Unclear	High	High	Low	High	High	High
Mahan *et al*, 2014^47^	Low	Unclear	Unclear	Unclear	High	Low	Unclear	Unclear	High
Mlaver *et al*, 2020^48^	Unclear	Unclear	Unclear	Unclear	High	Unclear	Unclear	Unclear	High
Moumneh *et al*, 2020^49^	High	Unclear	Unclear	Low	High	Low	Low	High	High
Nafee *et al*, 2020^50^	Unclear	Low	Low	Low	Unclear	Low	Low	Unclear	Unclear
Nendaz *et al*, 2014^51^	Low	Unclear	Low	High	Low	Unclear	Low	High	Unclear
Pannucci *et al*, 2012^53^	High	Unclear	Unclear	High	High	High	Unclear	High	High
Pannucci *et al*, 2014^52^	Low	Unclear	High	High	High	Low	Low	High	High
Patell *et al*, 2017^54^	High	Unclear	Unclear	High	High	Unclear	Unclear	High	High
Rogers *et al*, 2007^56^	Unclear	Unclear	Unclear	High	Low	Unclear	Unclear	High	Unclear
Rogers *et al*, 2012^55^	High	High	Unclear	High	High	High	Unclear	High	High
Rosenberg *et al*, 2014^57^	Low	Unclear	Unclear	Unclear	Unclear	Unclear	Unclear	Unclear	Unclear
Rothberg *et al*, 2011^58^	High	Unclear	Unclear	High	Low	Unclear	Unclear	High	Unclear
Shaikh *et al*, 2016^59^	High	Unclear	High	High	High	Unclear	High	High	High
Shang *et al*, 2020^60^	Low	Unclear	Unclear	Unclear	High	Unclear	Unclear	Unclear	High
Shen *et al*, 2020^61^	Unclear	High	Unclear	Unclear	High	Unclear	Unclear	High	High
Tachino *et al*, 2019^62^	High	Unclear	Unclear	High	High	Unclear	Unclear	High	High
Tian *et al*, 2019^63^	High	Unclear	High	High	High	High	High	High	High
Vardi *et al*, 2013^64^	Unclear	Low	Low	High	High	Low	Low	High	High
Vaziri *et al*, 2018^65^	Unclear	Unclear	Unclear	High	High	Unclear	Unclear	High	High
Vincentelli *et al*, 2018^66^	High	Low	Unclear	High	High	Low	Unclear	High	High
Wang *et al*, 2020^67^	Low	Unclear	Unclear	Unclear	High	Unclear	Unclear	Unclear	High
Winoker *et al*, 2017^68^	High	Unclear	Unclear	High	High	High	High	High	High
Woller *et al*, 2011^69^	High	High	Unclear	High	Unclear	Unclear	Unclear	High	Unclear
Zhou *et al*, 2014^71^	Unclear	Unclear	Unclear	High	High	Unclear	Unclear	High	High
Zhou *et al*, 2018^70^	Low	High	High	High	High	Unclear	Unclear	High	High

Assessment of applicability to the review question led to the majority of studies being classed either as high (n=35)^21 22 29 30 32 34 35 38–49 52–55 59–68 70 71^ or unclear (n=12)^23 24 27 28 31 33 50 51 56–58 69^ risk of inapplicability. These assessments were generally related to patient selection (highly selected study populations, eg, single pathologies, single site settings), predictors (inconsistency in definition, assessment or timing of predictors) and outcome determination.

### Predictive performance of VTE RAMs (summary of results)

**Figure 3:**
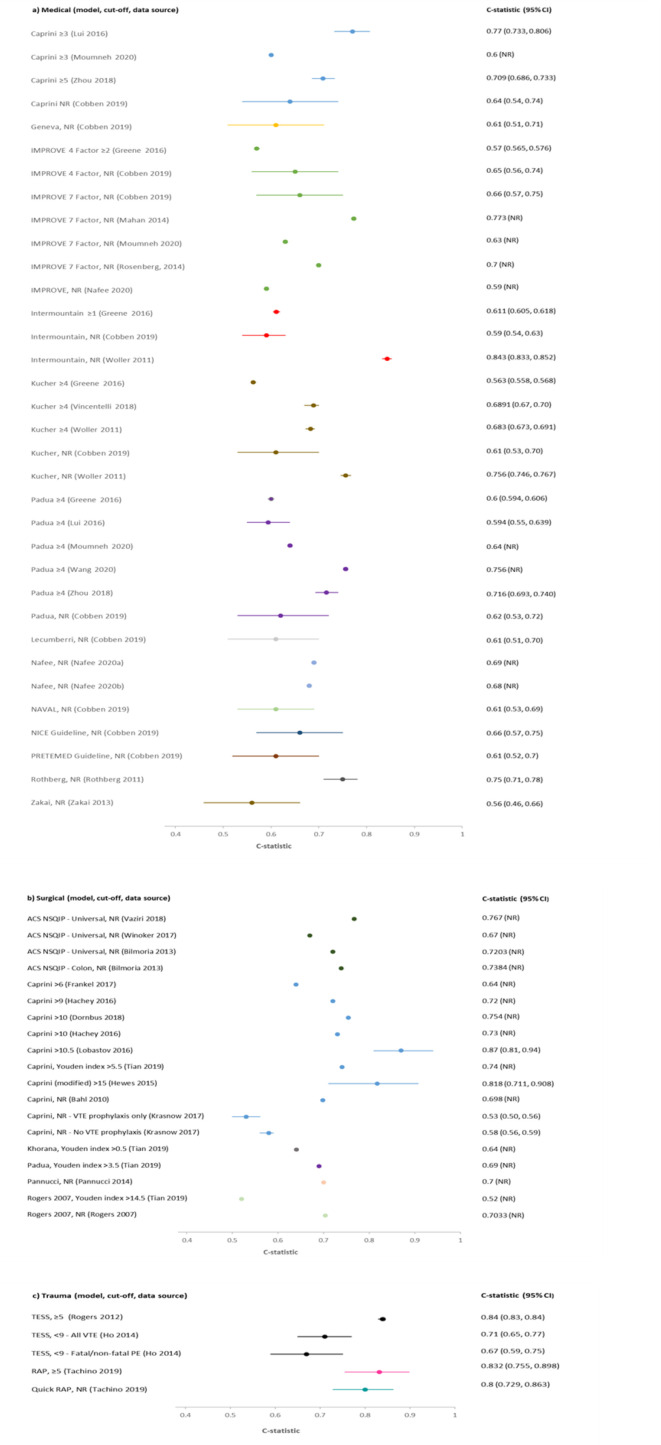
C-statistics by model for studies involving (a) medical, (b) surgical and (c) trauma inpatients. ACS NSQIP, American College of Surgeons National Surgical Quality Improvement Program; CI, confidence interval; DVT, deep vein thrombosis; NR, not reported; PE, pulmonary embolism; RAP, Risk Assessment Profile; TESS, Trauma Embolic Scoring System; VTE, venous thromboembolism.

As there were a reasonable number of studies to compare, a summary of the C-statistics for studies involving medical, surgical and trauma patients respectively is presented in figure 3a–c, with the results grouped by RAM. Results of other hospital inpatients are presented in online supplemental appendix S4. C-statistics varied markedly between these studies and between models, with no RAM performing obviously better than other models. In studies evaluating a single model, C-statistics^20^ were sometimes weak (<0.7; 10 studies with 17 data points), often good (0.7–0.8; 17 studies with 20 data points) and a few were excellent (>0.8; 5 studies with 5 data points). There was marked heterogeneity between multiple studies evaluating the same model. Studies evaluating multiple (more than 3) models^31 37^ tended to report weak accuracy across all the models (C-statistic <0.7; 2 studies with 16 data points).

Table 3 shows the sensitivity and specificity at various thresholds for studies involving medical, surgical and trauma patients respectively, with the results grouped by RAM. Interpretation was again limited by marked heterogeneity, which was exacerbated when different thresholds were reported by different studies evaluating the same model. Model accuracy was generally poor, with high sensitivity usually reflecting a threshold effect, as evidenced by corresponding low specificity (and vice versa).

**Table 3:** Sensitivity and specificity for studies involving medical, surgical and trauma inpatients

Risk assessment models	Threshold or cut-off	Endpoint	Data source	Sensitivity (95% CI)	Specificity (95% CI)
MEDICAL INPATIENTS					
Caprini (7 studies)	Risk score ≥3	VTE	Lui *et al*, 2016^45^	70.9% (NR)	73.4% (NR)
	Risk score ≥3	VTE	Moumneh *et al*, 2020^49^	98.1% (95.6 to 99.4)	7.5% (7.1 to 8.0)
	Risk score ≥3	VTE	Zhou *et al*, 2014^71^	82.3% (NR)	60.4% (NR)
	Risk score ≥3	VTE	Zhou *et al*, 2018^70^	84.3% (NR)	66.2% (NR)
	Risk score ≥5	VTE	Zhou *et al*, 2018^70^	57.1% (NR)	24.6% (NR)
	Risk score ≥5	VTE	Grant *et al*, 2016^36^	69.7% (NR)	50.28% (NR)
	Risk score ≥7	VTE	Grant *et al*, 2016^36^	42.69% (NR)	74.71% (NR)
	Risk score ≥9	VTE	Grant *et al*, 2016^36^	18.51% (NR)	89.03% (NR)
	NR*	VTE	de Bastos *et al*, 2016^32^	86.5% (NR)	47.0% (NR)
	NR	VTE	Cobben *et al*, 2019^31^	88.6% (NR)	21.4% (NR)
Chopard (1 study)	Risk score ≥3	VTE	Vincentelli *et al*, 2018^66^	64.2% (38.4 to 81.9)	57.7% (63.9 to 79.4)
Geneva models (4 studies)	Risk score ≥3	VTE	Blondon *et al*, 2018^28^; Nendaz *et al*, 2014^51^	All patients: 90.0% (73.5 to 97.9)	All patients: 35.3% (32.8 to 37.8)
				No prophylaxis: 85% (NR)	No prophylaxis: NR
	NR	VTE	Cobben *et al*, 2019^31^	75.0% (NR)	34.1% (NR)
	Simplified model: Risk score ≥3	VTE	Blondon *et al*, 2019a^26^	95.0% (NR)	44.0% (NR)
	Simplified model: NR	VTE	Blondon *et al*, 2019b (abstract)^27^	86.4% (NR)	NR
IMPROVE models (4 studies)	4-factor model: NR	VTE	Cobben *et al*, 2019^31^	27.9% (NR)	85.4% (NR)
	7-factor model: Risk score ≥2	VTE	Moumneh *et al*, 2020^49^	73.8% (68.0 to 79.0)	47.1% (46.3 to 47.9)
	7-factor model: Risk score 2–3	VTE	Blondon *et al*, 2018^28^; Nendaz *et al*, 2014^51^	All patients: 87% (NR)	All patients: NR
				No prophylaxis: 85% (NR)	No prophylaxis: NR
	7-factor model: Risk score ≥3	VTE	Blondon *et al*, 2018^28^; Nendaz *et al*, 2014^51^	All patients: 73% (NR)	All patients: NR
				No prophylaxis: 54% (NR)	No prophylaxis: NR
	7-factor model: Risk score ≥4	VTE	Moumneh *et al*, 2020^49^	24.7% (19.6 to 30.4)	85.5% (84.9 to 86.1)
	7-factor model: NR	VTE	Cobben *et al*, 2019^31^	63.3% (NR)	70.7% (NR)
	NR	VTE	Blondon *et al*, 2019b (abstract)^27^	57.6% (NR)	NR
Intermountain (1 study)	NR	VTE	Cobben *et al*, 2019^31^	26.4% (NR)	90.2% (NR)
Kucher (2 studies)	Risk Score ≥4	VTE	Vincentelli *et al*, 2018^66^	25.1% (17.0 to 55.1)	92.9% (81.0 to 95.4)
	NR	VTE	Cobben *et al*, 2019^31^	28.0% (NR)	85.7% (NR)
Lecumberri (1 study)	NR	VTE	Cobben *et al*, 2019^31^	61.6% (NR)	46.3% (NR)
NAVAL (1 study)	NR	VTE	Cobben *et al*, 2019^31^	19.0% (NR)	92.7% (NR)
NICE Guidelines (1 study)	NR	VTE	Cobben *et al*, 2019^31^	77.6% (NR)	39.0% (NR)
Padua (10 studies)	Risk score ≥4	VTE	Barbar *et al*, 2010^24^	94.6% (NR)	62.0% (NR)
	Risk score ≥4	VTE	Blondon *et al*, 2018^28^; Nendaz, 2014^51^	All patients: 73.3% (54.1 to 87.7)	All patients: 51.9% (49.3 to 54.5)
				No prophylaxis: 62% (NR)	No prophylaxis: NR
	Risk score ≥4	VTE	Lui *et al*, 2016^45^	23.4% (NR)	85.6% (NR)
	Risk score ≥4	VTE	Moumneh *et al*, 2020^49^	91.6% (87.6 to 94.7)	25.6% (24.9 to 26.3)
	Risk score ≥4	VTE	Zhou *et al*, 2014^71^	30.1% (NR)	12.7% (NR)
	Risk score ≥4	VTE	Zhou *et al*, 2018^70^	49.1% (NR)	16.2% (NR)
	Risk score ≥4	VTE	Vincentelli *et al*, 2018^66^	52.4% (38.4 to 81.9)	72.3% (63.9 to 79.4)
	Risk score ≥4	VTE	Wang *et al*, 2020^67^	76.2% (NR)	61.6% (NR)
	NR	VTE	Blondon *et al*, 2019b (abstract)^27^	72.7% (NR)	NR
	NR	VTE	Cobben *et al*, 2019^31^	61.8% (NR)	48.8% (NR)
PRETEMED guidelines (1 study)	NR	VTE	Cobben *et al*, 2019^31^	81.6% (NR)	24.4% (NR)
Shen 2020 (1 study)	NR	VTE	Shen *et al*, 2020^61^	77.8% (NR)	84.7% (NR)
Zakai 2013 (1 study)	Model 2: NR	VTE	Cobben *et al*, 2019^31^	63.8% (NR)	31.7% (NR)
SURGICAL INPATIENTS					
Caprini (8 studies)	Risk score >5	VTE	Hachey *et al*, 2016^38^	100% (100 to 100)	7.2% (4.1 to 11.0)
	Risk score ≥5	VTE	Mlaver *et al*, 2020^48^	88.9% (NR)	32.7% (NR)
	Risk score >5	VTE	Shaikh *et al*, 2016^59^	70.8% (48.9 to 87.4)	39.39% (37.0 to 41.9)
	Youden index >5.5	VTE	Tian *et al*, 2019^63^	76.0% (NR)	64.0% (NR)
	Risk score >6	VTE	Frankel *et al*, 2017 (abstract)^35^	61.5% (NR)	59.8% (NR)
	Risk score >6	VTE	Shaikh *et al*, 2016^59^	58.3% (36.6 to 77.9)	60.1% (57.6 to 62.5)
	Risk score >7	VTE	Hachey *et al*, 2016^38^	100% (100 to 100)	31.4% (25 to 37.3)
	Risk score >9	VTE	Hachey *et al*, 2016^38^	83.3% (58.3 to 100)	60.5% (54.4 to 67.3)
	Risk score >9	VTE	Shaikh *et al*, 2016^59^	16.7% (NR)	93.3% (NR)
	Risk score >10	VTE	Hachey *et al*, 2016^38^	75.0% (50 to 100)	69.6% (64.6 to 76.4)
	Risk score >10	VTE	Dornbus *et al*, 2018 (abstract)^33^	78.9% (NR)	60.9% (NR)
	Risk score >10.5	DVT or PE	Lobastov *et al*, 2016^46^	95.0% (NR)	73.0% (NR)
	Risk score >15 †	VTE	Hewes *et al*, 2015^40^	100% (100 to 100)	66.7% (55.0 to 78.3)
Khorana (1 study)	Youden index >0.5	VTE	Tian *et al*, 2019^63^	78.0% (NR)	48.0% (NR)
Padua (2 studies)	Risk score ≥4	VTE	Mlaver *et al*, 2020^48^	61.1% (NR)	47.4% (NR)
	Youden index >3.5	VTE	Tian *et al*, 2019^63^	36.0% (NR)	93.0% (NR)
Rogers 2007 (1 study)	Youden index >14.5	VTE	Tian *et al*, 2019^63^	53.0% (NR)	54.0% (NR)
TRAUMA PATIENTS					
RAP (2 studies)	Risk score ≥5	VTE	Tachino *et al*, 2019^62^	100% (86.8 to 100)	37.9% (34.6 to 41.3)
	Risk score 5 to ≤14	DVT or PE	Hegsted *et al*, 2013^39^	DVT: 82.0% (77 to 87) PE: 71.0% (55 to 86)	DVT: 57.0% (55 to 59) PE: 53.0% (51 to 56)
	Risk score >14	DVT or PE	Hegsted *et al*, 2013^39^	DVT: 15.0% (11 to 20) PE: 12.0% (1 to 23)	DVT: 97.0% (97 to 98) PE: 96.0% (95 to 97)
TESS (2 studies)	Risk score ≥5	VTE	Rogers *et al*, 2012^55^	77.4% (NR)	75.6% (NR)
	Risk score <9	VTE	Ho *et al*, 2014^41^	All VTE: 97.0% (91 to 99)	All VTE: 27.0% (22 to 32)
	Risk score <9	VTE	Ho *et al*, 2014^41^	Fatal and non-fatal PE: 97.0% (87 to 99)	Fatal and non-fatal PE: 24.0% (20 to 29)
	Risk score <9	VTE	Ho *et al*, 2014^41^	Fatal PE only: 100% (81 to 100)	Fatal PE only: 20.0% (13 to 28)

*Paper states ‘moderate and high risk’.

†Modified Caprini model.

DVT, deep vein thrombosis; NR, not reported; PE, pulmonary embolism; RAP, Risk Assessment Profile; TESS, Trauma Embolic Scoring System; VTE, venous thromboembolism.

## Discussion

### Summary of results

In this systematic review of 51 observational studies evaluating RAMs for predicting the risk of developing VTE in hospital inpatients, we found that VTE RAMs have generally weak predictive accuracy. The studies validating these models are heterogeneous and most have a high risk of bias. Lack of methodological clarity was common, leading to difficulty in assessing the applicability of the individual study results.

### Interpretation of results

We were unable to undertake meta-analysis or statistical examination of the causes of the observed heterogeneity. Potential sources of heterogeneity include variation in study design, the study population, how RAMs are implemented, outcome definition and measurement, and the use of thromboprophylaxis. The latter point warrants further attention. Thromboprophylaxis was employed in about half (n=25) of the studies,^21 22 24 26 28 30 34 36–38 40 42 44 46 49–52 54 57–59 64 70 71^ with the proportion receiving thromboprophylaxis ranging from 3.8%^42^ to 100%.^46 50^ It was not employed in 3 studies,^32 61 63^ and 23 studies^23 25 27 29 31 33 35 39 41 43 45 47 48 53 55 56 60 62 65–69^ did not report on thromboprophylaxis use. The use of thromboprophylaxis may lead to underestimation of predictive accuracy if a given RAM were to predict VTE events that were subsequently prevented by thromboprophylaxis. Limited reporting of thromboprophylaxis use precludes further analysis of its impact on the performance of the RAMs.

### Comparison to the existing literature

The present review is the largest and most comprehensive systematic review in this field to date. It includes 18 recent studies^26–31 33 42 48–50 60–63 66 67 70^ published since the completion of the previous systematic review.^10 12 13^ These studies are consistent with the previous literature in that they report modest performance of the assessed RAMs, with limitations in methodology and reporting preventing further analysis. The conclusion of this review therefore concurs with previous systematic reviews: there is insufficient evidence to recommend one RAM over another.

### Strengths and limitations

This systematic review has a number of strengths. The review was conducted with robust methodology in accordance with the PRISMA statement and the protocol was registered with the PROSPERO register. Clinical experts were involved throughout as checkers and to assess the validity and applicability of research during the review. We reported descriptive statistics to provide insight into the limited evidence base applicable to the subject matter, and the scientific concerns regarding validity of the data. However, there are a number of potential weaknesses. Decisions on study relevance, information gathering and validity were unblinded and could potentially have been influenced by pre-formed opinions. However, masking is resource intensive with uncertain benefits. The studies of risk prediction were a combination of prospective cohorts and retrospective health database registries. Both have significant limitations. Retrospective studies of health database registries may have large numbers but may be limited by poor data quality and failure to accurately ascertain outcomes. Prospective cohorts may have better quality data but with smaller numbers lack statistical power. The included studies demonstrated high levels of heterogeneity so we were unable to undertake any meta-analysis.

### Implications for policy, practice and future research

Guidelines from the American College of Chest Physicians (ACCP)^72 73^ and the UK National Institute for Health and Care Excellence (NICE)^10^ suggest using a validated RAM to guide the decision on whether to prescribe thromboprophylaxis. This review identifies all relevant RAMs and their validation studies. The reported results are insufficient to recommend one RAM over another. A RAM with weak predictive accuracy may still be better than no RAM at all but it is unclear whether RAMs predict VTE risk better than unstructured clinical assessment. Further research is clearly needed but routine use of thromboprophylaxis may present an insurmountable barrier to generating accurate and precise estimates of the prognostic accuracy of RAMs. The evidence that thromboprophylaxis is effective means that it is unethical to withhold thromboprophylaxis when a significant risk of VTE is identified. This inevitably reduces the number of VTE events in any study and confounds the association between risk factors and VTE events. Further studies of RAM accuracy will add little to our review unless they can address this issue.

Alternative approaches therefore need to be considered. Decision-analytic modelling can use existing data to explore the trade-off between the benefits and harms of thromboprophylaxis and identify key uncertainties for future primary research. The data presented in our review show how well RAMs predict VTE but do not tell us the threshold score on the RAM at which thromboprophylaxis should be given to maximise prevention of VTE and minimise harm from bleeding. This may be a more important determinant of RAM effectiveness than predictive accuracy for VTE. Le *et al*^74^ suggested thromboprophylaxis is beneficial and cost-effective if a patient’s VTE risk exceeds 1%. Further work to improve RAMs to help stratify the risk of VTE in different types of hospitalised patients could focus on using decision-analytic modelling to compare the effects, harms and costs of giving thromboprophylaxis to patients with varying risk of VTE. This would allow determination of the risk threshold at which thromboprophylaxis provides optimal overall benefit.

Findings from decision-analytic modelling would require validation through primary research. The limitations of undertaking accuracy studies in populations where thromboprophylaxis is routinely used mean that future research should focus on research that compares the effectiveness of different risk assessment approaches. Observational studies could draw on variation in practice to compare outcomes between different risk assessment methods. Alternatively, a controlled trial could compare risk assessment methods in low-risk patients where existing evidence (synthesised using decision-analytic modelling) suggests the benefits of thromboprophylaxis are uncertain.

## Conclusions

We identified a number of validated RAMs for potential risk stratification of hospitalised inpatients. The available evidence is insufficient to recommend one over another.

## Supplementary Material

Reviewer comments

Author's manuscript

## Data Availability

All data relevant to the study are included in the article or uploaded as supplementary information.

## References

[1] Goldhaber SZ, Visani L, De Rosa M. Acute pulmonary embolism: clinical outcomes in the International cooperative pulmonary embolism registry (ICOPER). Lancet 1999;353:1386–9. 10.1016/S0140-6736(98)07534-5 10227218

[2] Prandoni P, Lensing AW, Cogo A, et al. The long-term clinical course of acute deep venous thrombosis. Ann Intern Med 1996;125:1–7. 10.7326/0003-4819-125-1-199607010-00001 8644983

[3] ISTH Steering Committee for world thrombosis day. Thrombosis: a major contributor to the global disease burden. J Thromb Haemost 2014;12. 10.1111/jth.12698 25302663

[4] Jha AK, Larizgoitia I, Audera-Lopez C, et al. The global burden of unsafe medical care: Analytic modelling of observational studies. BMJ Qual Saf 2013;22:809–15. 10.1136/bmjqs-2012-001748 24048616

[5] Alikhan R, Bedenis R, Cohen AT. Heparin for the prevention of venous thromboembolism in acutely ill medical patients (excluding stroke and myocardial infarction). Cochrane Database Syst Rev 2014;2014:Cd003747. 10.1002/14651858.CD003747.pub4 PMC6491079 24804622

[6] Dentali F, Douketis JD, Gianni M, et al. Meta-Analysis: anticoagulant prophylaxis to prevent symptomatic venous thromboembolism in hospitalized medical patients. Ann Intern Med 2007;146:278–88. 10.7326/0003-4819-146-4-200702200-00007 17310052

[7] Kahn SR, Diendéré G, Morrison DR, et al. Effectiveness of interventions for the implementation of thromboprophylaxis in hospitalised patients at risk of venous thromboembolism: an updated abridged Cochrane systematic review and meta-analysis of randomised controlled trials. BMJ Open 2019;9:e024444. 10.1136/bmjopen-2018-024444 PMC6537979 31129575

[8] Lloyd NS, Douketis JD, Moinuddin I, et al. Anticoagulant prophylaxis to prevent asymptomatic deep vein thrombosis in hospitalized medical patients: a systematic review and meta-analysis. J Thromb Haemost 2008;6:405–14. 10.1111/j.1538-7836.2007.02847.x 18031292

[9] Henke PK, Kahn SR, Pannucci CJ, et al. Call to action to prevent venous thromboembolism in hospitalized patients: a policy statement from the American heart association. Circulation 2020;141:e914–31. 10.1161/CIR.0000000000000769 32375490

[10] NICE. Venous thromboembolism in over 16s:reducing the risk of hospital-acquired deep vein thrombosis or pulmonary embolism. London, UK: National Institute for Health and Care Excellence, 2018. https://www.nice.org.uk/guidance/NG89 29697228

[11] Chan NC, Gross PL, Weitz JI. Addressing the burden of hospital-related venous thromboembolism: the role of extended anticoagulant prophylaxis. J Thromb Haemost 2018;16:413–7. 10.1111/jth.13942 29480565

[12] Huang W, Anderson FA, Spencer FA, et al. Risk-assessment models for predicting venous thromboembolism among hospitalized non-surgical patients: a systematic review. J Thromb Thrombolysis 2013;35:67–80. 10.1007/s11239-012-0780-0 22826096

[13] Stuck AK, Spirk D, Schaudt J, et al. Risk assessment models for venous thromboembolism in acutely ill medical patients. A systematic review. Thromb Haemost 2017;117:801–8. 10.1160/TH16-08-0631 28150851

[14] Moher D, Liberati A, Tetzlaff J, et al. Preferred reporting items for systematic reviews and meta-analyses: the PRISMA statement. Ann Intern Med 2009;151:264–9. w64. 10.7326/0003-4819-151-4-200908180-00135 19622511

[15] Goodacre S, Hogg K, Griffin X, et al. The cost-effectiveness of venous thromboembolism risk assessment tools for hospital inpatients. UK: National Institute of Health Research UK, 2019.

[16] Moons KGM, Wolff RF, Riley RD, et al. PROBAST: a tool to assess risk of bias and applicability of prediction model studies: explanation and elaboration. Ann Intern Med 2019;170:W1–33. 10.7326/M18-1377 30596876

[17] Wolff RF, Moons KGM, Riley RD, et al. PROBAST: a tool to assess the risk of bias and applicability of prediction model studies. Ann Intern Med 2019;170:51–8. 10.7326/M18-1376 30596875

[18] Centre for Reviews and Dissemination. Systematic reviews: CRD’s guidance for undertaking reviews in health care. York, 2009.

[19] McKenzie JE, Brennan SE, Ryan RE. Chapter 9: Summarizing study characteristics and preparing for synthesis. In: Cochrane Handbook for systematic reviews of interventions version 6.2 updated February 2021. Cochrane, 2021.

[20] Hosmer DW, Lemeshow S. Applied logistic regression. 2 edn. New York: John Wiley & Sons, 2000.

[21] Abdel-Razeq HN, Hijjawi SB, Jallad SG, et al. Venous thromboembolism risk stratification in medically-ill hospitalized cancer patients. A comprehensive cancer center experience. J Thromb Thrombolysis 2010;30:286–93. 10.1007/s11239-010-0445-9 20127272

[22] Autar R. The management of deep vein thrombosis: the Autar DVT risk assessment scale re-visited. J Orthop Nurs 2003;7:114–24. 10.1016/S1361-3111(03)00051-7

[23] Bahl V, Hu HM, Henke PK, et al. A validation study of a retrospective venous thromboembolism risk scoring method. Ann Surg 2010;251:344–50. 10.1097/SLA.0b013e3181b7fca6 19779324

[24] Barbar S, Noventa F, Rossetto V, et al. A risk assessment model for the identification of hospitalized medical patients at risk for venous thromboembolism: the Padua prediction score. J Thromb Haemost 2010;8:2450–7. 10.1111/j.1538-7836.2010.04044.x 20738765

[25] Bilimoria KY, Liu Y, Paruch JL, et al. Development and evaluation of the universal ACS NSQIP surgical risk calculator: a decision aid and informed consent tool for patients and surgeons. J Am Coll Surg 2013;217:833–42. 10.1016/j.jamcollsurg.2013.07.385 24055383 PMC3805776

[26] Blondon M, Righini M, Nendaz M. External validation of the simplified Geneva risk assessment model for hospital-associated venous thromboembolism in the Padua cohort. J Thromb Haemost 2020;18:676–80. 10.1111/jth.14688 31782886

[27] Blondon M, Limacher A, Righini M, et al. Adequacy of hospital thromboprophylaxis and risk assessment models in the SWITCO65+ cohort. Res Pract Thromb Haemost 2019;3:760. 10.1002/rth2.12361 PMC7845057 33537538

[28] Blondon M, Spirk D, Kucher N, et al. Comparative performance of clinical risk assessment models for hospital-acquired venous thromboembolism in medical patients. Thromb Haemost 2018;118:82–9. 10.1160/TH17-06-0403 29304528

[29] Bo H, Li Y, Liu G, et al. Assessing the risk for development of deep vein thrombosis among Chinese patients using the 2010 Caprini risk assessment model: a prospective multicenter study. J Atheroscler Thromb 2020;27:801–8. 10.5551/jat.51359 31852858 PMC7458789

[30] Chen X, Pan L, Deng H, et al. Risk assessment in Chinese hospitalized patients comparing the Padua and Caprini scoring algorithms. Clin Appl Thromb Hemost 2018;24:127S–35. 10.1177/1076029618797465 30198321 PMC6714840

[31] Cobben MRR, Nemeth B, Lijfering WM, et al. Validation of risk assessment models for venous thrombosis in hospitalized medical patients. Res Pract Thromb Haemost 2019;3:217–25. 10.1002/rth2.12181

[32] de Bastos M, Barreto SM, Caiafa JS, et al. Derivation of a risk assessment model for hospital-acquired venous thrombosis: the naval score. J Thromb Thrombolysis 2016;41:628–35. 10.1007/s11239-015-1277-4 26446587

[33] Dornbos DL, Shah V, Priddy B. Predicting venous thromboembolic complications following neurological surgery procedures. J Neurosurg 2018;128:55.

[34] Elias P, Khanna R, Dudley A, et al. Automating venous thromboembolism risk calculation using electronic health record data upon hospital admission: the automated Padua prediction score. J Hosp Med 2017;12:231–7. 10.12788/jhm.2714 28411291

[35] Frankel MD JK, Belanger M, Tortora BA J, et al. MP47-17 CAPRINI score predicts venous thromboembolic events in patients undergoing robotic assisted prostatectomy. J Urol 2017;197:e635. 10.1016/j.juro.2017.02.1477

[36] Grant PJ, Greene MT, Chopra V, et al. Assessing the Caprini score for risk assessment of venous thromboembolism in hospitalized medical patients results presented at: the Society of hospital medicine annual meeting, March 26, 2014, Las Vegas, Nevada. Am J Med 2016;129:528–35. 10.1016/j.amjmed.2015.10.027 26551977 PMC5331485

[37] Greene MT, Spyropoulos AC, Chopra V, et al. Validation of risk assessment models of venous thromboembolism in hospitalized medical patients. Am J Med 2016;129:1001.e9–1001.e18. 10.1016/j.amjmed.2016.03.031 27107925

[38] Hachey KJ, Hewes PD, Porter LP, et al. Caprini venous thromboembolism risk assessment permits selection for postdischarge prophylactic anticoagulation in patients with resectable lung cancer. J Thorac Cardiovasc Surg 2016;151:37–44. 10.1016/j.jtcvs.2015.08.039 26386868

[39] Hegsted D, Gritsiouk Y, Schlesinger P, et al. Utility of the risk assessment profile for risk stratification of venous thrombotic events for trauma patients. Am J Surg 2013;205:517–20. 10.1016/j.amjsurg.2013.01.022 23592157

[40] Hewes PD, Hachey KJ, Zhang XW, et al. Evaluation of the Caprini model for Venothromboembolism in esophagectomy patients. Ann Thorac Surg 2015;100:2072–8. 10.1016/j.athoracsur.2015.05.098 26279363

[41] Ho KM, Rao S, Rittenhouse KJ, et al. Use of the trauma embolic scoring system (Tess) to predict symptomatic deep vein thrombosis and fatal and non-fatal pulmonary embolism in severely injured patients. Anaesth Intensive Care 2014;42:709–14. 10.1177/0310057X1404200605 25342402

[42] Hu Y, Li X, Zhou H, et al. Comparison between the Khorana prediction score and Caprini risk assessment models for assessing the risk of venous thromboembolism in hospitalized patients with cancer: a retrospective case control study. Interact Cardiovasc Thorac Surg 2020;31:454–60. 10.1093/icvts/ivaa137 32910201

[43] Krasnow R, Preston M, Chung B. Validation of venous thromboembolism risk assessment score in major urologic cancer surgery: a population based study. J Urol 2017;197:e1126.

[44] Liu L-P, Zheng H-G, Wang DZ, et al. Risk assessment of deep-vein thrombosis after acute stroke: a prospective study using clinical factors. CNS Neurosci Ther 2014;20:403–10. 10.1111/cns.12227 24612485 PMC6493054

[45] Liu X, Liu C, Chen X, et al. Comparison between Caprini and Padua risk assessment models for hospitalized medical patients at risk for venous thromboembolism: a retrospective study. Interact Cardiovasc Thorac Surg 2016;23:538–43. 10.1093/icvts/ivw158 27297558

[46] Lobastov K, Barinov V, Schastlivtsev I, et al. Validation of the Caprini risk assessment model for venous thromboembolism in high-risk surgical patients in the background of standard prophylaxis. J Vasc Surg Venous Lymphat Disord 2016;4:153–60. 10.1016/j.jvsv.2015.09.004 26993860

[47] Mahan CE, Liu Y, Turpie AG, et al. External validation of a risk assessment model for venous thromboembolism in the hospitalised acutely-ill medical patient (VTE-VALOURR). Thromb Haemost 2014;112:692–9. 10.1160/TH14-03-0239 24990708

[48] Mlaver E, Lynde GC, Gallion C, et al. Development of a novel preoperative venous thromboembolism risk assessment model. Am Surg 2020;86:1098–105. 10.1177/0003134820943556 32967431

[49] Moumneh T, Riou J, Douillet D, et al. Validation of risk assessment models predicting venous thromboembolism in acutely ill medical inpatients: a cohort study. J Thromb Haemost 2020;18:1398–407. 10.1111/jth.14796 32168402

[50] Nafee T, Gibson CM, Travis R, et al. Machine learning to predict venous thrombosis in acutely ill medical patients. Res Pract Thromb Haemost 2020;4:230–7. 10.1002/rth2.12292 32110753 PMC7040551

[51] Nendaz M, Spirk D, Kucher N, et al. Multicentre validation of the Geneva risk score for hospitalised medical patients at risk of venous thromboembolism. explicit assessment of thromboembolic risk and prophylaxis for medical patients in Switzerland (estimate). Thromb Haemost 2014;111:531–8. 10.1160/TH13-05-0427 24226257

[52] Pannucci CJ, Laird S, Dimick JB, et al. A validated risk model to predict 90-day VTe events in postsurgical patients. Chest 2014;145:567–73. 10.1378/chest.13-1553 24091567 PMC4502716

[53] Pannucci CJ, Osborne NH, Wahl WL. Creation and validation of a simple venous thromboembolism risk scoring tool for thermally injured patients: analysis of the National burn Repository. J Burn Care Res 2012;33:20–5. 10.1097/BCR.0b013e318234d8b5 21979848 PMC3253918

[54] Patell R, Rybicki L, McCrae KR, et al. Predicting risk of venous thromboembolism in hospitalized cancer patients: utility of a risk assessment tool. Am J Hematol 2017;92:501–7. 10.1002/ajh.24700 28240823 PMC5729904

[55] Rogers FB, Shackford SR, Horst MA, et al. Determining venous thromboembolic risk assessment for patients with trauma: the trauma embolic scoring system. J Trauma Acute Care Surg 2012;73:511–5. 10.1097/ta.0b013e3182588b54 23019680

[56] Rogers SO, Kilaru RK, Hosokawa P, et al. Multivariable predictors of postoperative venous thromboembolic events after general and vascular surgery: results from the patient safety in surgery study. J Am Coll Surg 2007;204:1211–21. 10.1016/j.jamcollsurg.2007.02.072 17544079

[57] Rosenberg D, Eichorn A, Alarcon M, et al. External validation of the risk assessment model of the International medical prevention registry on venous thromboembolism (improve) for medical patients in a tertiary health system. J Am Heart Assoc 2014;3:e001152. 10.1161/JAHA.114.001152 25404191 PMC4338701

[58] Rothberg MB, Lindenauer PK, Lahti M, et al. Risk factor model to predict venous thromboembolism in hospitalized medical patients. J Hosp Med 2011;6:202–9. 10.1002/jhm.888 21480491

[59] Shaikh M-A, Jeong HS, Mastro A, et al. Analysis of the American Society of Anesthesiologists physical status classification system and Caprini risk assessment model in predicting venous thromboembolic outcomes in plastic surgery patients. Aesthet Surg J 2016;36:497–505. 10.1093/asj/sjv198 26673574

[60] Shang M-M, Yan R, Wang X-L, et al. Comparison of 2013 and 2009 versions of Caprini risk assessment models for predicting VTe in Chinese cancer patients: a retrospective study. J Thromb Thrombolysis 2020;50:446–51. 10.1007/s11239-020-02038-2 31975322

[61] Shen C, Ge B, Liu X, et al. Predicting the occurrence of venous thromboembolism: construction and verification of risk warning model. BMC Cardiovasc Disord 2020;20:249. 10.1186/s12872-020-01519-9 32460701 PMC7251685

[62] Tachino J, Yamamoto K, Shimizu K, et al. Quick risk assessment profile (qRAP) is a prediction model for post-traumatic venous thromboembolism. Injury 2019;50:1540–4. 10.1016/j.injury.2019.06.020 31248583

[63] Tian B, Li H, Cui S, et al. A novel risk assessment model for venous thromboembolism after major thoracic surgery: a Chinese single-center study. J Thorac Dis 2019;11:1903–10. 10.21037/jtd.2019.05.11 31285883 PMC6588735

[64] Vardi M, Ghanem-Zoubi NO, Zidan R, et al. Venous thromboembolism and the utility of the Padua prediction score in patients with sepsis admitted to internal medicine departments. J Thromb Haemost 2013;11:467–73. 10.1111/jth.12108 23279085

[65] Vaziri S, Wilson J, Abbatematteo J, et al. Predictive performance of the American College of surgeons universal risk calculator in neurosurgical patients. J Neurosurg 2018;128:942–7. 10.3171/2016.11.JNS161377 28452615

[66] Vincentelli GM, Timpone S, Murdolo G, et al. A new risk assessment model for the stratification of the thromboembolism risk in medical patients: the TEVere score. Minerva Med 2018;109:436–42. 10.23736/S0026-4806.18.05689-6 29856190

[67] Wang X, Yang Y-Q, Liu S-H, et al. Comparing different venous thromboembolism risk assessment machine learning models in Chinese patients. J Eval Clin Pract 2020;26:26–34. 10.1111/jep.13324 31840330

[68] Winoker JS, Paulucci DJ, Anastos H, et al. Predicting Complications Following Robot-Assisted Partial Nephrectomy with the ACS NSQIP^®^ Universal Surgical Risk Calculator. J Urol 2017;198:803–9. 10.1016/j.juro.2017.04.021 28400189

[69] Woller SC, Stevens SM, Jones JP, et al. Derivation and validation of a simple model to identify venous thromboembolism risk in medical patients. Am J Med 2011;124:947–54. 10.1016/j.amjmed.2011.06.004 21962315

[70] Zhou H, Hu Y, Li X, et al. Assessment of the risk of venous thromboembolism in medical inpatients using the Padua prediction score and Caprini risk assessment model. J Atheroscler Thromb 2018;25:1091–104. 10.5551/jat.43653 29540637 PMC6224205

[71] Zhou H, Wang L, Wu X, et al. Validation of a venous thromboembolism risk assessment model in hospitalized Chinese patients: a case-control study. J Atheroscler Thromb 2014;21:261–72. 10.5551/jat.20891 24304962

[72] Kahn SR, Lim W, Dunn AS, et al. Prevention of VTe in nonsurgical patients: antithrombotic therapy and prevention of thrombosis, 9th ed: American College of chest physicians evidence-based clinical practice guidelines. Chest 2012;141:e195S–226. 10.1378/chest.11-2296 22315261 PMC3278052

[73] Gould MK, Garcia DA, Wren SM, et al. Prevention of VTe in nonorthopedic surgical patients: antithrombotic therapy and prevention of thrombosis, 9th ED: American College of chest physicians evidence-based clinical practice guidelines. Chest 2012;141:e227S–77. 10.1378/chest.11-2297 22315263 PMC3278061

[74] Le P, Martinez KA, Pappas MA, et al. A decision model to estimate a risk threshold for venous thromboembolism prophylaxis in hospitalized medical patients. J Thromb Haemost 2017;15:1132–41. 10.1111/jth.13687 28371250 PMC5712445

