# Supplementary material for: Risk assessment models for venous thromboembolism in hospitalised adult patients: a systematic review
Source: BMJ Open. 2021 Jul 29;11(7):e045672. doi: 10.1136/bmjopen-2020-045672 (PMC8323381; doi:10.1136/bmjopen-2020-045672)
Supplement: Supplementary data [file bmjopen-2020-045672supp001.pdf]

**ONLINE SUPPLEMENTARY**

**APPENDIX S1: LITERATURE SEARCH STRATEGY**

**Table S1 Literature search strategy for the review of RAMs for VTE in hospital inpatients**

| | |
|---|---|
| **Database searched:** | **Ovid MEDLINE(R) Epub Ahead of Print, In-Process & Other Non-Indexed Citations, Ovid MEDLINE(R) Daily, Ovid MEDLINE and Versions(R)** |
| **Platform or provider used:** | **Ovid SP** |
| **Date of coverage:** | **1946 to February 2021** |
| **Search undertaken:** | **February 2021** |

1 pulmonary embolism/ or thromboembolism/ or venous thromboembolism/ or venous thrombosis/ or upper extremity deep vein thrombosis/
2 (((venous or vein) adj (thrombosis or thromboses or thrombus or thromboembolism)) or (dvt or vte) or ((pulmonary or lung) adj3 (embolism or emboli or embolus or emboliz* or thromboembolism))).ti,ab.
3 1 or 2
4 letter/ or editorial/ or news/ or exp historical article/ or anecdotes as topic/ or comment/ or case report/ or (letter or comment).ti.
5 randomized controlled trial/ or random*.ti,ab.
6 4 not 5
7 animals/ not humans/
8 exp animals, laboratory/
9 exp animal experimentation/
10 exp models, animal/
11 exp rodentia/
12 (rat or rats or mouse or mice).ti.
13 6 or 7 or 8 or 9 or 10 or 11 or 12
14 3 not 13
15 (risk* adj2 assess*).ti,ab.
16 ((score* or scoring) adj2 (tool* or system*)).ti,ab.
17 ((risk* or predict* or prognos*) adj4 (tool* or rule* or index* or indices or score* or scoring or scale* or model* or system* or algorithm* or stratif* or criteria or calculat*)).ti,ab.
18 (vienna adj5 cats).ti,ab.
19 (vienna cancer and thrombosis study).ti,ab.
20 trauma embolic scoring.ti,ab.
21 tess.ti,ab.
22 (roger* or caprini* or kucher* or cohen* or padua* or khorana* or autar).ti,ab.
23 (well* adj2 (score* or scoring)).ti,ab.
24 department of health.ti,ab,au.
25 or/15-24
26 14 and 25
27 limit 26 to yr="2017 -Current"

| | |
|---|---|
| **Databases searched:** | **EMBASE** |
| **Platform or provider used:** | **Ovid SP** |
| **Date of coverage:** | **1974 to February 2021** |
| **Search undertaken:** | **February 2021** |

1 thromboembolism/ or venous thromboembolism/ or vein thrombosis/ or deep veinthrombosis/ or leg thrombosis/ or lower extremity deep vein thrombosis/ or postoperativethrombosis/ or lung embolism/ or upper extremity deep vein thrombosis/
2 (((venous or vein) adj (thrombosis or thromboses or thrombus or thromboembolism)) or (dvt or vte) or ((pulmonary or lung) adj3 (embolism or emboli or embolus or emboliz* or thromboembolism))).ti,ab.
3 1 or 2
4 letter.pt. or letter/ or note.pt. or editorial.pt. or case report/ or case study/ or (letter or comment*).ti.
5 randomized controlled trial/ or random*.ti,ab.
6 4 not 5
7 animal/ not human/
8 nonhuman/
9 exp animal experiment/ or exp experimental animal/
10 animal model/
11 exp rodent/
12 (rat or rats or mouse or mice).ti.
13 6 or 7 or 8 or 9 or 10 or 11 or 12
14 3 not 13
15 (risk* adj2 assess*).ti,ab.
16 ((score* or scoring) adj2 (tool* or system*)).ti,ab.
17 ((risk* or predict* or prognos*) adj4 (tool* or rule* or index* or indices or score* or scoring or scale* or model* or system* or algorithm* or stratif* or criteria or calculat*)).ti,ab.
18 (vienna adj5 cats).ti,ab.
19 (vienna cancer and thrombosis study).ti,ab.
20 trauma embolic scoring.ti,ab.
21 tess.ti,ab.
22 (roger* or caprini* or kucher* or cohen* or padua* or khorana* or autar).ti,ab.
23 (well* adj2 (score* or scoring)).ti,ab.
24 department of health.ti,ab,au.
25 or/15-24
26 14 and 25
27 limit 26 to yr="2017 -Current"

| | |
|---|---|
| **Databases searched:** | **Cochrane CENTRAL Register of Randomised Controlled Trials & Cochrane Database of Systematic Reviews** |
| **Platform or provider used:** | **www.thecochranelibrary.com** |
| **Date of coverage:** | **Inception to February 2021** |
| **Search undertaken:** | **February 2021** |

#1. MeSH descriptor: [venous thromboembolism] this term only

#2. MeSH descriptor: [pulmonary embolism] this term only

#3. MeSH descriptor: [venous thrombosis] this term only

#4. MeSH descriptor: [thromboembolism] this term only

#5. MeSH descriptor: [upper extremity deep vein thrombosis] this term only

#6. ((*venous or *vein) next (thrombosis or thromboses or thrombus or thromboembolism) or dvt or vte or (pulmonary or lung) near/3 (embolism or emboli or embolus or emboliz* or thromboembolism)):ti,ab

#7. #1 or #2 or #3 or #4 or #6

#8. (risk* near/2 assess*):ti,ab

#9. ((score* or scoring) near/2 (tool* or system*)):ti,ab

#10. ((risk* or predict* or prognos*) near/4 (tool* or rule* or index* or indices or score* or scoring or scale* or model* or system* or algorithm* or stratif* or criteria or calculat*)):ti,ab

#11. (vienna near/5 cats):ti,ab

#12. (vienna cancer and thrombosis study):ti,ab

#13. trauma embolic scoring:ti,ab

#14. tess:ti,ab

#15. (roger* or caprini* or kucher* or cohen* or padua* or khorana* or autar):ti,ab

#16. (well* near/2 (score* or scoring)):ti,ab

#17. (department of health):ti,ab

#18. (or #8-#17)

#19. #7 and #18 with Publication Year from 2017 to 21

## APPENDIX S2: LIST OF EXCLUDED STUDIES WITH RATIONALE

| | Authors, year | Reason for exclusion |
|---|---|---|
| 1. | Alikhan et al., 2004 [1] | Derivation only |
| 2. | Alper et al., 2018 [2] | Not a RAM for predicting the risk of developing VTE in hospital inpatients |
| 3. | Arcelus et al., 1991 [3] | No relevant/useable outcome data |
| 4. | Arpaia et al., 2020 [4] | Not a RAM for predicting the risk of developing VTE in hospital inpatients |
| 5. | Bagot et al., 2010 [5] | No relevant/useable outcome data |
| 6. | Bellizzi et al., 2018 [6] | No relevant/useable outcome data |
| 7. | Blondon et al., 2020 [7] | Duplicate of included study (Blondon et al., 2019a) |
| 8. | Blondon et al., 2017 [8] | Abstract of an included full-text study |
| 9. | Caprini et al., 2001 [9] | Review |
| 10. | Chen et al., 2018 [10] | Not available (foreign language) |
| 11. | Chopard et al., 2006 [11] | No relevant/useable outcome data |
| 12. | Coelho et al., 2020 [12] | Not a RAM for predicting the risk of developing VTE in hospital inpatients |
| 13. | Dang et al., 2019 [13] | Derivation only |
| 14. | Davis and Intagliata, 2018 [14] | No relevant/useable outcome data |
| 15. | Depietri et al., 2018 [15] | No relevant/useable outcome data |
| 16. | Ellis et al., 2019 [16] | No relevant/useable outcome data |
| 17. | Fadoi Foundation, 2020 [17] | Trial protocol (no results) |
| 18. | Ferreira et al., 2018 [18] | No relevant/useable outcome data |
| 19. | Ferreira et al., 2017 [19] | No relevant/useable outcome data |
| 20. | Fritz et al., 2021 [20] | No relevant/useable outcome data |
| 21. | Gibson et al., 2017 [21] | RAM involves diagnostic testing |
| 22. | Girardi et al., 2018 [22] | Not a RAM for predicting the risk of developing VTE in hospital inpatients |
| 23. | Grzelak et al., 2019 [23] | Not a RAM for predicting the risk of developing VTE in hospital inpatients |
| 24. | Hostler et al., 2016 [24] | No relevant/useable outcome data |
| 25. | Hu, 2018 [25] | No relevant/useable outcome data |
| 26. | Koren et al., 2017 [26] | Not a RAM for predicting the risk of developing VTE in hospital inpatients |
| 27. | Kucher et al., 2005 [27] | No relevant/useable outcome data |
| 28. | Lecumberri et al., 2008 [28] | No relevant/useable outcome data |
| 29. | Luo and Zhang, 2017 [29] | Not available (foreign language) |
| 30. | Maynard et al., 2010 [30] | No relevant/useable outcome data |
| 31. | McCaffrey et al., 2007 [31] | No relevant/useable outcome data |

| | | |
|---|---|---|
| 32. | Meizoso et al., 2017 [32] | Derivation only |
| 33. | Monti et al., 2019 [33] | Abstract of included full text paper |
| 34. | Mull et al., 2017 [34] | Review |
| 35. | Nafee et al., 2018 [35] | Abstract of included full text paper |
| 36. | Nnadi et al., 2017 [36] | No relevant/useable outcome data |
| 37. | Obi et al., 2015 [37] | Critical care patients |
| 38. | Pannucci et al., 2017 [38] | Review |
| 39. | Rafizadeh et al., 2016 [39] | No relevant/useable outcome data |
| 40. | Rastogi et al., 2020 [40] | No relevant/useable outcome data |
| 41. | Razak et al., 2019 [41] | Not a RAM for predicting the risk of developing VTE in hospital inpatients |
| 42. | Robert-Ebadi et al., 2017 [42] | Not a RAM for predicting the risk of developing VTE in hospital inpatients |
| 43. | Salim et al., 2018 [43] | No relevant/useable outcome data |
| 44. | Samama et al., 2006 [44] | Expert opinion |
| 45. | Shrotriya et al., 2018 [45] | Not a RAM for predicting the risk of developing VTE in hospital inpatients |
| 46. | Smilg Nicolas et al., 2018 [46] | No relevant/useable outcome data |
| 47. | Spirk et al., 2017 [47] | No relevant/useable outcome data |
| 48. | Spyropoulos et al., 2020 [48] | RAM involves diagnostic testing |
| 49. | Spyropoulos et al., 2011 [49] | Derivation only |
| 50. | Stuck et al., 2017 [50] | Review |
| 51. | Tadesse et al., 2020 [51] | No relevant/useable outcome data |
| 52. | Taha et al., 2020 [52] | No relevant/useable outcome data |
| 53. | Tung et al., 2020 [53] | No relevant/useable outcome data |
| 54. | Veith et al., 2019 [54] | No relevant/useable outcome data |
| 55. | Winoker et al., 2017 [55] | Abstract of an included full-text study |
| 56. | Yale et al., 2005 [56] | Derivation only |
| 57. | Ye et al., 2017 [57] | Review |
| 58. | Zakai et al., 2013 [58] | Derivation only |
| 59. | Zambelli et al., 2020 [59] | Not a RAM for predicting the risk of developing VTE in hospital inpatients |
| 60. | Zhou et al., 2012 [60] | Not a RAM for predicting the risk of developing VTE in hospital inpatients |

**REFERENCES (APPENDIX S2)**


1. Alikhan R, Cohen AT, Combe S, et al. Risk Factors for Venous Thromboembolism in Hospitalized Patients With Acute Medical Illness: Analysis of the MEDENOX Study. *JAMA Internal Medicine* 2004;164(9):963-68. doi: 10.1001/archinte.164.9.963
2. Alper EC, Ip IK, Balthazar P, et al. Risk Stratification Model: Lower-Extremity Ultrasonography for Hospitalized Patients with Suspected Deep Vein Thrombosis. *Journal of General Internal Medicine* 2018;33(1):21-25. doi: https://dx.doi.org/10.1007/s11606-017-4170-3
3. Arcelus JI, Candocia S, Traverso CI, et al. Venous thromboembolism prophylaxis and risk assessment in medical patients. *Semin Thromb Hemost* 1991;17:3313-8.
4. Arpaia GG, Caleffi A, Marano G, et al. Padua prediction score and IMPROVE score do predict in-hospital mortality in Internal Medicine patients. *Internal and Emergency Medicine* 2020;15(6):997-1003. doi: https://dx.doi.org/10.1007/s11739-019-02264-4
5. Bagot C, Gohil S, Perrott R, et al. The use of an exclusion-based risk-assessment model for venous thrombosis improves uptake of appropriate thromboprophylaxis in hospitalized medical patients. *QJM* 2010;103:597-605.
6. Bellizzi A, Palombi G, Bardi G, et al. Score Padua and IMPROVE: What changes in management of venous thromboembolism prophylaxis in Internal Medicine? *Italian Journal of Medicine* 2018;12(2 Supplement 1):2. doi: http://dx.doi.org/10.4081/itjm.2018.s2
7. Blondon M, Righini M, Nendaz M, et al. External validation of the simplified Geneva risk assessment model for hospital-associated venous thromboembolism in the Padua cohort. *Journal of Thrombosis and Haemostasis* 2020;18(3):676-80. doi: https://dx.doi.org/10.1111/jth.14688
8. Blondon M, Spirk D, Kucher N, et al. External validation and comparison of the improve risk assessment model with the Geneva risk assessment model in the estimate cohort. *Research and Practice in Thrombosis and Haemostasis* 2017;1(Supplement 1):180. doi: http://dx.doi.org/10.1002/rth2.12012
9. Caprini JA, Arcelus JI, Reyna J. Effective risk stratification of surgical and nonsurgical patients for venous thromboembolic disease. *Seminars in Hematology* 2001;38:12-19. doi: https://doi.org/10.1016/S0037-1963(01)90094-0
10. Chen XL, Pan L, Wang Y. [Validity of Padua risk assessment scale for assessing the risk of deep venous thrombosis in hospitalized patients]. *Zhonghua nei ke za zhi* 2018;57(7):514-17. doi: https://dx.doi.org/10.3760/cma.j.issn.0578-1426.2018.07.009
11. Chopard P, Spirk D, Bounameaux H, et al. Identifying acutely ill medical patients requiring thromboprophylaxis. *J Thromb Haemost* 2006;4(4):915-6.
12. Coelho J, Divernet-Queriaud M, Roy P-M, et al. Comparison of the Wells score and the revised Geneva score as a tool to predict pulmonary embolism in outpatients over age 65. *Thrombosis Research* 2020;196:120-26. doi: https://dx.doi.org/10.1016/j.thromres.2020.07.026

13. Dang JT, Switzer N, Delisle M, et al. Predicting venous thromboembolism following laparoscopic bariatric surgery: development of the BariClot tool using the MBSAQIP database. *Surgical Endoscopy* 2019;33(3):821-31. doi: https://dx.doi.org/10.1007/s00464-018-6348-0
14. Davis J, Intagliata NM. Practice patterns in application of venous thromboembolism prophylaxis to a hepatology inpatient service: A quality improvement project. *Hepatology* 2018;68(Supplement 1):296A. doi: http://dx.doi.org/10.1002/hep.30257
15. Depietri L, Marietta M, Scarlini S, et al. Clinical impact of application of risk assessment models (Padua Prediction Score and Improve Bleeding Score) on venous thromboembolism, major hemorrhage and health expenditure associated with pharmacologic VTE prophylaxis: a "real life" prospective and retrospective observational study on patients hospitalized in a Single Internal Medicine Unit (the STIME study). *Internal and Emergency Medicine* 2018;13(4):527-34. doi: https://dx.doi.org/10.1007/s11739-018-1808-z
16. Ellis N, Grubb C-M, Mustoe S, et al. Venous thromboembolism risk in psychiatric in-patients: a multicentre cross-sectional study. *BJPsych Bulletin* 2019:1-5. doi: https://dx.doi.org/10.1192/bjb.2019.25
17. Fadoi Foundation. Efficacy of the Use of Risk Scores in Reducing Important Clinical Outcomes in Hospitalized Medical Ill Patients, 2020. Available *https://clinicaltrialsgov/show/NCT04267718* [Accessed 2 March 2021]
18. Ferreira C, Avelino Mancini R, Monique Lana Diniz M, et al. Evaluation of inter-rater agreement of a risk assessment model for thromboprophylaxis in medical hospitalised patients: Preliminary results of a cohort study. *Research and Practice in Thrombosis and Haemostasis* 2018;2(Supplement 1):323-24. doi: http://dx.doi.org/10.1002/rth2.12125
19. Ferreira D, de Sousa JA, Felicissimo P, et al. Venous thromboembolism risk and prophylaxis in the Portuguese hospital care setting: The ARTE study. *Revista portuguesa de cardiologia : orgao oficial da Sociedade Portuguesa de Cardiologia (Portuguese Journal of Cardiology)* 2017;36(11):823-30. doi: https://dx.doi.org/10.1016/j.repc.2017.01.009
20. Fritz MK, Kincaid SE, Sargent CG, et al. Venous thromboembolism (VTE) risk stratification in general medical patients at an academic medical center. *Journal of Thrombosis and Thrombolysis* 2021;51(1):67-73. doi: https://dx.doi.org/10.1007/s11239-020-02144-1
21. Gibson CM, Spyropoulos AC, Cohen AT, et al. The IMPROVEDD VTE Risk Score: Incorporation of D-Dimer into the IMPROVE Score to Improve Venous Thromboembolism Risk Stratification. *TH Open* 2017;1(1):e56-e65. doi: https://dx.doi.org/10.1055/s-0037-1603929
22. Girardi AM, Bettiol RS, Garcia TS, et al. Wells and Geneva Scores Are Not Reliable Predictors of Pulmonary Embolism in Critically Ill Patients: A Retrospective Study. *Journal of Intensive Care Medicine* 2018:885066618816280. doi: https://dx.doi.org/10.1177/0885066618816280

23. Grzelak M, Javia V, Cox C, et al. Incidence and patient characteristics for the development of venous thromboembolic events within the adult burn center population and an evaluation of the caprini risk assessment model. *Journal of Burn Care and Research* 2019;40(Supplement 1):S57. doi: http://dx.doi.org/10.1093/jbcr/irz013.088
24. Hostler DC, Marx ES, Moores LK, et al. Validation of the international medical prevention registry on venous thromboembolism bleeding risk score. *Chest* 2016;149(2):372-79. doi: http://dx.doi.org/10.1378/chest.14-2842
25. Hu Y. Comparison between caprini and Khorana risk assessment models for hospitalized medical patients with cancer at high risk for venous thromboembolism: a retrospective study. *American Journal of Respiratory and Critical Care Medicine* 2018;197(MeetingAbstracts)
26. Koren O, Hourri A, Elias M, et al. Failure to validate padua score's ability to predict the risk of venous thromboembolism in medical patients. *Clinical Therapeutics* 2017;39(8 Supplement 1):e59.
27. Kucher N, Koo S, Quiroz R, et al. Electronic alerts to prevent venous thromboembolism among hospitalized patients. *N Engl J Med* 2005;352(10):969-77.
28. Lecumberri R, Marqués M, Díaz-Navarlaz MT, et al. Maintained effectiveness of an electronic alert system to prevent venous thromboembolism among hospitalized patients. *Thromb Haemost* 2008;100(4):699-704.
29. Luo X, Zhang F. Validation of the Caprini risk assessment model for venous thromboembolism in Chinese hospitalized patients in a general hospital. *National Medical Journal of China* 2017;97(24):1875-77. doi: http://dx.doi.org/10.3760/cma.j.issn.0376-2491.2017.24.007
30. Maynard GA, Morris TA, Jenkins IH, et al. Optimizing prevention of hospital-acquired venous thromboembolism (VTE): prospective validation of a VTE risk assessment model. *J Hosp Med* 2010;5(1):10-18.
31. McCaffrey R, Bishop M, Adonis-Rizzo M, et al. Development and Testing of a DVT Risk Assessment Tool: Providing Evidence of Validity and Reliability. *Worldviews on Evidence-Based Nursing* 2007;4(1):14-20. doi: 10.1111/j.1741-6787.2007.00074.x
32. Meizoso JP, Karcutskie CAt, Ray JJ, et al. A simplified stratification system for venous thromboembolism risk in severely injured trauma patients. *The Journal of Surgical Research* 2017;207:138-44. doi: https://dx.doi.org/10.1016/j.jss.2016.08.072
33. Monti M, Timpone S, Murdolo G, et al. A new risk assessment model for the stratification of the thromboembolism risk in medical patients: The TEVere Score. *Italian Journal of Medicine* 2019;13(Supplement 2):7-8. doi: http://dx.doi.org/10.4081/itjm.2019.s2
34. Mull N, Hecht T, Umscheid C, et al. Clinical risk prediction scores for venous thromboembolism in hospitalized patients. *International Journal of Technology Assessment in Health Care* 2017;33(Supplement 1):75-76. doi: http://dx.doi.org/10.1017/S0266462317002094

35. Nafee T, Gibson CM, Travis R, et al. Performance of a machine learning model vs. IMPROVE score for VTE prediction in acute medically ill patients: Insights from the APEX trial. *European Heart Journal* 2018;39(Supplement 1):431. doi: http://dx.doi.org/10.1093/eurheartj/ehy565.2160
36. Nnadi C, Soltani SJ, Brice HG, et al. Evaluation of a new thrombo-embolism scoring system (TESS) for spinal surgery. *Spine Journal* 2017;17(3):S21-S22. doi: http://dx.doi.org/10.1016/j.spinee.2016.12.068
37. Obi AT, Pannucci CJ, Nackashi A, et al. Validation of the Caprini Venous Thromboembolism Risk Assessment Model in Critically Ill Surgical Patients. *JAMA Surgery* 2015;150(10):941-48. doi: 10.1001/jamasurg.2015.1841
38. Pannucci CJ, Swistun L, MacDonald JK, et al. Individualized Venous Thromboembolism Risk Stratification Using the 2005 Caprini Score to Identify the Benefits and Harms of Chemoprophylaxis in Surgical Patients: A Meta-analysis. *Annals of Surgery* 2017;265(6):1094-103. doi: https://dx.doi.org/10.1097/SLA.0000000000002126
39. Rafizadeh R, Turgeon RD, Batterink J, et al. Characterization of venous thromboembolism risk in medical inpatients using different clinical risk assessment models. *Canadian Journal of Hospital Pharmacy* 2016;69(6):454-59.
40. Rastogi R, Mehaffey JH, Turrentine BE, et al. Implementation of an Electronic Medical Record Risk Stratification Tool to Reduce Venous Thromboembolism Events. *Journal of the American College of Surgeons* 2020;231(4 Supplement 1):S141-S42. doi: http://dx.doi.org/10.1016/j.jamcollsurg.2020.07.273
41. Razak MKA, Rasheed JI, Hamad AA. A comparison between wells' score and modified doppler ultrasound in the diagnosis of deep venous thrombosis of the lower limb. *International Journal of Pharmaceutical Sciences and Research* 2019;10(5):2369-74. doi: http://dx.doi.org/10.13040/IJPSR.0975-8232.10%285%29.2369-74
42. Robert-Ebadi H, Mostaguir K, Hovens MM, et al. Assessing clinical probability of pulmonary embolism: prospective validation of the simplified Geneva score. *Journal of Thrombosis and Haemostasis* 2017;15(9):1764-69. doi: https://dx.doi.org/10.1111/jth.13770
43. Salim S, Onida S, Davies A. Variability in venous thromboembolism (VTE) risk assessment tools. *Phlebology* 2018;33(1 Supplement 1):36-37. doi: http://dx.doi.org/10.1177/0268355518817748
44. Samama MM, Dahl OE, Mismetti P, et al. An electronic tool for venous thromboembolism prevention in medical and surgical patients. *Haematologica* 2006;91(1):64.
45. Shrotriya S, Dhakal P, Sharma M, et al. Predictors of venous thromboembolism among cancer inpatients: A predictive model. *Journal of Clinical Oncology* 2018;36(15 Supplement 1) doi: http://dx.doi.org/10.1200/JCO.2018.36.15_suppl.e18824

46. Smilg Nicolas C, Tornel Sanchez G, Trujillo Santos J. Concordance among venous thromboembolism risk assessment models. *Concordancia entre las escalas de prediccion de riesgo de tromboembolia venosa* 2018;150(2):61-63. doi: https://dx.doi.org/10.1016/j.medcli.2017.06.021
47. Spirk D, Stuck AK, Hager A, et al. Electronic alert system for improving appropriate thromboprophylaxis in hospitalized medical patients: a randomized controlled trial. *Journal of Thrombosis and Haemostasis* 2017;15(11):2138-46. doi: https://dx.doi.org/10.1111/jth.13812
48. Spyropoulos AC, Lipardi C, Xu J, et al. Modified IMPROVE VTE Risk Score and Elevated D-Dimer Identify a High Venous Thromboembolism Risk in Acutely Ill Medical Population for Extended Thromboprophylaxis. *TH Open* 2020;4(1):e59-e65. doi: https://dx.doi.org/10.1055/s-0040-1705137
49. Spyropoulos AC, Anderson FA, FitzGerald G, et al. Predictive and Associative Models to Identify Hospitalized Medical Patients at Risk for VTE. *Chest* 2011;140(3):706-14. doi: https://doi.org/10.1378/chest.10-1944
50. Stuck AK, Spirk D, Schaudt J, et al. Risk assessment models for venous thromboembolism in acutely ill medical patients. A systematic review. *Thrombosis and Haemostasis* 2017;117(4):801-08. doi: https://dx.doi.org/10.1160/TH16-08-0631
51. Tadesse TA, Kedir HM, Fentie AM, et al. Venous Thromboembolism Risk and Thromboprophylaxis Assessment in Surgical Patients Based on Caprini Risk Assessment Model. *Risk Management and Healthcare Policy* 2020;13:2545-52. doi: https://dx.doi.org/10.2147/RMHP.S272852
52. Taha H, Govindraj E, Jaber F, et al. Improving venous thromboembolism prophylaxis through critical thinking and health informatics. *BMJ Open Quality* 2020;9(2) doi: https://dx.doi.org/10.1136/bmjoq-2019-000885
53. Tung EC, Yu S-Y, Shah K, et al. Reassessment of venous thromboembolism and bleeding risk in medical patients receiving VTE prophylaxis. *Journal of Evaluation in Clinical Practice* 2020;26(1):18-25. doi: https://dx.doi.org/10.1111/jep.13213
54. Veith J, Collier W, Rockwell WB, et al. Direct Comparison of Patient-completed and Physician-completed Caprini Scores for Plastic Surgery Patients. *Plastic and Reconstructive Surgery Global Open* 2019;7(8):e2363. doi: https://dx.doi.org/10.1097/GOX.0000000000002363
55. Winoker JS, Paulucci DJ, Anastos H, et al. MP76-07 Predicting complications following robot-assisted partial nephrectomy with the ACS-NSQIP universal surgical risk calculator. *Journal of Urology* 2017;197(4S):e1015.
56. Yale S, Medlin S, Liang H, et al. Risk assessment model for venothromboembolism in post-hospitalized patients. *International Angiology* 2005;24(3):250.

57. Ye F, Stalvey C, Khuddus MA, et al. A systematic review of mobility/immobility in thromboembolism risk assessment models for hospitalized patients. *Journal of Thrombosis and Thrombolysis* 2017;44(1):94-103. doi: https://dx.doi.org/10.1007/s11239-017-1501-5
58. Zakai NA, Callas PW, Repp AB, et al. Venous thrombosis risk assessment in medical inpatients: the medical inpatients and thrombosis (MITH) study. *J Thromb Haemost* 2013;11(4):634-41.
59. Zambelli R, Nemeth B, Touw C, et al. Is the caprini score a reliable tool to predict the risk of venous thrombosis in orthopedic patients. *Research and Practice in Thrombosis and Haemostasis* 2020;4(SUPPL 1):1194-95. doi: http://dx.doi.org/10.1002/rth2.12393
60. Zhou H-X, Peng L-Q, Yan Y, et al. Validation of the Caprini risk assessment model in Chinese hospitalized patients with venous thromboembolism. *Thrombosis Research* 2012;130(5):735-40. doi: https://doi.org/10.1016/j.thromres.2012.08.001

BMJ Publishing Group Limited (BMJ) disclaims all liability and responsibility arising from any reliance placed on this supplemental material which has been supplied by the author(s)


## APPENDIX S3: WIDELY EVALUATED GENERIC RAMS, THEIR ASSOCIATED CHARACTERISTICS AND COMPOSITE CLINICAL VARIABLES

| Characteristics | Name of VTE risk assessment model | | | | | |
|---|---|---|---|---|---|---|
| | **Caprini Score** | **Padua Prediction Score** | **IMPROVE Predictive score** | **IMPROVE Associative score** | **Geneva Risk Score** | **Kucher Score** |
| **General** | | | | | | |
| Author, year | Caprini 2005[1] | Barbar 2010[2] | Tapson 2007[3] | Spyropoulos 2011[4] | Chopard 2006[5] | Kucher 2005[6] |
| Applicable cohort | Surgical and medical | Medical | Medical | Medical | Medical | Surgical and medical |
| Design | Ordinal with cumulative score | Dichotomous variables with cumulative score | Dichotomous variables with VTE probability estimate | Dichotomous variables with VTE probability estimate | Dichotomous variables with cumulative score | Dichotomous variables with cumulative score |
| Number of VTE risk variables | 31 | 11 | 4 | 7 | 19 | 8 |
| C-statistic (range) across medical, surgical and trauma cohorts | 0.53 - 0.87 (12 studies) | 0.594 - 0.756 (7 studies) | 0.57 – 0.65 (2 studies) | 0.63 – 0.7731 (4 studies) | 0.61 (1 study) | 0.563-0.756 (4 studies) |
| When is pharmacological thromboprophylaxis recommended? | Score ≥5 | Score ≥4 | No specific threshold Identified | No specific threshold identified | Score ≥3 | Score ≥4 |
| **Clinical Variables** | | | | | | |
| Patient related | | | | | | |
| Active cancer | Yes | Yes | Yes | Yes | Yes | Yes (Major risk) |
| Age | Yes | Yes (≥70) | Yes (≥60) | Yes (≥60) | Yes (≥60) | Yes (≥70 Minor risk) |
| Dehydration | No | No | No | No | Yes | No |
| Thrombophilia | Yes (generic and named conditions) | Yes (generic) | Yes (generic) | Yes (generic) | Yes (generic) | Yes (Major risk) |
| Obesity | Yes (≥25kg/m$^2$) | Yes (≥30kg/m$^2$) | No | No | Yes (≥30kg/m$^2$) | Yes (≥30kg/m$^2$ Minor risk) |
| Comorbidity | Yes (1 to 5 points for individual comorbidities) | Yes (1 point each for several individual comorbidities) | No | No | Yes (2 points each for several individual comorbidities) | No |
| Prior VTE | Yes | Yes | Yes | Yes | Yes | Yes (Major risk) |
| Family history of VTE | Yes | No | No | No | No | No |
| Use of HRT | Yes | Yes | No | No | Yes | Yes (Minor risk) |
| Use of oestrogen containing contraceptive therapy | Yes | Yes | No | No | Yes | Yes (Minor risk) |
| Varicose veins | Yes | No | No | No | No | No |
| Pregnancy or postpartum period | No | No | No | No | Yes | No |
| Unexplained stillbirth or spontaneous abortions | Yes (≥3 spontaneous abortions) | No | No | No | No | No |
| Current swollen legs | Yes | No | No | No | Yes | No |
| Current central venous access | Yes | No | No | No | No | No |
| Recent major surgery | Yes (<1 month) | Yes (<1 month) | No | No | No | No |
| Recent use of plaster cast immobilisation | Yes (<1 month) | No | No | No | No | No |

| | | | | | | |
|---|---|---|---|---|---|---|
| Lower limb paralysis | Yes | No | No | Yes | No | No |
| Travel related | No | No | No | No | Yes (>6hours) | No |
| Admission related | | | | | | |
| Reduced mobility | Yes (variable points) | Yes | No | Yes (≥7days) | Yes (≥3 days) | Yes (Minor risk) |
| Arthroplasty surgery | Yes | No | No | No | No | No |
| Hip fracture | Yes | No | No | No | No | No |
| Pelvic or lower limb surgery | Yes (arthroscopic) | No | No | No | No | No |
| Total anaesthetic and surgical time | Yes (≥45mins) | No | No | No | No | Yes (≥60mins intermediate risk) |
| Acute surgical admission | No | No | No | No | No | No |
| Acute infection | No | Yes | No | No | Yes | No |
| Acute rheumatologic disorder | No | Yes | No | No | Yes | No |
| Critical care admission | No | No | No | Yes | No | No |
| Surgery leading to reduced mobility | Yes | No | No | No | No | No |
| Other | | | | | | |
| 'Other risk factors' | Yes | No | No | No | No | No |

HRT, hormone replacement therapy; VTE, venous thromboembolism

## REFERENCES (APPENDIX S3)


1. Caprini JA, Arcelus JI, Hasty JH, et al. Clinical assessment of venous thromboembolic risk in surgical patients. *Semin Thromb Hemost* 1991;17:304-12.
2. Barbar S, Noventa F, Rossetto V, et al. A risk assessment model for the identification of hospitalized medical patients at risk for venous thromboembolism: the Padua Prediction Score. *Journal of Thrombosis and Haemostasis* 2010;8(11):2450-57. doi: 10.1111/j.1538-7836.2010.04044.x
3. Tapson VF, Decousus H, Pini M, et al. Venous thromboembolism prophylaxis in acutely ill hospitalized medical patients: findings from the International Medical Prevention Registry on Venous Thromboembolism. *Chest* 2007;132(3):936-45.
4. Spyropoulos AC, Anderson FA, FitzGerald G, et al. Predictive and Associative Models to Identify Hospitalized Medical Patients at Risk for VTE. *Chest* 2011;140(3):706-14. doi: https://doi.org/10.1378/chest.10-1944
5. Chopard P, Spirk D, Bounameaux H, et al. Identifying acutely ill medical patients requiring thromboprophylaxis. . *J Thromb Haemost* 2006;4(4):915-6.
6. Kucher N, Koo S, Quiroz R, et al. Electronic alerts to prevent venous thromboembolism among hospitalized patients. *N Engl J Med* 2005;352(10):969-77.

## APPENDIX S4: SUMMARY OF PREDICTIVE PERFORMANCE FOR STUDIES INVOLVING HOSPITAL INPATIENTS WHO REQUIRED CARE FOR CANCER, STROKE, BURN INJURIES AND SEPSIS OR WERE A MIXED MEDICAL/SURGICAL COHORT

| Data source | Endpoint | Incidence | Risk assessment model | Threshold or cut-off | Predictive performance | | |
|---|---|---|---|---|---|---|---|
| | | | | | C-statistic (95% CI) | Sensitivity (95% CI) | Specificity (95% CI) |
| **MIXED INPATIENTS** | | | | | | | |
| Autar, 2003[22] | DVT | 18.9% | •Novel (Autar, 2003) | Risk score ≤6 | NR | 100% (NR) | 100% (NR) |
| | | | •Novel (Autar, 2003) | Risk score 7-10 | NR | 86.0% (NR) | 68.0% (NR) |
| | | | •Novel (Autar, 2003) | Risk score 11-14 | NR | 68.0% (NR) | 31.0% (NR) |
| | | | •Novel (Autar, 2003) | Risk score ≥15 | NR | 25.0% (NR) | 10.0% (NR) |
| Elias, 2017[34] | VTE | 0.8% | •Padua (automated) | Risk score ≥5 | 0.81 (0.79, 0.83) | 85.4% (NR) | 53.3% (NR) |
| Chen, 2018[30] | DVT | NA | •Caprini | Risk score ≥4 | NR | 73.8% (NR) | 64.7% (NR) |
| | | | •Caprini | Risk score ≥5 | NR | 62.8% (NR) | 82.6% (NR) |
| | | | •Padua | Risk score ≥3 | NR | 53.5% (NR) | 82.1% (NR) |
| | | | •Padua | Risk score ≥4 | NR | 42.1% (NR) | 92.5% (NR) |
| Bo, 2020[29] | DVT | 0.9% | •Caprini | Risk score ≥3.5 | 0.74 (0.71, 0.77) | 75.0% (NR) | 62.0% (NR) |
| **CANCER INPATIENTS** | | | | | | | |
| Abdel-Razeq, 2010[21] | VTE | 3.5% | •Caprini (modified) | Risk score ≥3 | NR | 100% (NR) | 9.2% (NR) |
| | | | •Caprini (modified) | Risk score ≥5 | NR | 57.1% (NR) | 53.2% (NR) |
| Patell, 2017[54] | VTE | 3.8% | •Khorana | Risk score ≥3 | NR | 18.9% (NR) | 87.2% (NR) |
| Hu, 2020[42] | VTE | NA | •Caprini | Risk score ≥5 | 0.71 (0.66, 0.75) | 82.4% (NR) | 46.2% (NR) |
| | | | •Khorana | Risk score ≥2 | 0.58 (0.53, 0.63) | 35.3% (NR) | 78.7% (NR) |
| Shang, 2020[60] | VTE | NA | •Caprini 2009 | Risk score ≥3 | 0.72 (0.70, 0.74) | 83.5% (NR) | 52.7% (NR) |
| | | | •Caprini 2013 | Risk score ≥5 | 0.80 (0.78, 0.82) | 80.9% (NR) | 65.9% (NR) |
| **BURNS INPATIENTS** | | | | | | | |
| Pannucci, 2012[53] | VTE | 1.0% | •Novel (Pannucci 2012) | NR | 0.75 | NR | NR |
| **POST-STROKE INPATIENTS** | | | | | | | |
| Liu, 2014[44] | DVT | 10.5% | •Post-stroke DVT Prediction System | NR | 0.65 (0.59, 0.70) | NR | NR |
| **SEPSIS INPATIENTS** | | | | | | | |
| Vardi, 2013[64] | VTE | 1.3% | •Padua | NR | •All patients<br>0.58 (0.43, 0.73) | •All patients<br>NR | •All patients<br>NR |
| | | | •Padua | NR | •No prophylaxis<br>0.54 (0.37, 0.71) | •No prophylaxis<br>NR | •No prophylaxis<br>NR |

CI, confidence interval; DVT, deep vein thrombosis; NA, not applicable; NR, not reported; VTE, venous thromboembolism
