# Supplementary material for: Risk assessment models for venous thromboembolism in hospitalised adult patients: a systematic review
Source: BMJ Open. 2021 Jul 29;11(7):e045672. doi: 10.1136/bmjopen-2020-045672 (PMC8323381; doi:10.1136/bmjopen-2020-045672)
Supplement: Author's manuscript [file bmjopen-2020-045672.draft_revisions.pdf]

BMJ Open

BMJ Open is committed to open peer review. As part of this commitment we make the peer review history of every article we publish publicly available.

When an article is published we post the peer reviewers' comments and the authors' responses online. We also post the versions of the paper that were used during peer review. These are the versions that the peer review comments apply to.

The versions of the paper that follow are the versions that were submitted during the peer review process. They are not the versions of record or the final published versions. They should not be cited or distributed as the published version of this manuscript.

BMJ Open is an open access journal and the full, final, typeset and author-corrected version of record of the manuscript is available on our site with no access controls, subscription charges or pay-per-view fees (http://bmjopen.bmj.com).

If you have any questions on BMJ Open's open peer review process please email info.bmjopen@bmj.com

BMJ Open

Risk assessment models for venous thromboembolism in hospitalised adult patients – a systematic review

Journal:	*BMJ Open*
Manuscript ID	bmjopen-2020-045672
Article Type:	Original research
Date Submitted by the Author:	12-Oct-2020
Complete List of Authors:	Pandor, Abdullah; The University of Sheffield, ScHARR<br>Tonkins, Michael; The University of Sheffield, ScHARR<br>Goodacre, Steve; The University of Sheffield, ScHARR<br>Sworn, Katie; The University of Sheffield, ScHARR<br>Clowes, Mark; The University of Sheffield, ScHARR<br>Griffin, Xavier; Queen Mary University of London<br>Holland, Mark; Salford Royal NHS Foundation Trust<br>Hunt, Beverley; Guy's and St Thomas' NHS Foundation Trust, Dept of Haematology<br>de Wit, Kerstin; McMaster University Department of Medicine<br>Horner, Daniel; Salford Royal NHS Foundation Trust
Keywords:	VASCULAR MEDICINE, HAEMATOLOGY, Anticoagulation < HAEMATOLOGY, Quality in health care < HEALTH SERVICES ADMINISTRATION & MANAGEMENT

SCHOLARONE™
Manuscripts

I, the Submitting Author has the right to grant and does grant on behalf of all authors of the Work (as defined in the below author licence), an exclusive licence and/or a non-exclusive licence for contributions from authors who are: i) UK Crown employees; ii) where BMJ has agreed a CC-BY licence shall apply, and/or iii) in accordance with the terms applicable for US Federal Government officers or employees acting as part of their official duties; on a worldwide, perpetual, irrevocable, royalty-free basis to BMJ Publishing Group Ltd ("BMJ") its licensees and where the relevant Journal is co-owned by BMJ to the co-owners of the Journal, to publish the Work in this journal and any other BMJ products and to exploit all rights, as set out in our licence.

The Submitting Author accepts and understands that any supply made under these terms is made by BMJ to the Submitting Author unless you are acting as an employee on behalf of your employer or a postgraduate student of an affiliated institution which is paying any applicable article publishing charge ("APC") for Open Access articles. Where the Submitting Author wishes to make the Work available on an Open Access basis (and intends to pay the relevant APC), the terms of reuse of such Open Access shall be governed by a Creative Commons licence – details of these licences and which Creative Commons licence will apply to this Work are set out in our licence referred to above.

Other than as permitted in any relevant BMJ Author's Self Archiving Policies, I confirm this Work has not been accepted for publication elsewhere, is not being considered for publication elsewhere and does not duplicate material already published. I confirm all authors consent to publication of this Work and authorise the granting of this licence.

For peer review only

Complete manuscript title:

Risk assessment models for venous thromboembolism in hospitalised adult patients – a systematic review

Short title: Predicting VTE in hospitalised patients

Authors:

Abdullah Pandor, ScHARR, The University of Sheffield, Sheffield, UK

Michael Tonkins, ScHARR, The University of Sheffield, Sheffield, UK

Steve Goodacre, ScHARR, The University of Sheffield, Sheffield, UK

Katie Sworn, ScHARR, The University of Sheffield, Sheffield, UK

Mark Clowes, ScHARR, The University of Sheffield, Sheffield, UK

Xavier L Griffin, Queen Mary University of London, London, UK

Mark Holland, Salford Royal NHS Foundation Trust, Salford, UK

Beverley J Hunt, Guy's and St Thomas' NHS Foundation Trust, London, UK

Kerstin de Wit, Department of Medicine, McMaster University, Canada

Daniel Horner, Salford Royal NHS Foundation Trust, Salford, UK

Corresponding author: Abdullah Pandor, ScHARR, The University of Sheffield, Regent Court, 30 Regent Street, Sheffield, S1 4DA, telephone +44 (0) 114 222 0778, email: a.pandor@sheffield.ac.uk

Word count: 2871

For peer review only - http://bmjopen.bmj.com/site/about/guidelines.xhtml

ABSTRACT

INTRODUCTION

Hospital acquired thrombosis accounts for a large proportion of all venous thromboembolism (VTE), with significant morbidity and mortality. This subset of VTE can be reduced through accurate risk assessment and tailored pharmacological thromboprophylaxis. This systematic review aimed to determine the comparative accuracy of risk assessment models (RAMs) for predicting VTE in patients admitted to hospital.

METHODS

A systematic search was performed across five electronic databases (including MEDLINE, EMBASE and the Cochrane Library) to September 2019. All primary validation studies were eligible if they examined the accuracy of a multi-variable RAM (or scoring system) for predicting the risk of developing VTE in hospitalised inpatients. Two or more reviewers independently undertook study selection, data extraction, and risk of bias assessments using the PROBAST tool. We used narrative synthesis to summarise the findings.

RESULTS

We identified 44 studies, comprising 23 unique validated RAMs. The majority of studies included hospital inpatients who required medical care (n=18), were undergoing surgery (n=14) or receiving care for trauma (n=4). The most widely evaluated RAMs were the Caprini RAM (17 studies), Padua prediction score (13 studies), IMPROVE models (7 studies), the Geneva risk score (4 studies) and the Kucher score (4 studies). C-statistics varied markedly between studies and between models, with no one RAM performing obviously better than other models. C-statistics were often weak (<0.7), sometimes good (0.7-0.8) and rarely excellent (>0.8). Similarly, estimates for sensitivity and specificity were highly variable. Sensitivity estimates ranged from 12.0% to 100% and specificity estimates ranged from 7.2 to 100%.

For peer review only - http://bmjopen.bmj.com/site/about/guidelines.xhtml

CONCLUSION

Available data suggest that RAMs have generally weak predictive accuracy for VTE. There is insufficient evidence and too much heterogeneity to recommend the use of any particular RAM.

PROSPERO REGISTRATION

Steve Goodacre, Abdullah Pandor, Katie Sworn, Daniel Horner, Mark Clowes. A systematic review of venous thromboembolism RAMs for hospital inpatients. PROSPERO 2020 CRD42020165778 Available from https://www.crd.york.ac.uk/prospero/display_record.php?RecordID=165778

KEYWORDS

Hospitalization, inpatients, risk assessment, systematic review, venous thromboembolism

For peer review only

For peer review only - http://bmjopen.bmj.com/site/about/guidelines.xhtml

ARTICLE SUMMARY

Strengths and limitations of this study

- This systematic review provides an up-to-date comprehensive review of risk assessment models for predicting venous thromboembolism in patients admitted to hospital.
- The newly developed PROBAST tool was used to evaluate the risk of bias and applicability of the available evidence.
- Heterogeneity in the included studies (participants, inclusion criteria, clinical condition, outcome definition and measurement) and variable reporting of items precluded meta-analysis.
- Limitations of the existing evidence and areas of future research are highlighted.

For peer review only

For peer review only - http://bmjopen.bmj.com/site/about/guidelines.xhtml

INTRODUCTION

Venous thromboembolism (VTE) is an important and life threatening complication of hospitalisation and illness, and is associated with significant morbidity and mortality.[1 2] Globally, an estimated 10 million VTE episodes are diagnosed each year; over half of these episodes are associated with hospital inpatients stays and result in significant loss of disability adjusted life years.[3 4] Consequently, there has been a substantial and sustained focus on VTE prevention over the last three decades, with good evidence indicating a reduction in morbidity with primary thromboprophylaxis in hospitalised patients[5-8] Despite this evidence, thromboprophylaxis remains either underused or inappropriately applied.[9]

Risk assessment models (RAMs) have been developed to help stratify the risk of VTE amongst hospitalised patients.[10] These models use clinical information from the patient's history and examination to identify those with an increased risk of developing VTE who are most likely to benefit from pharmacological prophylaxis. Inappropriate use of VTE prophylaxis may not reduce VTE rates and may cause unnecessary harm.[11] Whilst RAMs could improve the ratio of benefit to risk and benefit to cost, it is unclear which VTE RAM should be applied to guide decision-making for prophylaxis in clinical practice and thereby optimise patient care.

The current review extends and updates three broadly overlapping existing reviews[10 12 13] and determines the accuracy of individual RAMs for predicting the risk of developing VTE in hospital inpatients.

METHODS

A systematic review was undertaken in accordance with the general principles recommended in the Preferred Reporting Items for Systematic Reviews and Meta-Analyses (PRISMA) statement.[14] This review was part of a larger project on VTE RAMs for hospital inpatients[15] and was registered on the International Prospective Register of Systematic Reviews (PROSPERO) database (CRD42020165778).

For peer review only - http://bmjopen.bmj.com/site/about/guidelines.xhtml

Eligibility criteria

We sought studies evaluating RAMs which could be applied to a general inpatient population (medical, surgical or trauma), rather than disease-specific models. All primary validation studies that evaluated the accuracy (e.g. sensitivity, specificity, C-statistic) of a multi-variable RAM (or scoring system) for predicting the risk of developing VTE were eligible for inclusion. We selected studies that included validation of the model in a group of patients that were not involved in model derivation. This involved either splitting the study cohort (internal) or using a new cohort (external). The study could have reported derivation of the model but we only used the validation data to estimate accuracy. The study population consisted of hospital inpatients including those who required medical care, undergoing any surgery (excluding day surgery) or received care following an injury. Studies that primarily focussed on children (aged under 16 years), women admitted to hospital for pregnancy-related reasons and any patient admitted to a level 2 or above critical care environment (e.g. patients requiring more detailed observation or intervention including support for a single failing organ system or post-operative care and those 'stepping down' from higher levels of care) were excluded. These patient groups have VTE risk profiles that differ markedly from the general inpatient population, making the use of a generic model inappropriate.

For peer review only

Data sources and searches

Potentially relevant studies were identified through searches of five electronic databases including MEDLINE (with MEDLINE In-process and Epub Ahead of Print), EMBASE and the Cochrane Library (2019, issue 9). The search strategy used free text and thesaurus terms and combined synonyms relating to the condition (e.g. VTE in medical inpatients) with risk prediction modelling terms. No language restrictions were used. However, as the current review updated three previous systematic reviews,[10 12 13] searches were limited by date from 2017 (last search date from earlier reviews)[9] to September 2019. Searches were supplemented by hand-searching the reference lists of all relevant studies (including existing systematic reviews); forward citation searching of included studies; contacting key experts in the field; and undertaking targeted searches of the World Wide Web

For peer review only - http://bmjopen.bmj.com/site/about/guidelines.xhtml

using the Google search engine. Further details on the search strategy can be found in **online supplementary Appendix S1.**

Study selection

All titles were examined for inclusion by one reviewer (KS) and any citations that clearly did not meet the inclusion criteria (e.g. non-human, unrelated to VTE inpatients) were excluded. All abstracts and full text articles were then examined independently by two reviewers (KS and AP). Any disagreements in the selection process were resolved through discussion or if necessary, arbitration by a third reviewer (SG) and included by consensus.

Data extraction and quality assessment

Data relating to study design, methodological quality and outcomes were extracted by one reviewer (KS) into a standardised data extraction form and independently checked for accuracy by a second (AP or MT). Any discrepancies were resolved through discussion to achieve agreement. Where differences were unresolved, a third reviewer's opinion was sought (SG). Where multiple publications of the same study were identified, data were extracted and reported as a single study.

The methodological quality of each included study was assessed using PROBAST (Prediction model Risk Of Bias ASsessment Tool).[16 17] This instrument evaluates four key domains: patient selection, predictors, outcome, and analysis. Each domain is assessed in terms of risk of bias and the concern regarding applicability to the review (first three domains only). To guide the overall domain-level judgement about whether a study is at high, low or an unclear (in the event of insufficient data in the publication to answer the corresponding question) risk of bias, sub-domains within each domain include a number of signalling questions to help judge with bias and applicability concerns. An overall risk of bias for each individual study was defined as low risk when all domains were judged as low; and high risk of bias when one or more domains were considered as high. Studies were assigned an unclear risk of bias if one or more domains were unclear and all other domains were low.

For peer review only - http://bmjopen.bmj.com/site/about/guidelines.xhtml

Data synthesis and analysis

We were unable to perform meta-analysis due to significant levels of heterogeneity between studies (participants, inclusion criteria, clinical condition) and variable reporting of items. As a result, a pre-specified narrative synthesis approach[18 19] was undertaken, with data being summarised in tables with accompanying narrative summaries that included a description of the included variables, statistical methods and performance measures (e.g. sensitivity, specificity and C-statistic [a value between 0.7 to 0.8 and >0.8 indicated good and excellent discrimination, respectively; values <0.7 were considered weak, and <0.5 as very weak[20]]), where applicable. All analyses were conducted using Microsoft Excel 2010 (Microsoft Corporation, Redmond, WA, USA).

Patient and public involvement

Patients and the public were not involved in the design or conduct of this systematic review.

RESULTS

Study flow

Figure 1 summarises the process of identifying and selecting relevant literature. Of the 3719 citations identified, 44 studies investigating 23 unique RAMs met the inclusion criteria. The majority of the articles were excluded primarily for not using a RAM for predicting the risk of developing VTE, having no useable or relevant outcome data or an inappropriate study design (e.g. derivation study, reviews, commentaries or editorials). A full list of excluded studies with reasons for exclusion is provided in **online supplementary Appendix S2.**

[INSERT FIGURE 1]

For peer review only - http://bmjopen.bmj.com/site/about/guidelines.xhtml

Study and patient characteristics

The design and participant characteristics of the 44 included studies[21-64] are summarised in **Table 1**. All studies were published between 2003 and 2019 and were undertaken in North America (n=23),[23 25 32-39 41 45 48-55 59 61 62] Asia (n=8),[29 42-44 56 57 63 64] Europe (n=8),[22 24 26-28 30 47 60] the Middle-East (n=2),[21 58] South America (n=1),[31] Australia (n=1)[40] and one study was intercontinental.[46] Sample sizes ranged from 70[39] to 1,099,093[41] patients in 35 observational cohort studies (10 prospective[21 22 24 28 31 42 47 48 52 58] [4 of which were multicentre] and 25 retrospective[23 25-27 32 33 35-41 44 46 49-51 54-57 59 61 62] [13 of which were multicentre] in design). Sample sizes in nine case control studies[29 30 34 43 45 53 60 63 64] (4 of which were multicentre) ranged from 149[34] to 19,217[53] patients.

For peer review only - http://bmjopen.bmj.com/site/about/guidelines.xhtml

Table 1: Study and population characteristics

Author, year	Country	Design	Single/ multi-centre	Sample size	Population	Mean age (years)	Male	VTE prophylaxis	RAMs	Target condition (risk period)	Incidence	Validation methodology
Autar, 2003[22]	UK	P,CS	Single	148	Hospitalised patients from orthopaedic, medical, and surgical specialties	NR	NR	50%	•Novel (Autar, 2003)	DVT, not defined (90 days)	18.9%	External
Rogers, 2007[52]	USA	P,CS	Multi	91,308	Hospitalised surgical patients (undergoing vascular and general surgery)	NR	NR	NR	•Novel (Rogers, 2007)	VTE (30 days)	0.6%	Internal: split (half)
Abdel-Razeq, 2010[21]	Jordan	P,CS	Single	606	Hospitalised (>24 hours) cancer patients aged ≥18 years	51	51%	55%	•Caprini (modified)	VTE, symptomatic (60 days)	3.5%	External
Bahl, 2010[23]	USA	R,CS	Multi	8,216	Hospitalised surgical patients (undergoing general, vascular, and urologic surgery)	NR	NR	NR	•Caprini	VTE, (30 days)	1.4%	External
Barbar, 2010[24]	Italy	P,CS	Single	1,180	Hospitalised medical patients	NR	47%	16%	•Padua	VTE, symptomatic (90 days)	3.1%	External
Rothberg, 2011[54]	USA	R,CS	Multi	48,540	Hospitalised (≥ 3 days) medical patients aged ≥18 years	NR	NR	30%	•Novel (Rothberg 2011)	VTE, hospital associated (NR)	0.5%	Internal: split (20%)
Woller, 2011[62]	USA	R,CS	Multi	46,856	Hospitalised medical patients aged ≥18 years	61	46%	NR	•Intermountain<br>•Kucher	VTE, defined by ICD-9 codes (90 days)	4.5%	Internal: split (25%)
Pannucci, 2012[49]	USA and Canada	R,CS	Multi	5,761	Hospitalised (>2 days) patients with a burn injury aged ≥18 years	46	69%	NR	•Novel (Panunucci 2012)	VTE, not defined (NR)	1.0%	Internal: split (25%)
Rogers, 2012[51]	USA	R,CS	Multi	234,032	Hospitalised trauma patients	NR	NR	NR	•TESS	VTE (NR)	NR	Internal: split
Bilimoria, 2013[25]	USA	R,CS	Multi	88,053	Hospitalised surgical patients (undergoing colorectal surgery)	NR	NR	NR	•ACS NSQIP - Colon specific<br>•ACS NSQIP - Universal	DVT, not defined (30 days)	2.3%	External: split by year)
Hegsted, 2013[38]	USA	R,CS	Single	2,281	Hospitalised (≥2 days) trauma patients aged ≥13 years	45	70%	NR	•RAP	DVT, not defined or PE (NR)	•DVT: 10.5%<br>•PE: 1.5%	External
Vardi, 2013[58]	Israel	P,CS	Single	1,080	Hospitalised (≥2 days) sepsis patients aged >18 years	75	52%	18%	•Padua	VTE, hospital associated (NR)	1.3%	External
Ho, 2014[40]	Australia	R,CS	Single	357	Hospitalised major trauma patients	NR	75%	NR	•TESS	VTE, symptomatic (NR)	20.7%	External
Liu, 2014[42]	China	P,CS	Single	287	Hospitalised acute stroke patients aged >18 years	NR	63%	22%	•Post-stroke DVT Prediction System	DVT (14±3 days)	10.5%	Internal: split (33%)
Mahan, 2014[45]	USA	CC	Multi	417	Hospitalised (≥3 days) medical patients aged ≥18 years	NR	49%	NR	•IMPROVE (7-factor)	VTE, hospital associated (92 days)	NA	External
Nendaz, 2014[47]	Switzerland	P,CS	Multi	1,478	Hospitalised (>24 hours) medical patients aged ≥18 years	65	53%	57%	•Geneva<br>•Padua	VTE, symptomatic including PE or DVT (90 days)	2.0%	External
Pannucci, 2014[48]	USA	P,CS	Multi	3,576	Hospitalised surgical patients aged ≥18 years	NR	NR	66%	•Novel (Panunucci 2014)	VTE (90 days)	1.4%	Internal: split (35%)

For peer review only - http://bmjopen.bmj.com/site/about/guidelines.xhtml

Rosenberg, 2014[53]	USA	CC	Multi	19,217	Hospitalised (≥3 days) medical patients aged ≥18 years	NR	47%	43%	•IMPROVE (7-factor)	VTE, defined by ICD-9 codes (90 days)	NA	External
Zhou 2014[64]	China	CC	Single	998	Hospitalised (≥2 days) medical patients aged >18 years	NR	58%	15%	•Caprini<br>•Padua	VTE, defined by ICD-10 codes (NR)	NA	External
Hewes, 2015[39]	USA	R,CS	Single	70	Hospitalised cancer patients (undergoing oesophagectomy)	NR	83%	96%	•Caprini (modified)	VTE (60 days)	14.3%	External
de Bastos, 2016[31]	Brazil	P,CS	Single	11,091	Hospitalised medical patients aged >18 years	50	61%	0%	•Caprini	VTE, symptomatic (NR)	0.3%	External
Grant, 2016[35]	USA	R,CS	Multi	63,548	Hospitalised (≥2 days) medical patients aged ≥18 years	66	45%	61%	•Caprini	VTE, hospital associated (90 days)	1.1%	External
Greene, 2016[36]	USA	R,CS	Multi	63,548	Acutely ill, hospitalised (≥2 days) medical patients aged ≥18 years	66	45%	61%	•IMPROVE (4-factor)<br>•Intermountain<br>•Kucher<br>•Padua	VTE, hospital associated (90 days)	1.1%	External
Hachey, 2016[37]	USA	R,CS	Single	232	Hospitalised surgical patients (undergoing segmenectomy, lobectomy or pneumonectomy for lung cancer)	NR	43%	92%	•Caprini	VTE (60 days)	5.2%	External
Lui, 2016[43]	China	CC	Single	640	Hospitalised (>2 days) medical patients aged ≥18 years	NR	52%	NR	•Caprini<br>•Padua	VTE (NR)	N/A	External
Lobastov, 2016[44]	Russia	R,CS [a]	Multi	140	Hospitalised high risk emergency surgery patients (undergoing general and neurosurgery)	69	49%	100%	•Caprini	DVT or PE, new (NR)	27.9%	External
Shaikh, 2016[55]	USA	R,CS	Multi	1,598	Hospitalised surgical patients (undergoing plastic surgery)	50	19%	34%	•Caprini	VTE, not defined (30 days)	1.5%	External
Elias, 2017[33]	USA	R,CS	Single	30,726	Hospitalised (>2 days) medical and surgical patients	NR	44%	21%	•Padua (automated)	VTE, defined by ICD-9 codes (NR)	0.8%	External
Frankel, 2017 (abstract)[34]	USA	CC	NR	149	Hospitalised surgical patients aged ≥18 years (undergoing robotic assisted laparoscopic prostatectomy)	NR	NR	NR	•Caprini	VTE, not defined (90 days)	NA	External
Krasnow, 2017 (abstract)[41]	USA	R,CS	Multi	1,099,093	Hospitalised surgical patients (major urological cancer surgery)	NR	NR	NR	•Caprini	VTE, symptomatic (90 days)	1.2%	External
Patell, 2017[50]	USA	R,CS	Single	2,780	Hospitalised (>24 hours) cancer patients aged >18 years	62 (median)	56%	65%	•Khorana	VTE, defined by ICD-9 codes (NR)	3.8%	External
Winoker, 2017[61]	USA	R,CS	Multi	300	Hospitalised surgical patients (undergoing urological surgery using robot-assisted partial nephrectomy)	61 (median)	62%	NR	•ACS NSQIP - Universal	VTE, not defined (NR)	0.3%	External
Blondon, 2018[28]	Switzerland	P,CS	Multi	1,478	Hospitalised (>24h) medical patients aged ≥18 years	65	53%	59%	•IMPROVE (7-factor)<br>•Geneva [b]<br>•Padua [b]	VTE, symptomatic including PE or DVT (90 days)	2.0%	External

For peer review only - http://bmjopen.bmj.com/site/about/guidelines.xhtml

Chen, 2018[29]	China	CC	Single	390	Hospitalised (>2days) patients aged ≥18 years with and without DVT	NR	51%	41%	•Caprini<br>•Padua	DVT (NR)	NA	External
Dornbus, 2018 (abstract)[32]	USA	R,CS	NR	2,830	Hospitalised surgical patients (undergoing neurosurgery)	NR	NR	NR	•Caprini	VTE, not defined (NR)	NR	External
Nafee, 2018 (abstract)[46]	35 countries	R,CS [a]	Multi	7,513	Hospitalised medical patients	NR	NR	100%	•IMPROVE (NR)<br>•Novel (Nafee, 2018)	VTE, not defined (77 days)	NR	External
Vaziri, 2018[59]	USA	R,CS	Single	1,006	Hospitalised surgical patients (undergoing neurosurgery)	NR	46%	NR	•ACS NSQIP- Universal	VTE, not defined (NR)	1.3%	External
Vincentelli, 2018[60]	Italy	CC	Multi	1,215	Acutely ill, hospitalised medical patients aged >18 years	NR	44%	NR	•Chopard<br>•Kucher<br>•Padua	VTE (NR)	NA	External
Zhou, 2018[63]	China	CC	Single	1,804	Hospitalised (≥2 days) medical patients aged >18 years	NR	59%	5%	•Caprini<br>•Padua	VTE, defined by ICD-10 codes (NR)	NA	External
Blondon, 2019a[26]	Italy	R,CS [a]	Single	1,180	Hospitalised medical patients	72	47%	20%	•Geneva (simplified)	VTE, symptomatic (90 days)	3.1%	External
Blondon, 2019b (abstract)[27]	Switzerland	R,CS [a]	Multi	991	Hospitalised elderly medical patients	75	55%	NR	•Geneva (simplified)<br>•IMPROVE (NR)<br>•Padua	VTE, symptomatic (NR)	15.0%	External
Cobben, 2019[30]	Netherlands	CC	Multi	556	Hospitalised (>24h) medical patients	NR	52%	NR	•Caprini<br>•Geneva<br>•IMPROVE (4-factor)<br>•IMPROVE (7-factor)<br>•Intermountain<br>•Kucher<br>•Lecumberri<br>•NAVAL<br>•NICE Guideline<br>•Padua<br>•PRETEMED Guideline<br>•Zakai et al (model 2)	VTE (NR)	NA	External
Tachino, 2019[56]	Japan	R,CS	Multi	859	Hospitalised (>24h) trauma patients aged ≥18 years	NR	64%	NR	•RAP<br>•Quick RAP	VTE (NR)	3.0%	External (RAP)/internal (qRAP)
Tian, 2019[57]	China	R,CS	Single	533	Hospitalised surgical patients (undergoing thoracic surgery)	53	53%	0%	•Caprini<br>•Khorana<br>•Padua<br>•Novel (Rogers, 2007)	VTE (NR)	8.4%	External

[a] Prospective cohort study with retrospective analysis, thus classified as retrospective cohort study
[b] Data overlap with Nendaz, 2014[47]
ACS NSQIP, American College of Surgeons National Surgical Quality Improvement Program; CC, case-control; CS, cohort study; DVT, deep vein thrombosis; NA, not applicable; NR, not reported; P, prospective; PE, pulmonary embolism; R, retrospective; RAMs, risk assessment models; RAP, Risk Assessment Profile; TESS, Trauma Embolic Scoring System VTE, venous thromboembolism

For peer review only

For peer review only - http://bmjopen.bmj.com/site/about/guidelines.xhtml

The vast majority of studies evaluated VTE risk assessment in hospital inpatients who required medical care (n=18),[24 26-28 30 31 35 36 43 45-47 53 54 60 62-64] were undergoing surgery (n=14)[23 25 32 34 37 39 41 44 48 52 55 57 59 61] or were a mixed medical and surgical cohort (n=3).[22 29 33] The remaining studies focused on patients receiving care for trauma (n=4),[38 40 51 56] cancer (n=2),[21 50] stroke (n=1),[42] burn injuries (n=1)[49] and sepsis (n=1).[58]. The mean age ranged from 45 years[38] to 75 years[27 58] (not reported in 29 studies)[22-25 29 30 32-34 37 39-43 45 46 48 50-54 56 59-61 63 64] and the proportion of female subjects ranged from 17%[39] to 81%[55] (not reported in 11 studies).[22 23 25 32 34 41 46 48 51 52 54]

VTE definition and case ascertainment

The majority of studies (n=31)[21 23 24 26-31 35-37 39-45 47 48 52-54 56-58 60 63 64] defined the VTE endpoint (DVT and or PE) as being objectively confirmed. Of the remainder, three studies[33 50 62] had no objective confirmation of VTE and 10 studies[22 25 32 34 38 46 49 55 59 61] did not report the methods for diagnosis confirmation. In terms of VTE risk period, half of the studies (n=22)[21-26 28 34-37 39 41 42 45-48 52 53 55 62] utilised the RAMs to predict the occurrence of VTE within 3 months of the index hospitalisation. The remaining studies did not report the VTE risk period. The reported incidence of VTE ranged widely from 0.3%[31 61] to 27.9%[44] depending on definition, study design and study participants (e.g. medical, surgical or trauma).

RAMs

The studies included in this review evaluated 23 validated unique RAMs. The most widely evaluated models were the Caprini RAM (17 studies),[21 23 29-32 34 35 37 39 41 43 44 55 57 63 64] Padua prediction score (13 studies),[24 27-30 33 36 43 47 57 58 60 63 64] IMPROVE models (7 studies),[27 28 30 36 45-47 53] the Geneva risk score (4 studies)[26-28 30 47] and the Kucher score (4 studies).[30 36 60 62]

Statistical methods

Statistical methods varied significantly between studies. Most studies reported the discrimination of the RAMs using a combination of the c-statistic and sensitivity or specificity. A minority reported calibration measures, such as the Hosmer-Lemeshow test.[23 39 40 46]

For peer review only - http://bmjopen.bmj.com/site/about/guidelines.xhtml
For peer review only

Risk of bias and applicability assessment

The overall methodological quality of the 44 included studies[21-64] is summarised in **Table 2 and Figure 2**. The methodological quality of the included studies was variable, with most studies having high or unclear risk of bias in at least one item of the PROBAST tool. The main sources of potential bias were related to the following domains:

1) Patient selection factors, such as retrospective data collection, incomplete patient enrolment, or unclear criteria for patients receiving VTE prophylaxis;

2) Predictor and outcome bias arising from inappropriate inclusion of predictors within RAMs, unclear methods of outcome definition, low event rates, and missing predictor or outcome data;

3) Analysis factors, such as small sample sizes, inappropriate handling of missing data and failure in reporting relevant performance measures such as calibration.

Assessment of applicability to the review question led to the majority of studies being classed either as high (n=28)[21 22 29 31 33 34 37-45 48-51 55-61 63 64] or unclear (n=12)[23 24 27 28 30 32 46 47 52-54 62] risk of inapplicability. These assessments were generally related to patient selection (highly selected study populations e.g. single pathologies, single site settings), predictors (inconsistency in definition, assessment or timing of predictors) and outcome determination.

For peer review only - http://bmjopen.bmj.com/site/about/guidelines.xhtml

Table 2. Summary of each study's risk of bias and applicability concern using the PROBAST tool – Review authors' judgements

Author, year	Risk of Bias				Concern regarding Applicability			OVERALL	OVERALL
	1. Participant selection	2. Predictors	3. Outcome	4. Analysis	1. Participant selection	2. Predictors	3. Outcomes	RISK OF BIAS	APPLICABILITY
Abdel-Razeq, 2010[21]	High	High	High	High	High	High	High	High	High
Autar, 2003[22]	High	High	High	High	High	High	High	High	High
Bahl, 2010[23]	High	High	High	High	Unclear	Unclear	Unclear	High	Unclear
Barbar, 2010[24]	Low	Unclear	Unclear	High	Low	Unclear	Unclear	High	Unclear
Bilimoria, 2013[25]	Low	Low	Low	High	Low	Low	Low	High	Low
Blondon, 2019a[26]	Low	Unclear	High	High	Low	Low	Low	High	Low
Blondon, 2019b (abstract)[27]	Unclear	Unclear	Unclear	Unclear	Unclear	Unclear	Unclear	Unclear	Unclear
Blondon, 2018[28]	Low	Unclear	Unclear	High	Unclear	Low	Unclear	High	Unclear
Chen, 2018[29]	High	High	High	High	Unclear	High	High	High	High
Cobben, 2019[30]	Unclear	Unclear	High	High	Unclear	Low	Unclear	High	Unclear
de Bastos, 2016[31]	High	Low	High	High	High	Low	Low	High	High
Dornbus, 2018 (abstract)[32]	High	Unclear	High	Unclear	Unclear	Unclear	Unclear	High	Unclear
Elias, 2017[33]	High	Unclear	High	High	Low	Low	High	High	High
Frankel, 2017 (abstract)[34]	High	Unclear	Unclear	High	High	Unclear	Unclear	High	High
Grant, 2016[35]	High	Unclear	Unclear	Unclear	Low	Low	Low	High	Low
Greene, 2016[36]	Unclear	Unclear	Unclear	Unclear	Low	Low	Low	Unclear	Low
Hachey, 2016[37]	High	Unclear	Unclear	High	High	Low	High	High	High
Hegsted, 2013[38]	High	Unclear	High	High	High	Low	Unclear	High	High
Hewes, 2015[39]	High	Unclear	Unclear	High	High	Unclear	Low	High	High
Ho, 2014[40]	Unclear	Unclear	Unclear	High	High	Unclear	Unclear	High	High
Krasnow, 2017 (abstract)[41]	Unclear	Unclear	Unclear	Unclear	High	Unclear	Unclear	Unclear	High
Liu, 2014[42]	Low	Low	Unclear	Unclear	High	High	High	Unclear	High
Lui, 2016[43]	High	Unclear	High	High	High	Low	Low	High	High
Lobastov, 2016[44]	Unclear	Unclear	Unclear	High	High	Low	High	High	High
Mahan, 2014[45]	Low	Unclear	Unclear	Unclear	High	Low	Unclear	Unclear	High
Nafee, 2018 (abstract)[46]	Unclear	Unclear	Unclear	Unclear	Unclear	Unclear	Unclear	Unclear	Unclear
Nendaz, 2014[47]	Low	Unclear	Low	High	Low	Unclear	Low	High	Unclear
Pannucci, 2012[49]	High	Unclear	Unclear	High	High	High	Unclear	High	High
Pannucci, 2014[48]	Low	Unclear	High	High	High	Low	Low	High	High
Patell, 2017[50]	High	Unclear	Unclear	High	High	Unclear	Unclear	High	High
Rogers, 2007[52]	Unclear	Unclear	Unclear	High	Low	Unclear	Unclear	High	Unclear
Rogers, 2012[51]	High	High	Unclear	High	High	High	Unclear	High	High
Rosenberg, 2014[53]	Low	Unclear	Unclear	Unclear	Unclear	Unclear	Unclear	Unclear	Unclear
Rothberg, 2011[54]	High	Unclear	Unclear	High	Low	Unclear	Unclear	High	Unclear
Shaikh, 2016[55]	High	Unclear	High	High	High	Unclear	High	High	High
Tachino, 2019[56]	High	Unclear	Unclear	High	High	Unclear	Unclear	High	High
Tian, 2019[57]	High	Unclear	High	High	High	High	High	High	High
Vardi, 2013[58]	Unclear	Low	Low	High	High	Low	Low	High	High
Vaziri, 2018[59]	Unclear	Unclear	Unclear	High	High	Unclear	Unclear	High	High
Vincentelli, 2018[60]	High	Low	Unclear	High	High	Low	Unclear	High	High

Winoker, 2017[61]	High	Unclear	Unclear	High	High	High	High	High	High
Woller, 2011[62]	High	High	Unclear	High	Unclear	Unclear	Unclear	High	Unclear
Zhou 2014[64]	Unclear	Unclear	Unclear	High	High	Unclear	Unclear	High	High
Zhou, 2018[63]	Low	High	High	High	High	Unclear	Unclear	High	High

For peer review only

For peer review only - http://bmjopen.bmj.com/site/about/guidelines.xhtml

[INSERT FIGURE 2]

Predictive performance of VTE RAMs (summary of results)

As there were a reasonable number of studies to compare, a summary of the c-statistics for studies involving medical, surgical and trauma patients respectively is presented in **Figures 3a-c**, with the results grouped by RAM. Results of other hospital inpatients are presented in **online supplementary Appendix S3**. C-statistics varied markedly between these studies and between models, with no RAM performing obviously better than other models. C-statistics[20] were often weak (<0.7), sometimes good (0.7-0.8) and rarely excellent (>0.8). There was marked heterogeneity between multiple studies evaluating the same model. Studies evaluating multiple models [30 36] tended to report weak accuracy across all the models (c-statistic<0.7).

[INSERT FIGURE 3]

Table 3 shows the sensitivity and specificity at various thresholds for studies involving medical, surgical and trauma patients respectively, with the results grouped by RAM. Interpretation was again limited by marked heterogeneity, which was exacerbated when different thresholds were reported by different studies evaluating the same model. Model accuracy was generally poor, with high sensitivity usually reflecting a threshold effect, as evidenced by corresponding low specificity (and vice versa).

For peer review only - http://bmjopen.bmj.com/site/about/guidelines.xhtml

Table 3. Sensitivity and specificity for studies involving medical, surgical and trauma inpatients

Risk assessment models	Threshold or cut-off	Endpoint	Data source	Sensitivity (95% CI)	Specificity (95% CI)
**MEDICAL INPATIENTS**					
**Caprini (6 studies)**	Risk Score ≥3	VTE	Lui, 2016[43]	70.9 (NR)	73.4 (NR)
	Risk Score ≥3	VTE	Zhou 2014[64]	82.3 (NR)	60.4 (NR)
	Risk Score ≥3	VTE	Zhou, 2018[63]	84.3 (NR)	66.2 (NR)
	Risk Score ≥5	VTE	Zhou, 2018[63]	57.1 (NR)	24.6 (NR)
	Risk score ≥5	VTE	Grant, 2016[35]	69.7 (NR)	50.28 (NR)
	Risk score ≥7	VTE	Grant, 2016[35]	42.69 (NR)	74.71 (NR)
	Risk score ≥9	VTE	Grant, 2016[35]	18.51 (NR)	89.03 (NR)
	NR[a]	VTE	de Bastos, 2016[31]	86.5 (NR)	47.0 (NR)
	NR	VTE	Cobben, 2019[30]	88.6 (NR)	21.4 (NR)
**Chopard (1 study)**	Risk Score ≥3	VTE	Vincentelli, 2018[60]	64.2 (38.4, 81.9)	57.7 (63.9, 79.4)
**Geneva models (4 studies)**	Risk Score ≥3	VTE	Blondon, 2018[28]; Nendaz, 2014[47]	All patients: 90.0 (73.5, 97.9)	All patients: 35.3 (32.8, 37.8)
				No prophylaxis: 85 (NR)	No prophylaxis: NR
	NR	VTE	Cobben, 2019[30]	75.0 (NR)	34.1 (NR)
	Simplified model: Risk Score ≥3	VTE	Blondon, 2019a[26]	95.0 (NR)	44.0 (NR)
	Simplified model: NR	VTE	Blondon, 2019b (abstract)[27]	86.4 (NR)	NR
**IMPROVE models (3 studies)**	4-factor model: NR	VTE	Cobben, 2019[30]	27.9 (NR)	85.4 (NR)
	7-factor model: Risk score 2-3	VTE	Blondon, 2018[28]; Nendaz, 2014[47]	All patients: 87 (NR)	All patients: NR
				No prophylaxis: 85 (NR)	No prophylaxis: NR
	7-factor model: Risk score ≥3	VTE	Blondon, 2018[28]; Nendaz, 2014[47]	All patients: 73 (NR)	All patients: NR
				No prophylaxis: 54 (NR)	No prophylaxis: NR
	7-factor model: NR	VTE	Cobben, 2019[30]	63.3 (NR)	70.7 (NR)
	NR	VTE	Blondon, 2019b (abstract)[27]	57.6 (NR)	NR
**Intermountain (1 study)**	NR	VTE	Cobben, 2019[30]	26.4 (NR)	90.2 (NR)
**Kucher (2 studies)**	Risk Score ≥4	VTE	Vincentelli, 2018[60]	25.1 (17.0, 55.1)	92.9 (81.0, 95.4)
	NR	VTE	Cobben, 2019[30]	28.0 (NR)	85.7 (NR)
**Lecumberri (1 study)**	NR	VTE	Cobben, 2019[30]	61.6 (NR)	46.3 (NR)
**NAVAL (1 study)**	NR	VTE	Cobben, 2019[30]	19.0 (NR)	92.7 (NR)
**NICE Guidelines (1 study)**	NR	VTE	Cobben, 2019[30]	77.6 (NR)	39.0 (NR)
**Padua (8 studies)**	Risk score ≥4	VTE	Barbar, 2010[24]	94.6 (NR)	62.0 (NR)
	Risk Score ≥4	VTE	Blondon, 2018[28]; Nendaz, 2014[47]	All patients: 73.3 (54.1, 87.7)	All patients: 51.9 (49.3, 54.5)
				No prophylaxis: 62 (NR)	No prophylaxis: NR
	Risk Score ≥4	VTE	Lui, 2016[43]	23.4 (NR)	85.6 (NR)
	Risk Score ≥4	VTE	Zhou 2014[64]	30.1 (NR)	12.7 (NR)
	Risk Score ≥4	VTE	Zhou, 2018[63]	49.1 (NR)	16.2 (NR)
	Risk Score ≥4	VTE	Vincentelli, 2018[60]	52.4 (38.4, 81.9)	72.3 (63.9, 79.4)
	NR	VTE	Blondon, 2019b (abstract)[27]	72.7 (NR)	NR
	NR	VTE	Cobben, 2019[30]	61.8 (NR)	48.8 (NR)
**PRETEMED Guidelines (1 study)**	NR	VTE	Cobben, 2019[30]	81.6 (NR)	24.4 (NR)
**Zakai 2013 (1 study)**	Model 2: NR	VTE	Cobben, 2019[30]	63.8 (NR)	31.7 (NR)
**SURGICAL INPATIENTS**					
**Caprini (7 studies)**	Risk Score >5	VTE	Hachey, 2016[37]	100 (100, 100)	7.2 (4.1, 11.0)
	Risk Score >5	VTE	Shaikh, 2016[55]	70.8 (48.9, 87.4)	39.39 (37.0, 41.9)
	Youden index >5.5	VTE	Tian, 2019[57]	76.0 (NR)	64.0 (NR)
	Risk score >6	VTE	Frankel, 2017 (abstract)[34]	61.5 (NR)	59.8 (NR)
	Risk Score >6	VTE	Shaikh, 2016[55]	58.3 (36.6, 77.9)	60.1 (57.6, 62.5)
	Risk Score >7	VTE	Hachey, 2016[37]	100 (100, 100)	31.4 (25, 37.3)
	Risk Score >9	VTE	Hachey, 2016[37]	83.3 (58.3, 100)	60.5 (54.4, 67.3)
	Risk Score >9	VTE	Shaikh, 2016[55]	16.7 (NR)	93.3 (NR)
	Risk Score >10	VTE	Hachey, 2016[37]	75.0 (50, 100)	69.6 (64.6, 76.4)
	Risk score >10	VTE	Dornbus, 2018 (abstract)[32]	78.9 (NR)	60.9 (NR)
	Risk Score >10.5	DVT or PE	Lobastov, 2016[44]	95.0 (NR)	73.0 (NR)
	Risk Score >15 [b]	VTE	Hewes, 2015[39]	100 (100, 100)	66.7 (55.0, 78.3)

For peer review only

**Khorana (1 study)**	Youden index >0.5	VTE	Tian, 2019[57]	78.0 (NR)	48.0 (NR)
**Padua (1 study)**	Youden index >3.5	VTE	Tian, 2019[57]	36.0 (NR)	93.0 (NR)
**Rogers 2007 (1 study)**	Youden index >14.5	VTE	Tian, 2019[57]	53.0 (NR)	54.0 (NR)
**TRAUMA PATIENTS**					
**RAP (2 studies)**	Risk score ≥5	VTE	Tachino, 2019[56]	100 (86.8, 100)	37.9 (34.6, 41.3)
	Risk score 5 to ≤ 14	DVT or PE	Hegsted, 2013[38]	•DVT: 82.0 (77, 87)<br>•PE: 71.0 (55, 86)	•DVT: 57.0 (55, 59)<br>•PE: 53.0 (51, 56)
	Risk score >14	DVT or PE	Hegsted, 2013[38]	•DVT: 15.0 (11, 20)<br>•PE: 12.0 (1, 23)	•DVT: 97.0 (97, 98)<br>•PE: 96.0 (95, 97)
**TESS (2 studies)**	Risk score ≥5	VTE	Rogers, 2012[51]	77.4 (NR)	75.6 (NR)
	Risk score <9	VTE	Ho, 2014[40]	•All VTE:<br>97.0 (91, 99)	•All VTE:<br>27 (22, 32)
	Risk score <9	VTE	Ho, 2014[40]	•Fatal and non-fatal PE: 97.0 (87, 99)	•Fatal and non-fatal PE: 24.0 (20, 29)
	Risk score <9	VTE	Ho, 2014[40]	•Fatal PE only:<br>100 (81, 100)	•Fatal PE only:<br>20.0 (13, 28)

[a] Paper states 'moderate and high risk'
[b] Modified Caprini model
CI, confidence interval; DVT, deep vein thrombosis; NR, not reported; PE, pulmonary embolism; RAP, Risk Assessment Profile; TESS, Trauma Embolic Scoring System VTE, venous thromboembolism

For peer review only

For peer review only - http://bmjopen.bmj.com/site/about/guidelines.xhtml

DISCUSSION

Summary of results

In this systematic review of 44 observational studies evaluating RAMs for predicting the risk of developing VTE in hospital inpatients, we found that VTE RAMs have generally weak predictive accuracy. The studies validating these models are heterogeneous and most have a high risk of bias. Lack of methodological clarity was common, leading to difficulty in assessing the applicability of the individual study results.

Interpretation of results

We were unable to undertake meta-analysis or statistical examination of the causes of the observed heterogeneity. Potential sources of heterogeneity include variation in study design, the study population, how RAMs are implemented, outcome definition and measurement, and the use of thromboprophylaxis. The latter point warrants further attention. Thromboprophylaxis was employed in just over half (n=23) of the studies,[21 22 24 26 28 29 33 35-37 39 42 44 46-48 50 53-55 58 63 64] with the proportion receiving thromboprophylaxis ranging from 5%[63] to 100%.[44 46] It was not employed in two studies[31 57] and 19 studies[23 25 27 30 32 34 38 40 41 43 45 49 51 52 56 59-62] did not report on thromboprophylaxis use. The use of thromboprophylaxis may lead to underestimation of predictive accuracy if a given RAM were to predict VTE events that were subsequently prevented by thromboprophylaxis. Limited reporting of thromboprophylaxis use precludes further analysis of its impact upon the performance of the RAMs.

Comparison to the existing literature

The present review is the largest and most comprehensive systematic review in this field to date. It includes 11 recent studies[26-30 32 46 56 57 60 63] published since the completion of the previous systematic review.[10 12 13] These studies are consistent with the previous literature in that they report modest performance of the assessed RAMs, with limitations in methodology and reporting preventing further analysis. The conclusion of this review therefore concurs with previous systematic reviews: there is insufficient evidence to recommend one RAM over another.

For peer review only - http://bmjopen.bmj.com/site/about/guidelines.xhtml

Strengths and limitations

This systematic review has a number of strengths. The review was conducted with robust methodology in accordance with the PRISMA statement and the protocol was registered with the PROSPERO register. Clinical experts were involved throughout as checkers and to assess the validity and applicability of research during the review. We reported descriptive statistics to provide insight into the limited evidence base applicable to the subject matter, and the scientific concerns regarding validity of the data. However, there are a number of potential weaknesses. Decisions on study relevance, information gathering, and validity were un-blinded and could potentially have been influenced by pre-formed opinions. However, masking is resource intensive with uncertain benefits. The studies of risk prediction were a combination of prospective cohorts and retrospective health database registries. Both have significant limitations. Retrospective studies of health database registries may have large numbers but may be limited by poor data quality and failure to accurately ascertain outcomes. Prospective cohorts may have better quality data but with smaller numbers lack statistical power. The included studies demonstrated high levels of heterogeneity so we were unable to undertake any meta-analysis

Implications for policy, practice and future research

Guidelines from the American College of Chest Physicians (ACCP)[65 66] and the UK National Institute for Health and Care Excellence (NICE)[10] suggest using a validated RAM to guide the decision on whether to prescribe thromboprophylaxis. This review identifies all relevant RAMs and their validation studies. The reported results are insufficient to recommend one RAM over another. A RAM with weak predictive accuracy may still be better than no RAM at all but it is unclear whether RAMs predict VTE risk better than unstructured clinical assessment. RAMs need to be implemented at a threshold that optimises sensitivity and specificity. Currently it is unclear what an appropriate risk threshold is for implementing thromboprophylaxis. In a study examining the consequences of VTE and the risks of thromboprophylaxis, Le *et al*.[67] suggest thromboprophylaxis is beneficial and cost-effective if a patient's VTE risk exceeds 1%. Further work to improve RAMs to help stratify the risk of VTE in different types of hospitalised patients could focus on undertaking an economic analysis to compare the effects, harms and costs of giving thromboprophylaxis to patients with varying risk of

For peer review only - http://bmjopen.bmj.com/site/about/guidelines.xhtml

VTE. This would allow determination of the risk threshold at which thromboprophylaxis provides optimal overall benefit. In addition, a study to evaluate use of RAMs to identify a low risk group where thromboprophylaxis can be withheld would be beneficial, since the ideal study (a prognostic cohort study in which no one receives thromboprophylaxis) is not ethical.

CONCLUSIONS

We identified a number of validated RAMs for potential risk stratification of hospitalised inpatients. The available evidence is insufficient to recommend one over another.

For peer review only

For peer review only - http://bmjopen.bmj.com/site/about/guidelines.xhtml

Acknowledgements

The authors would like to thank all additional members of the core project group for NIHR HTA 127454 for input and commentary throughout the work. We are also indebted to Helen Shulver for assistance with logistics and administration.

Competing interest

None to declare

Source of funding

This study was funded by the United Kingdom National Institute for Health Research Health Technology Assessment Programme (project number 127454). The views expressed in this report are those of the authors and not necessarily those of the NIHR HTA Programme. Any errors are the responsibility of the authors. The funders had no role in the study design, in the collection, analysis and interpretation of data; in the writing of the manuscript; and in the decision to submit the manuscript for publication.

Author contributions

AP co-ordinated the study. SG, DH, AP, XG, MH, BH, KW were responsible for conception, design and obtaining funding for the study. MC developed the search strategy, undertook searches, and organised retrieval of papers. AP, KS, MT and SG were responsible for the acquisition, analysis and interpretation of data. SG, MT, DH, XG, MH, BH, KW helped interpret and provided a methodological, policy and clinical perspective on the data. AP, MT and SG were responsible for the drafting of this paper, although all authors provided comments on the drafts, read and approved the final version. AP is the guarantor for the paper.

Copyright licence statement

The project was funded by the United Kingdom National Institute for Health Research Health Technology Assessment Programme and as such is covered by Crown copyright. The Corresponding

For peer review only

Author has the right to grant on behalf of all authors and does grant on behalf of all authors, **a non-exclusive license** on a worldwide basis to BMJ publishing (BMJ) and its Licensees to permit this article (if accepted) to be published by BMJ, as set out in our licence.

For peer review only

For peer review only - http://bmjopen.bmj.com/site/about/guidelines.xhtml

REFERENCES

1. Goldhaber SZ, Visani L, De Rosa M, et al. Acute ulmonary embolism: clinical outcomes in the International Cooperative Pulmonary Embolism Registry (ICOPER). *Lancet* 1999;353:1386–9.

2. Prandoni P, Lensing AW, Cogo A, et al. The longterm clinical course of acute deep venous thrombosis. *Ann Intern Med* 1996;125:1-7.

3. ISTH Steering Committee for World Thrombosis Day. Thrombosis: a major contributor to the global disease burden. *J Thromb Haemost* 2014;12

4. Jha AK, Larizgoitia I, Audera-Lopez C, et al. The global burden of unsafe medical care: analytic modelling of observational studies. *BMJ Qual Saf* 2013;22:809-15.

5. Alikhan R, Bedenis R, Cohen AT. Heparin for the prevention of venous thromboembolism in acutely ill medical patients (excluding stroke and myocardial infarction). *Cochrane Database Syst Rev* 2014;2014(5):Cd003747. doi: 10.1002/14651858.CD003747.

6. Dentali F, Douketis JD, Gianni M, et al. Meta-analysis: anticoagulant prophylaxis to prevent symptomatic venous thromboembolism in hospitalized medical patients. *Ann Intern Med* 2007;146(4)

7. Kahn SR, Diendéré G, Morrison DR, et al. Effectiveness of interventions for the implementation of thromboprophylaxis in hospitalised patients at risk of venous thromboembolism: an updated abridged Cochrane systematic review and meta-analysis of randomised controlled trials. *BMJ Open* 2019;9(5):e024444. doi: 10.1136/bmjopen-2018-024444 [published Online First: 2019/05/28]

8. Lloyd NS, Douketis JD, Moinuddin I, et al. Anticoagulant prophylaxis to prevent asymptomatic deep vein thrombosis in hospitalized medical patients: a systematic review and meta-analysis. *J Thromb Haemost* 2008;6(3):405-14. doi: 10.1111/j.1538-7836.2007.02847.x [published Online First: 2007/11/23]

9. Henke PK, Kahn SR, Pannucci CJ, et al. Call to Action to Prevent Venous Thromboembolism in Hospitalized Patients: A Policy Statement From the American Heart Association. *Circulation*

For peer review only - http://bmjopen.bmj.com/site/about/guidelines.xhtml

2020;141(24):e914-e31. doi: 10.1161/cir.0000000000000769 [published Online First: 2020/05/08]

10. NICE. Venous Thromboembolism in Over 16s: Reducing the Risk of Hospital-Acquired Deep Vein Thrombosis or Pulmonary Embolism. NICE guideline [NG89]: National Institute for Health and Care Excellence, London, UK. https://www.nice.org.uk/guidance/NG89, 2018.

11. Chan NC, Gross PL, Weitz JI. Addressing the burden of hospital-related venous thromboembolism: the role of extended anticoagulant prophylaxis. *J Thromb Haemost* 2018;16(3):413-17. doi: 10.1111/jth.13942 [published Online First: 2018/02/27]

12. Huang W, Anderson FA, Spencer FA, et al. Risk-assessment models for predicting venous thromboembolism among hospitalized non-surgical patients: a systematic review. *J Thromb Thrombolysis* 2013;35(1):67-80. doi: 10.1007/s11239-012-0780-0 [published Online First: 2012/07/25]

13. Stuck AK, Spirk D, Schaudt J, et al. Risk assessment models for venous thromboembolism in acutely ill medical patients. A systematic review. *Thrombosis and haemostasis* 2017;117(4):801-08. doi: https://dx.doi.org/10.1160/TH16-08-0631

14. Moher D, Liberati A, Tetzlaff J, et al. Preferred reporting items for systematic reviews and meta-analyses: the PRISMA statement. *Ann Intern Med* 2009;151(4):264-9, w64. doi: 10.7326/0003-4819-151-4-200908180-00135 [published Online First: 2009/07/23]

15. Goodacre S, Hogg K, Griffin X, et al. The cost-effectiveness of venous thromboembolism risk assessment tools for hospital inpatients. UK: National Institute of Health Research, 2019.

16. Moons KGM, Wolff RF, Riley RD, et al. PROBAST: A Tool to Assess Risk of Bias and Applicability of Prediction Model Studies: Explanation and Elaboration. *Ann Intern Med* 2019;170(1):W1-w33. doi: 10.7326/m18-1377 [published Online First: 2019/01/01]

17. Wolff RF, Moons KGM, Riley RD, et al. PROBAST: A Tool to Assess the Risk of Bias and Applicability of Prediction Model Studies. *Ann Intern Med* 2019;170(1):51-58. doi: 10.7326/m18-1376 [published Online First: 2019/01/01]

18. Centre for Reviews and Dissemination. Systematic Reviews: CRD's Guidance for Undertaking Reviews in Health Care: York: Centre for Reviews and Dissemination, 2009.

For peer review only - http://bmjopen.bmj.com/site/about/guidelines.xhtml

19. McKenzie JE, Brennan SE, Ryan RE, et al. Chapter 9: Summarizing study characteristics and preparing for synthesis. Cochrane Handbook for Systematic Reviews of Interventions version 60 (updated July 2019) [Internet]: Cochrane, 2019.

20. Hosmer DW, Lemeshow S. Applied Logistic Regression (2nd Edition): John Wiley & Sons. New York, NY. 2000.

21. Abdel-Razeq HN, Hijjawi SB, Jallad SG, et al. Venous thromboembolism risk stratification in medically-ill hospitalized cancer patients. A comprehensive cancer center experience. *Journal of Thrombosis and Thrombolysis* 2010;30(3):286-93. doi: 10.1007/s11239-010-0445-9

22. Autar R. The management of deep vein thrombosis: the autar DVT risk assessment scale re-visited. *Journal of Orthopaedic Nursing* 2003;7:114-24.

23. Bahl V, Hu HM, Henke PK, et al. A validation study of a retrospective venous thromboembolism risk scoring method. *Ann Surg* 2010;251(2):344-50. doi: 10.1097/SLA.0b013e3181b7fca6 [published Online First: 2009/09/26]

24. Barbar S, Noventa F, Rossetto V, et al. A risk assessment model for the identification of hospitalized medical patients at risk for venous thromboembolism: the Padua Prediction Score. *Journal of Thrombosis and Haemostasis* 2010;8(11):2450-57. doi: 10.1111/j.1538-7836.2010.04044.x

25. Bilimoria KY, Liu Y, Paruch JL, et al. Development and Evaluation of the Universal ACS NSQIP Surgical Risk Calculator: A Decision Aid and Informed Consent Tool for Patients and Surgeons. *Journal of the American College of Surgeons* 2013;217(5):833-42.e3. doi: https://doi.org/10.1016/j.jamcollsurg.2013.07.385

26. Blondon M, Righini M, Nendaz M, et al. External validation of the simplified Geneva risk assessment model for hospital-associated venous thromboembolism in the Padua cohort. *J Thromb Haemost* 2019:[Epub ahead of print]. doi: doi: 10.1111/jth.14688

27. Blondon M, Limacher A, Righini M, et al. Adequacy of hospital thromboprophylaxis and risk assessment models in the SWITCO65+ cohort. *Research and Practice in Thrombosis and Haemostasis* 2019;3(Supplement 1):760. doi: http://dx.doi.org/10.1002/rth2.12229

For peer review only

28. Blondon M, Spirk D, Kucher N, et al. Comparative Performance of Clinical Risk Assessment Models for Hospital-Acquired Venous Thromboembolism in Medical Patients. *Thrombosis and haemostasis* 2018;118(1):82-89. doi: https://dx.doi.org/10.1160/TH17-06-0403

29. Chen X, Pan L, Deng H, et al. Risk Assessment in Chinese Hospitalized Patients Comparing the Padua and Caprini Scoring Algorithms. *Clinical and applied thrombosis/hemostasis : official journal of the International Academy of Clinical and Applied Thrombosis/Hemostasis* 2018:1076029618797465. doi: https://dx.doi.org/10.1177/1076029618797465

30. Cobben MRR, Nemeth B, Lijfering WM, et al. Validation of risk assessment models for venous thrombosis in hospitalized medical patients. *Research and Practice in Thrombosis and Haemostasis* 2019;3(2):217-25. doi: http://dx.doi.org/10.1002/rth2.12181

31. de Bastos M, Barreto SM, Caiafa JS, et al. Derivation of a risk assessment model for hospital-acquired venous thrombosis: the NAVAL score. *Journal of Thrombosis and Thrombolysis* 2016;41(4):628-35. doi: http://dx.doi.org/10.1007/s11239-015-1277-4

32. Dornbos DL, Shah V, Priddy B, et al. Predicting venous thromboembolic complications following neurological surgery procedures. *Journal of Neurosurgery* 2018;128(4):55. doi: http://dx.doi.org/10.3171/2018.4.JNS.AANS2018abstracts

33. Elias P, Khanna R, Dudley A, et al. Automating Venous Thromboembolism Risk Calculation Using Electronic Health Record Data upon Hospital Admission: The Automated Padua Prediction Score. *Journal of Hospital Medicine* 2017;12(4):231-37. doi: https://dx.doi.org/10.12788/jhm.2714

34. Frankel JK, Belanger M, Tortora J, et al. Caprini score predicts venous thromboembolic events in patients undergoing robotic assisted prostatectomy. *Journal of Urology* 2017;197(4 Supplement 1):e635.

35. Grant PJ, Greene MT, Chopra V, et al. Assessing the Caprini Score for Risk Assessment of Venous Thromboembolism in Hospitalized Medical Patients Results presented at: The Society of Hospital Medicine Annual Meeting, March 26, 2014, Las Vegas, Nevada. *American Journal of Medicine* 2016;129(5):528-35. doi: http://dx.doi.org/10.1016/j.amjmed.2015.10.027

For peer review only - http://bmjopen.bmj.com/site/about/guidelines.xhtml

36. Greene MT, Spyropoulos AC, Chopra V, et al. Validation of Risk Assessment Models of Venous Thromboembolism in Hospitalized Medical Patients. *American Journal of Medicine* 2016;129(9):1001. doi: http://dx.doi.org/10.1016/j.amjmed.2016.03.031

37. Hachey KJ, Hewes PD, Porter LP, et al. Caprini venous thromboembolism risk assessment permits selection for postdischarge prophylactic anticoagulation in patients with resectable lung cancer. *The Journal of Thoracic and Cardiovascular Surgery* 2016;151(1):37-44.e1. doi: https://doi.org/10.1016/j.jtcvs.2015.08.039

38. Hegsted D, Gritsiouk Y, Schlesinger P, et al. Utility of the risk assessment profile for risk stratification of venous thrombotic events for trauma patients. *The American Journal of Surgery* 2013;205(5):517-20. doi: https://doi.org/10.1016/j.amjsurg.2013.01.022

39. Hewes PD, Hachey KJ, Zhang XW, et al. Evaluation of the Caprini Model for Venothromboembolism in Esophagectomy Patients. *The Annals of Thoracic Surgery* 2015;100(6):2072-78. doi: https://doi.org/10.1016/j.athoracsur.2015.05.098

40. Ho KM, Rao S, Rittenhouse KJ, et al. Use of the Trauma Embolic Scoring System (TESS) to predict symptomatic deep vein thrombosis and fatal and non-fatal pulmonary embolism in severely injured patients. *Anaesth Intensive Care* 2014;42(6):709-14. doi: 10.1177/0310057x1404200605 [published Online First: 2014/10/25]

41. Krasnow R, Preston M, Chung B, et al. Validation of venous thromboembolism risk assessment score in major urologic cancer surgery: A population based study. *Journal of Urology* 2017;197(4 Supplement 1):e1126.

42. Liu L-P, Zheng H-G, Wang DZ, et al. Risk Assessment of Deep-Vein Thrombosis After Acute Stroke: a Prospective Study Using Clinical Factors. *CNS Neuroscience & Therapeutics* 2014;20(5):403-10. doi: 10.1111/cns.12227

43. Liu X, Liu C, Chen X, et al. Comparison between Caprini and Padua risk assessment models for hospitalized medical patients at risk for venous thromboembolism: A retrospective study. *Interactive Cardiovascular and Thoracic Surgery* 2016;23(4):538-43. doi: http://dx.doi.org/10.1093/icvts/ivw158

For peer review only - http://bmjopen.bmj.com/site/about/guidelines.xhtml

44. Lobastov K, Barinov V, Schastlivtsev I, et al. Validation of the Caprini risk assessment model for venous thromboembolism in high-risk surgical patients in the background of standard prophylaxis. *Journal of Vascular Surgery: Venous and Lymphatic Disorders* 2016;4(2):153-60. doi: https://doi.org/10.1016/j.jvsv.2015.09.004

45. Mahan CE, Liu Y, Turpie AG, et al. External validation of a risk assessment model for venous thromboembolism in the hospitalised acutely-ill medical patient (VTE-VALOURR). *Thromb Haemost* 2014;112(4):692-9. doi: 10.1160/th14-03-0239 [published Online First: 2014/07/06]

46. Nafee T, Gibson CM, Travis R, et al. Performance of a machine learning model vs. IMPROVE score for VTE prediction in acute medically ill patients: Insights from the APEX trial. *European Heart Journal* 2018;39(Supplement 1):431. doi: http://dx.doi.org/10.1093/eurheartj/ehy565.2160

47. Nendaz M, Spirk D, Kucher N, et al. Multicentre validation of the Geneva Risk Score for hospitalised medical patients at risk of venous thromboembolism. Explicit ASsessment of Thromboembolic RIsk and Prophylaxis for Medical PATients in SwitzErland (ESTIMATE). *Thromb Haemost* 2014;111(3):531-8. doi: 10.1160/th13-05-0427 [published Online First: 2013/11/15]

48. Pannucci CJ, Laird S, Dimick JB, et al. A Validated Risk Model to Predict 90-Day VTE Events in Postsurgical Patients. *Chest* 2014;145(3):567-73. doi: https://doi.org/10.1378/chest.13-1553

49. Pannucci CJ, Osborne NH, Wahl WL. Creation and validation of a simple venous thromboembolism risk scoring tool for thermally injured patients: analysis of the National Burn Repository. *J Burn Care Res* 2012;33(1):20-25. doi: 10.1097/BCR.0b013e318234d8b5

50. Patell R, Rybicki L, McCrae KR, et al. Predicting risk of venous thromboembolism in hospitalized cancer patients: Utility of a risk assessment tool. *American Journal of Hematology* 2017;92(6):501-07. doi: https://dx.doi.org/10.1002/ajh.24700

51. Rogers FB, Shackford SR, Horst MA, et al. Determining venous thromboembolic risk assessment for patients with trauma: the Trauma Embolic Scoring System. *J Trauma Acute Care Surg* 2012;73(2):511-5. [published Online First: 2012/09/29]

For peer review only

For peer review only - http://bmjopen.bmj.com/site/about/guidelines.xhtml

52. Rogers SO Jr, Kilaru RK, Hosokawa P, et al. Multivariable predictors of postoperative venous thromboembolic events after general and vascular surgery: results from the patient safety in surgery study. *J Am Coll Surg* 2007;204(6):1211-21.

53. Rosenberg D, Eichorn A, Alarcon M, et al. External validation of the risk assessment model of the International Medical Prevention Registry on Venous Thromboembolism (IMPROVE) for medical patients in a tertiary health system. *Journal of the American Heart Association* 2014;3(6):e001152-e52. doi: 10.1161/JAHA.114.001152

54. Rothberg MB, Lindenauer PK, Lahti M, et al. Risk factor model to predict venous thromboembolism in hospitalized medical patients. *J Hosp Med* 2011;6(4):202-9. doi: 10.1002/jhm.888 [published Online First: 2011/04/12]

55. Shaikh M-A, Jeong HS, Mastro A, et al. Analysis of the American Society of Anesthesiologists Physical Status Classification System and Caprini Risk Assessment Model in Predicting Venous Thromboembolic Outcomes in Plastic Surgery Patients. *Aesthetic Surgery Journal* 2016;36(4):497-505. doi: 10.1093/asj/sjv198

56. Tachino J, Yamamoto K, Shimizu K, et al. Quick risk assessment profile (qRAP) is a prediction model for post-traumatic venous thromboembolism. *Injury* 2019;50(9):1540-44. doi: https://dx.doi.org/10.1016/j.injury.2019.06.020

57. Tian B, Li H, Cui S, et al. A novel risk assessment model for venous thromboembolism after major thoracic surgery: a Chinese single-center study. *Journal of Thoracic Disease* 2019;11(5):1903-10. doi: https://dx.doi.org/10.21037/jtd.2019.05.11

58. Vardi M, Ghanem-Zoubi NO, Zidan R, et al. Venous thromboembolism and the utility of the Padua Prediction Score in patients with sepsis admitted to internal medicine departments. *Journal of Thrombosis and Haemostasis* 2013;11(3):467-73. doi: 10.1111/jth.12108

59. Vaziri S, Wilson J, Abbatematteo J, et al. Predictive performance of the American College of Surgeons universal risk calculator in neurosurgical patients. *Journal of Neurosurgery* 2018;128(3):942-47. doi: https://dx.doi.org/10.3171/2016.11.JNS161377

For peer review only - http://bmjopen.bmj.com/site/about/guidelines.xhtml

60. Vincentelli GM, Timpone S, Murdolo G, et al. A new risk assessment model for the stratification of the thromboembolism risk in medical patients: the TEVere Score. *Minerva medica* 2018;109(6):436-42. doi: https://dx.doi.org/10.23736/S0026-4806.18.05689-6

61. Winoker JS, Paulucci DJ, Anastos H, et al. Predicting Complications Following Robot-Assisted Partial Nephrectomy with the ACS NSQIP Universal Surgical Risk Calculator. *The Journal of Urology* 2017;198(4):803-09. doi: https://dx.doi.org/10.1016/j.juro.2017.04.021

62. Woller SC, Stevens SM, Jones JP, et al. Derivation and Validation of a Simple Model to Identify Venous Thromboembolism Risk in Medical Patients. *The American Journal of Medicine* 2011;124(10):947-54.e2. doi: https://doi.org/10.1016/j.amjmed.2011.06.004

63. Zhou H, Hu Y, Li X, et al. Assessment of the Risk of Venous Thromboembolism in Medical Inpatients using the Padua Prediction Score and Caprini Risk Assessment Model. *Journal of Atherosclerosis and Thrombosis* 2018;25(11):1091-104. doi: https://dx.doi.org/10.5551/jat.43653

64. Zhou H, Wang L, Wu X, et al. Validation of a Venous Thromboembolism Risk Assessment Model in Hospitalized Chinese Patients: A Case-Control Study. *Journal of Atherosclerosis and Thrombosis* 2014;21(3):261-72. doi: 10.5551/jat.20891

65. Kahn SR, Lim W, Dunn AS, et al. Prevention of VTE in nonsurgical patients: Antithrombotic Therapy and Prevention of Thrombosis, 9th ed: American College of Chest Physicians Evidence-Based Clinical Practice Guidelines. *Chest* 2012;141:e195S-e226S.

66. Gould MK, Garcia DA, Wren SM, et al. Prevention of VTE in nonorthopedic surgical patients: Antithrombotic Therapy and Prevention of Thrombosis, 9th ed: American College of Chest Physicians Evidence-Based Clinical Practice Guidelines. *Chest* 2012;141:e227S-e77S.

67. Le P, Martinez KA, Pappas MA, et al. A decision model to estimate a risk threshold for venous thromboembolism prophylaxis in hospitalized medical patients. *J Thromb Haemost* 2017;15(6):1132-41.

For peer review only - http://bmjopen.bmj.com/site/about/guidelines.xhtml

Figure legends

**Figure 1**	**Study flow chart**
**Figure 2**	**PROBAST assessment summary graph - Review authors' judgements**
**Figure 3**	**C-statistics by model for studies involving a) medical b) surgical and c) trauma inpatients**

For peer review only

For peer review only - http://bmjopen.bmj.com/site/about/guidelines.xhtml

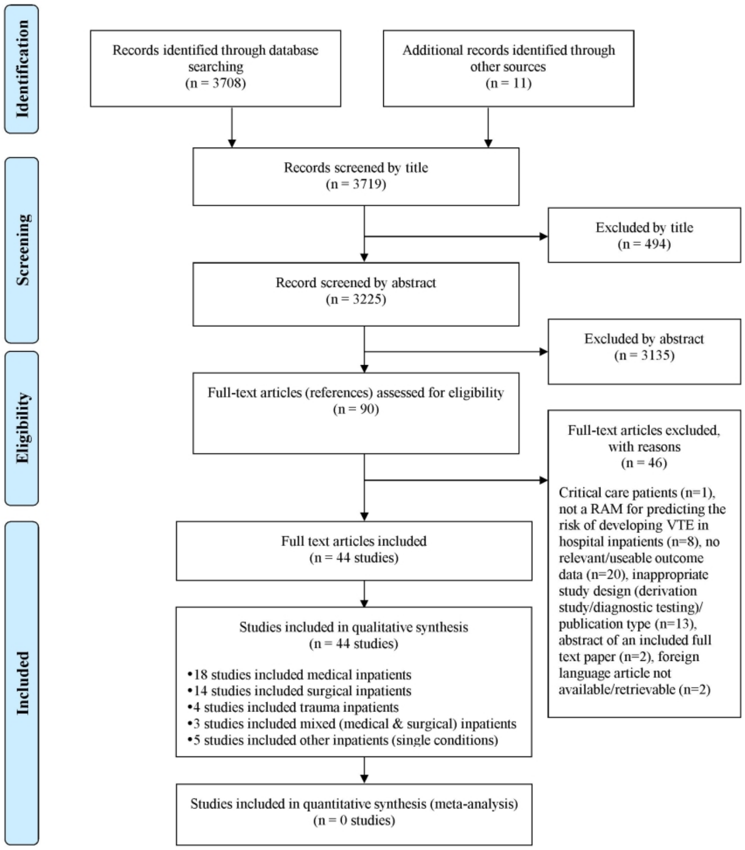

Figure 1

Study flow chart

209x297mm (300 x 300 DPI)

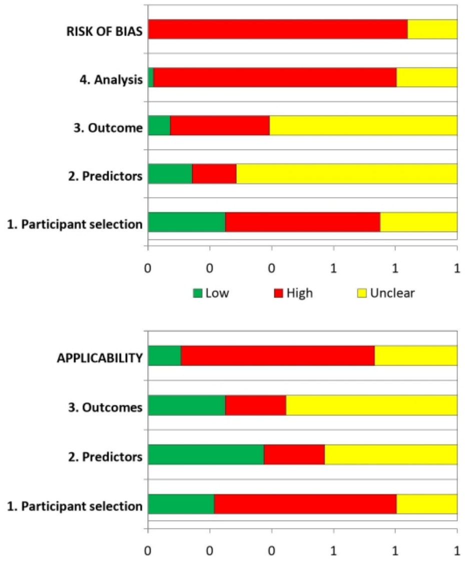

Figure 2

PROBAST assessment summary graph - Review authors' judgements

209x297mm (300 x 300 DPI)

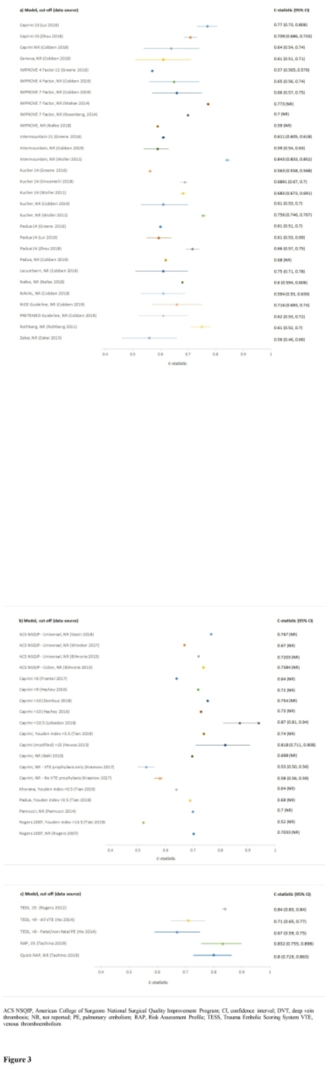

C-statistics by model for studies involving a) medical b) surgical and c) trauma inpatients

209x593mm (300 x 300 DPI)

ONLINE SUPPLEMENTARY

APPENDIX S1: LITERATURE SEARCH STRATEGY

Table S1 Literature search strategy for the review of RAMs for VTE in hospital inpatients

**Database searched:**	**Ovid MEDLINE(R) Epub Ahead of Print, In-Process & Other Non-Indexed Citations, Ovid MEDLINE(R) Daily, Ovid MEDLINE and Versions(R)**
**Platform or provider used:**	**Ovid SP**
**Date of coverage:**	**1946 to September 2019**
**Search undertaken:**	**September 2019**

1 pulmonary embolism/ or thromboembolism/ or venous thromboembolism/ or venous thrombosis/ or upper extremity deep vein thrombosis/
2 (((venous or vein) adj (thrombosis or thromboses or thrombus or thromboembolism)) or (dvt or vte) or ((pulmonary or lung) adj3 (embolism or emboli or embolus or emboliz* or thromboembolism))).ti,ab.
3 1 or 2
4 letter/ or editorial/ or news/ or exp historical article/ or anecdotes as topic/ or comment/ or case report/ or (letter or comment).ti.
5 randomized controlled trial/ or random*.ti,ab.
6 4 not 5
7 animals/ not humans/
8 exp animals, laboratory/
9 exp animal experimentation/
10 exp models, animal/
11 exp rodentia/
12 (rat or rats or mouse or mice).ti.
13 6 or 7 or 8 or 9 or 10 or 11 or 12
14 3 not 13
15 (risk* adj2 assess*).ti,ab.
16 ((score* or scoring) adj2 (tool* or system*)).ti,ab.
17 ((risk* or predict* or prognos*) adj4 (tool* or rule* or index* or indices or score* or scoring or scale* or model* or system* or algorithm* or stratif* or criteria or calculat*)).ti,ab.
18 (vienna adj5 cats).ti,ab.
19 (vienna cancer and thrombosis study).ti,ab.
20 trauma embolic scoring.ti,ab.
21 tess.ti,ab.
22 (roger* or caprini* or kucher* or cohen* or padua* or khorana* or autar).ti,ab.
23 (well* adj2 (score* or scoring)).ti,ab.
24 department of health.ti,ab,au.
25 or/15-24
26 14 and 25
27 limit 26 to yr="2017 -Current"

For peer review only

**Databases searched:**	**EMBASE**
**Platform or provider used:**	**Ovid SP**
**Date of coverage:**	**1974 to September 2019**
**Search undertaken:**	**September 2019**

1 thromboembolism/ or venous thromboembolism/ or vein thrombosis/ or deep veinthrombosis/ or leg thrombosis/ or lower extremity deep vein thrombosis/ or postoperativethrombosis/ or lung embolism/ or upper extremity deep vein thrombosis/
2 (((venous or vein) adj (thrombosis or thromboses or thrombus or thromboembolism)) or (dvt or vte) or ((pulmonary or lung) adj3 (embolism or emboli or embolus or emboliz* or thromboembolism))).ti,ab.
3 1 or 2
4 letter.pt. or letter/ or note.pt. or editorial.pt. or case report/ or case study/ or (letter or comment*).ti.
5 randomized controlled trial/ or random*.ti,ab.
6 4 not 5
7 animal/ not human/
8 nonhuman/
9 exp animal experiment/ or exp experimental animal/
10 animal model/
11 exp rodent/
12 (rat or rats or mouse or mice).ti.
13 6 or 7 or 8 or 9 or 10 or 11 or 12
14 3 not 13
15 (risk* adj2 assess*).ti,ab.
16 ((score* or scoring) adj2 (tool* or system*)).ti,ab.
17 ((risk* or predict* or prognos*) adj4 (tool* or rule* or index* or indices or score* or scoring or scale* or model* or system* or algorithm* or stratif* or criteria or calculat*)).ti,ab.
18 (vienna adj5 cats).ti,ab.
19 (vienna cancer and thrombosis study).ti,ab.
20 trauma embolic scoring.ti,ab.
21 tess.ti,ab.
22 (roger* or caprini* or kucher* or cohen* or padua* or khorana* or autar).ti,ab.
23 (well* adj2 (score* or scoring)).ti,ab.
24 department of health.ti,ab,au.
25 or/15-24
26 14 and 25
27 limit 26 to yr="2017 -Current"

For peer review only

**Databases searched:**	**Cochrane CENTRAL Register of Randomised Controlled Trials & Cochrane Database of Systematic Reviews**
**Platform or provider used:**	**www.thecochranelibrary.com**
**Date of coverage:**	**Inception to September 2019**
**Search undertaken:**	**September 2019**

#1. MeSH descriptor: [venous thromboembolism] this term only

#2. MeSH descriptor: [pulmonary embolism] this term only

#3. MeSH descriptor: [venous thrombosis] this term only

#4. MeSH descriptor: [thromboembolism] this term only

#5. MeSH descriptor: [upper extremity deep vein thrombosis] this term only

#6. ((*venous or *vein) next (thrombosis or thromboses or thrombus or thromboembolism) or dvt or vte or (pulmonary or lung) near/3 (embolism or emboli or embolus or emboliz* or thromboembolism)):ti,ab

#7. #1 or #2 or #3 or #4 or #6

#8. (risk* near/2 assess*):ti,ab

#9. ((score* or scoring) near/2 (tool* or system*)):ti,ab

#10. ((risk* or predict* or prognos*) near/4 (tool* or rule* or index* or indices or score* or scoring or scale* or model* or system* or algorithm* or stratif* or criteria or calculat*)):ti,ab

#11. (vienna near/5 cats):ti,ab

#12. (vienna cancer and thrombosis study):ti,ab

#13. trauma embolic scoring:ti,ab

#14. tess:ti,ab

#15. (roger* or caprini* or kucher* or cohen* or padua* or khorana* or autar):ti,ab

#16. (well* near/2 (score* or scoring)):ti,ab

#17. (department of health):ti,ab

#18. (or #8-#17)

#19. #7 and #18 with Publication Year from 2017 to 20

For peer review only

APPENDIX S2: List of excluded studies with rationale

	Authors, year	Reason for exclusion
1.	Alikhan et al., 2004 [1]	Derivation only
2.	Alper et al., 2018 [2]	Not a RAM for predicting the risk of developing VTE in hospital inpatients
3.	Arcelus et al., 1991 [3]	No relevant/useable outcome data
4.	Bagot et al., 2010 [4]	No relevant/useable outcome data
5.	Bellizzi et al., 2018 [5]	No relevant/useable outcome data
6.	Blondon et al., 2017 [6]	Abstract of an included full-text study
7.	Caprini et al., 2001 [7]	Review
8.	Chen et al., 2018 [8]	Not available/retrievable (foreign language)
9.	Chopard et al.,2006 [9]	No relevant/useable outcome data
10.	Dang et al., 2019 [10]	Derivation only
11.	Davis and Intagliata, 2018 [11]	No relevant/useable outcome data
12.	Depietri et al., 2018 [12]	No relevant/useable outcome data
13.	Ellis et al., 2019 [13]	No relevant/useable outcome data
14.	Ferreira et al., 2018 [14]	No relevant/useable outcome data
15.	Ferreira et al., 2017 [15]	No relevant/useable outcome data
16.	Gibson et al., 2017 [16]	RAM involves diagnostic testing
17.	Girardi et al., 2018 [17]	Not a RAM for predicting the risk of developing VTE in hospital inpatients
18.	Grzelak et al., 2019 [18]	Not a RAM for predicting the risk of developing VTE in hospital inpatients
19.	Hostler et al., 2016 [19]	No relevant/useable outcome data
20.	Hu, 2018 [20]	No relevant/useable outcome data
21.	Koren et al., 2017 [21]	Not a RAM for predicting the risk of developing VTE in hospital inpatients
22.	Kucher et al., 2005 [22]	No relevant/useable outcome data
23.	Lecumberri et al., 2008 [23]	No relevant/useable outcome data
24.	Luo and Zhang, 2017 [24]	Not available/retrievable (foreign language)
25.	Maynard et al., 2010 [25]	No relevant/useable outcome data
26.	McCaffrey et al., 2007 [26]	No relevant/useable outcome data
27.	Meizoso et al., 2017 [27]	Derivation only
28.	Mull et al., 2017 [28]	Review
29.	Nnadi et al., 2017 [29]	No relevant/useable outcome data
30.	Obi et al., 2015 [30]	Critical care patients
31.	Pannucci et al., 2017 [31]	Review
32.	Rafizadeh et al., 2016 [32]	No relevant/useable outcome data

For peer review only

33.	Razak et al., 2019 [33]	Not a RAM for predicting the risk of developing VTE in hospital inpatients
34.	Robert-Ebadi et al., 2017 [34]	Not a RAM for predicting the risk of developing VTE in hospital inpatients
35.	Salim et al., 2018 [35]	No relevant/useable outcome data
36.	Samama et al., 2006 [36]	Expert opinion
37.	Shrotriya et al., 2018 [37]	Not a RAM for predicting the risk of developing VTE in hospital inpatients
38.	Smilg Nicolas et al., 2018 [38]	No relevant/useable outcome data
39.	Spirk et al., 2017 [39]	No relevant/useable outcome data
40.	Spyropoulos et al., 2011 [40]	Derivation only
41.	Stuck et al., 2017 [41]	Review
42.	Winoker et al., 2017 [42]	Abstract of an included full-text study
43.	Yale et al., 2005 [43]	Derivation only
44.	Ye et al., 2017 [44]	Review
45.	Zakai et al., 2013 [45]	Derivation only
46.	Zhou et al., 2012 [46]	Not a RAM for predicting the risk of developing VTE in hospital inpatients

For peer review only

REFERENCES (Appendix S2)

1. Alikhan R, Cohen AT, Combe S, et al. Risk Factors for Venous Thromboembolism in Hospitalized Patients With Acute Medical Illness: Analysis of the MEDENOX Study. *JAMA Internal Medicine* 2004;164(9):963-68. doi: 10.1001/archinte.164.9.963
2. Alper EC, Ip IK, Balthazar P, et al. Risk Stratification Model: Lower-Extremity Ultrasonography for Hospitalized Patients with Suspected Deep Vein Thrombosis. *Journal of General Internal Medicine* 2018;33(1):21-25. doi: https://dx.doi.org/10.1007/s11606-017-4170-3
3. Arcelus JI, Candocia S, Traverso CI, et al. Venous thromboembolism prophylaxis and risk assessment in medical patients. *Semin Thromb Hemost* 1991;17:3313-8.
4. Bagot C, Gohil S, Perrott R, et al. The use of an exclusion-based risk-assessment model for venous thrombosis improves uptake of appropriate thromboprophylaxis in hospitalized medical patients. *QJM* 2010;103:597-605.
5. Bellizzi A, Palombi G, Bardi G, et al. Score Padua and IMPROVE: What changes in management of venous thromboembolism prophylaxis in Internal Medicine? *Italian Journal of Medicine* 2018;12(2 Supplement 1):2. doi: http://dx.doi.org/10.4081/itjm.2018.s2
6. Blondon M, Spirk D, Kucher N, et al. External validation and comparison of the improve risk assessment model with the Geneva risk assessment model in the estimate cohort. *Research and Practice in Thrombosis and Haemostasis* 2017;1(Supplement 1):180. doi: http://dx.doi.org/10.1002/rth2.12012
7. Caprini JA, Arcelus JI, Reyna J. Effective risk stratification of surgical and nonsurgical patients for venous thromboembolic disease. *Seminars in Hematology* 2001;38:12-19. doi: https://doi.org/10.1016/S0037-1963(01)90094-0
8. Chen XL, Pan L, Wang Y. [Validity of Padua risk assessment scale for assessing the risk of deep venous thrombosis in hospitalized patients]. *Zhonghua nei ke za zhi* 2018;57(7):514-17. doi: https://dx.doi.org/10.3760/cma.j.issn.0578-1426.2018.07.009
9. Chopard P, Spirk D, Bounameaux H, et al. Identifying acutely ill medical patients requiring thromboprophylaxis. *J Thromb Haemost* 2006;4(4):915-6.
10. Dang JT, Switzer N, Delisle M, et al. Predicting venous thromboembolism following laparoscopic bariatric surgery: development of the BariClot tool using the MBSAQIP database. *Surgical Endoscopy* 2019;33(3):821-31. doi: https://dx.doi.org/10.1007/s00464-018-6348-0
11. Davis J, Intagliata NM. Practice patterns in application of venous thromboembolism prophylaxis to a hepatology inpatient service: A quality improvement project. *Hepatology* 2018;68(Supplement 1):296A. doi: http://dx.doi.org/10.1002/hep.30257
12. Depietri L, Marietta M, Scarlini S, et al. Clinical impact of application of risk assessment models (Padua Prediction Score and Improve Bleeding Score) on venous thromboembolism, major hemorrhage and health expenditure associated with pharmacologic VTE prophylaxis: a "real life" prospective and retrospective observational study on patients hospitalized in a Single

For peer review only

Internal Medicine Unit (the STIME study). *Internal and Emergency Medicine* 2018;13(4):527-34. doi: https://dx.doi.org/10.1007/s11739-018-1808-z

13. Ellis N, Grubb C-M, Mustoe S, et al. Venous thromboembolism risk in psychiatric in-patients: a multicentre cross-sectional study. *BJPsych bulletin* 2019:1-5. doi: https://dx.doi.org/10.1192/bjb.2019.25
14. Ferreira C, Avelino Mancini R, Monique Lana Diniz M, et al. Evaluation of inter-rater agreement of a risk assessment model for thromboprophylaxis in medical hospitalised patients: Preliminary results of a cohort study. *Research and Practice in Thrombosis and Haemostasis* 2018;2(Supplement 1):323-24. doi: http://dx.doi.org/10.1002/rth2.12125
15. Ferreira D, de Sousa JA, Felicissimo P, et al. Venous thromboembolism risk and prophylaxis in the Portuguese hospital care setting: The ARTE study. *Revista portuguesa de cardiologia : orgao oficial da Sociedade Portuguesa de Cardiologia (Portuguese Journal of Cardiology: an Official Journal of the Portuguese Society of Cardiology)* 2017;36(11):823-30. doi: https://dx.doi.org/10.1016/j.repc.2017.01.009
16. Gibson CM, Spyropoulos AC, Cohen AT, et al. The IMPROVEDD VTE Risk Score: Incorporation of D-Dimer into the IMPROVE Score to Improve Venous Thromboembolism Risk Stratification. *TH Open* 2017;1(1):e56-e65. doi: https://dx.doi.org/10.1055/s-0037-1603929
17. Girardi AM, Bettiol RS, Garcia TS, et al. Wells and Geneva Scores Are Not Reliable Predictors of Pulmonary Embolism in Critically Ill Patients: A Retrospective Study. *Journal of Intensive Care Medicine* 2018:885066618816280. doi: https://dx.doi.org/10.1177/0885066618816280
18. Grzelak M, Javia V, Cox C, et al. Incidence and patient characteristics for the development of venous thromboembolic events within the adult burn center population and an evaluation of the caprini risk assessment model. *Journal of Burn Care and Research* 2019;40(Supplement 1):S57. doi: http://dx.doi.org/10.1093/jbcr/irz013.088
19. Hostler DC, Marx ES, Moores LK, et al. Validation of the international medical prevention registry on venous thromboembolism bleeding risk score. *Chest* 2016;149(2):372-79. doi: http://dx.doi.org/10.1378/chest.14-2842
20. Hu Y. Comparison between caprini and Khorana risk assessment models for hospitalized medical patients with cancer at high risk for venous thromboembolism: a retrospective study. *American Journal of Respiratory and Critical Care Medicine* 2018;197(MeetingAbstracts)
21. Koren O, Hourri A, Elias M, et al. Failure to validate padua score's ability to predict the risk of venous thromboembolism in medical patients. *Clinical Therapeutics* 2017;39(8 Supplement 1):e59.
22. Kucher N, Koo S, Quiroz R, et al. Electronic alerts to prevent venous thromboembolism among hospitalized patients. *N Engl J Med* 2005;352(10):969-77.

For peer review only

23. Lecumberri R, Marqués M, Díaz-Navarlaz MT, et al. Maintained effectiveness of an electronic alert system to prevent venous thromboembolism among hospitalized patients. *Thromb Haemost* 2008;100(4):699-704.

24. Luo X, Zhang F. Validation of the Caprini risk assessment model for venous thromboembolism in Chinese hospitalized patients in a general hospital. *National Medical Journal of China* 2017;97(24):1875-77. doi: http://dx.doi.org/10.3760/cma.j.issn.0376-2491.2017.24.007

25. Maynard GA, Morris TA, Jenkins IH, et al. Optimizing prevention of hospital-acquired venous thromboembolism (VTE): prospective validation of a VTE risk assessment model. *J Hosp Med* 2010;5(1):10-18.

26. McCaffrey R, Bishop M, Adonis-Rizzo M, et al. Development and Testing of a DVT Risk Assessment Tool: Providing Evidence of Validity and Reliability. *Worldviews on Evidence-Based Nursing* 2007;4(1):14-20. doi: 10.1111/j.1741-6787.2007.00074.x

27. Meizoso JP, Karcutskie CAt, Ray JJ, et al. A simplified stratification system for venous thromboembolism risk in severely injured trauma patients. *The Journal of Surgical Research* 2017;207:138-44. doi: https://dx.doi.org/10.1016/j.jss.2016.08.072

28. Mull N, Hecht T, Umscheid C, et al. Clinical risk prediction scores for venous thromboembolism in hospitalized patients. *International Journal of Technology Assessment in Health Care* 2017;33(Supplement 1):75-76. doi: http://dx.doi.org/10.1017/S0266462317002094

29. Nnadi C, Soltani SJ, Brice HG, et al. Evaluation of a new thrombo-embolism scoring system (TESS) for spinal surgery. *Spine Journal* 2017;17(3):S21-S22. doi: http://dx.doi.org/10.1016/j.spinee.2016.12.068

30. Obi AT, Pannucci CJ, Nackashi A, et al. Validation of the Caprini Venous Thromboembolism Risk Assessment Model in Critically Ill Surgical Patients. *JAMA Surgery* 2015;150(10):941-48. doi: 10.1001/jamasurg.2015.1841

31. Pannucci CJ, Swistun L, MacDonald JK, et al. Individualized Venous Thromboembolism Risk Stratification Using the 2005 Caprini Score to Identify the Benefits and Harms of Chemoprophylaxis in Surgical Patients: A Meta-analysis. *Annals of Surgery* 2017;265(6):1094-103. doi: https://dx.doi.org/10.1097/SLA.0000000000002126

32. Rafizadeh R, Turgeon RD, Batterink J, et al. Characterization of venous thromboembolism risk in medical inpatients using different clinical risk assessment models. *Canadian Journal of Hospital Pharmacy* 2016;69(6):454-59.

33. Razak MKA, Rasheed JI, Hamad AA. A comparison between wells' score and modified doppler ultrasound in the diagnosis of deep venous thrombosis of the lower limb. *International Journal of Pharmaceutical Sciences and Research* 2019;10(5):2369-74. doi: http://dx.doi.org/10.13040/IJPSR.0975-8232.10%285%29.2369-74

For peer review only

34. Robert-Ebadi H, Mostaguir K, Hovens MM, et al. Assessing clinical probability of pulmonary embolism: prospective validation of the simplified Geneva score. *J Thromb Haemost.* 2017;15(9):1764-69. doi: https://dx.doi.org/10.1111/jth.13770

35. Salim S, Onida S, Davies A. Variability in venous thromboembolism (VTE) risk assessment tools. *Phlebology* 2018;33(1 Supplement 1):36-37. doi: http://dx.doi.org/10.1177/0268355518817748

36. Samama MM, Dahl OE, Mismetti P, et al. An electronic tool for venous thromboembolism prevention in medical and surgical patients. *Haematologica* 2006;91(1):64.

37. Shrotriya S, Dhakal P, Sharma M, et al. Predictors of venous thromboembolism among cancer inpatients: A predictive model. *Journal of Clinical Oncology* 2018;36(15 Supplement 1) doi: http://dx.doi.org/10.1200/JCO.2018.36.15_suppl.e18824

38. Smilg Nicolas C, Tornel Sanchez G, Trujillo Santos J. Concordance among venous thromboembolism risk assessment models. *Concordancia entre las escalas de prediccion de riesgo de tromboembolia venosa* 2018;150(2):61-63. doi: https://dx.doi.org/10.1016/j.medcli.2017.06.021

39. Spirk D, Stuck AK, Hager A, et al. Electronic alert system for improving appropriate thromboprophylaxis in hospitalized medical patients: a randomized controlled trial. *J Thrombo. Haemost.* 2017;15(11):2138-46. doi: https://dx.doi.org/10.1111/jth.13812

40. Spyropoulos AC, Anderson FA, FitzGerald G, et al. Predictive and Associative Models to Identify Hospitalized Medical Patients at Risk for VTE. *Chest* 2011;140(3):706-14. doi: https://doi.org/10.1378/chest.10-1944

41. Stuck AK, Spirk D, Schaudt J, et al. Risk assessment models for venous thromboembolism in acutely ill medical patients. A systematic review. *Thrombosis and Haemostasis* 2017;117(4):801-08. doi: https://dx.doi.org/10.1160/TH16-08-0631

42. Winoker JS, Paulucci DJ, Anastos H, et al. MP76-07 Predicting complications following robot-assisted partial nephrectomy with the ACS-NSQIP universal surgical risk calculator. *Journal of Urology* 2017;197(4S):e1015.

43. Yale S, Medlin S, Liang H, et al. Risk assessment model for venothromboembolism in post-hospitalized patients. *International Angiology* 2005;24(3):250.

44. Ye F, Stalvey C, Khuddus MA, et al. A systematic review of mobility/immobility in thromboembolism risk assessment models for hospitalized patients. *Journal of Thrombosis and Thrombolysis* 2017;44(1):94-103. doi: https://dx.doi.org/10.1007/s11239-017-1501-5

45. Zakai NA, Callas PW, Repp AB, et al. Venous thrombosis risk assessment in medical inpatients: the medical inpatients and thrombosis (MITH) study. *J Thromb Haemost* 2013;11(4):634-41.

46. Zhou H-X, Peng L-Q, Yan Y, et al. Validation of the Caprini risk assessment model in Chinese hospitalized patients with venous thromboembolism. *Thrombosis Research* 2012;130(5):735-40. doi: https://doi.org/10.1016/j.thromres.2012.08.001

For peer review only

Appendix S3: Summary of predictive performance for studies involving hospital inpatients who required care for cancer, stroke, burn injuries and sepsis or were a mixed medical/surgical cohort

Data source	Endpoint	Incidence	Risk assessment model	Threshold or cut-off	Predictive performance		
					C-statistic (95% CI)	Sensitivity (95% CI)	Specificity (95% CI)
**MIXED INPATIENTS**							
Autar, 2003[22]	DVT	18.9%	•Novel (Autar, 2003)	Risk score ≤6	NR	100 (NR)	100 (NR)
			•Novel (Autar, 2003)	Risk score 7-10	NR	86.0(NR)	68.0 (NR)
			•Novel (Autar, 2003)	Risk score 11-14	NR	68.0 (NR)	31.0 (NR)
			•Novel (Autar, 2003)	Risk score ≥15	NR	25.0 (NR)	10.0 (NR)
Elias, 2017[33]	VTE	0.8%	•Padua (automated)	Risk score ≥5	0.81 (0.79, 0.83)	85.4 (NR)	53.3 (NR)
Chen, 2018[29]	DVT	NA	•Caprini	Risk score ≥4	NR	73.8 (NR)	64.7 (NR)
			•Caprini	Risk score ≥5	NR	62.8 (NR)	82.6 (NR)
			•Padua	Risk score ≥3	NR	53.5 (NR)	82.1 (NR)
			•Padua	Risk score ≥4	NR	42.1 (NR)	92.5 (NR)
**CANCER INPATIENTS**							
Abdel-Razeq, 2010[21]	VTE	3.5%	•Caprini (modified)	Risk score ≥3	NR	100 (NR)	9.2 (NR)
			•Caprini (modified)	Risk score ≥5	NR	57.1 (NR)	53.2 (NR)
Patell, 2017[50]	VTE	3.8%	•Khorana	Risk score ≥3	NR	18.9 (NR)	87.2 (NR)
**BURNS INPATIENTS**							
Pannucci, 2012[49]	VTE	1.0%	•Novel (Pannucci 2012)	NR	0.75	NR	NR
**POST-STROKE INPATIENTS**							
Liu, 2014[42]	DVT	10.5%	•Post-stroke DVT Prediction System	NR	0.65 (0.59, 0.70)	NR	NR
**SEPSIS INPATIENTS**							
Vardi, 2013[58]	VTE	1.3%	•Padua	NR	•All patients<br>0.58 (0.43, 0.73)	•All patients<br>NR	•All patients<br>NR
			•Padua	NR	•No prophylaxis<br>0.54 (0.37, 0.71)	•No prophylaxis<br>NR	•No prophylaxis<br>NR

CI, confidence interval; DVT, deep vein thrombosis; NA, not applicable; NR, not reported; VTE, venous thromboembolism

For peer review only - http://bmjopen.bmj.com/site/about/guidelines.xhtml

PRISMA 2009 Checklist

Section/topic	#	Checklist item	Reported on page #
**TITLE**			
Title	1	Identify the report as a systematic review, meta-analysis, or both.	1
**ABSTRACT**			
Structured summary	2	Provide a structured summary including, as applicable: background; objectives; data sources; study eligibility criteria, participants, and interventions; study appraisal and synthesis methods; results; limitations; conclusions and implications of key findings; systematic review registration number.	2-3
**INTRODUCTION**			
Rationale	3	Describe the rationale for the review in the context of what is already known.	5
Objectives	4	Provide an explicit statement of questions being addressed with reference to participants, interventions, comparisons, outcomes, and study design (PICOS).	5
**METHODS**			
Protocol and registration	5	Indicate if a review protocol exists, if and where it can be accessed (e.g., Web address), and, if available, provide registration information including registration number.	3, 5
Eligibility criteria	6	Specify study characteristics (e.g., PICOS, length of follow-up) and report characteristics (e.g., years considered, language, publication status) used as criteria for eligibility, giving rationale.	6
Information sources	7	Describe all information sources (e.g., databases with dates of coverage, contact with study authors to identify additional studies) in the search and date last searched.	6-7
Search	8	Present full electronic search strategy for at least one database, including any limits used, such that it could be repeated.	Online supplementary - Appendix S1
Study selection	9	State the process for selecting studies (i.e., screening, eligibility, included in systematic review, and, if applicable, included in the meta-analysis).	7
Data collection process	10	Describe method of data extraction from reports (e.g., piloted forms, independently, in duplicate) and any processes for obtaining and confirming data from investigators.	7
Data items	11	List and define all variables for which data were sought (e.g., PICOS, funding sources) and any assumptions and simplifications made.	7
Risk of bias in individual studies	12	Describe methods used for assessing risk of bias of individual studies (including specification of whether this was done at the study or outcome level), and how this information is to be used in any data synthesis.	7-8
Summary measures	13	State the principal summary measures (e.g., risk ratio, difference in means).	8
Synthesis of results	14	Describe the methods of handling data and combining results of studies, if done, including measures of consistency (e.g., I^2) for each meta-analysis.	8

For peer review only

PRISMA 2009 Checklist

Section/topic	#	Checklist item	Reported on page #
Risk of bias across studies	15	Specify any assessment of risk of bias that may affect the cumulative evidence (e.g., publication bias, selective reporting within studies).	7-8
Additional analyses	16	Describe methods of additional analyses (e.g., sensitivity or subgroup analyses, meta-regression), if done, indicating which were pre-specified.	N/A
**RESULTS**			
Study selection	17	Give numbers of studies screened, assessed for eligibility, and included in the review, with reasons for exclusions at each stage, ideally with a flow diagram.	8; Figure 1; Online supplementary - Appendix S2
Study characteristics	18	For each study, present characteristics for which data were extracted (e.g., study size, PICOS, follow-up period) and provide the citations.	9-13
Risk of bias within studies	19	Present data on risk of bias of each study and, if available, any outcome level assessment (see item 12).	14-16
Results of individual studies	20	For all outcomes considered (benefits or harms), present, for each study: (a) simple summary data for each intervention group (b) effect estimates and confidence intervals, ideally with a forest plot.	17-19; Figure 3; Online supplementary – Appendix S3
Synthesis of results	21	Present results of each meta-analysis done, including confidence intervals and measures of consistency.	N/A
Risk of bias across studies	22	Present results of any assessment of risk of bias across studies (see Item 15).	17, Figure 2
Additional analysis	23	Give results of additional analyses, if done (e.g., sensitivity or subgroup analyses, meta-regression [see Item 16]).	N/A
**DISCUSSION**			
Summary of evidence	24	Summarize the main findings including the strength of evidence for each main outcome; consider their relevance to key groups (e.g., healthcare providers, users, and policy makers).	20-22
Limitations	25	Discuss limitations at study and outcome level (e.g., risk of bias), and at review-level (e.g., incomplete retrieval of identified research, reporting bias).	21-22
Conclusions	26	Provide a general interpretation of the results in the context of other evidence, and implications for future research.	22
**FUNDING**			
Funding	27	Describe sources of funding for the systematic review and other support (e.g., supply of data); role of funders for the systematic review.	23

From: Moher D, Liberati A, Tetzlaff J, Altman DG, The PRISMA Group (2009). Preferred Reporting Items for Systematic Reviews and Meta-Analyses: The PRISMA Statement. PLoS Med 6(6): e1000097. doi:10.1371/journal.pmed1000097

For peer review only - http://bmjopen.bmj.com/site/about/guidelines.xhtml

PRISMA 2009 Checklist

For more information, visit: www.prisma-statement.org.

Page 2 of 2

For peer review only

BMJ Open

Risk assessment models for venous thromboembolism in hospitalised adult patients – a systematic review

Journal:	*BMJ Open*
Manuscript ID	bmjopen-2020-045672.R1
Article Type:	Original research
Date Submitted by the Author:	14-Mar-2021
Complete List of Authors:	Pandor, Abdullah; The University of Sheffield, ScHARR<br>Tonkins, Michael; The University of Sheffield, ScHARR<br>Goodacre, Steve; The University of Sheffield, ScHARR<br>Sworn, Katie; The University of Sheffield, ScHARR<br>Clowes, Mark; The University of Sheffield, ScHARR<br>Griffin, Xavier; Queen Mary University of London<br>Holland, Mark; University of Bolton, Department of Clinical and Biomedical Sciences<br>Hunt, Beverley; Guy's and St Thomas' NHS Foundation Trust, Dept of Haematology<br>de Wit, Kerstin; McMaster University Department of Medicine<br>Horner, Daniel; Salford Royal NHS Foundation Trust
**Primary Subject Heading**:	Haematology (incl blood transfusion)
Secondary Subject Heading:	Haematology (incl blood transfusion)
Keywords:	VASCULAR MEDICINE, HAEMATOLOGY, Anticoagulation < HAEMATOLOGY, Quality in health care < HEALTH SERVICES ADMINISTRATION & MANAGEMENT

SCHOLARONE™
Manuscripts

I, the Submitting Author has the right to grant and does grant on behalf of all authors of the Work (as defined in the below author licence), an exclusive licence and/or a non-exclusive licence for contributions from authors who are: i) UK Crown employees; ii) where BMJ has agreed a CC-BY licence shall apply, and/or iii) in accordance with the terms applicable for US Federal Government officers or employees acting as part of their official duties; on a worldwide, perpetual, irrevocable, royalty-free basis to BMJ Publishing Group Ltd ("BMJ") its licensees and where the relevant Journal is co-owned by BMJ to the co-owners of the Journal, to publish the Work in this journal and any other BMJ products and to exploit all rights, as set out in our licence.

The Submitting Author accepts and understands that any supply made under these terms is made by BMJ to the Submitting Author unless you are acting as an employee on behalf of your employer or a postgraduate student of an affiliated institution which is paying any applicable article publishing charge ("APC") for Open Access articles. Where the Submitting Author wishes to make the Work available on an Open Access basis (and intends to pay the relevant APC), the terms of reuse of such Open Access shall be governed by a Creative Commons licence – details of these licences and which Creative Commons licence will apply to this Work are set out in our licence referred to above.

Other than as permitted in any relevant BMJ Author's Self Archiving Policies, I confirm this Work has not been accepted for publication elsewhere, is not being considered for publication elsewhere and does not duplicate material already published. I confirm all authors consent to publication of this Work and authorise the granting of this licence.

For peer review only

Complete manuscript title:

Risk assessment models for venous thromboembolism in hospitalised adult patients – a systematic review

Short title: Predicting VTE in hospitalised patients

Authors:

Abdullah Pandor, ScHARR, The University of Sheffield, Sheffield, UK

Michael Tonkins, ScHARR, The University of Sheffield, Sheffield, UK

Steve Goodacre, ScHARR, The University of Sheffield, Sheffield, UK

Katie Sworn, ScHARR, The University of Sheffield, Sheffield, UK

Mark Clowes, ScHARR, The University of Sheffield, Sheffield, UK

Xavier L Griffin, Queen Mary University of London, London, UK

Mark Holland, University of Bolton, Bolton, UK

Beverley J Hunt, Guy's and St Thomas' NHS Foundation Trust, London, UK

Kerstin de Wit, Department of Medicine, McMaster University, Canada

Daniel Horner, Salford Royal NHS Foundation Trust, Salford, UK

Corresponding author: Abdullah Pandor, ScHARR, The University of Sheffield, Regent Court, 30 Regent Street, Sheffield, S1 4DA, telephone +44 (0) 114 222 0778, email: a.pandor@sheffield.ac.uk

Word count: 3227

For peer review only - http://bmjopen.bmj.com/site/about/guidelines.xhtml

ABSTRACT

INTRODUCTION

Hospital acquired thrombosis accounts for a large proportion of all venous thromboembolism (VTE), with significant morbidity and mortality. This subset of VTE can be reduced through accurate risk assessment and tailored pharmacological thromboprophylaxis. This systematic review aimed to determine the comparative accuracy of risk assessment models (RAMs) for predicting VTE in patients admitted to hospital.

METHODS

A systematic search was performed across five electronic databases (including MEDLINE, EMBASE and the Cochrane Library) from inception to February 2021. All primary validation studies were eligible if they examined the accuracy of a multivariable RAM (or scoring system) for predicting the risk of developing VTE in hospitalised inpatients. Two or more reviewers independently undertook study selection, data extraction, and risk of bias assessments using the PROBAST tool. We used narrative synthesis to summarise the findings.

RESULTS

Among 6355 records, we included 51 studies, comprising 24 unique validated RAMs. The majority of studies included hospital inpatients who required medical care (21 studies), were undergoing surgery (15 studies) or receiving care for trauma (4 studies). The most widely evaluated RAMs were the Caprini RAM (22 studies), Padua prediction score (16 studies), IMPROVE models (8 studies), the Geneva risk score (4 studies) and the Kucher score (4 studies). C-statistics varied markedly between studies and between models, with no one RAM performing obviously better than other models. Across all models, C-statistics were often weak (<0.7), sometimes good (0.7-0.8) and a few were excellent (>0.8). Similarly, estimates for sensitivity and specificity were highly variable. Sensitivity estimates ranged from 12.0% to 100% and specificity estimates ranged from 7.2% to 100%.

For peer review only - http://bmjopen.bmj.com/site/about/guidelines.xhtml

CONCLUSION

Available data suggest that RAMs have generally weak predictive accuracy for VTE. There is insufficient evidence and too much heterogeneity to recommend the use of any particular RAM.

PROSPERO REGISTRATION

Steve Goodacre, Abdullah Pandor, Katie Sworn, Daniel Horner, Mark Clowes. A systematic review of venous thromboembolism RAMs for hospital inpatients. PROSPERO 2020 CRD42020165778 Available from https://www.crd.york.ac.uk/prospero/display_record.php?RecordID=165778

KEYWORDS

Hospitalization, inpatients, risk assessment, systematic review, venous thromboembolism

For peer review only

For peer review only - http://bmjopen.bmj.com/site/about/guidelines.xhtml

ARTICLE SUMMARY

Strengths and limitations of this study

- This systematic review provides an up-to-date comprehensive review of risk assessment models for predicting venous thromboembolism in patients admitted to hospital.
- The newly developed PROBAST tool was used to evaluate the risk of bias and applicability of the available evidence.
- Heterogeneity in the included studies (participants, inclusion criteria, clinical condition, outcome definition and measurement) and variable reporting of items precluded meta-analysis.
- Limitations of the existing evidence and areas of future research are highlighted.

For peer review only

For peer review only - http://bmjopen.bmj.com/site/about/guidelines.xhtml

INTRODUCTION

Venous thromboembolism (VTE) is an important and life threatening complication of hospitalisation and illness, and is associated with significant morbidity and mortality.[1 2] Globally, an estimated 10 million VTE episodes are diagnosed each year; over half of these episodes are associated with hospital inpatients stays and result in significant loss of disability adjusted life years.[3 4] Consequently, there has been a substantial and sustained focus on VTE prevention over the last three decades, with good evidence indicating a reduction in morbidity with primary thromboprophylaxis in hospitalised patients[5-8] Despite this evidence, thromboprophylaxis remains either underused or inappropriately applied.[9]

Risk assessment models (RAMs) have been developed to help stratify the risk of VTE amongst hospitalised patients.[10] These models use clinical information from the patient's history and examination to identify those with an increased risk of developing VTE who are most likely to benefit from pharmacological prophylaxis. Inappropriate use of VTE prophylaxis may not reduce VTE rates and may cause unnecessary harm.[11] Whilst RAMs could improve the ratio of benefit to risk and benefit to cost, it is unclear which VTE RAM should be applied to guide decision-making for prophylaxis in clinical practice and thereby optimise patient care.

The current review extends and updates three broadly overlapping existing reviews.[10 12 13] Whilst these reviews identified the use of various (derived and validated) RAMs for VTE in hospitalised patients, they did not find any evidence to suggest which RAM was superior. The aim of this systematic review was to identify primary validation studies (as derivation studies may give an overoptimistic assessment of model performance measures) and determine the accuracy of individual RAMs for predicting the risk of developing VTE in hospital inpatients.

METHODS

A systematic review was undertaken in accordance with the general principles recommended in the Preferred Reporting Items for Systematic Reviews and Meta-Analyses (PRISMA) statement.[14] This

For peer review only - http://bmjopen.bmj.com/site/about/guidelines.xhtml

review was part of a larger project on VTE RAMs for hospital inpatients[15] and was registered on the International Prospective Register of Systematic Reviews (PROSPERO) database (CRD42020165778).

Eligibility criteria

We sought studies evaluating RAMs which could be applied to a general inpatient population (medical, surgical or trauma), rather than disease-specific models. All primary validation studies that evaluated the accuracy (e.g. sensitivity, specificity, C-statistic) of a multivariable RAM (or scoring system) for predicting the risk of developing VTE were eligible for inclusion. We selected studies that included validation of the model in a group of patients that were not involved in model derivation. This involved either splitting the study cohort (internal) or using a new cohort (external). The study could have reported derivation of the model but we only used the validation data to estimate accuracy. The study population consisted of hospital inpatients including those who required medical care, undergoing any surgery (excluding day surgery) or received care following an injury. Studies that primarily focussed on children (aged under 16 years), women admitted to hospital for pregnancy-related reasons and any patient admitted to a level 2 or above critical care environment (e.g. patients requiring more detailed observation or intervention including support for a single failing organ system or post-operative care and those 'stepping down' from higher levels of care) were excluded. These patient groups have VTE risk profiles that differ markedly from the general inpatient population, making the use of a generic model inappropriate.

Data sources and searches

Potentially relevant studies were identified through searches of five electronic databases including MEDLINE (with MEDLINE In-process and Epub Ahead of Print), EMBASE and the Cochrane Library. The search strategy used free text and thesaurus terms and combined synonyms relating to the condition (e.g. VTE in medical inpatients) with risk prediction modelling terms. No language restrictions were used. However, as the current review updated three previous systematic reviews,[10 12 13] searches were limited by date from 2017 (last search date from earlier reviews)[10] to February

For peer review only - http://bmjopen.bmj.com/site/about/guidelines.xhtml

2021. Searches were supplemented by hand-searching the reference lists of all relevant studies (including existing systematic reviews); forward citation searching of included studies; contacting key experts in the field; and undertaking targeted searches of the World Wide Web using the Google search engine. Further details on the search strategy can be found in **online supplementary Appendix S1.**

Study selection

All titles were examined for inclusion by one reviewer (KS) and any citations that clearly did not meet the inclusion criteria (e.g. non-human, unrelated to VTE inpatients) were excluded. All abstracts and full text articles were then examined independently by two reviewers (KS and AP). Any disagreements in the selection process were resolved through discussion or if necessary, arbitration by a third reviewer (SG) and included by consensus.

Data extraction and quality assessment

Data relating to study design, methodological quality and outcomes were extracted by one reviewer (KS) into a standardised data extraction form and independently checked for accuracy by a second (AP or MT). Any discrepancies were resolved through discussion to achieve agreement. Where differences were unresolved, a third reviewer's opinion was sought (SG). Where multiple publications of the same study were identified, data were extracted and reported as a single study.

The methodological quality of each included study was assessed using PROBAST (Prediction model Risk Of Bias ASsessment Tool).[16 17] This instrument evaluates four key domains: patient selection, predictors, outcome, and analysis. Each domain is assessed in terms of risk of bias and the concern regarding applicability to the review (first three domains only). To guide the overall domain-level judgement about whether a study is at high, low or an unclear (in the event of insufficient data in the publication to answer the corresponding question) risk of bias, sub-domains within each domain include a number of signalling questions to help judge with bias and applicability concerns. An overall risk of bias for each individual study was defined as low risk when all domains were judged as

For peer review only - http://bmjopen.bmj.com/site/about/guidelines.xhtml

low; and high risk of bias when one or more domains were considered as high. Studies were assigned an unclear risk of bias if one or more domains were unclear and all other domains were low.

Data synthesis and analysis

We were unable to perform meta-analysis due to significant levels of heterogeneity between studies (participants, inclusion criteria, clinical condition) and variable reporting of items. As a result, a pre-specified narrative synthesis approach[18 19] was undertaken, with data being summarised in tables with accompanying narrative summaries that included a description of the included variables, statistical methods and performance measures (e.g. sensitivity, specificity and C-statistic [a value between 0.7 to 0.8 and >0.8 indicated good and excellent discrimination, respectively; and values <0.7 were considered weak[20]]), where applicable. All analyses were conducted using Microsoft Excel 2010 (Microsoft Corporation, Redmond, WA, USA).

Patient and public involvement

Patients and the public were not involved in the design or conduct of this systematic review.

RESULTS

Study flow

Figure 1 summarises the process of identifying and selecting relevant literature. Of the 6355 citations identified, 51 studies investigating 24 unique RAMs met the inclusion criteria. The majority of the articles were excluded primarily for not using a RAM for predicting the risk of developing VTE, having no useable or relevant outcome data or an inappropriate study design (e.g. derivation study, reviews, commentaries or editorials). A full list of excluded studies with reasons for exclusion is provided in **online supplementary Appendix S2.**

[INSERT FIGURE 1]

For peer review only - http://bmjopen.bmj.com/site/about/guidelines.xhtml

Study and patient characteristics

The design and participant characteristics of the 51 included studies[21-71] are summarised in **Table 1**. All studies were published between 2003 and 2020 and were undertaken in North America (n=24),[23 25 33-40 43 47 48 52-59 65 68 69] Asia (n=13),[29 30 42 44-46 60-63 67 70 71] Europe (n=9),[22 24 26-28 31 49 51 66] the Middle-East (n=2),[21 64] South America (n=1),[32] Australia (n=1)[41] and one study was intercontinental.[50] Sample sizes ranged from 70[40] to 1,099,093[43] patients in 37 observational cohort studies (11 prospective[21 22 24 28 29 32 44 51 52 56 64] [5 of which were multicentre] and 26 retrospective[23 25-27 33 34 36-41 43 46 49 50 53-55 58 59 62 63 65 68 69] [16 of which were multicentre] in design). Sample sizes in 14 case control studies[30 31 35 42 45 47 48 57 60 61 66 67 70 71] (4 of which were multicentre) ranged from 148[61] to 19,217[57] patients.

For peer review only - http://bmjopen.bmj.com/site/about/guidelines.xhtml

Table 1: Study and population characteristics

Author, year	Country	Design	Single/ multi-centre	Sample size	Population	Mean age (years)	Male	VTE prophylaxis	RAMs	Target condition (risk period)	Incidence	Validation methodology
Autar, 2003[22]	UK	P,CS	Single	148	Hospitalised patients from orthopaedic, medical, and surgical specialties	NR	NR	50%	•Novel (Autar, 2003)	DVT, not defined (90 days)	18.9%	External
Rogers, 2007[56]	USA	P,CS	Multi	91,308	Hospitalised surgical patients (undergoing vascular and general surgery)	NR	NR	NR	•Novel (Rogers, 2007)	VTE (30 days)	0.6%	Internal: split (half)
Abdel-Razeq, 2010[21]	Jordan	P,CS	Single	606	Hospitalised (>24 hours) cancer patients aged ≥18 years	51	51%	55%	•Caprini (modified)	VTE, symptomatic (60 days)	3.5%	External
Bahl, 2010[23]	USA	R,CS	Multi	8,216	Hospitalised surgical patients (undergoing general, vascular, and urologic surgery)	NR	NR	NR	•Caprini	VTE, (30 days)	1.4%	External
Barbar, 2010[24]	Italy	P,CS	Single	1,180	Hospitalised medical patients	NR	47%	16%	•Padua	VTE, symptomatic (90 days)	3.1%	External
Rothberg, 2011[58]	USA	R,CS	Multi	48,540	Hospitalised (≥ 3 days) medical patients aged ≥18 years	NR	NR	30%	•Novel (Rothberg 2011)	VTE, hospital associated (NR)	0.5%	Internal: split (20%)
Woller, 2011[69]	USA	R,CS	Multi	46,856	Hospitalised medical patients aged ≥18 years	61	46%	NR	•Intermountain<br>•Kucher	VTE, defined by ICD-9 codes (90 days)	4.5%	Internal: split (25%)
Pannucci, 2012[53]	USA and Canada	R,CS	Multi	5,761	Hospitalised (>2 days) patients with a burn injury aged ≥18 years	46	69%	NR	•Novel (Panunucci 2012)	VTE, not defined (NR)	1.0%	Internal: split (25%)
Rogers, 2012[55]	USA	R,CS	Multi	234,032	Hospitalised trauma patients	NR	NR	NR	•TESS	VTE (NR)	NR	Internal: split
Bilimoria, 2013[25]	USA	R,CS	Multi	88,053	Hospitalised surgical patients (undergoing colorectal surgery)	NR	NR	NR	•ACS NSQIP - Colon specific<br>•ACS NSQIP - Universal	DVT, not defined (30 days)	2.3%	External: split (by year)
Hegsted, 2013[39]	USA	R,CS	Single	2,281	Hospitalised (≥2 days) trauma patients aged ≥13 years	45	70%	NR	•RAP	DVT, not defined or PE (NR)	•DVT: 10.5%<br>•PE: 1.5%	External
Vardi, 2013[64]	Israel	P,CS	Single	1,080	Hospitalised (≥2 days) sepsis patients aged >18 years	75	52%	18%	•Padua	VTE, hospital associated (NR)	1.3%	External
Ho, 2014[41]	Australia	R,CS	Single	357	Hospitalised major trauma patients	NR	75%	NR	•TESS	VTE, symptomatic (NR)	20.7%	External
Liu, 2014[44]	China	P,CS	Single	287	Hospitalised acute stroke patients aged >18 years	NR	63%	22%	•Post-stroke DVT Prediction System	DVT (14±3 days)	10.5%	Internal: split (33%)
Mahan, 2014[47]	USA	CC	Multi	417	Hospitalised (≥3 days) medical patients aged ≥18 years	NR	49%	NR	•IMPROVE (7-factor)	VTE, hospital associated (92 days)	NA	External
Nendaz, 2014[51]	Switzerland	P,CS	Multi	1,478	Hospitalised (>24 hours) medical patients aged ≥18 years	65	53%	57%	•Geneva<br>•Padua	VTE, symptomatic including PE or DVT (90 days)	2.0%	External
Pannucci, 2014[52]	USA	P,CS	Multi	3,576	Hospitalised surgical patients aged ≥18 years	NR	NR	66%	•Novel (Panunucci 2014)	VTE (90 days)	1.4%	Internal: split (35%)

For peer review only - http://bmjopen.bmj.com/site/about/guidelines.xhtml

For peer review only

Rosenberg, 2014[57]	USA	CC	Multi	19,217	Hospitalised (≥3 days) medical patients aged ≥18 years	NR	47%	43%	•IMPROVE (7-factor)	VTE, defined by ICD-9 codes (90 days)	NA	External
Zhou 2014[71]	China	CC	Single	998	Hospitalised (≥2 days) medical patients aged >18 years	NR	58%	15%	•Caprini<br>•Padua	VTE, defined by ICD-10 codes (NR)	NA	External
Hewes, 2015[40]	USA	R,CS	Single	70	Hospitalised cancer patients (undergoing oesophagectomy)	NR	83%	96%	•Caprini (modified)	VTE (60 days)	14.3%	External
de Bastos, 2016[32]	Brazil	P,CS	Single	11,091	Hospitalised medical patients aged >18 years	50	61%	0%	•Caprini	VTE, symptomatic (NR)	0.3%	External
Grant, 2016[36]	USA	R,CS	Multi	63,548	Hospitalised (≥2 days) medical patients aged ≥18 years	66	45%	61%	•Caprini	VTE, hospital associated (90 days)	1.1%	External
Greene, 2016[37]	USA	R,CS	Multi	63,548	Acutely ill, hospitalised (≥2 days) medical patients aged ≥18 years	66	45%	61%	•IMPROVE (4-factor)<br>•Intermountain<br>•Kucher<br>•Padua	VTE, hospital associated (90 days)	1.1%	External
Hachey, 2016[38]	USA	R,CS	Single	232	Hospitalised surgical patients (undergoing segmenectomy, lobectomy or pneumonectomy for lung cancer)	NR	43%	92%	•Caprini	VTE (60 days)	5.2%	External
Lui, 2016[45]	China	CC	Single	640	Hospitalised (>2 days) medical patients aged ≥18 years	NR	52%	NR	•Caprini<br>•Padua	VTE (NR)	N/A	External
Lobastov, 2016[46]	Russia	R,CS [a]	Multi	140	Hospitalised high risk emergency surgery patients (undergoing general and neurosurgery)	69	49%	100%	•Caprini	DVT or PE, new (NR)	27.9%	External
Shaikh, 2016[59]	USA	R,CS	Multi	1,598	Hospitalised surgical patients (undergoing plastic surgery)	50	19%	34%	•Caprini	VTE, not defined (30 days)	1.5%	External
Elias, 2017[34]	USA	R,CS	Single	30,726	Hospitalised (>2 days) medical and surgical patients	NR	44%	21%	•Padua (automated)	VTE, defined by ICD-9 codes (NR)	0.8%	External
Frankel, 2017 (abstract)[35]	USA	CC	NR	149	Hospitalised surgical patients aged ≥18 years (undergoing robotic assisted laparoscopic prostatectomy)	NR	NR	NR	•Caprini	VTE, not defined (90 days)	NA	External
Krasnow, 2017 (abstract)[43]	USA	R,CS	Multi	1,099,093	Hospitalised surgical patients (major urological cancer surgery)	NR	NR	NR	•Caprini	VTE, symptomatic (90 days)	1.2%	External
Patell, 2017[54]	USA	R,CS	Single	2,780	Hospitalised (>24 hours) cancer patients aged >18 years	62 (median)	56%	65%	•Khorana	VTE, defined by ICD-9 codes (NR)	3.8%	External
Winoker, 2017[68]	USA	R,CS	Multi	300	Hospitalised surgical patients (undergoing urological surgery using robot-assisted partial nephrectomy)	61 (median)	62%	NR	•ACS NSQIP - Universal	VTE, not defined (NR)	0.3%	External
Blondon, 2018[28]	Switzerland	P,CS	Multi	1,478	Hospitalised (>24h) medical patients aged ≥18 years	65	53%	59%	•IMPROVE (7-factor)<br>•Geneva [b]<br>•Padua [b]	VTE, symptomatic including PE or DVT (90 days)	2.0%	External

For peer review only - http://bmjopen.bmj.com/site/about/guidelines.xhtml

Chen, 2018[30]	China	CC	Single	390	Hospitalised (>2 days) patients aged ≥18 years with and without DVT	NR	51%	41%	•Caprini<br>•Padua	DVT (NR)	NA	External
Dornbus, 2018 (abstract)[33]	USA	R,CS	NR	2,830	Hospitalised surgical patients (undergoing neurosurgery)	NR	NR	NR	•Caprini	VTE, not defined (NR)	NR	External
Vaziri, 2018[65]	USA	R,CS	Single	1,006	Hospitalised surgical patients (undergoing neurosurgery)	NR	46%	NR	•ACS NSQIP- Universal	VTE, not defined (NR)	1.3%	External
Vincentelli, 2018[66]	Italy	CC	Multi	1,215	Acutely ill, hospitalised medical patients aged >18 years	NR	44%	NR	•Chopard<br>•Kucher<br>•Padua	VTE (NR)	NA	External
Zhou, 2018[70]	China	CC	Single	1,804	Hospitalised (≥2 days) medical patients aged >18 years	NR	59%	5%	•Caprini<br>•Padua	VTE, defined by ICD-10 codes (NR)	NA	External
Blondon, 2019a[26]	Italy	R,CS [a]	Single	1,180	Hospitalised medical patients	72	47%	20%	•Geneva (simplified)	VTE, symptomatic (90 days)	3.1%	External
Blondon, 2019b (abstract)[27]	Switzerland	R,CS [a]	Multi	991	Hospitalised elderly medical patients	75	55%	NR	•Geneva (simplified)<br>•IMPROVE (NR)<br>•Padua	VTE, symptomatic (NR)	15.0%	External
Cobben, 2019[31]	Netherlands	CC	Multi	556	Hospitalised (>24h) medical patients	NR	52%	NR	•Caprini<br>•Geneva<br>•IMPROVE (4-factor)<br>•IMPROVE (7-factor)<br>•Intermountain<br>•Kucher<br>•Lecumberri<br>•NAVAL<br>•NICE Guideline<br>•Padua<br>•PRETEMED Guideline<br>•Zakai et al (model 2)	VTE (NR)	NA	External
Tachino, 2019[62]	Japan	R,CS	Multi	859	Hospitalised (>24h) trauma patients aged ≥18 years	NR	64%	NR	•RAP<br>•Quick RAP	VTE (NR)	3.0%	External (RAP)/internal (qRAP)
Tian, 2019[63]	China	R,CS	Single	533	Hospitalised surgical patients (undergoing thoracic surgery)	53	53%	0%	•Caprini<br>•Khorana<br>•Padua<br>•Novel (Rogers, 2007)	VTE (NR)	8.4%	External
Bo, 2020[29]	China	P,CS	Multi	24,524	Hospitalised (≥2 days) patients from medical and surgical specialties aged ≥18 years	57	57	NR	•Caprini	DVT (NR)	0.9%	External
Hu, 2020[42]	China	CC	Single	442	Hospitalised (≥2 days) cancer patients aged ≥18 years	NR	62	3.8	•Caprini<br>•Khorana	VTE, defined by ICD-10 codes (NR)	NA	External
Mlaver, 2020[48]	USA	CC	Single	189	Hospitalised surgical patients (undergoing hepatobiliary, colorectal, endocrine, plastic, transplant, or general surgery)	NR	NR	NR	•Caprini<br>•Padua	VTE, not defined (NR)	NA	External

For peer review only - http://bmjopen.bmj.com/site/about/guidelines.xhtml

Moumneh, 2020[49]	France	R,CS [a]	Multi	14,660	Acutely ill, hospitalised (≥2 days) medical patients aged ≥40 years	73	50	46.1	•Caprini<br>•Padua<br>•IMPROVE (7 factor)	VTE, symptomatic including PE or DVT (90 days)	1.8%	External
Nafee, 2020[50]	35 countries	R,CS [a]	Multi	6,459	Hospitalised medical patients	76	45	100	•IMPROVE (NR)<br>•Novel (Nafee, 2020a)<br>•Novel (Nafee, 2020b)	VTE (77 days)	6.3%	External
Shang, 2020[60]	China	CC	Single	2,878	Hospitalised (≥2 days) cancer patients aged ≥18 years	56	47	NR	•Caprini (2009)<br>•Caprini (2013)	VTE, (NR)	NA	External
Shen, 2020[61]	China	CC	Single	148	Hospitalised (≥2 days) medical patients aged ≥18 years	NR	NR	0	•Novel (Shen 2020)	VTE, not defined (NR)	NA	Internal: split (by time, months)
Wang, 2020[67]	China	CC	Single	1,579	Hospitalised (≥3 days) medical patients aged ≥18 years	53	57	NR	•Padua	VTE, (NR)	NA	Internal: split (by year, months)

[a] Prospective cohort study with retrospective analysis, thus classified as retrospective cohort study
[b] Data overlap with Nendaz, 2014[51]
ACS NSQIP, American College of Surgeons National Surgical Quality Improvement Program; CC, case-control; CS, cohort study; DVT, deep vein thrombosis; NA, not applicable; NR, not reported; P, prospective; PE, pulmonary embolism; R, retrospective; RAMs, risk assessment models; RAP, Risk Assessment Profile; TESS, Trauma Embolic Scoring System VTE, venous thromboembolism

For peer review only

The vast majority of studies evaluated VTE risk assessment in hospital inpatients who required medical care (n=21),[24 26-28 31 32 36 37 45 47 49-51 57 58 61 66 67 69-71] were undergoing surgery (n=15)[23 25 33 35 38 40 43 46 48 52 56 59 63 65 68] or were a mixed medical and surgical cohort (n=4).[22 29 30 34] The remaining studies focused on patients receiving care for trauma (n=4),[39 41 55 62] cancer (n=4),[21 42 54 60] stroke (n=1),[44] burn injuries (n=1)[53] and sepsis (n=1).[64]. The mean age ranged from 45 years[39] to 76 years[50] (not reported in 29 studies)[22-25 30 31 33-35 38 40-45 47 48 52 55-58 61 62 65 66 70 71] and the proportion of female subjects ranged from 17%[40] to 81%[59] (not reported in 12 studies).[22 23 25 33 35 43 48 52 55 56 58 61]

VTE definition and case ascertainment

The majority of studies (n=37)[21 23 24 26-32 36-38 40-47 49-52 55-58 60 62-64 66 67 70 71] defined the VTE endpoint (DVT and or PE) as being objectively confirmed. Of the remainder, three studies[34 54 69] had no objective confirmation of VTE and 11 studies[22 25 33 35 39 48 53 59 61 65 68] did not report the methods for diagnosis confirmation. In terms of VTE risk period, half of the studies (n=23)[21-26 28 35-38 40 43 44 47 49-52 56 57 59 69] utilised the RAMs to predict the occurrence of VTE within 3 months of the index hospitalisation. The remaining studies did not report the VTE risk period. The reported incidence of VTE ranged widely from 0.3%[32 68] to 27.9%[46] depending on definition, study design and study participants (e.g. medical, surgical or trauma).

RAMs

The studies included in this review evaluated 24 validated unique RAMs. The most widely evaluated models were the Caprini RAM (22 studies),[21 23 29-33 35 36 38 40 42 43 45 46 48 49 59 60 63 70 71] Padua prediction score (16 studies),[24 27 28 30 31 34 37 45 48 49 63 64 66 67 70 71] IMPROVE models (8 studies),[27 28 31 37 47 49 50 57] the Geneva risk score (4 studies)[26-28 31] and the Kucher score (4 studies).[31 37 66 69] A summary of their associated characteristics and composite clinical variables is provided in **online supplementary Appendix S3.**

Statistical methods

For peer review only - http://bmjopen.bmj.com/site/about/guidelines.xhtml

Statistical methods varied significantly between studies. Most studies reported the discrimination of the RAMs using a combination of the C-statistic and sensitivity or specificity. A minority reported calibration measures, such as the Hosmer-Lemeshow test.[23 40 41 50]

Risk of bias and applicability assessment

The overall methodological quality of the 51 included studies[21-71] is summarised in **Table 2 and Figure 2**. The methodological quality of the included studies was variable, with most studies having high or unclear risk of bias in at least one item of the PROBAST tool. The main sources of potential bias were related to the following domains:

1) Patient selection factors, such as retrospective data collection, incomplete patient enrolment, or unclear criteria for patients receiving VTE prophylaxis;

2) Predictor and outcome bias arising from inappropriate inclusion of predictors within RAMs, unclear methods of outcome definition, low event rates, and missing predictor or outcome data;

3) Analysis factors, such as small sample sizes, inappropriate handling of missing data and failure in reporting relevant performance measures such as calibration.

Assessment of applicability to the review question led to the majority of studies being classed either as high (n=35)[21 22 29 30 32 34 35 38-49 52-55 59-68 70 71] or unclear (n=12)[23 24 27 28 31 33 50 51 56-58 69] risk of inapplicability. These assessments were generally related to patient selection (highly selected study populations e.g. single pathologies, single site settings), predictors (inconsistency in definition, assessment or timing of predictors) and outcome determination.

For peer review only

For peer review only - http://bmjopen.bmj.com/site/about/guidelines.xhtml

Table 2. Summary of each study's risk of bias and applicability concern using the PROBAST tool – Review authors' judgements

Author, year	Risk of Bias				Concern regarding Applicability			OVERALL	OVERALL
	1. Participant selection	2. Predictors	3. Outcome	4. Analysis	1. Participant selection	2. Predictors	3. Outcomes	RISK OF BIAS	APPLICABILITY
Abdel-Razeq, 2010[21]	High	High	High	High	High	High	High	High	High
Autar, 2003[22]	High	High	High	High	High	High	High	High	High
Bahl, 2010[23]	High	High	High	High	Unclear	Unclear	Unclear	High	Unclear
Barbar, 2010[24]	Low	Unclear	Unclear	High	Low	Unclear	Unclear	High	Unclear
Bilimoria, 2013[25]	Low	Low	Low	High	Low	Low	Low	High	Low
Blondon, 2019a[26]	Low	Unclear	High	High	Low	Low	Low	High	Low
Blondon, 2019b (abstract)[27]	Unclear	Unclear	Unclear	Unclear	Unclear	Unclear	Unclear	Unclear	Unclear
Blondon, 2018[28]	Low	Unclear	Unclear	High	Unclear	Low	Unclear	High	Unclear
Bo, 2020[29]	Low	Unclear	Unclear	Unclear	High	Low	Low	Unclear	High
Chen, 2018[30]	High	High	High	High	Unclear	High	High	High	High
Cobben, 2019[31]	Unclear	Unclear	High	High	Unclear	Low	Unclear	High	Unclear
de Bastos, 2016[32]	High	Low	High	High	High	Low	Low	High	High
Dornbus, 2018 (abstract)[33]	High	Unclear	High	Unclear	Unclear	Unclear	Unclear	High	Unclear
Elias, 2017[34]	High	Unclear	High	High	Low	Low	High	High	High
Frankel, 2017 (abstract)[35]	High	Unclear	Unclear	High	High	Unclear	Unclear	High	High
Grant, 2016[36]	High	Unclear	Unclear	Unclear	Low	Low	Low	High	Low
Greene, 2016[37]	Unclear	Unclear	Unclear	Unclear	Low	Low	Low	Unclear	Low
Hachey, 2016[38]	High	Unclear	Unclear	High	High	Low	High	High	High
Hegsted, 2013[39]	High	Unclear	High	High	High	Low	Unclear	High	High
Hewes, 2015[40]	High	Unclear	Unclear	High	High	Unclear	Low	High	High
Ho, 2014[41]	Unclear	Unclear	Unclear	High	High	Unclear	Unclear	High	High
Hu, 2020[42]	Unclear	Unclear	Unclear	Unclear	High	Unclear	Unclear	Unclear	High
Krasnow, 2017 (abstract)[43]	Unclear	Unclear	Unclear	Unclear	High	Unclear	Unclear	Unclear	High
Liu, 2014[44]	Low	Low	Unclear	Unclear	High	High	High	Unclear	High
Liu, 2016[45]	High	Unclear	High	High	High	Low	Low	High	High
Lobastov, 2016[46]	Unclear	Unclear	Unclear	High	High	Low	High	High	High
Mahan, 2014[47]	Low	Unclear	Unclear	Unclear	High	Low	Unclear	Unclear	High
Mlaver, 2020[48]	Unclear	Unclear	Unclear	Unclear	High	Unclear	Unclear	Unclear	High
Moumneh, 2020[49]	High	Unclear	Unclear	Low	High	Low	Low	High	High
Nafee, 2020[50]	Unclear	Low	Low	Low	Unclear	Low	Low	Unclear	Unclear
Nendaz, 2014[51]	Low	Unclear	Low	High	Low	Unclear	Low	High	Unclear
Pannucci, 2012[53]	High	Unclear	Unclear	High	High	High	Unclear	High	High
Pannucci, 2014[52]	Low	Unclear	High	High	High	Low	Low	High	High
Patell, 2017[54]	High	Unclear	Unclear	High	High	Unclear	Unclear	High	High
Rogers, 2007[56]	Unclear	Unclear	Unclear	High	Low	Unclear	Unclear	High	Unclear
Rogers, 2012[55]	High	High	Unclear	High	High	High	Unclear	High	High
Rosenberg, 2014[57]	Low	Unclear	Unclear	Unclear	Unclear	Unclear	Unclear	Unclear	Unclear
Rothberg, 2011[58]	High	Unclear	Unclear	High	Low	Unclear	Unclear	High	Unclear
Shaikh, 2016[59]	High	Unclear	High	High	High	Unclear	High	High	High
Shang, 2020[60]	Low	Unclear	Unclear	Unclear	High	Unclear	Unclear	Unclear	High

Shen, 2020[61]	Unclear	High	Unclear	Unclear	High	Unclear	Unclear	High	High
Tachino, 2019[62]	High	Unclear	Unclear	High	High	Unclear	Unclear	High	High
Tian, 2019[63]	High	Unclear	High	High	High	High	High	High	High
Vardi, 2013[64]	Unclear	Low	Low	High	High	Low	Low	High	High
Vaziri, 2018[65]	Unclear	Unclear	Unclear	High	High	Unclear	Unclear	High	High
Vincentelli, 2018[66]	High	Low	Unclear	High	High	Low	Unclear	High	High
Wang, 2020[67]	Low	Unclear	Unclear	Unclear	High	Unclear	Unclear	Unclear	High
Winoker, 2017[68]	High	Unclear	Unclear	High	High	High	High	High	High
Woller, 2011[69]	High	High	Unclear	High	Unclear	Unclear	Unclear	High	Unclear
Zhou 2014[71]	Unclear	Unclear	Unclear	High	High	Unclear	Unclear	High	High
Zhou, 2018[70]	Low	High	High	High	High	Unclear	Unclear	High	High

For peer review only

[INSERT FIGURE 2]

Predictive performance of VTE RAMs (summary of results)

As there were a reasonable number of studies to compare, a summary of the C-statistics for studies involving medical, surgical and trauma patients respectively is presented in **Figures 3a-c**, with the results grouped by RAM. Results of other hospital inpatients are presented in **online supplementary Appendix S4**. C-statistics varied markedly between these studies and between models, with no RAM performing obviously better than other models. In studies evaluating a single model, C-statistics[20] were sometimes weak (<0.7; 10 studies with 17 data points), often good (0.7-0.8; 17 studies with 20 data points) and a few were excellent (>0.8; 5 studies with 5 data points). There was marked heterogeneity between multiple studies evaluating the same model. Studies evaluating multiple (more than 3) models [31 37] tended to report weak accuracy across all the models (C-statistic <0.7; 2 studies with 16 data points).

[INSERT FIGURE 3]

Table 3 shows the sensitivity and specificity at various thresholds for studies involving medical, surgical and trauma patients respectively, with the results grouped by RAM. Interpretation was again limited by marked heterogeneity, which was exacerbated when different thresholds were reported by different studies evaluating the same model. Model accuracy was generally poor, with high sensitivity usually reflecting a threshold effect, as evidenced by corresponding low specificity (and vice versa).

For peer review only - http://bmjopen.bmj.com/site/about/guidelines.xhtml

Table 3. Sensitivity and specificity for studies involving medical, surgical and trauma inpatients

Risk assessment models	Threshold or cut-off	Endpoint	Data source	Sensitivity (95% CI)	Specificity (95% CI)
**MEDICAL INPATIENTS**					
**Caprini (7 studies)**	Risk Score ≥3	VTE	Lui, 2016[45]	70.9% (NR)	73.4% (NR)
	Risk Score ≥3	VTE	Moumneh, 2020[49]	98.1% (95.6, 99.4)	7.5% (7.1, 8.0)
	Risk Score ≥3	VTE	Zhou 2014[71]	82.3% (NR)	60.4% (NR)
	Risk Score ≥3	VTE	Zhou, 2018[70]	84.3% (NR)	66.2% (NR)
	Risk Score ≥5	VTE	Zhou, 2018[70]	57.1% (NR)	24.6% (NR)
	Risk score ≥5	VTE	Grant, 2016[36]	69.7% (NR)	50.28% (NR)
	Risk score ≥7	VTE	Grant, 2016[36]	42.69% (NR)	74.71% (NR)
	Risk score ≥9	VTE	Grant, 2016[36]	18.51% (NR)	89.03% (NR)
	NR[a]	VTE	de Bastos, 2016[32]	86.5% (NR)	47.0% (NR)
	NR	VTE	Cobben, 2019[31]	88.6% (NR)	21.4% (NR)
**Chopard (1 study)**	Risk Score ≥3	VTE	Vincentelli, 2018[66]	64.2% (38.4, 81.9)	57.7% (63.9, 79.4)
**Geneva models (4 studies)**	Risk Score ≥3	VTE	Blondon, 2018[28]; Nendaz, 2014[51]	All patients: 90.0% (73.5, 97.9)	All patients: 35.3% (32.8, 37.8)
				No prophylaxis: 85% (NR)	No prophylaxis: NR
	NR	VTE	Cobben, 2019[31]	75.0% (NR)	34.1% (NR)
	Simplified model: Risk Score ≥3	VTE	Blondon, 2019a[26]	95.0% (NR)	44.0% (NR)
	Simplified model: NR	VTE	Blondon, 2019b (abstract)[27]	86.4% (NR)	NR
**IMPROVE models (4 studies)**	4-factor model: NR	VTE	Cobben, 2019[31]	27.9% (NR)	85.4% (NR)
	7-factor model: Risk score ≥2	VTE	Moumneh, 2020[49]	73.8% (68.0, 79.0)	47.1% (46.3, 47.9)
	7-factor model: Risk score 2-3	VTE	Blondon, 2018[28]; Nendaz, 2014[51]	All patients: 87% (NR)	All patients: NR
				No prophylaxis: 85% (NR)	No prophylaxis: NR
	7-factor model: Risk score ≥3	VTE	Blondon, 2018[28]; Nendaz, 2014[51]	All patients: 73% (NR)	All patients: NR
				No prophylaxis: 54% (NR)	No prophylaxis: NR
	7-factor model: Risk score ≥4	VTE	Moumneh, 2020[49]	24.7% (19.6, 30.4)	85.5% (84.9, 86.1)
	7-factor model: NR	VTE	Cobben, 2019[31]	63.3% (NR)	70.7% (NR)
	NR	VTE	Blondon, 2019b (abstract)[27]	57.6% (NR)	NR
**Intermountain (1 study)**	NR	VTE	Cobben, 2019[31]	26.4% (NR)	90.2% (NR)
**Kucher (2 studies)**	Risk Score ≥4	VTE	Vincentelli, 2018[66]	25.1% (17.0, 55.1)	92.9% (81.0, 95.4)
	NR	VTE	Cobben, 2019[31]	28.0% (NR)	85.7% (NR)
**Lecumberri (1 study)**	NR	VTE	Cobben, 2019[31]	61.6% (NR)	46.3% (NR)
**NAVAL (1 study)**	NR	VTE	Cobben, 2019[31]	19.0% (NR)	92.7% (NR)
**NICE Guidelines (1 study)**	NR	VTE	Cobben, 2019[31]	77.6% (NR)	39.0% (NR)
**Padua (10 studies)**	Risk score ≥4	VTE	Barbar, 2010[24]	94.6% (NR)	62.0% (NR)
	Risk Score ≥4	VTE	Blondon, 2018[28]; Nendaz, 2014[51]	All patients: 73.3% (54.1, 87.7)	All patients: 51.9% (49.3, 54.5)
				No prophylaxis: 62% (NR)	No prophylaxis: NR
	Risk Score ≥4	VTE	Lui, 2016[45]	23.4% (NR)	85.6% (NR)
	Risk Score ≥4	VTE	Moumneh, 2020[49]	91.6% (87.6, 94.7)	25.6% (24.9, 26.3)
	Risk Score ≥4	VTE	Zhou 2014[71]	30.1% (NR)	12.7% (NR)
	Risk Score ≥4	VTE	Zhou, 2018[70]	49.1% (NR)	16.2% (NR)
	Risk Score ≥4	VTE	Vincentelli, 2018[66]	52.4% (38.4, 81.9)	72.3% (63.9, 79.4)
	Risk Score ≥4	VTE	Wang, 2020[67]	76.2% (NR)	61.6% (NR)
	NR	VTE	Blondon, 2019b (abstract)[27]	72.7% (NR)	NR
	NR	VTE	Cobben, 2019[31]	61.8% (NR)	48.8% (NR)
**PRETEMED Guidelines (1 study)**	NR	VTE	Cobben, 2019[31]	81.6% (NR)	24.4% (NR)
**Shen 2020 (1 study)**	NR	VTE	Shen, 2020[61]	77.8% (NR)	84.7% (NR)
**Zakai 2013 (1 study)**	Model 2: NR	VTE	Cobben, 2019[31]	63.8% (NR)	31.7% (NR)
**SURGICAL INPATIENTS**					
**Caprini (8 studies)**	Risk Score >5	VTE	Hachey, 2016[38]	100% (100, 100)	7.2% (4.1, 11.0)
	Risk Score ≥5	VTE	Mlaver, 2020[48]	88.9% (NR)	32.7% (NR)
	Risk Score >5	VTE	Shaikh, 2016[59]	70.8% (48.9, 87.4)	39.39% (37.0, 41.9)
	Youden index >5.5	VTE	Tian, 2019[63]	76.0% (NR)	64.0% (NR)

For peer review only - http://bmjopen.bmj.com/site/about/guidelines.xhtml

	Risk score >6	VTE	Frankel, 2017 (abstract)[35]	61.5% (NR)	59.8% (NR)
	Risk Score >6	VTE	Shaikh, 2016[59]	58.3% (36.6, 77.9)	60.1% (57.6, 62.5)
	Risk Score >7	VTE	Hachey, 2016[38]	100% (100, 100)	31.4% (25, 37.3)
	Risk Score >9	VTE	Hachey, 2016[38]	83.3% (58.3, 100)	60.5% (54.4, 67.3)
	Risk Score >9	VTE	Shaikh, 2016[59]	16.7% (NR)	93.3% (NR)
	Risk Score >10	VTE	Hachey, 2016[38]	75.0% (50, 100)	69.6% (64.6, 76.4)
	Risk score >10	VTE	Dornbus, 2018 (abstract)[33]	78.9% (NR)	60.9% (NR)
	Risk Score >10.5	DVT or PE	Lobastov, 2016[46]	95.0% (NR)	73.0% (NR)
	Risk Score >15 [b]	VTE	Hewes, 2015[40]	100% (100, 100)	66.7% (55.0, 78.3)
**Khorana (1 study)**	Youden index >0.5	VTE	Tian, 2019[63]	78.0% (NR)	48.0% (NR)
**Padua (2 studies)**	Risk Score ≥4	VTE	Mlaver, 2020[48]	61.1% (NR)	47.4% (NR)
	Youden index >3.5	VTE	Tian, 2019[63]	36.0% (NR)	93.0% (NR)
**Rogers 2007 (1 study)**	Youden index >14.5	VTE	Tian, 2019[63]	53.0% (NR)	54.0% (NR)
**TRAUMA PATIENTS**					
**RAP (2 studies)**	Risk score ≥5	VTE	Tachino, 2019[62]	100% (86.8, 100)	37.9% (34.6, 41.3)
	Risk score 5 to ≤ 14	DVT or PE	Hegsted, 2013[39]	•DVT: 82.0% (77, 87)<br>•PE: 71.0% (55, 86)	•DVT: 57.0% (55, 59)<br>•PE: 53.0% (51, 56)
	Risk score >14	DVT or PE	Hegsted, 2013[39]	•DVT: 15.0% (11, 20)<br>•PE: 12.0% (1, 23)	•DVT: 97.0% (97, 98)<br>•PE: 96.0% (95, 97)
**TESS (2 studies)**	Risk score ≥5	VTE	Rogers, 2012[55]	77.4% (NR)	75.6% (NR)
	Risk score <9	VTE	Ho, 2014[41]	•All VTE:<br>97.0% (91, 99)	•All VTE:<br>27.0% (22, 32)
	Risk score <9	VTE	Ho, 2014[41]	•Fatal and non-fatal PE: 97.0% (87, 99)	•Fatal and non-fatal PE: 24.0% (20, 29)
	Risk score <9	VTE	Ho, 2014[41]	•Fatal PE only:<br>100% (81, 100)	•Fatal PE only:<br>20.0% (13, 28)

[a] Paper states 'moderate and high risk'
[b] Modified Caprini model
CI, confidence interval; DVT, deep vein thrombosis; NR, not reported; PE, pulmonary embolism; RAP, Risk Assessment Profile; TESS, Trauma Embolic Scoring System VTE, venous thromboembolism

For peer review only

For peer review only - http://bmjopen.bmj.com/site/about/guidelines.xhtml

DISCUSSION

Summary of results

In this systematic review of 51 observational studies evaluating RAMs for predicting the risk of developing VTE in hospital inpatients, we found that VTE RAMs have generally weak predictive accuracy. The studies validating these models are heterogeneous and most have a high risk of bias. Lack of methodological clarity was common, leading to difficulty in assessing the applicability of the individual study results.

Interpretation of results

We were unable to undertake meta-analysis or statistical examination of the causes of the observed heterogeneity. Potential sources of heterogeneity include variation in study design, the study population, how RAMs are implemented, outcome definition and measurement, and the use of thromboprophylaxis. The latter point warrants further attention. Thromboprophylaxis was employed in about half (n=25) of the studies,[21 22 24 26 28 30 34 36-38 40 42 44 46 49-52 54 57-59 64 70 71] with the proportion receiving thromboprophylaxis ranging from 3.8%[42] to 100%.[46 50] It was not employed in three studies[32 61 63] and 23 studies[23 25 27 29 31 33 35 39 41 43 45 47 48 53 55 56 60 62 65-69] did not report on thromboprophylaxis use. The use of thromboprophylaxis may lead to underestimation of predictive accuracy if a given RAM were to predict VTE events that were subsequently prevented by thromboprophylaxis. Limited reporting of thromboprophylaxis use precludes further analysis of its impact upon the performance of the RAMs.

Comparison to the existing literature

The present review is the largest and most comprehensive systematic review in this field to date. It includes 18 recent studies[26-31 33 42 48-50 60-63 66 67 70] published since the completion of the previous systematic review.[10 12 13] These studies are consistent with the previous literature in that they report modest performance of the assessed RAMs, with limitations in methodology and reporting preventing further analysis. The conclusion of this review therefore concurs with previous systematic reviews: there is insufficient evidence to recommend one RAM over another.

For peer review only - http://bmjopen.bmj.com/site/about/guidelines.xhtml

Strengths and limitations

This systematic review has a number of strengths. The review was conducted with robust methodology in accordance with the PRISMA statement and the protocol was registered with the PROSPERO register. Clinical experts were involved throughout as checkers and to assess the validity and applicability of research during the review. We reported descriptive statistics to provide insight into the limited evidence base applicable to the subject matter, and the scientific concerns regarding validity of the data. However, there are a number of potential weaknesses. Decisions on study relevance, information gathering, and validity were un-blinded and could potentially have been influenced by pre-formed opinions. However, masking is resource intensive with uncertain benefits. The studies of risk prediction were a combination of prospective cohorts and retrospective health database registries. Both have significant limitations. Retrospective studies of health database registries may have large numbers but may be limited by poor data quality and failure to accurately ascertain outcomes. Prospective cohorts may have better quality data but with smaller numbers lack statistical power. The included studies demonstrated high levels of heterogeneity so we were unable to undertake any meta-analysis

Implications for policy, practice and future research

Guidelines from the American College of Chest Physicians (ACCP)[72 73] and the UK National Institute for Health and Care Excellence (NICE)[10] suggest using a validated RAM to guide the decision on whether to prescribe thromboprophylaxis. This review identifies all relevant RAMs and their validation studies. The reported results are insufficient to recommend one RAM over another. A RAM with weak predictive accuracy may still be better than no RAM at all but it is unclear whether RAMs predict VTE risk better than unstructured clinical assessment. Further research is clearly needed but routine use of thromboprophylaxis may present an insurmountable barrier to generating accurate and precise estimates of the prognostic accuracy of RAMs. The evidence that thromboprophylaxis is effective means that it is unethical to withhold thromboprophylaxis when a significant risk of VTE is identified. This inevitably reduces the number of VTE events in any study

For peer review only - http://bmjopen.bmj.com/site/about/guidelines.xhtml

and confounds the association between risk factors and VTE events. Further studies of RAM accuracy will add little to our review unless they can address this issue.

Alternative approaches therefore need to be considered. Decision-analytic modelling can use existing data to explore the trade-off between the benefits and harms of thromboprophylaxis and identify key uncertainties for future primary research. The data presented in our review show how well RAMs predict VTE but do not tell us the threshold score on the RAM at which thromboprophylaxis should be given to maximise prevention of VTE and minimise harm from bleeding. This may be a more important determinant of RAM effectiveness than predictive accuracy for VTE. Le *et al.*[74] suggest thromboprophylaxis is beneficial and cost-effective if a patient's VTE risk exceeds 1%. Further work to improve RAMs to help stratify the risk of VTE in different types of hospitalised patients could focus on using decision-analytic modelling to compare the effects, harms and costs of giving thromboprophylaxis to patients with varying risk of VTE. This would allow determination of the risk threshold at which thromboprophylaxis provides optimal overall benefit.

Findings from decision-analytic modelling would require validation through primary research. The limitations of undertaking accuracy studies in populations where thromboprophylaxis is routinely used mean that future research should focus on research that compares the effectiveness of different risk assessment approaches. Observational studies could draw upon variation in practice to compare outcomes between different risk assessment methods. Alternatively, a controlled trial could compare risk assessment methods in low risk patients where existing evidence (synthesised using decision-analytic modelling) suggests the benefits of thromboprophylaxis are uncertain.

CONCLUSIONS

We identified a number of validated RAMs for potential risk stratification of hospitalised inpatients. The available evidence is insufficient to recommend one over another.

For peer review only - http://bmjopen.bmj.com/site/about/guidelines.xhtml

Acknowledgements

The authors would like to thank all additional members of the core project group for NIHR HTA 127454 for input and commentary throughout the work. We are also indebted to Helen Shulver for assistance with logistics and administration.

Competing interest

None to declare

Source of funding

This study was funded by the United Kingdom National Institute for Health Research Health Technology Assessment Programme (project number 127454). The views expressed in this report are those of the authors and not necessarily those of the NIHR HTA Programme. Any errors are the responsibility of the authors. The funders had no role in the study design, in the collection, analysis and interpretation of data; in the writing of the manuscript; and in the decision to submit the manuscript for publication.

Data sharing statement

All data relevant to the study are included in the article or uploaded as supplementary information.

Author contributions

AP co-ordinated the study. SG, DH, AP, XG, MH, BH, KW were responsible for conception, design and obtaining funding for the study. MC developed the search strategy, undertook searches, and organised retrieval of papers. AP, KS, MT and SG were responsible for the acquisition, analysis and interpretation of data. SG, MT, DH, XG, MH, BH, KW helped interpret and provided a methodological, policy and clinical perspective on the data. AP, MT and SG were responsible for the drafting of this paper, although all authors provided comments on the drafts, read and approved the final version. AP is the guarantor for the paper.

For peer review only - http://bmjopen.bmj.com/site/about/guidelines.xhtml

Copyright licence statement

The project was funded by the United Kingdom National Institute for Health Research Health Technology Assessment Programme and as such is covered by Crown copyright. The Corresponding Author has the right to grant on behalf of all authors and does grant on behalf of all authors, **a non-exclusive license** on a worldwide basis to BMJ publishing (BMJ) and its Licensees to permit this article (if accepted) to be published by BMJ, as set out in our licence.

For peer review only

For peer review only - http://bmjopen.bmj.com/site/about/guidelines.xhtml

REFERENCES

1. Goldhaber SZ, Visani L, De Rosa M, et al. Acute ulmonary embolism: clinical outcomes in the International Cooperative Pulmonary Embolism Registry (ICOPER). *Lancet* 1999;353:1386–9.
2. Prandoni P, Lensing AW, Cogo A, et al. The longterm clinical course of acute deep venous thrombosis. *Ann Intern Med* 1996;125:1-7.
3. ISTH Steering Committee for World Thrombosis Day. Thrombosis: a major contributor to the global disease burden. *J Thromb Haemost* 2014;12
4. Jha AK, Larizgoitia I, Audera-Lopez C, et al. The global burden of unsafe medical care: analytic modelling of observational studies. *BMJ Qual Saf* 2013;22:809-15.
5. Alikhan R, Bedenis R, Cohen AT. Heparin for the prevention of venous thromboembolism in acutely ill medical patients (excluding stroke and myocardial infarction). *Cochrane Database Syst Rev* 2014;2014(5):Cd003747. doi: 10.1002/14651858.CD003747.pub4 [published Online First: 2014/05/09]
6. Dentali F, Douketis JD, Gianni M, et al. Meta-analysis: anticoagulant prophylaxis to prevent symptomatic venous thromboembolism in hospitalized medical patients. *Ann Intern Med* 2007;146(4)
7. Kahn SR, Diendéré G, Morrison DR, et al. Effectiveness of interventions for the implementation of thromboprophylaxis in hospitalised patients at risk of venous thromboembolism: an updated abridged Cochrane systematic review and meta-analysis of randomised controlled trials. *BMJ Open* 2019;9(5):e024444. doi: 10.1136/bmjopen-2018-024444 [published Online First: 2019/05/28]
8. Lloyd NS, Douketis JD, Moinuddin I, et al. Anticoagulant prophylaxis to prevent asymptomatic deep vein thrombosis in hospitalized medical patients: a systematic review and meta-analysis. *J Thromb Haemost* 2008;6(3):405-14. doi: 10.1111/j.1538-7836.2007.02847.x [published Online First: 2007/11/23]
9. Henke PK, Kahn SR, Pannucci CJ, et al. Call to Action to Prevent Venous Thromboembolism in Hospitalized Patients: A Policy Statement From the American Heart Association. *Circulation* 2020;141(24):e914-e31. doi: 10.1161/cir.0000000000000769 [published Online First: 2020/05/08]
10. NICE. Venous Thromboembolism in Over 16s: Reducing the Risk of Hospital-Acquired Deep Vein Thrombosis or Pulmonary Embolism. NICE guideline [NG89]: National Institute for Health and Care Excellence, London, UK. https://www.nice.org.uk/guidance/NG89, 2018.
11. Chan NC, Gross PL, Weitz JI. Addressing the burden of hospital-related venous thromboembolism: the role of extended anticoagulant prophylaxis. *J Thromb Haemost* 2018;16(3):413-17. doi: 10.1111/jth.13942 [published Online First: 2018/02/27]

For peer review only - http://bmjopen.bmj.com/site/about/guidelines.xhtml

12. Huang W, Anderson FA, Spencer FA, et al. Risk assessment models for predicting venous thromboembolism among hospitalized non-surgical patients: a systematic review. *J Thromb Thrombolysis* 2013;35(1):67-80. doi: 10.1007/s11239-012-0780-0 [published Online First: 2012/07/25]

13. Stuck AK, Spirk D, Schaudt J, et al. Risk assessment models for venous thromboembolism in acutely ill medical patients. A systematic review. *Thrombosis and Haemostasis* 2017;117(4):801-08. doi: https://dx.doi.org/10.1160/TH16-08-0631

14. Moher D, Liberati A, Tetzlaff J, et al. Preferred reporting items for systematic reviews and meta-analyses: the PRISMA statement. *Ann Intern Med* 2009;151(4):264-9, w64. doi: 10.7326/0003-4819-151-4-200908180-00135 [published Online First: 2009/07/23]

15. Goodacre S, Hogg K, Griffin X, et al. The cost-effectiveness of venous thromboembolism risk assessment tools for hospital inpatients. UK: National Institute of Health Research, 2019.

16. Moons KGM, Wolff RF, Riley RD, et al. PROBAST: A Tool to Assess Risk of Bias and Applicability of Prediction Model Studies: Explanation and Elaboration. *Ann Intern Med* 2019;170(1):W1-w33. doi: 10.7326/m18-1377 [published Online First: 2019/01/01]

17. Wolff RF, Moons KGM, Riley RD, et al. PROBAST: A Tool to Assess the Risk of Bias and Applicability of Prediction Model Studies. *Ann Intern Med* 2019;170(1):51-58. doi: 10.7326/m18-1376 [published Online First: 2019/01/01]

18. Centre for Reviews and Dissemination. Systematic Reviews: CRD's Guidance for Undertaking Reviews in Health Care: York: Centre for Reviews and Dissemination, 2009.

19. McKenzie JE, Brennan SE, Ryan RE, et al. Chapter 9: Summarizing study characteristics and preparing for synthesis. Cochrane Handbook for Systematic Reviews of Interventions version 6.2 (updated February 2021) [Internet]: Cochrane, 2021.

20. Hosmer DW, Lemeshow S. Applied Logistic Regression (2nd Edition): John Wiley & Sons. New York, 2000.

21. Abdel-Razeq HN, Hijjawi SB, Jallad SG, et al. Venous thromboembolism risk stratification in medically-ill hospitalized cancer patients. A comprehensive cancer center experience. *Journal of Thrombosis and Thrombolysis* 2010;30(3):286-93. doi: 10.1007/s11239-010-0445-9

22. Autar R. The management of deep vein thrombosis: the autar DVT risk assessment scale re-visited. *Journal of Orthopaedic Nursing* 2003;7:114-24.

23. Bahl V, Hu HM, Henke PK, et al. A validation study of a retrospective venous thromboembolism risk scoring method. *Ann Surg* 2010;251(2):344-50. doi: 10.1097/SLA.0b013e3181b7fca6 [published Online First: 2009/09/26]

24. Barbar S, Noventa F, Rossetto V, et al. A risk assessment model for the identification of hospitalized medical patients at risk for venous thromboembolism: the Padua Prediction Score. *Journal of Thrombosis and Haemostasis* 2010;8(11):2450-57. doi: 10.1111/j.1538-7836.2010.04044.x

For peer review only - http://bmjopen.bmj.com/site/about/guidelines.xhtml

25. Bilimoria KY, Liu Y, Paruch JL, et al. Development and Evaluation of the Universal ACS NSQIP Surgical Risk Calculator: A Decision Aid and Informed Consent Tool for Patients and Surgeons. *Journal of the American College of Surgeons* 2013;217(5):833-42.e3. doi: https://doi.org/10.1016/j.jamcollsurg.2013.07.385

26. Blondon M, Righini M, Nendaz M, et al. External validation of the simplified Geneva risk assessment model for hospital-associated venous thromboembolism in the Padua cohort. *J Thromb Haemost* 2019:[Epub ahead of print]. doi: doi: 10.1111/jth.14688

27. Blondon M, Limacher A, Righini M, et al. Adequacy of hospital thromboprophylaxis and risk assessment models in the SWITCO65+ cohort. *Research and Practice in Thrombosis and Haemostasis* 2019;3(Supplement 1):760. doi: http://dx.doi.org/10.1002/rth2.12229

28. Blondon M, Spirk D, Kucher N, et al. Comparative Performance of Clinical Risk Assessment Models for Hospital-Acquired Venous Thromboembolism in Medical Patients. *Thrombosis and Haemostasis* 2018;118(1):82-89. doi: https://dx.doi.org/10.1160/TH17-06-0403

29. Bo H, Li Y, Liu G, et al. Assessing the Risk for Development of Deep Vein Thrombosis among Chinese Patients using the 2010 Caprini Risk Assessment Model: A Prospective Multicenter Study. *Journal of Atherosclerosis and Thrombosis* 2020;27(8):801-08. doi: https://dx.doi.org/10.5551/jat.51359

30. Chen X, Pan L, Deng H, et al. Risk Assessment in Chinese Hospitalized Patients Comparing the Padua and Caprini Scoring Algorithms. *Clinical and Applied Thrombosis/Hemostasis* 2018:1076029618797465. doi: https://dx.doi.org/10.1177/1076029618797465

31. Cobben MRR, Nemeth B, Lijfering WM, et al. Validation of risk assessment models for venous thrombosis in hospitalized medical patients. *Research and Practice in Thrombosis and Haemostasis* 2019;3(2):217-25. doi: http://dx.doi.org/10.1002/rth2.12181

32. de Bastos M, Barreto SM, Caiafa JS, et al. Derivation of a risk assessment model for hospital-acquired venous thrombosis: the NAVAL score. *Journal of Thrombosis and Thrombolysis* 2016;41(4):628-35. doi: http://dx.doi.org/10.1007/s11239-015-1277-4

33. Dornbos DL, Shah V, Priddy B, et al. Predicting venous thromboembolic complications following neurological surgery procedures. *Journal of Neurosurgery* 2018;128(4):55. doi: http://dx.doi.org/10.3171/2018.4.JNS.AANS2018abstracts

34. Elias P, Khanna R, Dudley A, et al. Automating Venous Thromboembolism Risk Calculation Using Electronic Health Record Data upon Hospital Admission: The Automated Padua Prediction Score. *Journal of Hospital Medicine* 2017;12(4):231-37. doi: https://dx.doi.org/10.12788/jhm.2714

35. Frankel JK, Belanger M, Tortora J, et al. Caprini score predicts venous thromboembolic events in patients undergoing robotic assisted prostatectomy. *Journal of Urology* 2017;197(4 Supplement 1):e635.

For peer review only - http://bmjopen.bmj.com/site/about/guidelines.xhtml

36. Grant PJ, Greene MT, Chopra V, et al. Assessing the Caprini Score for Risk Assessment of Venous Thromboembolism in Hospitalized Medical Patients Results presented at: The Society of Hospital Medicine Annual Meeting, March 26, 2014, Las Vegas, Nevada. *American Journal of Medicine* 2016;129(5):528-35. doi: http://dx.doi.org/10.1016/j.amjmed.2015.10.027
37. Greene MT, Spyropoulos AC, Chopra V, et al. Validation of Risk Assessment Models of Venous Thromboembolism in Hospitalized Medical Patients. *American Journal of Medicine* 2016;129(9):1001. doi: http://dx.doi.org/10.1016/j.amjmed.2016.03.031
38. Hachey KJ, Hewes PD, Porter LP, et al. Caprini venous thromboembolism risk assessment permits selection for postdischarge prophylactic anticoagulation in patients with resectable lung cancer. *The Journal of Thoracic and Cardiovascular Surgery* 2016;151(1):37-44.e1. doi: https://doi.org/10.1016/j.jtcvs.2015.08.039
39. Hegsted D, Gritsiouk Y, Schlesinger P, et al. Utility of the risk assessment profile for risk stratification of venous thrombotic events for trauma patients. *The American Journal of Surgery* 2013;205(5):517-20. doi: https://doi.org/10.1016/j.amjsurg.2013.01.022
40. Hewes PD, Hachey KJ, Zhang XW, et al. Evaluation of the Caprini Model for Venothromboembolism in Esophagectomy Patients. *The Annals of Thoracic Surgery* 2015;100(6):2072-78. doi: https://doi.org/10.1016/j.athoracsur.2015.05.098
41. Ho KM, Rao S, Rittenhouse KJ, et al. Use of the Trauma Embolic Scoring System (TESS) to predict symptomatic deep vein thrombosis and fatal and non-fatal pulmonary embolism in severely injured patients. *Anaesth Intensive Care* 2014;42(6):709-14. doi: 10.1177/0310057x1404200605 [published Online First: 2014/10/25]
42. Hu Y, Li X, Zhou H, et al. Comparison between the Khorana prediction score and Caprini risk assessment models for assessing the risk of venous thromboembolism in hospitalized patients with cancer: a retrospective case control study. *Interactive Cardiovascular and Thoracic Surgery* 2020;31(4):454-60. doi: https://dx.doi.org/10.1093/icvts/ivaa137
43. Krasnow R, Preston M, Chung B, et al. Validation of venous thromboembolism risk assessment score in major urologic cancer surgery: A population based study. *Journal of Urology* 2017;197(4 Supplement 1):e1126.
44. Liu L-P, Zheng H-G, Wang DZ, et al. Risk Assessment of Deep-Vein Thrombosis After Acute Stroke: a Prospective Study Using Clinical Factors. *CNS Neuroscience & Therapeutics* 2014;20(5):403-10. doi: 10.1111/cns.12227
45. Liu X, Liu C, Chen X, et al. Comparison between Caprini and Padua risk assessment models for hospitalized medical patients at risk for venous thromboembolism: A retrospective study. *Interactive Cardiovascular and Thoracic Surgery* 2016;23(4):538-43. doi: http://dx.doi.org/10.1093/icvts/ivw158

For peer review only - http://bmjopen.bmj.com/site/about/guidelines.xhtml

46. Lobastov K, Barinov V, Schastlivtsev I, et al. Validation of the Caprini risk assessment model for venous thromboembolism in high-risk surgical patients in the background of standard prophylaxis. *Journal of Vascular Surgery: Venous and Lymphatic Disorders* 2016;4(2):153-60. doi: https://doi.org/10.1016/j.jvsv.2015.09.004

47. Mahan CE, Liu Y, Turpie AG, et al. External validation of a risk assessment model for venous thromboembolism in the hospitalised acutely-ill medical patient (VTE-VALOURR). *Thromb Haemost* 2014;112(4):692-9. doi: 10.1160/th14-03-0239 [published Online First: 2014/07/06]

48. Mlaver E, Lynde GC, Gallion C, et al. Development of a Novel Preoperative Venous Thromboembolism Risk Assessment Model. *The American Surgeon* 2020;86(9):1098-105. doi: https://dx.doi.org/10.1177/0003134820943556

49. Moumneh T, Riou J, Douillet D, et al. Validation of risk assessment models predicting venous thromboembolism in acutely ill medical inpatients: A cohort study. *Journal of Thrombosis and Haemostasis : JTH* 2020;18(6):1398-407. doi: https://dx.doi.org/10.1111/jth.14796

50. Nafee T, Gibson CM, Travis R, et al. Machine learning to predict venous thrombosis in acutely ill medical patients. *Research and Practice in Thrombosis and Haemostasis* 2020;4(2):230-37. doi: https://dx.doi.org/10.1002/rth2.12292

51. Nendaz M, Spirk D, Kucher N, et al. Multicentre validation of the Geneva Risk Score for hospitalised medical patients at risk of venous thromboembolism. Explicit ASsessment of Thromboembolic RIsk and Prophylaxis for Medical PATients in SwitzErland (ESTIMATE). *Thromb Haemost* 2014;111(3):531-8. doi: 10.1160/th13-05-0427 [published Online First: 2013/11/15]

52. Pannucci CJ, Laird S, Dimick JB, et al. A Validated Risk Model to Predict 90-Day VTE Events in Postsurgical Patients. *Chest* 2014;145(3):567-73. doi: https://doi.org/10.1378/chest.13-1553

53. Pannucci CJ, Osborne NH, Wahl WL. Creation and validation of a simple venous thromboembolism risk scoring tool for thermally injured patients: analysis of the National Burn Repository. *J Burn Care Res* 2012;33(1):20-25. doi: 10.1097/BCR.0b013e318234d8b5

54. Patell R, Rybicki L, McCrae KR, et al. Predicting risk of venous thromboembolism in hospitalized cancer patients: Utility of a risk assessment tool. *American Journal of Hematology* 2017;92(6):501-07. doi: https://dx.doi.org/10.1002/ajh.24700

55. Rogers FB, Shackford SR, Horst MA, et al. Determining venous thromboembolic risk assessment for patients with trauma: the Trauma Embolic Scoring System. *J Trauma Acute Care Surg* 2012;73(2):511-5. [published Online First: 2012/09/29]

56. Rogers SO Jr, Kilaru RK, Hosokawa P, et al. Multivariable predictors of postoperative venous thromboembolic events after general and vascular surgery: results from the patient safety in surgery study. *J Am Coll Surg* 2007;204(6):1211-21.

57. Rosenberg D, Eichorn A, Alarcon M, et al. External validation of the risk assessment model of the International Medical Prevention Registry on Venous Thromboembolism (IMPROVE) for

For peer review only - http://bmjopen.bmj.com/site/about/guidelines.xhtml

medical patients in a tertiary health system. *Journal of the American Heart Association* 2014;3(6):e001152-e52. doi: 10.1161/JAHA.114.001152

58. Rothberg MB, Lindenauer PK, Lahti M, et al. Risk factor model to predict venous thromboembolism in hospitalized medical patients. *J Hosp Med* 2011;6(4):202-9. doi: 10.1002/jhm.888 [published Online First: 2011/04/12]

59. Shaikh M-A, Jeong HS, Mastro A, et al. Analysis of the American Society of Anesthesiologists Physical Status Classification System and Caprini Risk Assessment Model in Predicting Venous Thromboembolic Outcomes in Plastic Surgery Patients. *Aesthetic Surgery Journal* 2016;36(4):497-505. doi: 10.1093/asj/sjv198

60. Shang M-M, Yan R, Wang X-L, et al. Comparison of 2013 and 2009 versions of Caprini risk assessment models for predicting VTE in Chinese cancer patients: a retrospective study. *Journal of Thrombosis and Thrombolysis* 2020;50(2):446-51. doi: https://dx.doi.org/10.1007/s11239-020-02038-2

61. Shen C, Ge B, Liu X, et al. Predicting the occurrence of venous thromboembolism: construction and verification of risk warning model. *BMC Cardiovascular Disorders* 2020;20(1):249. doi: https://dx.doi.org/10.1186/s12872-020-01519-9

62. Tachino J, Yamamoto K, Shimizu K, et al. Quick risk assessment profile (qRAP) is a prediction model for post-traumatic venous thromboembolism. *Injury* 2019;50(9):1540-44. doi: https://dx.doi.org/10.1016/j.injury.2019.06.020

63. Tian B, Li H, Cui S, et al. A novel risk assessment model for venous thromboembolism after major thoracic surgery: a Chinese single-center study. *Journal of Thoracic Disease* 2019;11(5):1903-10. doi: https://dx.doi.org/10.21037/jtd.2019.05.11

64. Vardi M, Ghanem-Zoubi NO, Zidan R, et al. Venous thromboembolism and the utility of the Padua Prediction Score in patients with sepsis admitted to internal medicine departments. *Journal of Thrombosis and Haemostasis* 2013;11(3):467-73. doi: 10.1111/jth.12108

65. Vaziri S, Wilson J, Abbatematteo J, et al. Predictive performance of the American College of Surgeons universal risk calculator in neurosurgical patients. *Journal of Neurosurgery* 2018;128(3):942-47. doi: https://dx.doi.org/10.3171/2016.11.JNS161377

66. Vincentelli GM, Timpone S, Murdolo G, et al. A new risk assessment model for the stratification of the thromboembolism risk in medical patients: the TEVere Score. *Minerva Medica* 2018;109(6):436-42. doi: https://dx.doi.org/10.23736/S0026-4806.18.05689-6

67. Wang X, Yang Y-Q, Liu S-H, et al. Comparing different venous thromboembolism risk assessment machine learning models in Chinese patients. *Journal of Evaluation in Clinical Practice* 2020;26(1):26-34. doi: https://dx.doi.org/10.1111/jep.13324

68. Winoker JS, Paulucci DJ, Anastos H, et al. Predicting Complications Following Robot-Assisted Partial Nephrectomy with the ACS NSQIP Universal Surgical Risk Calculator. *The Journal of Urology* 2017;198(4):803-09. doi: https://dx.doi.org/10.1016/j.juro.2017.04.021

For peer review only - http://bmjopen.bmj.com/site/about/guidelines.xhtml

69. Woller SC, Stevens SM, Jones JP, et al. Derivation and Validation of a Simple Model to Identify Venous Thromboembolism Risk in Medical Patients. *The American Journal of Medicine* 2011;124(10):947-54.e2. doi: https://doi.org/10.1016/j.amjmed.2011.06.004

70. Zhou H, Hu Y, Li X, et al. Assessment of the Risk of Venous Thromboembolism in Medical Inpatients using the Padua Prediction Score and Caprini Risk Assessment Model. *Journal of Atherosclerosis and Thrombosis* 2018;25(11):1091-104. doi: https://dx.doi.org/10.5551/jat.43653

71. Zhou H, Wang L, Wu X, et al. Validation of a Venous Thromboembolism Risk Assessment Model in Hospitalized Chinese Patients: A Case-Control Study. *Journal of Atherosclerosis and Thrombosis* 2014;21(3):261-72. doi: 10.5551/jat.20891

72. Kahn SR, Lim W, Dunn AS, et al. Prevention of VTE in nonsurgical patients: Antithrombotic Therapy and Prevention of Thrombosis, 9th ed: American College of Chest Physicians Evidence-Based Clinical Practice Guidelines. *Chest* 2012;141:e195S-e226S.

73. Gould MK, Garcia DA, Wren SM, et al. Prevention of VTE in nonorthopedic surgical patients: Antithrombotic Therapy and Prevention of Thrombosis, 9th ed: American College of Chest Physicians Evidence-Based Clinical Practice Guidelines. *Chest* 2012;141:e227S-e77S.

74. Le P, Martinez KA, Pappas MA, et al. A decision model to estimate a risk threshold for venous thromboembolism prophylaxis in hospitalized medical patients. *J Thromb Haemost* 2017;15(6):1132-41.

For peer review only

For peer review only - http://bmjopen.bmj.com/site/about/guidelines.xhtml

Figure legends

**Figure 1**	**Study flow chart**
**Figure 2**	**PROBAST assessment summary graph - Review authors' judgements**
**Figure 3**	**C-statistics by model for studies involving a) medical b) surgical and c) trauma inpatients**

For peer review only

For peer review only - http://bmjopen.bmj.com/site/about/guidelines.xhtml

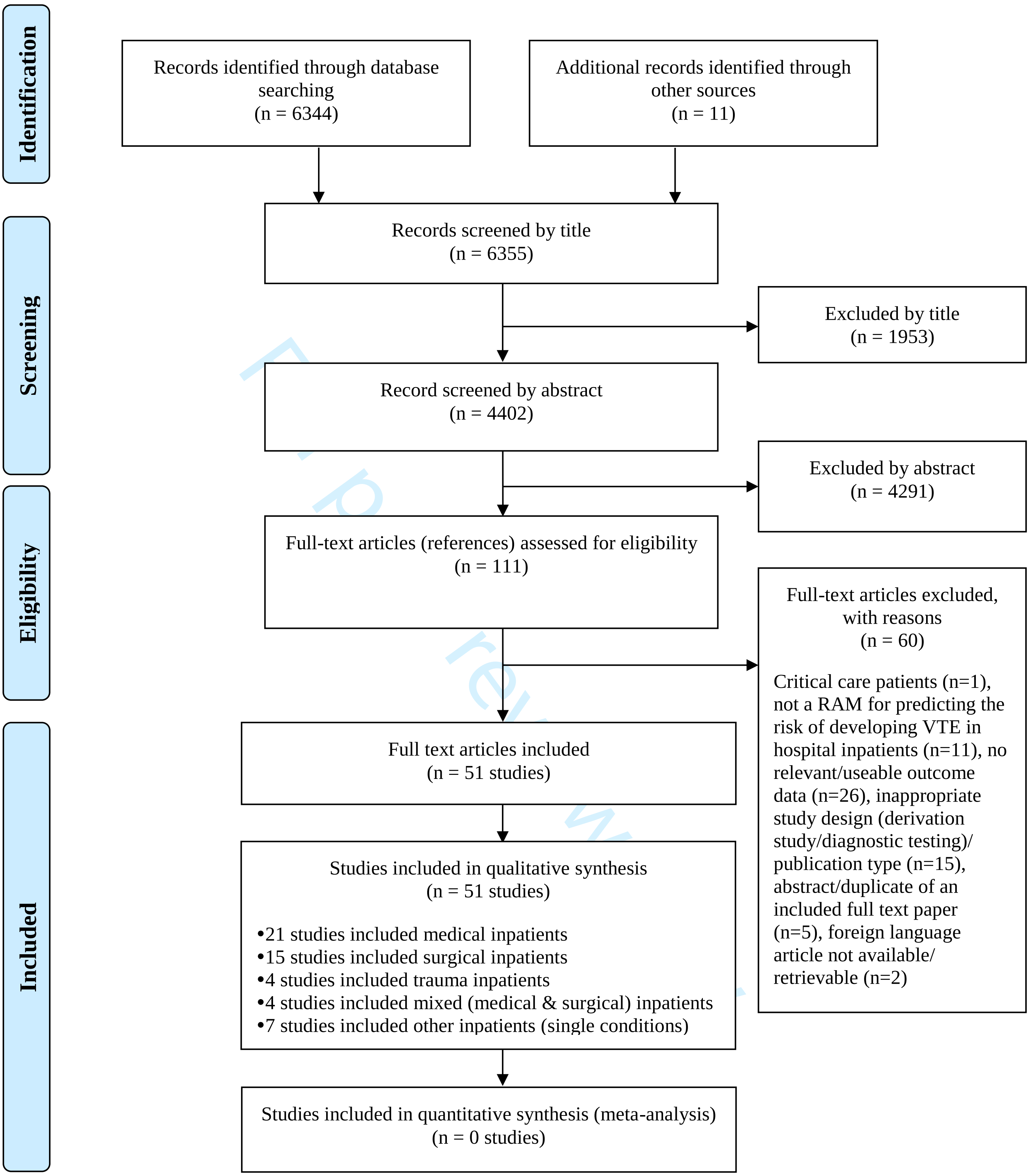

Figure 1

For peer review only - http://bmjopen.bmj.com/site/about/guidelines.xhtml

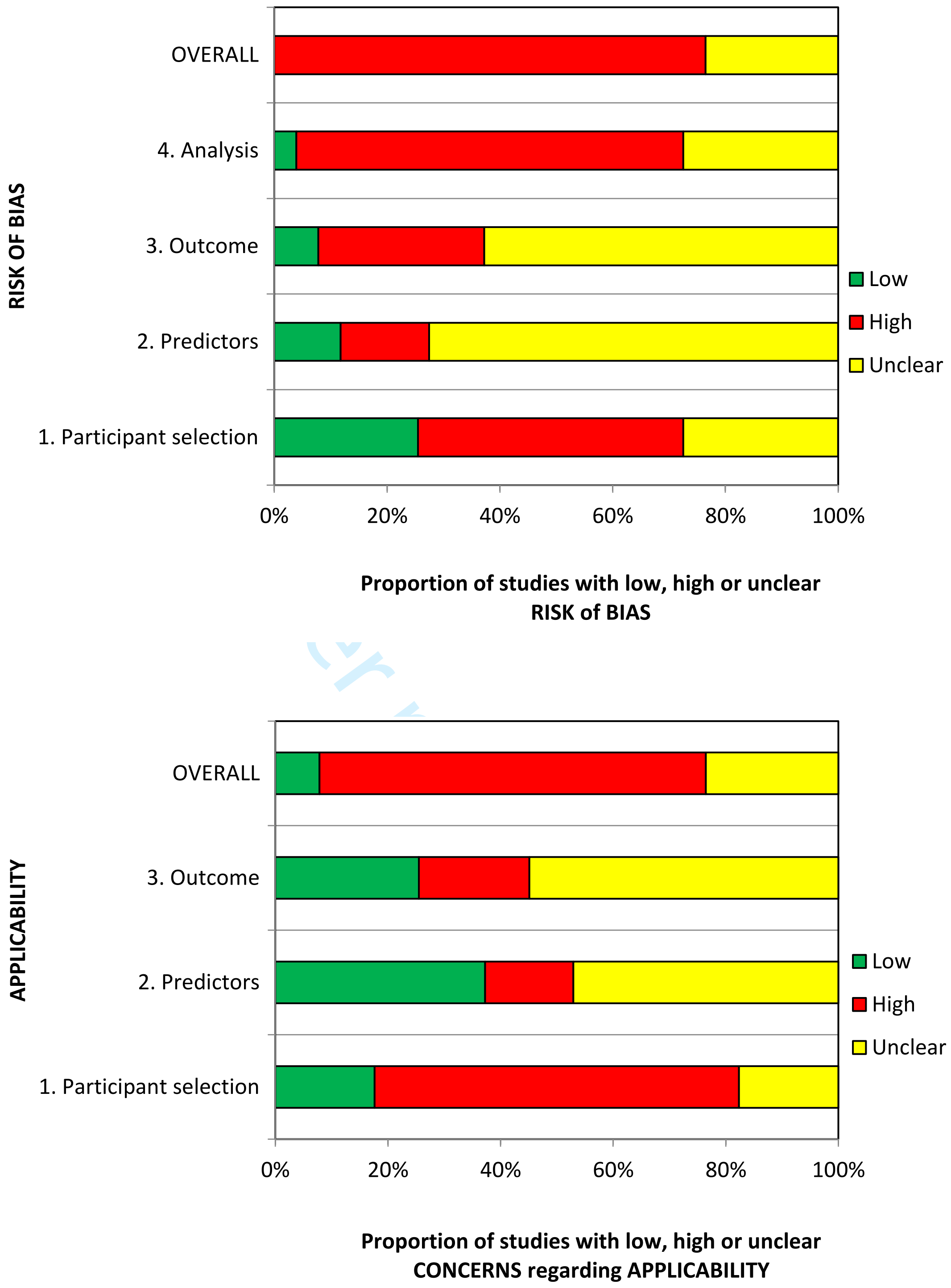
RISK OF BIAS
OVERALL
4. Analysis
3. Outcome
2. Predictors
1. Participant selection
Low
High
Unclear
0%
20%
40%
60%
80%
100%
Proportion of studies with low, high or unclear RISK of BIAS
APPLICABILITY
OVERALL
3. Outcome
2. Predictors
1. Participant selection
Low
High
Unclear
0%
20%
40%
60%
80%
100%
Proportion of studies with low, high or unclear CONCERNS regarding APPLICABILITY

Figure 2

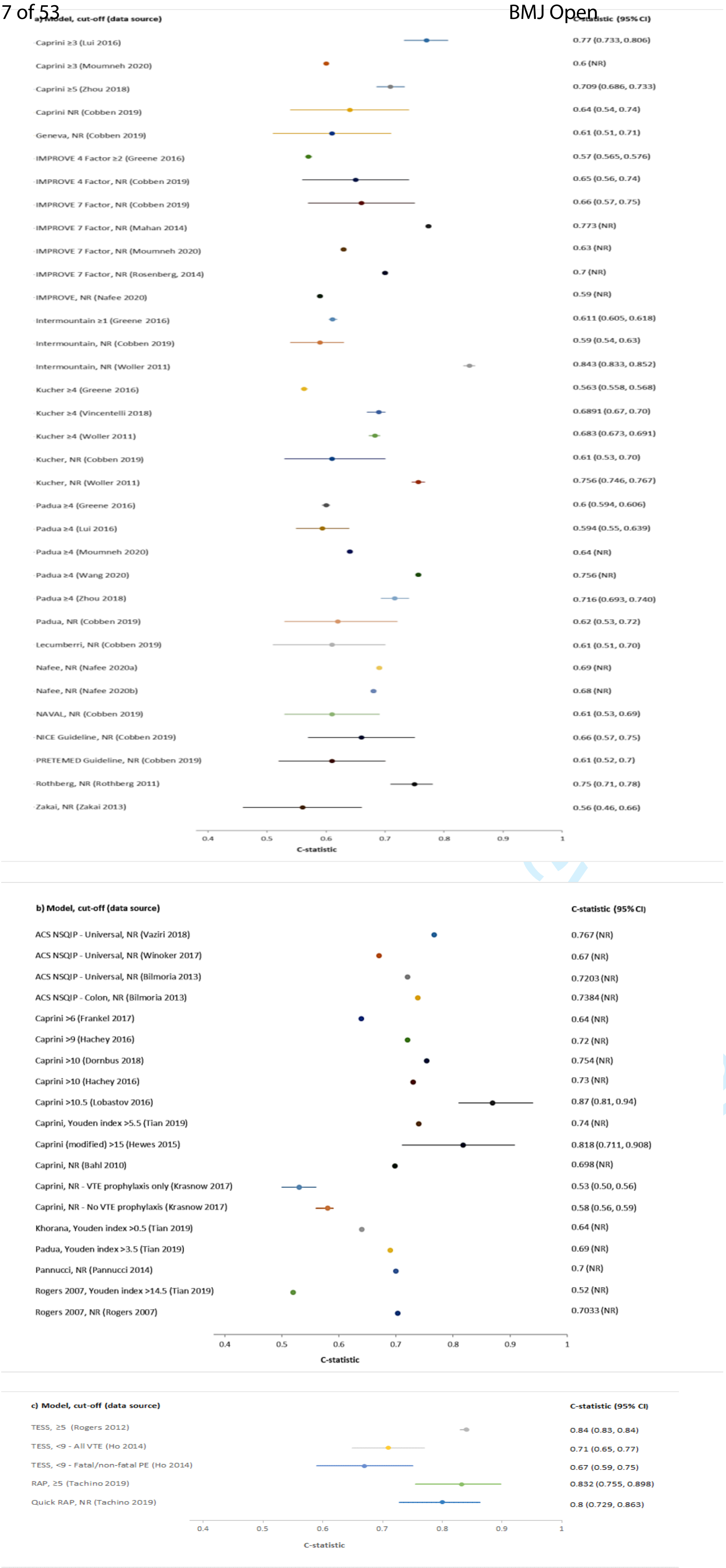

ACS NSQIP, American College of Surgeons National Surgical Quality Improvement Program; CI, confidence interval; DVT, deep vein thrombosis; NR, not reported; PE, pulmonary embolism; RAP, Risk Assessment Profile; TESS, Trauma Embolic Scoring System VTE, venous thromboembolism

Figure 3

ONLINE SUPPLEMENTARY

APPENDIX S1: LITERATURE SEARCH STRATEGY

Table S1 Literature search strategy for the review of RAMs for VTE in hospital inpatients

**Database searched:**	**Ovid MEDLINE(R) Epub Ahead of Print, In-Process & Other Non-Indexed Citations, Ovid MEDLINE(R) Daily, Ovid MEDLINE and Versions(R)**
**Platform or provider used:**	**Ovid SP**
**Date of coverage:**	**1946 to February 2021**
**Search undertaken:**	**February 2021**

1 pulmonary embolism/ or thromboembolism/ or venous thromboembolism/ or venous thrombosis/ or upper extremity deep vein thrombosis/
2 (((venous or vein) adj (thrombosis or thromboses or thrombus or thromboembolism)) or (dvt or vte) or ((pulmonary or lung) adj3 (embolism or emboli or embolus or emboliz* or thromboembolism))).ti,ab.
3 1 or 2
4 letter/ or editorial/ or news/ or exp historical article/ or anecdotes as topic/ or comment/ or case report/ or (letter or comment).ti.
5 randomized controlled trial/ or random*.ti,ab.
6 4 not 5
7 animals/ not humans/
8 exp animals, laboratory/
9 exp animal experimentation/
10 exp models, animal/
11 exp rodentia/
12 (rat or rats or mouse or mice).ti.
13 6 or 7 or 8 or 9 or 10 or 11 or 12
14 3 not 13
15 (risk* adj2 assess*).ti,ab.
16 ((score* or scoring) adj2 (tool* or system*)).ti,ab.
17 ((risk* or predict* or prognos*) adj4 (tool* or rule* or index* or indices or score* or scoring or scale* or model* or system* or algorithm* or stratif* or criteria or calculat*)).ti,ab.
18 (vienna adj5 cats).ti,ab.
19 (vienna cancer and thrombosis study).ti,ab.
20 trauma embolic scoring.ti,ab.
21 tess.ti,ab.
22 (roger* or caprini* or kucher* or cohen* or padua* or khorana* or autar).ti,ab.
23 (well* adj2 (score* or scoring)).ti,ab.
24 department of health.ti,ab,au.
25 or/15-24
26 14 and 25
27 limit 26 to yr="2017 -Current"

For peer review only

Databases searched: **EMBASE**

Platform or provider used: **Ovid SP**

Date of coverage: **1974 to February 2021**

Search undertaken: **February 2021**

1 thromboembolism/ or venous thromboembolism/ or vein thrombosis/ or deep veinthrombosis/ or leg thrombosis/ or lower extremity deep vein thrombosis/ or postoperativethrombosis/ or lung embolism/ or upper extremity deep vein thrombosis/
2 (((venous or vein) adj (thrombosis or thromboses or thrombus or thromboembolism)) or (dvt or vte) or ((pulmonary or lung) adj3 (embolism or emboli or embolus or emboliz* or thromboembolism))).ti,ab.
3 1 or 2
4 letter.pt. or letter/ or note.pt. or editorial.pt. or case report/ or case study/ or (letter or comment*).ti.
5 randomized controlled trial/ or random*.ti,ab.
6 4 not 5
7 animal/ not human/
8 nonhuman/
9 exp animal experiment/ or exp experimental animal/
10 animal model/
11 exp rodent/
12 (rat or rats or mouse or mice).ti.
13 6 or 7 or 8 or 9 or 10 or 11 or 12
14 3 not 13
15 (risk* adj2 assess*).ti,ab.
16 ((score* or scoring) adj2 (tool* or system*)).ti,ab.
17 ((risk* or predict* or prognos*) adj4 (tool* or rule* or index* or indices or score* or scoring or scale* or model* or system* or algorithm* or stratif* or criteria or calculat*)).ti,ab.
18 (vienna adj5 cats).ti,ab.
19 (vienna cancer and thrombosis study).ti,ab.
20 trauma embolic scoring.ti,ab.
21 tess.ti,ab.
22 (roger* or caprini* or kucher* or cohen* or padua* or khorana* or autar).ti,ab.
23 (well* adj2 (score* or scoring)).ti,ab.
24 department of health.ti,ab,au.
25 or/15-24
26 14 and 25
27 limit 26 to yr="2017 -Current"

For peer review only

**Databases searched:**	**Cochrane CENTRAL Register of Randomised Controlled Trials & Cochrane Database of Systematic Reviews**
**Platform or provider used:**	**www.thecochranelibrary.com**
**Date of coverage:**	**Inception to February 2021**
**Search undertaken:**	**February 2021**

#1. MeSH descriptor: [venous thromboembolism] this term only

#2. MeSH descriptor: [pulmonary embolism] this term only

#3. MeSH descriptor: [venous thrombosis] this term only

#4. MeSH descriptor: [thromboembolism] this term only

#5. MeSH descriptor: [upper extremity deep vein thrombosis] this term only

#6. ((*venous or *vein) next (thrombosis or thromboses or thrombus or thromboembolism) or dvt or vte or (pulmonary or lung) near/3 (embolism or emboli or embolus or emboliz* or thromboembolism)):ti,ab

#7. #1 or #2 or #3 or #4 or #6

#8. (risk* near/2 assess*):ti,ab

#9. ((score* or scoring) near/2 (tool* or system*)):ti,ab

#10. ((risk* or predict* or prognos*) near/4 (tool* or rule* or index* or indices or score* or scoring or scale* or model* or system* or algorithm* or stratif* or criteria or calculat*)):ti,ab

#11. (vienna near/5 cats):ti,ab

#12. (vienna cancer and thrombosis study):ti,ab

#13. trauma embolic scoring:ti,ab

#14. tess:ti,ab

#15. (roger* or caprini* or kucher* or cohen* or padua* or khorana* or autar):ti,ab

#16. (well* near/2 (score* or scoring)):ti,ab

#17. (department of health):ti,ab

#18. (or #8-#17)

#19. #7 and #18 with Publication Year from 2017 to 21

For peer review only

APPENDIX S2: LIST OF EXCLUDED STUDIES WITH RATIONALE

	Authors, year	Reason for exclusion
1.	Alikhan et al., 2004 [1]	Derivation only
2.	Alper et al., 2018 [2]	Not a RAM for predicting the risk of developing VTE in hospital inpatients
3.	Arcelus et al., 1991 [3]	No relevant/useable outcome data
4.	Arpaia et al., 2020 [4]	Not a RAM for predicting the risk of developing VTE in hospital inpatients
5.	Bagot et al., 2010 [5]	No relevant/useable outcome data
6.	Bellizzi et al., 2018 [6]	No relevant/useable outcome data
7.	Blondon et al., 2020 [7]	Duplicate of included study (Blondon et al., 2019a)
8.	Blondon et al., 2017 [8]	Abstract of an included full-text study
9.	Caprini et al., 2001 [9]	Review
10.	Chen et al., 2018 [10]	Not available (foreign language)
11.	Chopard et al., 2006 [11]	No relevant/useable outcome data
12.	Coelho et al., 2020 [12]	Not a RAM for predicting the risk of developing VTE in hospital inpatients
13.	Dang et al., 2019 [13]	Derivation only
14.	Davis and Intagliata, 2018 [14]	No relevant/useable outcome data
15.	Depietri et al., 2018 [15]	No relevant/useable outcome data
16.	Ellis et al., 2019 [16]	No relevant/useable outcome data
17.	Fadoi Foundation, 2020 [17]	Trial protocol (no results)
18.	Ferreira et al., 2018 [18]	No relevant/useable outcome data
19.	Ferreira et al., 2017 [19]	No relevant/useable outcome data
20.	Fritz et al., 2021 [20]	No relevant/useable outcome data
21.	Gibson et al., 2017 [21]	RAM involves diagnostic testing
22.	Girardi et al., 2018 [22]	Not a RAM for predicting the risk of developing VTE in hospital inpatients
23.	Grzelak et al., 2019 [23]	Not a RAM for predicting the risk of developing VTE in hospital inpatients
24.	Hostler et al., 2016 [24]	No relevant/useable outcome data
25.	Hu, 2018 [25]	No relevant/useable outcome data
26.	Koren et al., 2017 [26]	Not a RAM for predicting the risk of developing VTE in hospital inpatients
27.	Kucher et al., 2005 [27]	No relevant/useable outcome data
28.	Lecumberri et al., 2008 [28]	No relevant/useable outcome data
29.	Luo and Zhang, 2017 [29]	Not available (foreign language)
30.	Maynard et al., 2010 [30]	No relevant/useable outcome data
31.	McCaffrey et al., 2007 [31]	No relevant/useable outcome data

For peer review only

32.	Meizoso et al., 2017 [32]	Derivation only
33.	Monti et al., 2019 [33]	Abstract of included full text paper
34.	Mull et al., 2017 [34]	Review
35.	Nafee et al., 2018 [35]	Abstract of included full text paper
36.	Nnadi et al., 2017 [36]	No relevant/useable outcome data
37.	Obi et al., 2015 [37]	Critical care patients
38.	Pannucci et al., 2017 [38]	Review
39.	Rafizadeh et al., 2016 [39]	No relevant/useable outcome data
40.	Rastogi et al., 2020 [40]	No relevant/useable outcome data
41.	Razak et al., 2019 [41]	Not a RAM for predicting the risk of developing VTE in hospital inpatients
42.	Robert-Ebadi et al., 2017 [42]	Not a RAM for predicting the risk of developing VTE in hospital inpatients
43.	Salim et al., 2018 [43]	No relevant/useable outcome data
44.	Samama et al., 2006 [44]	Expert opinion
45.	Shrotriya et al., 2018 [45]	Not a RAM for predicting the risk of developing VTE in hospital inpatients
46.	Smilg Nicolas et al., 2018 [46]	No relevant/useable outcome data
47.	Spirk et al., 2017 [47]	No relevant/useable outcome data
48.	Spyropoulos et al., 2020 [48]	RAM involves diagnostic testing
49.	Spyropoulos et al., 2011 [49]	Derivation only
50.	Stuck et al., 2017 [50]	Review
51.	Tadesse et al., 2020 [51]	No relevant/useable outcome data
52.	Taha et al., 2020 [52]	No relevant/useable outcome data
53.	Tung et al., 2020 [53]	No relevant/useable outcome data
54.	Veith et al., 2019 [54]	No relevant/useable outcome data
55.	Winoker et al., 2017 [55]	Abstract of an included full-text study
56.	Yale et al., 2005 [56]	Derivation only
57.	Ye et al., 2017 [57]	Review
58.	Zakai et al., 2013 [58]	Derivation only
59.	Zambelli et al., 2020 [59]	Not a RAM for predicting the risk of developing VTE in hospital inpatients
60.	Zhou et al., 2012 [60]	Not a RAM for predicting the risk of developing VTE in hospital inpatients

For peer review only

REFERENCES (APPENDIX S2)

1. Alikhan R, Cohen AT, Combe S, et al. Risk Factors for Venous Thromboembolism in Hospitalized Patients With Acute Medical Illness: Analysis of the MEDENOX Study. *JAMA Internal Medicine* 2004;164(9):963-68. doi: 10.1001/archinte.164.9.963

2. Alper EC, Ip IK, Balthazar P, et al. Risk Stratification Model: Lower-Extremity Ultrasonography for Hospitalized Patients with Suspected Deep Vein Thrombosis. *Journal of General Internal Medicine* 2018;33(1):21-25. doi: https://dx.doi.org/10.1007/s11606-017-4170-3

3. Arcelus JI, Candocia S, Traverso CI, et al. Venous thromboembolism prophylaxis and risk assessment in medical patients. *Semin Thromb Hemost* 1991;17:3313-8.

4. Arpaia GG, Caleffi A, Marano G, et al. Padua prediction score and IMPROVE score do predict in-hospital mortality in Internal Medicine patients. *Internal and Emergency Medicine* 2020;15(6):997-1003. doi: https://dx.doi.org/10.1007/s11739-019-02264-4

5. Bagot C, Gohil S, Perrott R, et al. The use of an exclusion-based risk-assessment model for venous thrombosis improves uptake of appropriate thromboprophylaxis in hospitalized medical patients. *QJM* 2010;103:597-605.

6. Bellizzi A, Palombi G, Bardi G, et al. Score Padua and IMPROVE: What changes in management of venous thromboembolism prophylaxis in Internal Medicine? *Italian Journal of Medicine* 2018;12(2 Supplement 1):2. doi: http://dx.doi.org/10.4081/itjm.2018.s2

7. Blondon M, Righini M, Nendaz M, et al. External validation of the simplified Geneva risk assessment model for hospital-associated venous thromboembolism in the Padua cohort. *Journal of Thrombosis and Haemostasis* 2020;18(3):676-80. doi: https://dx.doi.org/10.1111/jth.14688

8. Blondon M, Spirk D, Kucher N, et al. External validation and comparison of the improve risk assessment model with the Geneva risk assessment model in the estimate cohort. *Research and Practice in Thrombosis and Haemostasis* 2017;1(Supplement 1):180. doi: http://dx.doi.org/10.1002/rth2.12012

9. Caprini JA, Arcelus JI, Reyna J. Effective risk stratification of surgical and nonsurgical patients for venous thromboembolic disease. *Seminars in Hematology* 2001;38:12-19. doi: https://doi.org/10.1016/S0037-1963(01)90094-0

10. Chen XL, Pan L, Wang Y. [Validity of Padua risk assessment scale for assessing the risk of deep venous thrombosis in hospitalized patients]. *Zhonghua nei ke za zhi* 2018;57(7):514-17. doi: https://dx.doi.org/10.3760/cma.j.issn.0578-1426.2018.07.009

11. Chopard P, Spirk D, Bounameaux H, et al. Identifying acutely ill medical patients requiring thromboprophylaxis. *J Thromb Haemost* 2006;4(4):915-6.

12. Coelho J, Divernet-Queriaud M, Roy P-M, et al. Comparison of the Wells score and the revised Geneva score as a tool to predict pulmonary embolism in outpatients over age 65. *Thrombosis Research* 2020;196:120-26. doi: https://dx.doi.org/10.1016/j.thromres.2020.07.026

13. Dang JT, Switzer N, Delisle M, et al. Predicting venous thromboembolism following laparoscopic bariatric surgery: development of the BariClot tool using the MBSAQIP database. *Surgical Endoscopy* 2019;33(3):821-31. doi: https://dx.doi.org/10.1007/s00464-018-6348-0

14. Davis J, Intagliata NM. Practice patterns in application of venous thromboembolism prophylaxis to a hepatology inpatient service: A quality improvement project. *Hepatology* 2018;68(Supplement 1):296A. doi: http://dx.doi.org/10.1002/hep.30257

15. Depietri L, Marietta M, Scarlini S, et al. Clinical impact of application of risk assessment models (Padua Prediction Score and Improve Bleeding Score) on venous thromboembolism, major hemorrhage and health expenditure associated with pharmacologic VTE prophylaxis: a "real life" prospective and retrospective observational study on patients hospitalized in a Single Internal Medicine Unit (the STIME study). *Internal and Emergency Medicine* 2018;13(4):527-34. doi: https://dx.doi.org/10.1007/s11739-018-1808-z

16. Ellis N, Grubb C-M, Mustoe S, et al. Venous thromboembolism risk in psychiatric in-patients: a multicentre cross-sectional study. *BJPsych Bulletin* 2019:1-5. doi: https://dx.doi.org/10.1192/bjb.2019.25

17. Fadoi Foundation. Efficacy of the Use of Risk Scores in Reducing Important Clinical Outcomes in Hospitalized Medical Ill Patients, 2020. Available *https://clinicaltrialsgov/show/NCT04267718* [Accessed 2 March 2021]

18. Ferreira C, Avelino Mancini R, Monique Lana Diniz M, et al. Evaluation of inter-rater agreement of a risk assessment model for thromboprophylaxis in medical hospitalised patients: Preliminary results of a cohort study. *Research and Practice in Thrombosis and Haemostasis* 2018;2(Supplement 1):323-24. doi: http://dx.doi.org/10.1002/rth2.12125

19. Ferreira D, de Sousa JA, Felicissimo P, et al. Venous thromboembolism risk and prophylaxis in the Portuguese hospital care setting: The ARTE study. *Revista portuguesa de cardiologia : orgao oficial da Sociedade Portuguesa de Cardiologia (Portuguese Journal of Cardiology)* 2017;36(11):823-30. doi: https://dx.doi.org/10.1016/j.repc.2017.01.009

20. Fritz MK, Kincaid SE, Sargent CG, et al. Venous thromboembolism (VTE) risk stratification in general medical patients at an academic medical center. *Journal of Thrombosis and Thrombolysis* 2021;51(1):67-73. doi: https://dx.doi.org/10.1007/s11239-020-02144-1

21. Gibson CM, Spyropoulos AC, Cohen AT, et al. The IMPROVEDD VTE Risk Score: Incorporation of D-Dimer into the IMPROVE Score to Improve Venous Thromboembolism Risk Stratification. *TH Open* 2017;1(1):e56-e65. doi: https://dx.doi.org/10.1055/s-0037-1603929

22. Girardi AM, Bettiol RS, Garcia TS, et al. Wells and Geneva Scores Are Not Reliable Predictors of Pulmonary Embolism in Critically Ill Patients: A Retrospective Study. *Journal of Intensive Care Medicine* 2018:885066618816280. doi: https://dx.doi.org/10.1177/0885066618816280

For peer review only

23. Grzelak M, Javia V, Cox C, et al. Incidence and patient characteristics for the development of venous thromboembolic events within the adult burn center population and an evaluation of the caprini risk assessment model. *Journal of Burn Care and Research* 2019;40(Supplement 1):S57. doi: http://dx.doi.org/10.1093/jbcr/irz013.088

24. Hostler DC, Marx ES, Moores LK, et al. Validation of the international medical prevention registry on venous thromboembolism bleeding risk score. *Chest* 2016;149(2):372-79. doi: http://dx.doi.org/10.1378/chest.14-2842

25. Hu Y. Comparison between caprini and Khorana risk assessment models for hospitalized medical patients with cancer at high risk for venous thromboembolism: a retrospective study. *American Journal of Respiratory and Critical Care Medicine* 2018;197(MeetingAbstracts)

26. Koren O, Hourri A, Elias M, et al. Failure to validate padua score's ability to predict the risk of venous thromboembolism in medical patients. *Clinical Therapeutics* 2017;39(8 Supplement 1):e59.

27. Kucher N, Koo S, Quiroz R, et al. Electronic alerts to prevent venous thromboembolism among hospitalized patients. *N Engl J Med* 2005;352(10):969-77.

28. Lecumberri R, Marqués M, Díaz-Navarlaz MT, et al. Maintained effectiveness of an electronic alert system to prevent venous thromboembolism among hospitalized patients. *Thromb Haemost* 2008;100(4):699-704.

29. Luo X, Zhang F. Validation of the Caprini risk assessment model for venous thromboembolism in Chinese hospitalized patients in a general hospital. *National Medical Journal of China* 2017;97(24):1875-77. doi: http://dx.doi.org/10.3760/cma.j.issn.0376-2491.2017.24.007

30. Maynard GA, Morris TA, Jenkins IH, et al. Optimizing prevention of hospital-acquired venous thromboembolism (VTE): prospective validation of a VTE risk assessment model. *J Hosp Med* 2010;5(1):10-18.

31. McCaffrey R, Bishop M, Adonis-Rizzo M, et al. Development and Testing of a DVT Risk Assessment Tool: Providing Evidence of Validity and Reliability. *Worldviews on Evidence-Based Nursing* 2007;4(1):14-20. doi: 10.1111/j.1741-6787.2007.00074.x

32. Meizoso JP, Karcutskie CAt, Ray JJ, et al. A simplified stratification system for venous thromboembolism risk in severely injured trauma patients. *The Journal of Surgical Research* 2017;207:138-44. doi: https://dx.doi.org/10.1016/j.jss.2016.08.072

33. Monti M, Timpone S, Murdolo G, et al. A new risk assessment model for the stratification of the thromboembolism risk in medical patients: The TEVere Score. *Italian Journal of Medicine* 2019;13(Supplement 2):7-8. doi: http://dx.doi.org/10.4081/itjm.2019.s2

34. Mull N, Hecht T, Umscheid C, et al. Clinical risk prediction scores for venous thromboembolism in hospitalized patients. *International Journal of Technology Assessment in Health Care* 2017;33(Supplement 1):75-76. doi: http://dx.doi.org/10.1017/S0266462317002094

For peer review only

35. Nafee T, Gibson CM, Travis R, et al. Performance of a machine learning model vs. IMPROVE score for VTE prediction in acute medically ill patients: Insights from the APEX trial. *European Heart Journal* 2018;39(Supplement 1):431. doi: http://dx.doi.org/10.1093/eurheartj/ehy565.2160

36. Nnadi C, Soltani SJ, Brice HG, et al. Evaluation of a new thrombo-embolism scoring system (TESS) for spinal surgery. *Spine Journal* 2017;17(3):S21-S22. doi: http://dx.doi.org/10.1016/j.spinee.2016.12.068

37. Obi AT, Pannucci CJ, Nackashi A, et al. Validation of the Caprini Venous Thromboembolism Risk Assessment Model in Critically Ill Surgical Patients. *JAMA Surgery* 2015;150(10):941-48. doi: 10.1001/jamasurg.2015.1841

38. Pannucci CJ, Swistun L, MacDonald JK, et al. Individualized Venous Thromboembolism Risk Stratification Using the 2005 Caprini Score to Identify the Benefits and Harms of Chemoprophylaxis in Surgical Patients: A Meta-analysis. *Annals of Surgery* 2017;265(6):1094-103. doi: https://dx.doi.org/10.1097/SLA.0000000000002126

39. Rafizadeh R, Turgeon RD, Batterink J, et al. Characterization of venous thromboembolism risk in medical inpatients using different clinical risk assessment models. *Canadian Journal of Hospital Pharmacy* 2016;69(6):454-59.

40. Rastogi R, Mehaffey JH, Turrentine BE, et al. Implementation of an Electronic Medical Record Risk Stratification Tool to Reduce Venous Thromboembolism Events. *Journal of the American College of Surgeons* 2020;231(4 Supplement 1):S141-S42. doi: http://dx.doi.org/10.1016/j.jamcollsurg.2020.07.273

41. Razak MKA, Rasheed JI, Hamad AA. A comparison between wells' score and modified doppler ultrasound in the diagnosis of deep venous thrombosis of the lower limb. *International Journal of Pharmaceutical Sciences and Research* 2019;10(5):2369-74. doi: http://dx.doi.org/10.13040/IJPSR.0975-8232.10%285%29.2369-74

42. Robert-Ebadi H, Mostaguir K, Hovens MM, et al. Assessing clinical probability of pulmonary embolism: prospective validation of the simplified Geneva score. *Journal of Thrombosis and Haemostasis* 2017;15(9):1764-69. doi: https://dx.doi.org/10.1111/jth.13770

43. Salim S, Onida S, Davies A. Variability in venous thromboembolism (VTE) risk assessment tools. *Phlebology* 2018;33(1 Supplement 1):36-37. doi: http://dx.doi.org/10.1177/0268355518817748

44. Samama MM, Dahl OE, Mismetti P, et al. An electronic tool for venous thromboembolism prevention in medical and surgical patients. *Haematologica* 2006;91(1):64.

45. Shrotriya S, Dhakal P, Sharma M, et al. Predictors of venous thromboembolism among cancer inpatients: A predictive model. *Journal of Clinical Oncology* 2018;36(15 Supplement 1) doi: http://dx.doi.org/10.1200/JCO.2018.36.15_suppl.e18824

For peer review only

46. Smilg Nicolas C, Tornel Sanchez G, Trujillo Santos J. Concordance among venous thromboembolism risk assessment models. *Concordancia entre las escalas de prediccion de riesgo de tromboembolia venosa* 2018;150(2):61-63. doi: https://dx.doi.org/10.1016/j.medcli.2017.06.021
47. Spirk D, Stuck AK, Hager A, et al. Electronic alert system for improving appropriate thromboprophylaxis in hospitalized medical patients: a randomized controlled trial. *Journal of Thrombosis and Haemostasis* 2017;15(11):2138-46. doi: https://dx.doi.org/10.1111/jth.13812
48. Spyropoulos AC, Lipardi C, Xu J, et al. Modified IMPROVE VTE Risk Score and Elevated D-Dimer Identify a High Venous Thromboembolism Risk in Acutely Ill Medical Population for Extended Thromboprophylaxis. *TH Open* 2020;4(1):e59-e65. doi: https://dx.doi.org/10.1055/s-0040-1705137
49. Spyropoulos AC, Anderson FA, FitzGerald G, et al. Predictive and Associative Models to Identify Hospitalized Medical Patients at Risk for VTE. *Chest* 2011;140(3):706-14. doi: https://doi.org/10.1378/chest.10-1944
50. Stuck AK, Spirk D, Schaudt J, et al. Risk assessment models for venous thromboembolism in acutely ill medical patients. A systematic review. *Thrombosis and Haemostasis* 2017;117(4):801-08. doi: https://dx.doi.org/10.1160/TH16-08-0631
51. Tadesse TA, Kedir HM, Fentie AM, et al. Venous Thromboembolism Risk and Thromboprophylaxis Assessment in Surgical Patients Based on Caprini Risk Assessment Model. *Risk Management and Healthcare Policy* 2020;13:2545-52. doi: https://dx.doi.org/10.2147/RMHP.S272852
52. Taha H, Govindraj E, Jaber F, et al. Improving venous thromboembolism prophylaxis through critical thinking and health informatics. *BMJ Open Quality* 2020;9(2) doi: https://dx.doi.org/10.1136/bmjoq-2019-000885
53. Tung EC, Yu S-Y, Shah K, et al. Reassessment of venous thromboembolism and bleeding risk in medical patients receiving VTE prophylaxis. *Journal of Evaluation in Clinical Practice* 2020;26(1):18-25. doi: https://dx.doi.org/10.1111/jep.13213
54. Veith J, Collier W, Rockwell WB, et al. Direct Comparison of Patient-completed and Physician-completed Caprini Scores for Plastic Surgery Patients. *Plastic and Reconstructive Surgery Global Open* 2019;7(8):e2363. doi: https://dx.doi.org/10.1097/GOX.0000000000002363
55. Winoker JS, Paulucci DJ, Anastos H, et al. MP76-07 Predicting complications following robot-assisted partial nephrectomy with the ACS-NSQIP universal surgical risk calculator. *Journal of Urology* 2017;197(4S):e1015.
56. Yale S, Medlin S, Liang H, et al. Risk assessment model for venothromboembolism in post-hospitalized patients. *International Angiology* 2005;24(3):250.

For peer review only

57. Ye F, Stalvey C, Khuddus MA, et al. A systematic review of mobility/immobility in thromboembolism risk assessment models for hospitalized patients. *Journal of Thrombosis and Thrombolysis* 2017;44(1):94-103. doi: https://dx.doi.org/10.1007/s11239-017-1501-5

58. Zakai NA, Callas PW, Repp AB, et al. Venous thrombosis risk assessment in medical inpatients: the medical inpatients and thrombosis (MITH) study. *J Thromb Haemost* 2013;11(4):634-41.

59. Zambelli R, Nemeth B, Touw C, et al. Is the caprini score a reliable tool to predict the risk of venous thrombosis in orthopedic patients. *Research and Practice in Thrombosis and Haemostasis* 2020;4(SUPPL 1):1194-95. doi: http://dx.doi.org/10.1002/rth2.12393

60. Zhou H-X, Peng L-Q, Yan Y, et al. Validation of the Caprini risk assessment model in Chinese hospitalized patients with venous thromboembolism. *Thrombosis Research* 2012;130(5):735-40. doi: https://doi.org/10.1016/j.thromres.2012.08.001

For peer review only

APPENDIX S3: WIDELY EVALUATED GENERIC RAMS, THEIR ASSOCIATED CHARACTERISTICS AND COMPOSITE CLINICAL VARIABLES

Characteristics	Name of VTE risk assessment model					
	**Caprini Score**	**Padua Prediction Score**	**IMPROVE Predictive score**	**IMPROVE Associative score**	**Geneva Risk Score**	**Kucher Score**
**General**						
Author, year	Caprini 2005[1]	Barbar 2010[2]	Tapson 2007[3]	Spyropoulos 2011[4]	Chopard 2006[5]	Kucher 2005[6]
Applicable cohort	Surgical and medical	Medical	Medical	Medical	Medical	Surgical and medical
Design	Ordinal with cumulative score	Dichotomous variables with cumulative score	Dichotomous variables with VTE probability estimate	Dichotomous variables with VTE probability estimate	Dichotomous variables with cumulative score	Dichotomous variables with cumulative score
Number of VTE risk variables	31	11	4	7	19	8
C-statistic (range) across medical, surgical and trauma cohorts	0.53 - 0.87 (12 studies)	0.594 - 0.756 (7 studies)	0.57 – 0.65 (2 studies)	0.63 – 0.7731 (4 studies)	0.61 (1 study)	0.563-0.756 (4 studies)
When is pharmacological thromboprophylaxis recommended?	Score ≥5	Score ≥4	No specific threshold Identified	No specific threshold identified	Score ≥3	Score ≥4
**Clinical Variables**						
Patient related						
Active cancer	Yes	Yes	Yes	Yes	Yes	Yes (Major risk)
Age	Yes	Yes (≥70)	Yes (≥60)	Yes (≥60)	Yes (≥60)	Yes (≥70 Minor risk)
Dehydration	No	No	No	No	Yes	No
Thrombophilia	Yes (generic and named conditions)	Yes (generic)	Yes (generic)	Yes (generic)	Yes (generic)	Yes (Major risk)
Obesity	Yes (≥25kg/m^2)	Yes (≥30kg/m^2)	No	No	Yes (≥30kg/m^2)	Yes (≥30kg/m^2 Minor risk)
Comorbidity	Yes (1 to 5 points for individual comorbidities)	Yes (1 point each for several individual comorbidities)	No	No	Yes (2 points each for several individual comorbidities)	No
Prior VTE	Yes	Yes	Yes	Yes	Yes	Yes (Major risk)
Family history of VTE	Yes	No	No	No	No	No
Use of HRT	Yes	Yes	No	No	Yes	Yes (Minor risk)
Use of oestrogen containing contraceptive therapy	Yes	Yes	No	No	Yes	Yes (Minor risk)
Varicose veins	Yes	No	No	No	No	No
Pregnancy or postpartum period	No	No	No	No	Yes	No
Unexplained stillbirth or spontaneous abortions	Yes (≥3 spontaneous abortions)	No	No	No	No	No
Current swollen legs	Yes	No	No	No	Yes	No
Current central venous access	Yes	No	No	No	No	No
Recent major surgery	Yes (<1 month)	Yes (<1 month)	No	No	No	No
Recent use of plaster cast immobilisation	Yes (<1 month)	No	No	No	No	No

For peer review only

Lower limb paralysis	Yes	No	No	Yes	No	No
Travel related	No	No	No	No	Yes (>6hours)	No
Admission related						
Reduced mobility	Yes (variable points)	Yes	No	Yes (≥7days)	Yes (≥3 days)	Yes (Minor risk)
Arthroplasty surgery	Yes	No	No	No	No	No
Hip fracture	Yes	No	No	No	No	No
Pelvic or lower limb surgery	Yes (arthroscopic)	No	No	No	No	No
Total anaesthetic and surgical time	Yes (≥45mins)	No	No	No	No	Yes (≥60mins intermediate risk)
Acute surgical admission	No	No	No	No	No	No
Acute infection	No	Yes	No	No	Yes	No
Acute rheumatologic disorder	No	Yes	No	No	Yes	No
Critical care admission	No	No	No	Yes	No	No
Surgery leading to reduced mobility	Yes	No	No	No	No	No
Other						
'Other risk factors'	Yes	No	No	No	No	No

HRT, hormone replacement therapy; VTE, venous thromboembolism

REFERENCES (APPENDIX S3)

1. Caprini JA, Arcelus JI, Hasty JH, et al. Clinical assessment of venous thromboembolic risk in surgical patients. *Semin Thromb Hemost* 1991;17:304-12.

2. Barbar S, Noventa F, Rossetto V, et al. A risk assessment model for the identification of hospitalized medical patients at risk for venous thromboembolism: the Padua Prediction Score. *Journal of Thrombosis and Haemostasis* 2010;8(11):2450-57. doi: 10.1111/j.1538-7836.2010.04044.x

3. Tapson VF, Decousus H, Pini M, et al. Venous thromboembolism prophylaxis in acutely ill hospitalized medical patients: findings from the International Medical Prevention Registry on Venous Thromboembolism. *Chest* 2007;132(3):936-45.

4. Spyropoulos AC, Anderson FA, FitzGerald G, et al. Predictive and Associative Models to Identify Hospitalized Medical Patients at Risk for VTE. *Chest* 2011;140(3):706-14. doi: https://doi.org/10.1378/chest.10-1944

5. Chopard P, Spirk D, Bounameaux H, et al. Identifying acutely ill medical patients requiring thromboprophylaxis. . *J Thromb Haemost* 2006;4(4):915-6.

6. Kucher N, Koo S, Quiroz R, et al. Electronic alerts to prevent venous thromboembolism among hospitalized patients. *N Engl J Med* 2005;352(10):969-77.

For peer review only

APPENDIX S4: SUMMARY OF PREDICTIVE PERFORMANCE FOR STUDIES INVOLVING HOSPITAL INPATIENTS WHO REQUIRED CARE FOR CANCER, STROKE, BURN INJURIES AND SEPSIS OR WERE A MIXED MEDICAL/SURGICAL COHORT

Data source	Endpoint	Incidence	Risk assessment model	Threshold or cut-off	Predictive performance		
					C-statistic (95% CI)	Sensitivity (95% CI)	Specificity (95% CI)
**MIXED INPATIENTS**							
Autar, 2003[22]	DVT	18.9%	•Novel (Autar, 2003)	Risk score ≤6	NR	100% (NR)	100% (NR)
			•Novel (Autar, 2003)	Risk score 7-10	NR	86.0% (NR)	68.0% (NR)
			•Novel (Autar, 2003)	Risk score 11-14	NR	68.0% (NR)	31.0% (NR)
			•Novel (Autar, 2003)	Risk score ≥15	NR	25.0% (NR)	10.0% (NR)
Elias, 2017[34]	VTE	0.8%	•Padua (automated)	Risk score ≥5	0.81 (0.79, 0.83)	85.4% (NR)	53.3% (NR)
Chen, 2018[30]	DVT	NA	•Caprini	Risk score ≥4	NR	73.8% (NR)	64.7% (NR)
			•Caprini	Risk score ≥5	NR	62.8% (NR)	82.6% (NR)
			•Padua	Risk score ≥3	NR	53.5% (NR)	82.1% (NR)
			•Padua	Risk score ≥4	NR	42.1% (NR)	92.5% (NR)
Bo, 2020[29]	DVT	0.9%	•Caprini	Risk score ≥3.5	0.74 (0.71, 0.77)	75.0% (NR)	62.0% (NR)
**CANCER INPATIENTS**							
Abdel-Razeq, 2010[21]	VTE	3.5%	•Caprini (modified)	Risk score ≥3	NR	100% (NR)	9.2% (NR)
			•Caprini (modified)	Risk score ≥5	NR	57.1% (NR)	53.2% (NR)
Patell, 2017[54]	VTE	3.8%	•Khorana	Risk score ≥3	NR	18.9% (NR)	87.2% (NR)
Hu, 2020[42]	VTE	NA	•Caprini	Risk score ≥5	0.71 (0.66, 0.75)	82.4% (NR)	46.2% (NR)
			•Khorana	Risk score ≥2	0.58 (0.53, 0.63)	35.3% (NR)	78.7% (NR)
Shang, 2020[60]	VTE	NA	•Caprini 2009	Risk score ≥3	0.72 (0.70, 0.74)	83.5% (NR)	52.7% (NR)
			•Caprini 2013	Risk score ≥5	0.80 (0.78, 0.82)	80.9% (NR)	65.9% (NR)
**BURNS INPATIENTS**							
Pannucci, 2012[53]	VTE	1.0%	•Novel (Pannucci 2012)	NR	0.75	NR	NR
**POST-STROKE INPATIENTS**							
Liu, 2014[44]	DVT	10.5%	•Post-stroke DVT Prediction System	NR	0.65 (0.59, 0.70)	NR	NR
**SEPSIS INPATIENTS**							
Vardi, 2013[64]	VTE	1.3%	•Padua	NR	•All patients 0.58 (0.43, 0.73)	•All patients NR	•All patients NR
			•Padua	NR	•No prophylaxis 0.54 (0.37, 0.71)	•No prophylaxis NR	•No prophylaxis NR

CI, confidence interval; DVT, deep vein thrombosis; NA, not applicable; NR, not reported; VTE, venous thromboembolism

For peer review only

PRISMA 2009 Checklist

Section/topic	#	Checklist item	Reported on page #
**TITLE**			
Title	1	Identify the report as a systematic review, meta-analysis, or both.	1
**ABSTRACT**			
Structured summary	2	Provide a structured summary including, as applicable: background; objectives; data sources; study eligibility criteria, participants, and interventions; study appraisal and synthesis methods; results; limitations; conclusions and implications of key findings; systematic review registration number.	2-3
**INTRODUCTION**			
Rationale	3	Describe the rationale for the review in the context of what is already known.	5
Objectives	4	Provide an explicit statement of questions being addressed with reference to participants, interventions, comparisons, outcomes, and study design (PICOS).	5
**METHODS**			
Protocol and registration	5	Indicate if a review protocol exists, if and where it can be accessed (e.g., Web address), and, if available, provide registration information including registration number.	3, 6
Eligibility criteria	6	Specify study characteristics (e.g., PICOS, length of follow-up) and report characteristics (e.g., years considered, language, publication status) used as criteria for eligibility, giving rationale.	6
Information sources	7	Describe all information sources (e.g., databases with dates of coverage, contact with study authors to identify additional studies) in the search and date last searched.	6-7
Search	8	Present full electronic search strategy for at least one database, including any limits used, such that it could be repeated.	Online supplementary - Appendix S1
Study selection	9	State the process for selecting studies (i.e., screening, eligibility, included in systematic review, and, if applicable, included in the meta-analysis).	7
Data collection process	10	Describe method of data extraction from reports (e.g., piloted forms, independently, in duplicate) and any processes for obtaining and confirming data from investigators.	7
Data items	11	List and define all variables for which data were sought (e.g., PICOS, funding sources) and any assumptions and simplifications made.	7
Risk of bias in individual studies	12	Describe methods used for assessing risk of bias of individual studies (including specification of whether this was done at the study or outcome level), and how this information is to be used in any data synthesis.	7-8
Summary measures	13	State the principal summary measures (e.g., risk ratio, difference in means).	8
Synthesis of results	14	Describe the methods of handling data and combining results of studies, if done, including measures of consistency (e.g., I^2) for each meta-analysis.	8

For peer review only - http://bmjopen.bmj.com/site/about/guidelines.xhtml

PRISMA 2009 Checklist

Section/topic	#	Checklist item	Reported on page #
Risk of bias across studies	15	Specify any assessment of risk of bias that may affect the cumulative evidence (e.g., publication bias, selective reporting within studies).	7-8
Additional analyses	16	Describe methods of additional analyses (e.g., sensitivity or subgroup analyses, meta-regression), if done, indicating which were pre-specified.	N/A
**RESULTS**			
Study selection	17	Give numbers of studies screened, assessed for eligibility, and included in the review, with reasons for exclusions at each stage, ideally with a flow diagram.	8; Figure 1; Online supplementary - Appendix S2
Study characteristics	18	For each study, present characteristics for which data were extracted (e.g., study size, PICOS, follow-up period) and provide the citations.	9-15
Risk of bias within studies	19	Present data on risk of bias of each study and, if available, any outcome level assessment (see item 12).	15-17
Results of individual studies	20	For all outcomes considered (benefits or harms), present, for each study: (a) simple summary data for each intervention group (b) effect estimates and confidence intervals, ideally with a forest plot.	18-20; Figure 3; Online supplementary – Appendix S4
Synthesis of results	21	Present results of each meta-analysis done, including confidence intervals and measures of consistency.	N/A
Risk of bias across studies	22	Present results of any assessment of risk of bias across studies (see Item 15).	18, Figure 2
Additional analysis	23	Give results of additional analyses, if done (e.g., sensitivity or subgroup analyses, meta-regression [see Item 16]).	N/A
**DISCUSSION**			
Summary of evidence	24	Summarize the main findings including the strength of evidence for each main outcome; consider their relevance to key groups (e.g., healthcare providers, users, and policy makers).	21-23
Limitations	25	Discuss limitations at study and outcome level (e.g., risk of bias), and at review-level (e.g., incomplete retrieval of identified research, reporting bias).	22
Conclusions	26	Provide a general interpretation of the results in the context of other evidence, and implications for future research.	23
**FUNDING**			
Funding	27	Describe sources of funding for the systematic review and other support (e.g., supply of data); role of funders for the systematic review.	24

From: Moher D, Liberati A, Tetzlaff J, Altman DG, The PRISMA Group (2009). Preferred Reporting Items for Systematic Reviews and Meta-Analyses: The PRISMA Statement. PLoS Med 6(6): e1000097. doi:10.1371/journal.pmed1000097

For peer review only - http://bmjopen.bmj.com/site/about/guidelines.xhtml

PRISMA 2009 Checklist

For more information, visit: www.prisma-statement.org.

For peer review only

Risk assessment models for venous thromboembolism in hospitalised adult patients – a systematic review

Journal:	*BMJ Open*
Manuscript ID	bmjopen-2020-045672.R2
Article Type:	Original research
Date Submitted by the Author:	08-Jun-2021

Complete List of Authors:	Pandor, Abdullah; The University of Sheffield, ScHARR<br>Tonkins, Michael; The University of Sheffield, ScHARR<br>Goodacre, Steve; The University of Sheffield, ScHARR<br>Sworn, Katie; The University of Sheffield, ScHARR<br>Clowes, Mark; The University of Sheffield, ScHARR<br>Griffin, Xavier; Queen Mary University of London<br>Holland, Mark; University of Bolton, Department of Clinical and Biomedical Sciences<br>Hunt, Beverley; Guy's and St Thomas' NHS Foundation Trust, Dept of Haematology<br>de Wit, Kerstin; McMaster University Department of Medicine<br>Horner, Daniel; Salford Royal NHS Foundation Trust

**Primary Subject Heading**:	Haematology (incl blood transfusion)
Secondary Subject Heading:	Haematology (incl blood transfusion)
Keywords:	VASCULAR MEDICINE, HAEMATOLOGY, Anticoagulation < HAEMATOLOGY, Quality in health care < HEALTH SERVICES ADMINISTRATION & MANAGEMENT

SCHOLARONE™
Manuscripts

I, the Submitting Author has the right to grant and does grant on behalf of all authors of the Work (as defined in the below author licence), an exclusive licence and/or a non-exclusive licence for contributions from authors who are: i) UK Crown employees; ii) where BMJ has agreed a CC-BY licence shall apply, and/or iii) in accordance with the terms applicable for US Federal Government officers or employees acting as part of their official duties; on a worldwide, perpetual, irrevocable, royalty-free basis to BMJ Publishing Group Ltd ("BMJ") its licensees and where the relevant Journal is co-owned by BMJ to the co-owners of the Journal, to publish the Work in this journal and any other BMJ products and to exploit all rights, as set out in our licence.

The Submitting Author accepts and understands that any supply made under these terms is made by BMJ to the Submitting Author unless you are acting as an employee on behalf of your employer or a postgraduate student of an affiliated institution which is paying any applicable article publishing charge ("APC") for Open Access articles. Where the Submitting Author wishes to make the Work available on an Open Access basis (and intends to pay the relevant APC), the terms of reuse of such Open Access shall be governed by a Creative Commons licence – details of these licences and which Creative Commons licence will apply to this Work are set out in our licence referred to above.

Other than as permitted in any relevant BMJ Author's Self Archiving Policies, I confirm this Work has not been accepted for publication elsewhere, is not being considered for publication elsewhere and does not duplicate material already published. I confirm all authors consent to publication of this Work and authorise the granting of this licence.

For peer review only

Complete manuscript title:

Risk assessment models for venous thromboembolism in hospitalised adult patients – a systematic review

Short title: Predicting VTE in hospitalised patients

Authors:

Abdullah Pandor, ScHARR, The University of Sheffield, Sheffield, UK

Michael Tonkins, ScHARR, The University of Sheffield, Sheffield, UK

Steve Goodacre, ScHARR, The University of Sheffield, Sheffield, UK

Katie Sworn, ScHARR, The University of Sheffield, Sheffield, UK

Mark Clowes, ScHARR, The University of Sheffield, Sheffield, UK

Xavier L Griffin, Queen Mary University of London, London, UK

Mark Holland, University of Bolton, Bolton, UK

Beverley J Hunt, Guy's and St Thomas' NHS Foundation Trust, London, UK

Kerstin de Wit, Department of Medicine, McMaster University, Canada

Daniel Horner, Salford Royal NHS Foundation Trust, Salford, UK

Corresponding author: Abdullah Pandor, ScHARR, The University of Sheffield, Regent Court, 30 Regent Street, Sheffield, S1 4DA, telephone +44 (0) 114 222 0778, email: a.pandor@sheffield.ac.uk

Word count: 3227

For peer review only - http://bmjopen.bmj.com/site/about/guidelines.xhtml

ABSTRACT

INTRODUCTION

Hospital acquired thrombosis accounts for a large proportion of all venous thromboembolism (VTE), with significant morbidity and mortality. This subset of VTE can be reduced through accurate risk assessment and tailored pharmacological thromboprophylaxis. This systematic review aimed to determine the comparative accuracy of risk assessment models (RAMs) for predicting VTE in patients admitted to hospital.

METHODS

A systematic search was performed across five electronic databases (including MEDLINE, EMBASE and the Cochrane Library) from inception to February 2021. All primary validation studies were eligible if they examined the accuracy of a multivariable RAM (or scoring system) for predicting the risk of developing VTE in hospitalised inpatients. Two or more reviewers independently undertook study selection, data extraction, and risk of bias assessments using the PROBAST tool. We used narrative synthesis to summarise the findings.

RESULTS

Among 6355 records, we included 51 studies, comprising 24 unique validated RAMs. The majority of studies included hospital inpatients who required medical care (21 studies), were undergoing surgery (15 studies) or receiving care for trauma (4 studies). The most widely evaluated RAMs were the Caprini RAM (22 studies), Padua prediction score (16 studies), IMPROVE models (8 studies), the Geneva risk score (4 studies) and the Kucher score (4 studies). C-statistics varied markedly between studies and between models, with no one RAM performing obviously better than other models. Across all models, C-statistics were often weak (<0.7), sometimes good (0.7-0.8) and a few were excellent (>0.8). Similarly, estimates for sensitivity and specificity were highly variable. Sensitivity estimates ranged from 12.0% to 100% and specificity estimates ranged from 7.2% to 100%.

For peer review only - http://bmjopen.bmj.com/site/about/guidelines.xhtml

CONCLUSION

Available data suggest that RAMs have generally weak predictive accuracy for VTE. There is insufficient evidence and too much heterogeneity to recommend the use of any particular RAM.

PROSPERO REGISTRATION

Steve Goodacre, Abdullah Pandor, Katie Sworn, Daniel Horner, Mark Clowes. A systematic review of venous thromboembolism RAMs for hospital inpatients. PROSPERO 2020 CRD42020165778 Available from https://www.crd.york.ac.uk/prospero/display_record.php?RecordID=165778

KEYWORDS

Hospitalization, inpatients, risk assessment, systematic review, venous thromboembolism

For peer review only

ARTICLE SUMMARY

Strengths and limitations of this study

- This systematic review provides an up-to-date comprehensive review of risk assessment models for predicting venous thromboembolism in patients admitted to hospital.
- The newly developed PROBAST tool was used to evaluate the risk of bias and applicability of the available evidence.
- Heterogeneity in the included studies (participants, inclusion criteria, clinical condition, outcome definition and measurement) and variable reporting of items precluded meta-analysis.
- Limitations of the existing evidence and areas of future research are highlighted.

For peer review only

For peer review only - http://bmjopen.bmj.com/site/about/guidelines.xhtml

INTRODUCTION

Venous thromboembolism (VTE) is an important and life threatening complication of hospitalisation and illness, and is associated with significant morbidity and mortality.[1 2] Globally, an estimated 10 million VTE episodes are diagnosed each year; over half of these episodes are associated with hospital inpatients stays and result in significant loss of disability adjusted life years.[3 4] Consequently, there has been a substantial and sustained focus on VTE prevention over the last three decades, with good evidence indicating a reduction in morbidity with primary thromboprophylaxis in hospitalised patients[5-8] Despite this evidence, thromboprophylaxis remains either underused or inappropriately applied.[9]

Risk assessment models (RAMs) have been developed to help stratify the risk of VTE amongst hospitalised patients.[10] These models use clinical information from the patient's history and examination to identify those with an increased risk of developing VTE who are most likely to benefit from pharmacological prophylaxis. Inappropriate use of VTE prophylaxis may not reduce VTE rates and may cause unnecessary harm.[11] Whilst RAMs could improve the ratio of benefit to risk and benefit to cost, it is unclear which VTE RAM should be applied to guide decision-making for prophylaxis in clinical practice and thereby optimise patient care.

The current review extends and updates three broadly overlapping existing reviews.[10 12 13] Whilst these reviews identified the use of various (derived and validated) RAMs for VTE in hospitalised patients, they did not find any evidence to suggest which RAM was superior. The aim of this systematic review was to identify primary validation studies (as derivation studies may give an overoptimistic assessment of model performance measures) and determine the accuracy of individual RAMs for predicting the risk of developing VTE in hospital inpatients.

METHODS

A systematic review was undertaken in accordance with the general principles recommended in the Preferred Reporting Items for Systematic Reviews and Meta-Analyses (PRISMA) statement.[14] This

For peer review only - http://bmjopen.bmj.com/site/about/guidelines.xhtml

review was part of a larger project on VTE RAMs for hospital inpatients[15] and was registered on the International Prospective Register of Systematic Reviews (PROSPERO) database (CRD42020165778).

Eligibility criteria

We sought studies evaluating RAMs which could be applied to a general inpatient population (medical, surgical or trauma), rather than disease-specific models. All primary validation studies that evaluated the accuracy (e.g. sensitivity, specificity, C-statistic) of a multivariable RAM (or scoring system) for predicting the risk of developing VTE were eligible for inclusion. We selected studies that included validation of the model in a group of patients that were not involved in model derivation. This involved either splitting the study cohort (internal) or using a new cohort (external). The study could have reported derivation of the model but we only used the validation data to estimate accuracy. The study population consisted of hospital inpatients including those who required medical care, undergoing any surgery (excluding day surgery) or received care following an injury. Studies that primarily focussed on children (aged under 16 years), women admitted to hospital for pregnancy-related reasons and any patient admitted to a level 2 or above critical care environment (e.g. patients requiring more detailed observation or intervention including support for a single failing organ system or post-operative care and those 'stepping down' from higher levels of care) were excluded. These patient groups have VTE risk profiles that differ markedly from the general inpatient population, making the use of a generic model inappropriate.

Data sources and searches

Potentially relevant studies were identified through searches of five electronic databases including MEDLINE (with MEDLINE In-process and Epub Ahead of Print), EMBASE and the Cochrane Library. The search strategy used free text and thesaurus terms and combined synonyms relating to the condition (e.g. VTE in medical inpatients) with risk prediction modelling terms. No language restrictions were used. However, as the current review updated three previous systematic reviews,[10 12 13] searches were limited by date from 2017 (last search date from earlier reviews)[10] to February

For peer review only - http://bmjopen.bmj.com/site/about/guidelines.xhtml

2021. Searches were supplemented by hand-searching the reference lists of all relevant studies (including existing systematic reviews); forward citation searching of included studies; contacting key experts in the field; and undertaking targeted searches of the World Wide Web using the Google search engine. Further details on the search strategy can be found in **online supplementary Appendix S1.**

Study selection

All titles were examined for inclusion by one reviewer (KS) and any citations that clearly did not meet the inclusion criteria (e.g. non-human, unrelated to VTE inpatients) were excluded. All abstracts and full text articles were then examined independently by two reviewers (KS and AP). Any disagreements in the selection process were resolved through discussion or if necessary, arbitration by a third reviewer (SG) and included by consensus.

Data extraction and quality assessment

Data relating to study design, methodological quality and outcomes were extracted by one reviewer (KS) into a standardised data extraction form and independently checked for accuracy by a second (AP or MT). Any discrepancies were resolved through discussion to achieve agreement. Where differences were unresolved, a third reviewer's opinion was sought (SG). Where multiple publications of the same study were identified, data were extracted and reported as a single study.

The methodological quality of each included study was assessed using PROBAST (Prediction model Risk Of Bias ASsessment Tool).[16 17] This instrument evaluates four key domains: patient selection, predictors, outcome, and analysis. Each domain is assessed in terms of risk of bias and the concern regarding applicability to the review (first three domains only). To guide the overall domain-level judgement about whether a study is at high, low or an unclear (in the event of insufficient data in the publication to answer the corresponding question) risk of bias, sub-domains within each domain include a number of signalling questions to help judge with bias and applicability concerns. An overall risk of bias for each individual study was defined as low risk when all domains were judged as

For peer review only - http://bmjopen.bmj.com/site/about/guidelines.xhtml

low; and high risk of bias when one or more domains were considered as high. Studies were assigned an unclear risk of bias if one or more domains were unclear and all other domains were low.

Data synthesis and analysis

We were unable to perform meta-analysis due to significant levels of heterogeneity between studies (participants, inclusion criteria, clinical condition) and variable reporting of items. As a result, a pre-specified narrative synthesis approach[18 19] was undertaken, with data being summarised in tables with accompanying narrative summaries that included a description of the included variables, statistical methods and performance measures (e.g. sensitivity, specificity and C-statistic [a value between 0.7 to 0.8 and >0.8 indicated good and excellent discrimination, respectively; and values <0.7 were considered weak[20]]), where applicable. All analyses were conducted using Microsoft Excel 2010 (Microsoft Corporation, Redmond, WA, USA).

Patient and public involvement

Patients and the public were not involved in the design or conduct of this systematic review.

RESULTS

Study flow

Figure 1 summarises the process of identifying and selecting relevant literature. Of the 6355 citations identified, 51 studies investigating 24 unique RAMs met the inclusion criteria. The majority of the articles were excluded primarily for not using a RAM for predicting the risk of developing VTE, having no useable or relevant outcome data or an inappropriate study design (e.g. derivation study, reviews, commentaries or editorials). A full list of excluded studies with reasons for exclusion is provided in **online supplementary Appendix S2.**

[INSERT FIGURE 1]

For peer review only - http://bmjopen.bmj.com/site/about/guidelines.xhtml

Study and patient characteristics

The design and participant characteristics of the 51 included studies[21-71] are summarised in **Table 1**. All studies were published between 2003 and 2020 and were undertaken in North America (n=24),[23 25 33-40 43 47 48 52-59 65 68 69] Asia (n=13),[29 30 42 44-46 60-63 67 70 71] Europe (n=9),[22 24 26-28 31 49 51 66] the Middle-East (n=2),[21 64] South America (n=1),[32] Australia (n=1)[41] and one study was intercontinental.[50] Sample sizes ranged from 70[40] to 1,099,093[43] patients in 37 observational cohort studies (11 prospective[21 22 24 28 29 32 44 51 52 56 64] [5 of which were multicentre] and 26 retrospective[23 25-27 33 34 36-41 43 46 49 50 53-55 58 59 62 63 65 68 69] [16 of which were multicentre] in design). Sample sizes in 14 case control studies[30 31 35 42 45 47 48 57 60 61 66 67 70 71] (4 of which were multicentre) ranged from 148[61] to 19,217[57] patients.

For peer review only - http://bmjopen.bmj.com/site/about/guidelines.xhtml

Table 1: Study and population characteristics

Author, year	Country	Design	Single/ multi-centre	Sample size	Population	Mean age (years)	Male	VTE prophylaxis	RAMs	Target condition (risk period)	Incidence	Validation methodology
Autar, 2003[22]	UK	P,CS	Single	148	Hospitalised patients from orthopaedic, medical, and surgical specialties	NR	NR	50%	•Novel (Autar, 2003)	DVT, not defined (90 days)	18.9%	External
Rogers, 2007[56]	USA	P,CS	Multi	91,308	Hospitalised surgical patients (undergoing vascular and general surgery)	NR	NR	NR	•Novel (Rogers, 2007)	VTE (30 days)	0.6%	Internal: split (half)
Abdel-Razeq, 2010[21]	Jordan	P,CS	Single	606	Hospitalised (>24 hours) cancer patients aged ≥18 years	51	51%	55%	•Caprini (modified)	VTE, symptomatic (60 days)	3.5%	External
Bahl, 2010[23]	USA	R,CS	Multi	8,216	Hospitalised surgical patients (undergoing general, vascular, and urologic surgery)	NR	NR	NR	•Caprini	VTE, (30 days)	1.4%	External
Barbar, 2010[24]	Italy	P,CS	Single	1,180	Hospitalised medical patients	NR	47%	16%	•Padua	VTE, symptomatic (90 days)	3.1%	External
Rothberg, 2011[58]	USA	R,CS	Multi	48,540	Hospitalised (≥ 3 days) medical patients aged ≥18 years	NR	NR	30%	•Novel (Rothberg 2011)	VTE, hospital associated (NR)	0.5%	Internal: split (20%)
Woller, 2011[69]	USA	R,CS	Multi	46,856	Hospitalised medical patients aged ≥18 years	61	46%	NR	•Intermountain<br>•Kucher	VTE, defined by ICD-9 codes (90 days)	4.5%	Internal: split (25%)
Pannucci, 2012[53]	USA and Canada	R,CS	Multi	5,761	Hospitalised (>2 days) patients with a burn injury aged ≥18 years	46	69%	NR	•Novel (Panunucci 2012)	VTE, not defined (NR)	1.0%	Internal: split (25%)
Rogers, 2012[55]	USA	R,CS	Multi	234,032	Hospitalised trauma patients	NR	NR	NR	•TESS	VTE (NR)	NR	Internal: split
Bilimoria, 2013[25]	USA	R,CS	Multi	88,053	Hospitalised surgical patients (undergoing colorectal surgery)	NR	NR	NR	•ACS NSQIP - Colon specific<br>•ACS NSQIP - Universal	DVT, not defined (30 days)	2.3%	External: split (by year)
Hegsted, 2013[39]	USA	R,CS	Single	2,281	Hospitalised (≥2 days) trauma patients aged ≥13 years	45	70%	NR	•RAP	DVT, not defined or PE (NR)	•DVT: 10.5%<br>•PE: 1.5%	External
Vardi, 2013[64]	Israel	P,CS	Single	1,080	Hospitalised (≥2 days) sepsis patients aged >18 years	75	52%	18%	•Padua	VTE, hospital associated (NR)	1.3%	External
Ho, 2014[41]	Australia	R,CS	Single	357	Hospitalised major trauma patients	NR	75%	NR	•TESS	VTE, symptomatic (NR)	20.7%	External
Liu, 2014[44]	China	P,CS	Single	287	Hospitalised acute stroke patients aged >18 years	NR	63%	22%	•Post-stroke DVT Prediction System	DVT (14±3 days)	10.5%	Internal: split (33%)
Mahan, 2014[47]	USA	CC	Multi	417	Hospitalised (≥3 days) medical patients aged ≥18 years	NR	49%	NR	•IMPROVE (7-factor)	VTE, hospital associated (92 days)	NA	External
Nendaz, 2014[51]	Switzerland	P,CS	Multi	1,478	Hospitalised (>24 hours) medical patients aged ≥18 years	65	53%	57%	•Geneva<br>•Padua	VTE, symptomatic including PE or DVT (90 days)	2.0%	External
Pannucci, 2014[52]	USA	P,CS	Multi	3,576	Hospitalised surgical patients aged ≥18 years	NR	NR	66%	•Novel (Panunucci 2014)	VTE (90 days)	1.4%	Internal: split (35%)

For peer review only

For peer review only - http://bmjopen.bmj.com/site/about/guidelines.xhtml

Rosenberg, 2014[57]	USA	CC	Multi	19,217	Hospitalised (≥3 days) medical patients aged ≥18 years	NR	47%	43%	•IMPROVE (7-factor)	VTE, defined by ICD-9 codes (90 days)	NA	External
Zhou 2014[71]	China	CC	Single	998	Hospitalised (≥2 days) medical patients aged >18 years	NR	58%	15%	•Caprini<br>•Padua	VTE, defined by ICD-10 codes (NR)	NA	External
Hewes, 2015[40]	USA	R,CS	Single	70	Hospitalised cancer patients (undergoing oesophagectomy)	NR	83%	96%	•Caprini (modified)	VTE (60 days)	14.3%	External
de Bastos, 2016[32]	Brazil	P,CS	Single	11,091	Hospitalised medical patients aged >18 years	50	61%	0%	•Caprini	VTE, symptomatic (NR)	0.3%	External
Grant, 2016[36]	USA	R,CS	Multi	63,548	Hospitalised (≥2 days) medical patients aged ≥18 years	66	45%	61%	•Caprini	VTE, hospital associated (90 days)	1.1%	External
Greene, 2016[37]	USA	R,CS	Multi	63,548	Acutely ill, hospitalised (≥2 days) medical patients aged ≥18 years	66	45%	61%	•IMPROVE (4-factor)<br>•Intermountain<br>•Kucher<br>•Padua	VTE, hospital associated (90 days)	1.1%	External
Hachey, 2016[38]	USA	R,CS	Single	232	Hospitalised surgical patients (undergoing segmenectomy, lobectomy or pneumonectomy for lung cancer)	NR	43%	92%	•Caprini	VTE (60 days)	5.2%	External
Lui, 2016[45]	China	CC	Single	640	Hospitalised (>2 days) medical patients aged ≥18 years	NR	52%	NR	•Caprini<br>•Padua	VTE (NR)	N/A	External
Lobastov, 2016[46]	Russia	R,CS [a]	Multi	140	Hospitalised high risk emergency surgery patients (undergoing general and neurosurgery)	69	49%	100%	•Caprini	DVT or PE, new (NR)	27.9%	External
Shaikh, 2016[59]	USA	R,CS	Multi	1,598	Hospitalised surgical patients (undergoing plastic surgery)	50	19%	34%	•Caprini	VTE, not defined (30 days)	1.5%	External
Elias, 2017[34]	USA	R,CS	Single	30,726	Hospitalised (>2 days) medical and surgical patients	NR	44%	21%	•Padua (automated)	VTE, defined by ICD-9 codes (NR)	0.8%	External
Frankel, 2017 (abstract)[35]	USA	CC	NR	149	Hospitalised surgical patients aged ≥18 years (undergoing robotic assisted laparoscopic prostatectomy)	NR	NR	NR	•Caprini	VTE, not defined (90 days)	NA	External
Krasnow, 2017 (abstract)[43]	USA	R,CS	Multi	1,099,093	Hospitalised surgical patients (major urological cancer surgery)	NR	NR	NR	•Caprini	VTE, symptomatic (90 days)	1.2%	External
Patell, 2017[54]	USA	R,CS	Single	2,780	Hospitalised (>24 hours) cancer patients aged >18 years	62 (median)	56%	65%	•Khorana	VTE, defined by ICD-9 codes (NR)	3.8%	External
Winoker, 2017[68]	USA	R,CS	Multi	300	Hospitalised surgical patients (undergoing urological surgery using robot-assisted partial nephrectomy)	61 (median)	62%	NR	•ACS NSQIP - Universal	VTE, not defined (NR)	0.3%	External
Blondon, 2018[28]	Switzerland	P,CS	Multi	1,478	Hospitalised (>24h) medical patients aged ≥18 years	65	53%	59%	•IMPROVE (7-factor)<br>•Geneva [b]<br>•Padua [b]	VTE, symptomatic including PE or DVT (90 days)	2.0%	External

For peer review only

For peer review only - http://bmjopen.bmj.com/site/about/guidelines.xhtml

Chen, 2018[30]	China	CC	Single	390	Hospitalised (>2 days) patients aged ≥18 years with and without DVT	NR	51%	41%	•Caprini<br>•Padua	DVT (NR)	NA	External
Dornbus, 2018 (abstract)[33]	USA	R,CS	NR	2,830	Hospitalised surgical patients (undergoing neurosurgery)	NR	NR	NR	•Caprini	VTE, not defined (NR)	NR	External
Vaziri, 2018[65]	USA	R,CS	Single	1,006	Hospitalised surgical patients (undergoing neurosurgery)	NR	46%	NR	•ACS NSQIP- Universal	VTE, not defined (NR)	1.3%	External
Vincentelli, 2018[66]	Italy	CC	Multi	1,215	Acutely ill, hospitalised medical patients aged >18 years	NR	44%	NR	•Chopard<br>•Kucher<br>•Padua	VTE (NR)	NA	External
Zhou, 2018[70]	China	CC	Single	1,804	Hospitalised (≥2 days) medical patients aged >18 years	NR	59%	5%	•Caprini<br>•Padua	VTE, defined by ICD-10 codes (NR)	NA	External
Blondon, 2019a[26]	Italy	R,CS [a]	Single	1,180	Hospitalised medical patients	72	47%	20%	•Geneva (simplified)	VTE, symptomatic (90 days)	3.1%	External
Blondon, 2019b (abstract)[27]	Switzerland	R,CS [a]	Multi	991	Hospitalised elderly medical patients	75	55%	NR	•Geneva (simplified)<br>•IMPROVE (NR)<br>•Padua	VTE, symptomatic (NR)	15.0%	External
Cobben, 2019[31]	Netherlands	CC	Multi	556	Hospitalised (>24h) medical patients	NR	52%	NR	•Caprini<br>•Geneva<br>•IMPROVE (4-factor)<br>•IMPROVE (7-factor)<br>•Intermountain<br>•Kucher<br>•Lecumberri<br>•NAVAL<br>•NICE Guideline<br>•Padua<br>•PRETEMED Guideline<br>•Zakai et al (model 2)	VTE (NR)	NA	External
Tachino, 2019[62]	Japan	R,CS	Multi	859	Hospitalised (>24h) trauma patients aged ≥18 years	NR	64%	NR	•RAP<br>•Quick RAP	VTE (NR)	3.0%	External (RAP)/internal (qRAP)
Tian, 2019[63]	China	R,CS	Single	533	Hospitalised surgical patients (undergoing thoracic surgery)	53	53%	0%	•Caprini<br>•Khorana<br>•Padua<br>•Novel (Rogers, 2007)	VTE (NR)	8.4%	External
Bo, 2020[29]	China	P,CS	Multi	24,524	Hospitalised (≥2 days) patients from medical and surgical specialties aged ≥18 years	57	57	NR	•Caprini	DVT (NR)	0.9%	External
Hu, 2020[42]	China	CC	Single	442	Hospitalised (≥2 days) cancer patients aged ≥18 years	NR	62	3.8	•Caprini<br>•Khorana	VTE, defined by ICD-10 codes (NR)	NA	External
Mlaver, 2020[48]	USA	CC	Single	189	Hospitalised surgical patients (undergoing hepatobiliary, colorectal, endocrine, plastic, transplant, or general surgery)	NR	NR	NR	•Caprini<br>•Padua	VTE, not defined (NR)	NA	External

For peer review only

For peer review only - http://bmjopen.bmj.com/site/about/guidelines.xhtml

Moumneh, 2020[49]	France	R,CS [a]	Multi	14,660	Acutely ill, hospitalised (≥2 days) medical patients aged ≥40 years	73	50	46.1	•Caprini<br>•Padua<br>•IMPROVE (7 factor)	VTE, symptomatic including PE or DVT (90 days)	1.8%	External
Nafee, 2020[50]	35 countries	R,CS [a]	Multi	6,459	Hospitalised medical patients	76	45	100	•IMPROVE (NR)<br>•Novel (Nafee, 2020a)<br>•Novel (Nafee, 2020b)	VTE (77 days)	6.3%	External
Shang, 2020[60]	China	CC	Single	2,878	Hospitalised (≥2 days) cancer patients aged ≥18 years	56	47	NR	•Caprini (2009)<br>•Caprini (2013)	VTE, (NR)	NA	External
Shen, 2020[61]	China	CC	Single	148	Hospitalised (≥2 days) medical patients aged ≥18 years	NR	NR	0	•Novel (Shen 2020)	VTE, not defined (NR)	NA	Internal: split (by time, months)
Wang, 2020[67]	China	CC	Single	1,579	Hospitalised (≥3 days) medical patients aged ≥18 years	53	57	NR	•Padua	VTE, (NR)	NA	Internal: split (by year, months)

[a] Prospective cohort study with retrospective analysis, thus classified as retrospective cohort study
[b] Data overlap with Nendaz, 2014[51]
ACS NSQIP, American College of Surgeons National Surgical Quality Improvement Program; CC, case-control; CS, cohort study; DVT, deep vein thrombosis; NA, not applicable; NR, not reported; P, prospective; PE, pulmonary embolism; R, retrospective; RAMs, risk assessment models; RAP, Risk Assessment Profile; TESS, Trauma Embolic Scoring System VTE, venous thromboembolism

For peer review only
For peer review only - http://bmjopen.bmj.com/site/about/guidelines.xhtml

The vast majority of studies evaluated VTE risk assessment in hospital inpatients who required medical care (n=21),[24 26-28 31 32 36 37 45 47 49-51 57 58 61 66 67 69-71] were undergoing surgery (n=15)[23 25 33 35 38 40 43 46 48 52 56 59 63 65 68] or were a mixed medical and surgical cohort (n=4).[22 29 30 34] The remaining studies focused on patients receiving care for trauma (n=4),[39 41 55 62] cancer (n=4),[21 42 54 60] stroke (n=1),[44] burn injuries (n=1)[53] and sepsis (n=1).[64]. The mean age ranged from 45 years[39] to 76 years[50] (not reported in 29 studies)[22-25 30 31 33-35 38 40-45 47 48 52 55-58 61 62 65 66 70 71] and the proportion of female subjects ranged from 17%[40] to 81%[59] (not reported in 12 studies).[22 23 25 33 35 43 48 52 55 56 58 61]

VTE definition and case ascertainment

The majority of studies (n=37)[21 23 24 26-32 36-38 40-47 49-52 55-58 60 62-64 66 67 70 71] defined the VTE endpoint (DVT and or PE) as being objectively confirmed. Of the remainder, three studies[34 54 69] had no objective confirmation of VTE and 11 studies[22 25 33 35 39 48 53 59 61 65 68] did not report the methods for diagnosis confirmation. In terms of VTE risk period, half of the studies (n=23)[21-26 28 35-38 40 43 44 47 49-52 56 57 59 69] utilised the RAMs to predict the occurrence of VTE within 3 months of the index hospitalisation. The remaining studies did not report the VTE risk period. The reported incidence of VTE ranged widely from 0.3%[32 68] to 27.9%[46] depending on definition, study design and study participants (e.g. medical, surgical or trauma).

RAMs

The studies included in this review evaluated 24 validated unique RAMs. The most widely evaluated models were the Caprini RAM (22 studies),[21 23 29-33 35 36 38 40 42 43 45 46 48 49 59 60 63 70 71] Padua prediction score (16 studies),[24 27 28 30 31 34 37 45 48 49 63 64 66 67 70 71] IMPROVE models (8 studies),[27 28 31 37 47 49 50 57] the Geneva risk score (4 studies)[26-28 31] and the Kucher score (4 studies).[31 37 66 69] A summary of their associated characteristics and composite clinical variables is provided in **online supplementary Appendix S3.**

Statistical methods

For peer review only - http://bmjopen.bmj.com/site/about/guidelines.xhtml

Statistical methods varied significantly between studies. Most studies reported the discrimination of the RAMs using a combination of the C-statistic and sensitivity or specificity. A minority reported calibration measures, such as the Hosmer-Lemeshow test.[23 40 41 50]

Risk of bias and applicability assessment

The overall methodological quality of the 51 included studies[21-71] is summarised in **Table 2 and Figure 2**. The methodological quality of the included studies was variable, with most studies having high or unclear risk of bias in at least one item of the PROBAST tool. The main sources of potential bias were related to the following domains:

1) Patient selection factors, such as retrospective data collection, incomplete patient enrolment, or unclear criteria for patients receiving VTE prophylaxis;

2) Predictor and outcome bias arising from inappropriate inclusion of predictors within RAMs, unclear methods of outcome definition, low event rates, and missing predictor or outcome data;

3) Analysis factors, such as small sample sizes, inappropriate handling of missing data and failure in reporting relevant performance measures such as calibration.

Assessment of applicability to the review question led to the majority of studies being classed either as high (n=35)[21 22 29 30 32 34 35 38-49 52-55 59-68 70 71] or unclear (n=12)[23 24 27 28 31 33 50 51 56-58 69] risk of inapplicability. These assessments were generally related to patient selection (highly selected study populations e.g. single pathologies, single site settings), predictors (inconsistency in definition, assessment or timing of predictors) and outcome determination.

For peer review only - http://bmjopen.bmj.com/site/about/guidelines.xhtml

Table 2. Summary of each study's risk of bias and applicability concern using the PROBAST tool – Review authors' judgements

Author, year	Risk of Bias				Concern regarding Applicability			OVERALL	OVERALL
	1. Participant selection	2. Predictors	3. Outcome	4. Analysis	1. Participant selection	2. Predictors	3. Outcomes	RISK OF BIAS	APPLICABILITY
Abdel-Razeq, 2010[21]	High	High	High	High	High	High	High	High	High
Autar, 2003[22]	High	High	High	High	High	High	High	High	High
Bahl, 2010[23]	High	High	High	High	Unclear	Unclear	Unclear	High	Unclear
Barbar, 2010[24]	Low	Unclear	Unclear	High	Low	Unclear	Unclear	High	Unclear
Bilimoria, 2013[25]	Low	Low	Low	High	Low	Low	Low	High	Low
Blondon, 2019a[26]	Low	Unclear	High	High	Low	Low	Low	High	Low
Blondon, 2019b (abstract)[27]	Unclear	Unclear	Unclear	Unclear	Unclear	Unclear	Unclear	Unclear	Unclear
Blondon, 2018[28]	Low	Unclear	Unclear	High	Unclear	Low	Unclear	High	Unclear
Bo, 2020[29]	Low	Unclear	Unclear	Unclear	High	Low	Low	Unclear	High
Chen, 2018[30]	High	High	High	High	Unclear	High	High	High	High
Cobben, 2019[31]	Unclear	Unclear	High	High	Unclear	Low	Unclear	High	Unclear
de Bastos, 2016[32]	High	Low	High	High	High	Low	Low	High	High
Dornbus, 2018 (abstract)[33]	High	Unclear	High	Unclear	Unclear	Unclear	Unclear	High	Unclear
Elias, 2017[34]	High	Unclear	High	High	Low	Low	High	High	High
Frankel, 2017 (abstract)[35]	High	Unclear	Unclear	High	High	Unclear	Unclear	High	High
Grant, 2016[36]	High	Unclear	Unclear	Unclear	Low	Low	Low	High	Low
Greene, 2016[37]	Unclear	Unclear	Unclear	Unclear	Low	Low	Low	Unclear	Low
Hachey, 2016[38]	High	Unclear	Unclear	High	High	Low	High	High	High
Hegsted, 2013[39]	High	Unclear	High	High	High	Low	Unclear	High	High
Hewes, 2015[40]	High	Unclear	Unclear	High	High	Unclear	Low	High	High
Ho, 2014[41]	Unclear	Unclear	Unclear	High	High	Unclear	Unclear	High	High
Hu, 2020[42]	Unclear	Unclear	Unclear	Unclear	High	Unclear	Unclear	Unclear	High
Krasnow, 2017 (abstract)[43]	Unclear	Unclear	Unclear	Unclear	High	Unclear	Unclear	Unclear	High
Liu, 2014[44]	Low	Low	Unclear	Unclear	High	High	High	Unclear	High
Liu, 2016[45]	High	Unclear	High	High	High	Low	Low	High	High
Lobastov, 2016[46]	Unclear	Unclear	Unclear	High	High	Low	High	High	High
Mahan, 2014[47]	Low	Unclear	Unclear	Unclear	High	Low	Unclear	Unclear	High
Mlaver, 2020[48]	Unclear	Unclear	Unclear	Unclear	High	Unclear	Unclear	Unclear	High
Moumneh, 2020[49]	High	Unclear	Unclear	Low	High	Low	Low	High	High
Nafee, 2020[50]	Unclear	Low	Low	Low	Unclear	Low	Low	Unclear	Unclear
Nendaz, 2014[51]	Low	Unclear	Low	High	Low	Unclear	Low	High	Unclear
Pannucci, 2012[53]	High	Unclear	Unclear	High	High	High	Unclear	High	High
Pannucci, 2014[52]	Low	Unclear	High	High	High	Low	Low	High	High
Patell, 2017[54]	High	Unclear	Unclear	High	High	Unclear	Unclear	High	High
Rogers, 2007[56]	Unclear	Unclear	Unclear	High	Low	Unclear	Unclear	High	Unclear
Rogers, 2012[55]	High	High	Unclear	High	High	High	Unclear	High	High
Rosenberg, 2014[57]	Low	Unclear	Unclear	Unclear	Unclear	Unclear	Unclear	Unclear	Unclear
Rothberg, 2011[58]	High	Unclear	Unclear	High	Low	Unclear	Unclear	High	Unclear
Shaikh, 2016[59]	High	Unclear	High	High	High	Unclear	High	High	High
Shang, 2020[60]	Low	Unclear	Unclear	Unclear	High	Unclear	Unclear	Unclear	High

Shen, 2020[61]	Unclear	High	Unclear	Unclear	High	Unclear	Unclear	High	High
Tachino, 2019[62]	High	Unclear	Unclear	High	High	Unclear	Unclear	High	High
Tian, 2019[63]	High	Unclear	High	High	High	High	High	High	High
Vardi, 2013[64]	Unclear	Low	Low	High	High	Low	Low	High	High
Vaziri, 2018[65]	Unclear	Unclear	Unclear	High	High	Unclear	Unclear	High	High
Vincentelli, 2018[66]	High	Low	Unclear	High	High	Low	Unclear	High	High
Wang, 2020[67]	Low	Unclear	Unclear	Unclear	High	Unclear	Unclear	Unclear	High
Winoker, 2017[68]	High	Unclear	Unclear	High	High	High	High	High	High
Woller, 2011[69]	High	High	Unclear	High	Unclear	Unclear	Unclear	High	Unclear
Zhou 2014[71]	Unclear	Unclear	Unclear	High	High	Unclear	Unclear	High	High
Zhou, 2018[70]	Low	High	High	High	High	Unclear	Unclear	High	High

For peer review only

[INSERT FIGURE 2]

Predictive performance of VTE RAMs (summary of results)

As there were a reasonable number of studies to compare, a summary of the C-statistics for studies involving medical, surgical and trauma patients respectively is presented in **Figures 3a-c**, with the results grouped by RAM. Results of other hospital inpatients are presented in **online supplementary Appendix S4**. C-statistics varied markedly between these studies and between models, with no RAM performing obviously better than other models. In studies evaluating a single model, C-statistics[20] were sometimes weak (<0.7; 10 studies with 17 data points), often good (0.7-0.8; 17 studies with 20 data points) and a few were excellent (>0.8; 5 studies with 5 data points). There was marked heterogeneity between multiple studies evaluating the same model. Studies evaluating multiple (more than 3) models [31 37] tended to report weak accuracy across all the models (C-statistic <0.7; 2 studies with 16 data points).

[INSERT FIGURE 3]

Table 3 shows the sensitivity and specificity at various thresholds for studies involving medical, surgical and trauma patients respectively, with the results grouped by RAM. Interpretation was again limited by marked heterogeneity, which was exacerbated when different thresholds were reported by different studies evaluating the same model. Model accuracy was generally poor, with high sensitivity usually reflecting a threshold effect, as evidenced by corresponding low specificity (and vice versa).

For peer review only - http://bmjopen.bmj.com/site/about/guidelines.xhtml

Table 3. Sensitivity and specificity for studies involving medical, surgical and trauma inpatients

Risk assessment models	Threshold or cut-off	Endpoint	Data source	Sensitivity (95% CI)	Specificity (95% CI)
**MEDICAL INPATIENTS**					
**Caprini (7 studies)**	Risk Score ≥3	VTE	Lui, 2016[45]	70.9% (NR)	73.4% (NR)
	Risk Score ≥3	VTE	Moumneh, 2020[49]	98.1% (95.6, 99.4)	7.5% (7.1, 8.0)
	Risk Score ≥3	VTE	Zhou 2014[71]	82.3% (NR)	60.4% (NR)
	Risk Score ≥3	VTE	Zhou, 2018[70]	84.3% (NR)	66.2% (NR)
	Risk Score ≥5	VTE	Zhou, 2018[70]	57.1% (NR)	24.6% (NR)
	Risk score ≥5	VTE	Grant, 2016[36]	69.7% (NR)	50.28% (NR)
	Risk score ≥7	VTE	Grant, 2016[36]	42.69% (NR)	74.71% (NR)
	Risk score ≥9	VTE	Grant, 2016[36]	18.51% (NR)	89.03% (NR)
	NR[a]	VTE	de Bastos, 2016[32]	86.5% (NR)	47.0% (NR)
	NR	VTE	Cobben, 2019[31]	88.6% (NR)	21.4% (NR)
**Chopard (1 study)**	Risk Score ≥3	VTE	Vincentelli, 2018[66]	64.2% (38.4, 81.9)	57.7% (63.9, 79.4)
**Geneva models (4 studies)**	Risk Score ≥3	VTE	Blondon, 2018[28]; Nendaz, 2014[51]	All patients: 90.0% (73.5, 97.9)	All patients: 35.3% (32.8, 37.8)
				No prophylaxis: 85% (NR)	No prophylaxis: NR
	NR	VTE	Cobben, 2019[31]	75.0% (NR)	34.1% (NR)
	Simplified model: Risk Score ≥3	VTE	Blondon, 2019a[26]	95.0% (NR)	44.0% (NR)
	Simplified model: NR	VTE	Blondon, 2019b (abstract)[27]	86.4% (NR)	NR
**IMPROVE models (4 studies)**	4-factor model: NR	VTE	Cobben, 2019[31]	27.9% (NR)	85.4% (NR)
	7-factor model: Risk score ≥2	VTE	Moumneh, 2020[49]	73.8% (68.0, 79.0)	47.1% (46.3, 47.9)
	7-factor model: Risk score 2-3	VTE	Blondon, 2018[28]; Nendaz, 2014[51]	All patients: 87% (NR)	All patients: NR
				No prophylaxis: 85% (NR)	No prophylaxis: NR
	7-factor model: Risk score ≥3	VTE	Blondon, 2018[28]; Nendaz, 2014[51]	All patients: 73% (NR)	All patients: NR
				No prophylaxis: 54% (NR)	No prophylaxis: NR
	7-factor model: Risk score ≥4	VTE	Moumneh, 2020[49]	24.7% (19.6, 30.4)	85.5% (84.9, 86.1)
	7-factor model: NR	VTE	Cobben, 2019[31]	63.3% (NR)	70.7% (NR)
	NR	VTE	Blondon, 2019b (abstract)[27]	57.6% (NR)	NR
**Intermountain (1 study)**	NR	VTE	Cobben, 2019[31]	26.4% (NR)	90.2% (NR)
**Kucher (2 studies)**	Risk Score ≥4	VTE	Vincentelli, 2018[66]	25.1% (17.0, 55.1)	92.9% (81.0, 95.4)
	NR	VTE	Cobben, 2019[31]	28.0% (NR)	85.7% (NR)
**Lecumberri (1 study)**	NR	VTE	Cobben, 2019[31]	61.6% (NR)	46.3% (NR)
**NAVAL (1 study)**	NR	VTE	Cobben, 2019[31]	19.0% (NR)	92.7% (NR)
**NICE Guidelines (1 study)**	NR	VTE	Cobben, 2019[31]	77.6% (NR)	39.0% (NR)
**Padua (10 studies)**	Risk score ≥4	VTE	Barbar, 2010[24]	94.6% (NR)	62.0% (NR)
	Risk Score ≥4	VTE	Blondon, 2018[28]; Nendaz, 2014[51]	All patients: 73.3% (54.1, 87.7)	All patients: 51.9% (49.3, 54.5)
				No prophylaxis: 62% (NR)	No prophylaxis: NR
	Risk Score ≥4	VTE	Lui, 2016[45]	23.4% (NR)	85.6% (NR)
	Risk Score ≥4	VTE	Moumneh, 2020[49]	91.6% (87.6, 94.7)	25.6% (24.9, 26.3)
	Risk Score ≥4	VTE	Zhou 2014[71]	30.1% (NR)	12.7% (NR)
	Risk Score ≥4	VTE	Zhou, 2018[70]	49.1% (NR)	16.2% (NR)
	Risk Score ≥4	VTE	Vincentelli, 2018[66]	52.4% (38.4, 81.9)	72.3% (63.9, 79.4)
	Risk Score ≥4	VTE	Wang, 2020[67]	76.2% (NR)	61.6% (NR)
	NR	VTE	Blondon, 2019b (abstract)[27]	72.7% (NR)	NR
	NR	VTE	Cobben, 2019[31]	61.8% (NR)	48.8% (NR)
**PRETEMED Guidelines (1 study)**	NR	VTE	Cobben, 2019[31]	81.6% (NR)	24.4% (NR)
**Shen 2020 (1 study)**	NR	VTE	Shen, 2020[61]	77.8% (NR)	84.7% (NR)
**Zakai 2013 (1 study)**	Model 2: NR	VTE	Cobben, 2019[31]	63.8% (NR)	31.7% (NR)
**SURGICAL INPATIENTS**					
**Caprini (8 studies)**	Risk Score >5	VTE	Hachey, 2016[38]	100% (100, 100)	7.2% (4.1, 11.0)
	Risk Score ≥5	VTE	Mlaver, 2020[48]	88.9% (NR)	32.7% (NR)
	Risk Score >5	VTE	Shaikh, 2016[59]	70.8% (48.9, 87.4)	39.39% (37.0, 41.9)
	Youden index >5.5	VTE	Tian, 2019[63]	76.0% (NR)	64.0% (NR)

For peer review only - http://bmjopen.bmj.com/site/about/guidelines.xhtml

	Risk score >6	VTE	Frankel, 2017 (abstract)[35]	61.5% (NR)	59.8% (NR)
	Risk Score >6	VTE	Shaikh, 2016[59]	58.3% (36.6, 77.9)	60.1% (57.6, 62.5)
	Risk Score >7	VTE	Hachey, 2016[38]	100% (100, 100)	31.4% (25, 37.3)
	Risk Score >9	VTE	Hachey, 2016[38]	83.3% (58.3, 100)	60.5% (54.4, 67.3)
	Risk Score >9	VTE	Shaikh, 2016[59]	16.7% (NR)	93.3% (NR)
	Risk Score >10	VTE	Hachey, 2016[38]	75.0% (50, 100)	69.6% (64.6, 76.4)
	Risk score >10	VTE	Dornbus, 2018 (abstract)[33]	78.9% (NR)	60.9% (NR)
	Risk Score >10.5	DVT or PE	Lobastov, 2016[46]	95.0% (NR)	73.0% (NR)
	Risk Score >15 [b]	VTE	Hewes, 2015[40]	100% (100, 100)	66.7% (55.0, 78.3)
**Khorana (1 study)**	Youden index >0.5	VTE	Tian, 2019[63]	78.0% (NR)	48.0% (NR)
**Padua (2 studies)**	Risk Score ≥4	VTE	Mlaver, 2020[48]	61.1% (NR)	47.4% (NR)
	Youden index >3.5	VTE	Tian, 2019[63]	36.0% (NR)	93.0% (NR)
**Rogers 2007 (1 study)**	Youden index >14.5	VTE	Tian, 2019[63]	53.0% (NR)	54.0% (NR)
**TRAUMA PATIENTS**					
**RAP (2 studies)**	Risk score ≥5	VTE	Tachino, 2019[62]	100% (86.8, 100)	37.9% (34.6, 41.3)
	Risk score 5 to ≤ 14	DVT or PE	Hegsted, 2013[39]	•DVT: 82.0% (77, 87)<br>•PE: 71.0% (55, 86)	•DVT: 57.0% (55, 59)<br>•PE: 53.0% (51, 56)
	Risk score >14	DVT or PE	Hegsted, 2013[39]	•DVT: 15.0% (11, 20)<br>•PE: 12.0% (1, 23)	•DVT: 97.0% (97, 98)<br>•PE: 96.0% (95, 97)
**TESS (2 studies)**	Risk score ≥5	VTE	Rogers, 2012[55]	77.4% (NR)	75.6% (NR)
	Risk score <9	VTE	Ho, 2014[41]	•All VTE:<br>97.0% (91, 99)	•All VTE:<br>27.0% (22, 32)
	Risk score <9	VTE	Ho, 2014[41]	•Fatal and non-fatal PE: 97.0% (87, 99)	•Fatal and non-fatal PE: 24.0% (20, 29)
	Risk score <9	VTE	Ho, 2014[41]	•Fatal PE only:<br>100% (81, 100)	•Fatal PE only:<br>20.0% (13, 28)

[a] Paper states 'moderate and high risk'
[b] Modified Caprini model
CI, confidence interval; DVT, deep vein thrombosis; NR, not reported; PE, pulmonary embolism; RAP, Risk Assessment Profile; TESS, Trauma Embolic Scoring System VTE, venous thromboembolism

For peer review only

DISCUSSION

Summary of results

In this systematic review of 51 observational studies evaluating RAMs for predicting the risk of developing VTE in hospital inpatients, we found that VTE RAMs have generally weak predictive accuracy. The studies validating these models are heterogeneous and most have a high risk of bias. Lack of methodological clarity was common, leading to difficulty in assessing the applicability of the individual study results.

Interpretation of results

We were unable to undertake meta-analysis or statistical examination of the causes of the observed heterogeneity. Potential sources of heterogeneity include variation in study design, the study population, how RAMs are implemented, outcome definition and measurement, and the use of thromboprophylaxis. The latter point warrants further attention. Thromboprophylaxis was employed in about half (n=25) of the studies,[21 22 24 26 28 30 34 36-38 40 42 44 46 49-52 54 57-59 64 70 71] with the proportion receiving thromboprophylaxis ranging from 3.8%[42] to 100%.[46 50] It was not employed in three studies[32 61 63] and 23 studies[23 25 27 29 31 33 35 39 41 43 45 47 48 53 55 56 60 62 65-69] did not report on thromboprophylaxis use. The use of thromboprophylaxis may lead to underestimation of predictive accuracy if a given RAM were to predict VTE events that were subsequently prevented by thromboprophylaxis. Limited reporting of thromboprophylaxis use precludes further analysis of its impact upon the performance of the RAMs.

Comparison to the existing literature

The present review is the largest and most comprehensive systematic review in this field to date. It includes 18 recent studies[26-31 33 42 48-50 60-63 66 67 70] published since the completion of the previous systematic review.[10 12 13] These studies are consistent with the previous literature in that they report modest performance of the assessed RAMs, with limitations in methodology and reporting preventing further analysis. The conclusion of this review therefore concurs with previous systematic reviews: there is insufficient evidence to recommend one RAM over another.

For peer review only - http://bmjopen.bmj.com/site/about/guidelines.xhtml

Strengths and limitations

This systematic review has a number of strengths. The review was conducted with robust methodology in accordance with the PRISMA statement and the protocol was registered with the PROSPERO register. Clinical experts were involved throughout as checkers and to assess the validity and applicability of research during the review. We reported descriptive statistics to provide insight into the limited evidence base applicable to the subject matter, and the scientific concerns regarding validity of the data. However, there are a number of potential weaknesses. Decisions on study relevance, information gathering, and validity were un-blinded and could potentially have been influenced by pre-formed opinions. However, masking is resource intensive with uncertain benefits. The studies of risk prediction were a combination of prospective cohorts and retrospective health database registries. Both have significant limitations. Retrospective studies of health database registries may have large numbers but may be limited by poor data quality and failure to accurately ascertain outcomes. Prospective cohorts may have better quality data but with smaller numbers lack statistical power. The included studies demonstrated high levels of heterogeneity so we were unable to undertake any meta-analysis

Implications for policy, practice and future research

Guidelines from the American College of Chest Physicians (ACCP)[72 73] and the UK National Institute for Health and Care Excellence (NICE)[10] suggest using a validated RAM to guide the decision on whether to prescribe thromboprophylaxis. This review identifies all relevant RAMs and their validation studies. The reported results are insufficient to recommend one RAM over another. A RAM with weak predictive accuracy may still be better than no RAM at all but it is unclear whether RAMs predict VTE risk better than unstructured clinical assessment. Further research is clearly needed but routine use of thromboprophylaxis may present an insurmountable barrier to generating accurate and precise estimates of the prognostic accuracy of RAMs. The evidence that thromboprophylaxis is effective means that it is unethical to withhold thromboprophylaxis when a significant risk of VTE is identified. This inevitably reduces the number of VTE events in any study

For peer review only - http://bmjopen.bmj.com/site/about/guidelines.xhtml

and confounds the association between risk factors and VTE events. Further studies of RAM accuracy will add little to our review unless they can address this issue.

Alternative approaches therefore need to be considered. Decision-analytic modelling can use existing data to explore the trade-off between the benefits and harms of thromboprophylaxis and identify key uncertainties for future primary research. The data presented in our review show how well RAMs predict VTE but do not tell us the threshold score on the RAM at which thromboprophylaxis should be given to maximise prevention of VTE and minimise harm from bleeding. This may be a more important determinant of RAM effectiveness than predictive accuracy for VTE. Le *et al*.[74] suggest thromboprophylaxis is beneficial and cost-effective if a patient's VTE risk exceeds 1%. Further work to improve RAMs to help stratify the risk of VTE in different types of hospitalised patients could focus on using decision-analytic modelling to compare the effects, harms and costs of giving thromboprophylaxis to patients with varying risk of VTE. This would allow determination of the risk threshold at which thromboprophylaxis provides optimal overall benefit.

Findings from decision-analytic modelling would require validation through primary research. The limitations of undertaking accuracy studies in populations where thromboprophylaxis is routinely used mean that future research should focus on research that compares the effectiveness of different risk assessment approaches. Observational studies could draw upon variation in practice to compare outcomes between different risk assessment methods. Alternatively, a controlled trial could compare risk assessment methods in low risk patients where existing evidence (synthesised using decision-analytic modelling) suggests the benefits of thromboprophylaxis are uncertain.

CONCLUSIONS

We identified a number of validated RAMs for potential risk stratification of hospitalised inpatients. The available evidence is insufficient to recommend one over another.

For peer review only - http://bmjopen.bmj.com/site/about/guidelines.xhtml

Acknowledgements

The authors would like to thank all additional members of the core project group for NIHR HTA 127454 for input and commentary throughout the work. We are also indebted to Helen Shulver for assistance with logistics and administration.

Competing interest

None to declare

Source of funding

This study was funded by the United Kingdom National Institute for Health Research Health Technology Assessment Programme (project number 127454). The views expressed in this report are those of the authors and not necessarily those of the NIHR HTA Programme. Any errors are the responsibility of the authors. The funders had no role in the study design, in the collection, analysis and interpretation of data; in the writing of the manuscript; and in the decision to submit the manuscript for publication.

Data sharing statement

All data relevant to the study are included in the article or uploaded as supplementary information.

Author contributions

AP co-ordinated the study. SG, DH, AP, XG, MH, BH, KW were responsible for conception, design and obtaining funding for the study. MC developed the search strategy, undertook searches, and organised retrieval of papers. AP, KS, MT and SG were responsible for the acquisition, analysis and interpretation of data. SG, MT, DH, XG, MH, BH, KW helped interpret and provided a methodological, policy and clinical perspective on the data. AP, MT and SG were responsible for the drafting of this paper, although all authors provided comments on the drafts, read and approved the final version. AP is the guarantor for the paper.

For peer review only

For peer review only - http://bmjopen.bmj.com/site/about/guidelines.xhtml

Copyright licence statement

The project was funded by the United Kingdom National Institute for Health Research Health Technology Assessment Programme and as such is covered by Crown copyright. The Corresponding Author has the right to grant on behalf of all authors and does grant on behalf of all authors, **a non-exclusive license** on a worldwide basis to BMJ publishing (BMJ) and its Licensees to permit this article (if accepted) to be published by BMJ, as set out in our licence.

Ethics statement

Ethics approval and consent to participate: not applicable.

For peer review only

For peer review only - http://bmjopen.bmj.com/site/about/guidelines.xhtml

REFERENCES

1. Goldhaber SZ, Visani L, De Rosa M, et al. Acute ulmonary embolism: clinical outcomes in the International Cooperative Pulmonary Embolism Registry (ICOPER). *Lancet* 1999;353:1386–9.
2. Prandoni P, Lensing AW, Cogo A, et al. The longterm clinical course of acute deep venous thrombosis. *Ann Intern Med* 1996;125:1-7.
3. ISTH Steering Committee for World Thrombosis Day. Thrombosis: a major contributor to the global disease burden. *J Thromb Haemost* 2014;12
4. Jha AK, Larizgoitia I, Audera-Lopez C, et al. The global burden of unsafe medical care: analytic modelling of observational studies. *BMJ Qual Saf* 2013;22:809-15.
5. Alikhan R, Bedenis R, Cohen AT. Heparin for the prevention of venous thromboembolism in acutely ill medical patients (excluding stroke and myocardial infarction). *Cochrane Database Syst Rev* 2014;2014(5):Cd003747. doi: 10.1002/14651858.CD003747.pub4 [published Online First: 2014/05/09]
6. Dentali F, Douketis JD, Gianni M, et al. Meta-analysis: anticoagulant prophylaxis to prevent symptomatic venous thromboembolism in hospitalized medical patients. *Ann Intern Med* 2007;146(4)
7. Kahn SR, Diendéré G, Morrison DR, et al. Effectiveness of interventions for the implementation of thromboprophylaxis in hospitalised patients at risk of venous thromboembolism: an updated abridged Cochrane systematic review and meta-analysis of randomised controlled trials. *BMJ Open* 2019;9(5):e024444. doi: 10.1136/bmjopen-2018-024444 [published Online First: 2019/05/28]
8. Lloyd NS, Douketis JD, Moinuddin I, et al. Anticoagulant prophylaxis to prevent asymptomatic deep vein thrombosis in hospitalized medical patients: a systematic review and meta-analysis. *J Thromb Haemost* 2008;6(3):405-14. doi: 10.1111/j.1538-7836.2007.02847.x [published Online First: 2007/11/23]
9. Henke PK, Kahn SR, Pannucci CJ, et al. Call to Action to Prevent Venous Thromboembolism in Hospitalized Patients: A Policy Statement From the American Heart Association. *Circulation* 2020;141(24):e914-e31. doi: 10.1161/cir.0000000000000769 [published Online First: 2020/05/08]
10. NICE. Venous Thromboembolism in Over 16s: Reducing the Risk of Hospital-Acquired Deep Vein Thrombosis or Pulmonary Embolism. NICE guideline [NG89]: National Institute for Health and Care Excellence, London, UK. https://www.nice.org.uk/guidance/NG89, 2018.
11. Chan NC, Gross PL, Weitz JI. Addressing the burden of hospital-related venous thromboembolism: the role of extended anticoagulant prophylaxis. *J Thromb Haemost* 2018;16(3):413-17. doi: 10.1111/jth.13942 [published Online First: 2018/02/27]

For peer review only - http://bmjopen.bmj.com/site/about/guidelines.xhtml

12. Huang W, Anderson FA, Spencer FA, et al. Risk assessment models for predicting venous thromboembolism among hospitalized non-surgical patients: a systematic review. *J Thromb Thrombolysis* 2013;35(1):67-80. doi: 10.1007/s11239-012-0780-0 [published Online First: 2012/07/25]

13. Stuck AK, Spirk D, Schaudt J, et al. Risk assessment models for venous thromboembolism in acutely ill medical patients. A systematic review. *Thrombosis and Haemostasis* 2017;117(4):801-08. doi: https://dx.doi.org/10.1160/TH16-08-0631

14. Moher D, Liberati A, Tetzlaff J, et al. Preferred reporting items for systematic reviews and meta-analyses: the PRISMA statement. *Ann Intern Med* 2009;151(4):264-9, w64. doi: 10.7326/0003-4819-151-4-200908180-00135 [published Online First: 2009/07/23]

15. Goodacre S, Hogg K, Griffin X, et al. The cost-effectiveness of venous thromboembolism risk assessment tools for hospital inpatients. UK: National Institute of Health Research, 2019.

16. Moons KGM, Wolff RF, Riley RD, et al. PROBAST: A Tool to Assess Risk of Bias and Applicability of Prediction Model Studies: Explanation and Elaboration. *Ann Intern Med* 2019;170(1):W1-w33. doi: 10.7326/m18-1377 [published Online First: 2019/01/01]

17. Wolff RF, Moons KGM, Riley RD, et al. PROBAST: A Tool to Assess the Risk of Bias and Applicability of Prediction Model Studies. *Ann Intern Med* 2019;170(1):51-58. doi: 10.7326/m18-1376 [published Online First: 2019/01/01]

18. Centre for Reviews and Dissemination. Systematic Reviews: CRD's Guidance for Undertaking Reviews in Health Care: York: Centre for Reviews and Dissemination, 2009.

19. McKenzie JE, Brennan SE, Ryan RE, et al. Chapter 9: Summarizing study characteristics and preparing for synthesis. Cochrane Handbook for Systematic Reviews of Interventions version 6.2 (updated February 2021) [Internet]: Cochrane, 2021.

20. Hosmer DW, Lemeshow S. Applied Logistic Regression (2nd Edition): John Wiley & Sons. New York, 2000.

21. Abdel-Razeq HN, Hijjawi SB, Jallad SG, et al. Venous thromboembolism risk stratification in medically-ill hospitalized cancer patients. A comprehensive cancer center experience. *Journal of Thrombosis and Thrombolysis* 2010;30(3):286-93. doi: 10.1007/s11239-010-0445-9

22. Autar R. The management of deep vein thrombosis: the autar DVT risk assessment scale re-visited. *Journal of Orthopaedic Nursing* 2003;7:114-24.

23. Bahl V, Hu HM, Henke PK, et al. A validation study of a retrospective venous thromboembolism risk scoring method. *Ann Surg* 2010;251(2):344-50. doi: 10.1097/SLA.0b013e3181b7fca6 [published Online First: 2009/09/26]

24. Barbar S, Noventa F, Rossetto V, et al. A risk assessment model for the identification of hospitalized medical patients at risk for venous thromboembolism: the Padua Prediction Score. *Journal of Thrombosis and Haemostasis* 2010;8(11):2450-57. doi: 10.1111/j.1538-7836.2010.04044.x

For peer review only - http://bmjopen.bmj.com/site/about/guidelines.xhtml

25. Bilimoria KY, Liu Y, Paruch JL, et al. Development and Evaluation of the Universal ACS NSQIP Surgical Risk Calculator: A Decision Aid and Informed Consent Tool for Patients and Surgeons. *Journal of the American College of Surgeons* 2013;217(5):833-42.e3. doi: https://doi.org/10.1016/j.jamcollsurg.2013.07.385

26. Blondon M, Righini M, Nendaz M, et al. External validation of the simplified Geneva risk assessment model for hospital-associated venous thromboembolism in the Padua cohort. *J Thromb Haemost* 2019:[Epub ahead of print]. doi: doi: 10.1111/jth.14688

27. Blondon M, Limacher A, Righini M, et al. Adequacy of hospital thromboprophylaxis and risk assessment models in the SWITCO65+ cohort. *Research and Practice in Thrombosis and Haemostasis* 2019;3(Supplement 1):760. doi: http://dx.doi.org/10.1002/rth2.12229

28. Blondon M, Spirk D, Kucher N, et al. Comparative Performance of Clinical Risk Assessment Models for Hospital-Acquired Venous Thromboembolism in Medical Patients. *Thrombosis and Haemostasis* 2018;118(1):82-89. doi: https://dx.doi.org/10.1160/TH17-06-0403

29. Bo H, Li Y, Liu G, et al. Assessing the Risk for Development of Deep Vein Thrombosis among Chinese Patients using the 2010 Caprini Risk Assessment Model: A Prospective Multicenter Study. *Journal of Atherosclerosis and Thrombosis* 2020;27(8):801-08. doi: https://dx.doi.org/10.5551/jat.51359

30. Chen X, Pan L, Deng H, et al. Risk Assessment in Chinese Hospitalized Patients Comparing the Padua and Caprini Scoring Algorithms. *Clinical and Applied Thrombosis/Hemostasis* 2018:1076029618797465. doi: https://dx.doi.org/10.1177/1076029618797465

31. Cobben MRR, Nemeth B, Lijfering WM, et al. Validation of risk assessment models for venous thrombosis in hospitalized medical patients. *Research and Practice in Thrombosis and Haemostasis* 2019;3(2):217-25. doi: http://dx.doi.org/10.1002/rth2.12181

32. de Bastos M, Barreto SM, Caiafa JS, et al. Derivation of a risk assessment model for hospital-acquired venous thrombosis: the NAVAL score. *Journal of Thrombosis and Thrombolysis* 2016;41(4):628-35. doi: http://dx.doi.org/10.1007/s11239-015-1277-4

33. Dornbos DL, Shah V, Priddy B, et al. Predicting venous thromboembolic complications following neurological surgery procedures. *Journal of Neurosurgery* 2018;128(4):55. doi: http://dx.doi.org/10.3171/2018.4.JNS.AANS2018abstracts

34. Elias P, Khanna R, Dudley A, et al. Automating Venous Thromboembolism Risk Calculation Using Electronic Health Record Data upon Hospital Admission: The Automated Padua Prediction Score. *Journal of Hospital Medicine* 2017;12(4):231-37. doi: https://dx.doi.org/10.12788/jhm.2714

35. Frankel JK, Belanger M, Tortora J, et al. Caprini score predicts venous thromboembolic events in patients undergoing robotic assisted prostatectomy. *Journal of Urology* 2017;197(4 Supplement 1):e635.

For peer review only - http://bmjopen.bmj.com/site/about/guidelines.xhtml

36. Grant PJ, Greene MT, Chopra V, et al. Assessing the Caprini Score for Risk Assessment of Venous Thromboembolism in Hospitalized Medical Patients Results presented at: The Society of Hospital Medicine Annual Meeting, March 26, 2014, Las Vegas, Nevada. *American Journal of Medicine* 2016;129(5):528-35. doi: http://dx.doi.org/10.1016/j.amjmed.2015.10.027

37. Greene MT, Spyropoulos AC, Chopra V, et al. Validation of Risk Assessment Models of Venous Thromboembolism in Hospitalized Medical Patients. *American Journal of Medicine* 2016;129(9):1001. doi: http://dx.doi.org/10.1016/j.amjmed.2016.03.031

38. Hachey KJ, Hewes PD, Porter LP, et al. Caprini venous thromboembolism risk assessment permits selection for postdischarge prophylactic anticoagulation in patients with resectable lung cancer. *The Journal of Thoracic and Cardiovascular Surgery* 2016;151(1):37-44.e1. doi: https://doi.org/10.1016/j.jtcvs.2015.08.039

39. Hegsted D, Gritsiouk Y, Schlesinger P, et al. Utility of the risk assessment profile for risk stratification of venous thrombotic events for trauma patients. *The American Journal of Surgery* 2013;205(5):517-20. doi: https://doi.org/10.1016/j.amjsurg.2013.01.022

40. Hewes PD, Hachey KJ, Zhang XW, et al. Evaluation of the Caprini Model for Venothromboembolism in Esophagectomy Patients. *The Annals of Thoracic Surgery* 2015;100(6):2072-78. doi: https://doi.org/10.1016/j.athoracsur.2015.05.098

41. Ho KM, Rao S, Rittenhouse KJ, et al. Use of the Trauma Embolic Scoring System (TESS) to predict symptomatic deep vein thrombosis and fatal and non-fatal pulmonary embolism in severely injured patients. *Anaesth Intensive Care* 2014;42(6):709-14. doi: 10.1177/0310057x1404200605 [published Online First: 2014/10/25]

42. Hu Y, Li X, Zhou H, et al. Comparison between the Khorana prediction score and Caprini risk assessment models for assessing the risk of venous thromboembolism in hospitalized patients with cancer: a retrospective case control study. *Interactive Cardiovascular and Thoracic Surgery* 2020;31(4):454-60. doi: https://dx.doi.org/10.1093/icvts/ivaa137

43. Krasnow R, Preston M, Chung B, et al. Validation of venous thromboembolism risk assessment score in major urologic cancer surgery: A population based study. *Journal of Urology* 2017;197(4 Supplement 1):e1126.

44. Liu L-P, Zheng H-G, Wang DZ, et al. Risk Assessment of Deep-Vein Thrombosis After Acute Stroke: a Prospective Study Using Clinical Factors. *CNS Neuroscience & Therapeutics* 2014;20(5):403-10. doi: 10.1111/cns.12227

45. Liu X, Liu C, Chen X, et al. Comparison between Caprini and Padua risk assessment models for hospitalized medical patients at risk for venous thromboembolism: A retrospective study. *Interactive Cardiovascular and Thoracic Surgery* 2016;23(4):538-43. doi: http://dx.doi.org/10.1093/icvts/ivw158

For peer review only - http://bmjopen.bmj.com/site/about/guidelines.xhtml

46. Lobastov K, Barinov V, Schastlivtsev I, et al. Validation of the Caprini risk assessment model for venous thromboembolism in high-risk surgical patients in the background of standard prophylaxis. *Journal of Vascular Surgery: Venous and Lymphatic Disorders* 2016;4(2):153-60. doi: https://doi.org/10.1016/j.jvsv.2015.09.004

47. Mahan CE, Liu Y, Turpie AG, et al. External validation of a risk assessment model for venous thromboembolism in the hospitalised acutely-ill medical patient (VTE-VALOURR). *Thromb Haemost* 2014;112(4):692-9. doi: 10.1160/th14-03-0239 [published Online First: 2014/07/06]

48. Mlaver E, Lynde GC, Gallion C, et al. Development of a Novel Preoperative Venous Thromboembolism Risk Assessment Model. *The American Surgeon* 2020;86(9):1098-105. doi: https://dx.doi.org/10.1177/0003134820943556

49. Moumneh T, Riou J, Douillet D, et al. Validation of risk assessment models predicting venous thromboembolism in acutely ill medical inpatients: A cohort study. *Journal of Thrombosis and Haemostasis : JTH* 2020;18(6):1398-407. doi: https://dx.doi.org/10.1111/jth.14796

50. Nafee T, Gibson CM, Travis R, et al. Machine learning to predict venous thrombosis in acutely ill medical patients. *Research and Practice in Thrombosis and Haemostasis* 2020;4(2):230-37. doi: https://dx.doi.org/10.1002/rth2.12292

51. Nendaz M, Spirk D, Kucher N, et al. Multicentre validation of the Geneva Risk Score for hospitalised medical patients at risk of venous thromboembolism. Explicit ASsessment of Thromboembolic RIsk and Prophylaxis for Medical PATients in SwitzErland (ESTIMATE). *Thromb Haemost* 2014;111(3):531-8. doi: 10.1160/th13-05-0427 [published Online First: 2013/11/15]

52. Pannucci CJ, Laird S, Dimick JB, et al. A Validated Risk Model to Predict 90-Day VTE Events in Postsurgical Patients. *Chest* 2014;145(3):567-73. doi: https://doi.org/10.1378/chest.13-1553

53. Pannucci CJ, Osborne NH, Wahl WL. Creation and validation of a simple venous thromboembolism risk scoring tool for thermally injured patients: analysis of the National Burn Repository. *J Burn Care Res* 2012;33(1):20-25. doi: 10.1097/BCR.0b013e318234d8b5

54. Patell R, Rybicki L, McCrae KR, et al. Predicting risk of venous thromboembolism in hospitalized cancer patients: Utility of a risk assessment tool. *American Journal of Hematology* 2017;92(6):501-07. doi: https://dx.doi.org/10.1002/ajh.24700

55. Rogers FB, Shackford SR, Horst MA, et al. Determining venous thromboembolic risk assessment for patients with trauma: the Trauma Embolic Scoring System. *J Trauma Acute Care Surg* 2012;73(2):511-5. [published Online First: 2012/09/29]

56. Rogers SO Jr, Kilaru RK, Hosokawa P, et al. Multivariable predictors of postoperative venous thromboembolic events after general and vascular surgery: results from the patient safety in surgery study. *J Am Coll Surg* 2007;204(6):1211-21.

57. Rosenberg D, Eichorn A, Alarcon M, et al. External validation of the risk assessment model of the International Medical Prevention Registry on Venous Thromboembolism (IMPROVE) for

For peer review only - http://bmjopen.bmj.com/site/about/guidelines.xhtml

medical patients in a tertiary health system. *Journal of the American Heart Association* 2014;3(6):e001152-e52. doi: 10.1161/JAHA.114.001152

58. Rothberg MB, Lindenauer PK, Lahti M, et al. Risk factor model to predict venous thromboembolism in hospitalized medical patients. *J Hosp Med* 2011;6(4):202-9. doi: 10.1002/jhm.888 [published Online First: 2011/04/12]

59. Shaikh M-A, Jeong HS, Mastro A, et al. Analysis of the American Society of Anesthesiologists Physical Status Classification System and Caprini Risk Assessment Model in Predicting Venous Thromboembolic Outcomes in Plastic Surgery Patients. *Aesthetic Surgery Journal* 2016;36(4):497-505. doi: 10.1093/asj/sjv198

60. Shang M-M, Yan R, Wang X-L, et al. Comparison of 2013 and 2009 versions of Caprini risk assessment models for predicting VTE in Chinese cancer patients: a retrospective study. *Journal of Thrombosis and Thrombolysis* 2020;50(2):446-51. doi: https://dx.doi.org/10.1007/s11239-020-02038-2

61. Shen C, Ge B, Liu X, et al. Predicting the occurrence of venous thromboembolism: construction and verification of risk warning model. *BMC Cardiovascular Disorders* 2020;20(1):249. doi: https://dx.doi.org/10.1186/s12872-020-01519-9

62. Tachino J, Yamamoto K, Shimizu K, et al. Quick risk assessment profile (qRAP) is a prediction model for post-traumatic venous thromboembolism. *Injury* 2019;50(9):1540-44. doi: https://dx.doi.org/10.1016/j.injury.2019.06.020

63. Tian B, Li H, Cui S, et al. A novel risk assessment model for venous thromboembolism after major thoracic surgery: a Chinese single-center study. *Journal of Thoracic Disease* 2019;11(5):1903-10. doi: https://dx.doi.org/10.21037/jtd.2019.05.11

64. Vardi M, Ghanem-Zoubi NO, Zidan R, et al. Venous thromboembolism and the utility of the Padua Prediction Score in patients with sepsis admitted to internal medicine departments. *Journal of Thrombosis and Haemostasis* 2013;11(3):467-73. doi: 10.1111/jth.12108

65. Vaziri S, Wilson J, Abbatematteo J, et al. Predictive performance of the American College of Surgeons universal risk calculator in neurosurgical patients. *Journal of Neurosurgery* 2018;128(3):942-47. doi: https://dx.doi.org/10.3171/2016.11.JNS161377

66. Vincentelli GM, Timpone S, Murdolo G, et al. A new risk assessment model for the stratification of the thromboembolism risk in medical patients: the TEVere Score. *Minerva Medica* 2018;109(6):436-42. doi: https://dx.doi.org/10.23736/S0026-4806.18.05689-6

67. Wang X, Yang Y-Q, Liu S-H, et al. Comparing different venous thromboembolism risk assessment machine learning models in Chinese patients. *Journal of Evaluation in Clinical Practice* 2020;26(1):26-34. doi: https://dx.doi.org/10.1111/jep.13324

68. Winoker JS, Paulucci DJ, Anastos H, et al. Predicting Complications Following Robot-Assisted Partial Nephrectomy with the ACS NSQIP Universal Surgical Risk Calculator. *The Journal of Urology* 2017;198(4):803-09. doi: https://dx.doi.org/10.1016/j.juro.2017.04.021

For peer review only - http://bmjopen.bmj.com/site/about/guidelines.xhtml

69. Woller SC, Stevens SM, Jones JP, et al. Derivation and Validation of a Simple Model to Identify Venous Thromboembolism Risk in Medical Patients. *The American Journal of Medicine* 2011;124(10):947-54.e2. doi: https://doi.org/10.1016/j.amjmed.2011.06.004

70. Zhou H, Hu Y, Li X, et al. Assessment of the Risk of Venous Thromboembolism in Medical Inpatients using the Padua Prediction Score and Caprini Risk Assessment Model. *Journal of Atherosclerosis and Thrombosis* 2018;25(11):1091-104. doi: https://dx.doi.org/10.5551/jat.43653

71. Zhou H, Wang L, Wu X, et al. Validation of a Venous Thromboembolism Risk Assessment Model in Hospitalized Chinese Patients: A Case-Control Study. *Journal of Atherosclerosis and Thrombosis* 2014;21(3):261-72. doi: 10.5551/jat.20891

72. Kahn SR, Lim W, Dunn AS, et al. Prevention of VTE in nonsurgical patients: Antithrombotic Therapy and Prevention of Thrombosis, 9th ed: American College of Chest Physicians Evidence-Based Clinical Practice Guidelines. *Chest* 2012;141:e195S-e226S.

73. Gould MK, Garcia DA, Wren SM, et al. Prevention of VTE in nonorthopedic surgical patients: Antithrombotic Therapy and Prevention of Thrombosis, 9th ed: American College of Chest Physicians Evidence-Based Clinical Practice Guidelines. *Chest* 2012;141:e227S-e77S.

74. Le P, Martinez KA, Pappas MA, et al. A decision model to estimate a risk threshold for venous thromboembolism prophylaxis in hospitalized medical patients. *J Thromb Haemost* 2017;15(6):1132-41.

For peer review only

For peer review only - http://bmjopen.bmj.com/site/about/guidelines.xhtml

Figure legends

**Figure 1**	**Study flow chart**
**Figure 2**	**PROBAST assessment summary graph - Review authors' judgements**
**Figure 3**	**C-statistics by model for studies involving a) medical b) surgical and c) trauma inpatients**

For peer review only

For peer review only - http://bmjopen.bmj.com/site/about/guidelines.xhtml

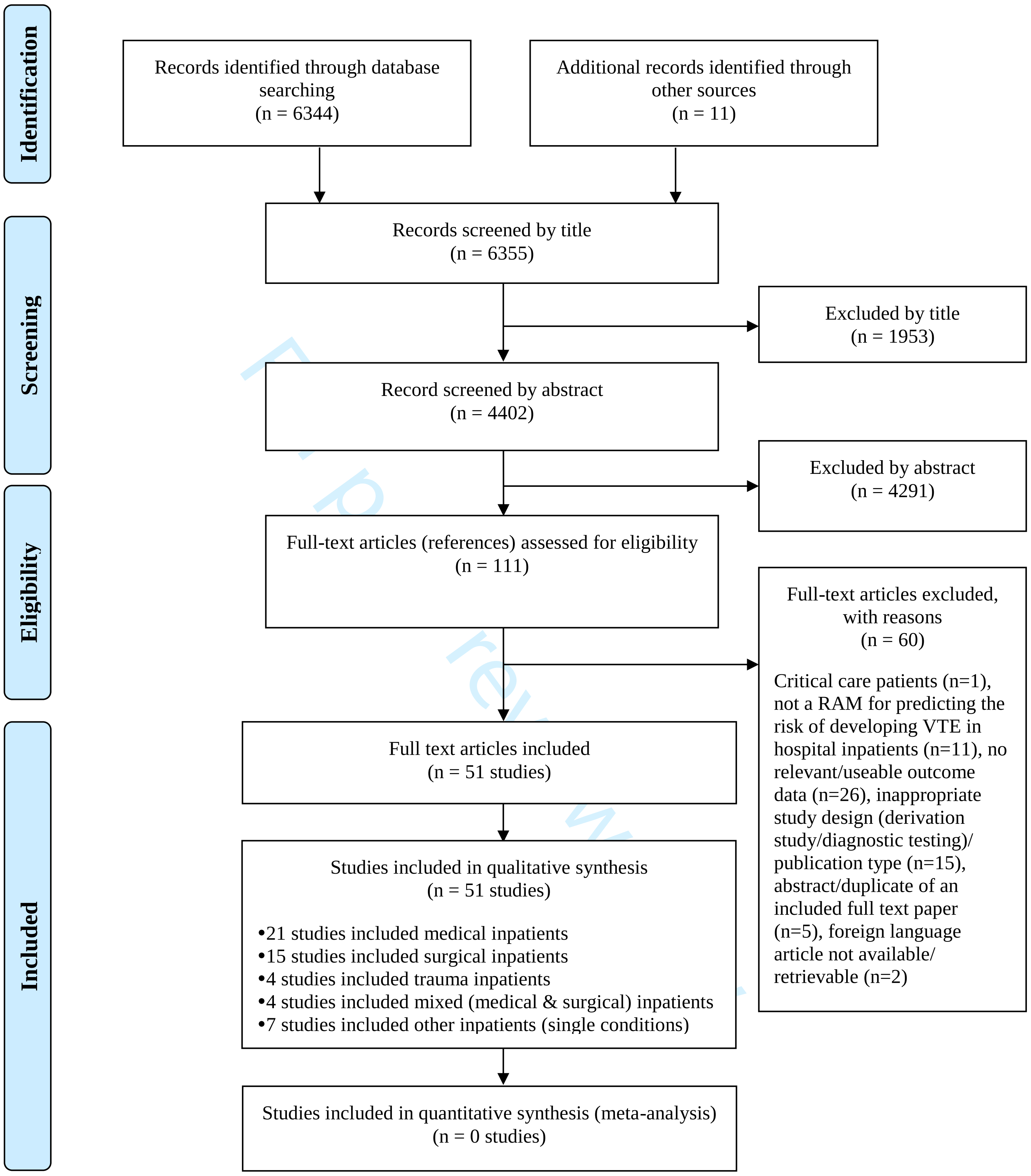

Identification
Screening
Eligibility
Included
Records identified through database searching (n = 6344)
Additional records identified through other sources (n = 11)
Records screened by title (n = 6355)
Excluded by title (n = 1953)
Record screened by abstract (n = 4402)
Excluded by abstract (n = 4291)
Full-text articles (references) assessed for eligibility (n = 111)
Full-text articles excluded, with reasons (n = 60)
Critical care patients (n=1), not a RAM for predicting the risk of developing VTE in hospital inpatients (n=11), no relevant/useable outcome data (n=26), inappropriate study design (derivation study/diagnostic testing)/ publication type (n=15), abstract/duplicate of an included full text paper (n=5), foreign language article not available/ retrievable (n=2)
Full text articles included (n = 51 studies)
Studies included in qualitative synthesis (n = 51 studies)
•21 studies included medical inpatients
•15 studies included surgical inpatients
•4 studies included trauma inpatients
•4 studies included mixed (medical & surgical) inpatients
•7 studies included other inpatients (single conditions)
Studies included in quantitative synthesis (meta-analysis) (n = 0 studies)

Figure 1

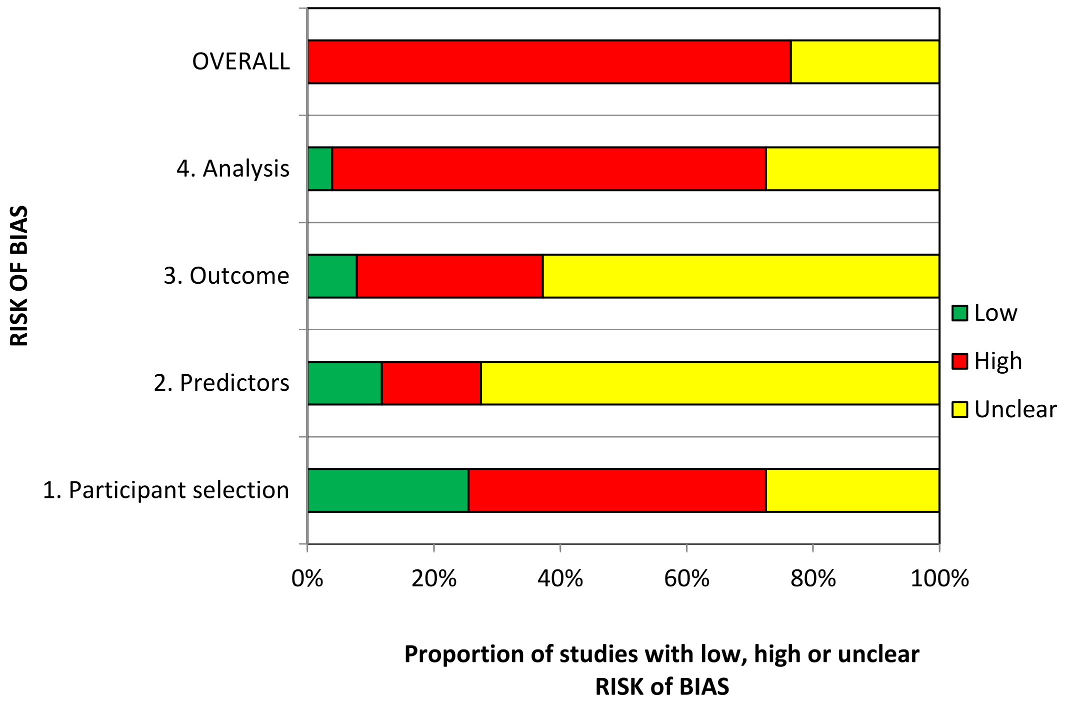
RISK OF BIAS
OVERALL
4. Analysis
3. Outcome
2. Predictors
1. Participant selection
0%
20%
40%
60%
80%
100%
Low
High
Unclear
Proportion of studies with low, high or unclear RISK of BIAS

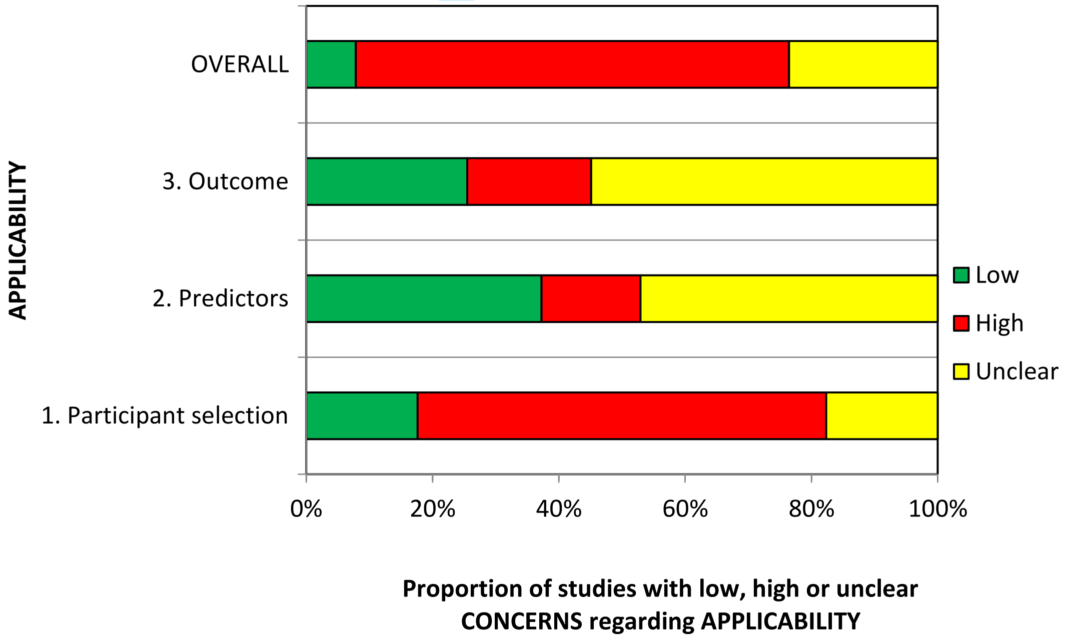
APPLICABILITY
OVERALL
3. Outcome
2. Predictors
1. Participant selection
0%
20%
40%
60%
80%
100%
Low
High
Unclear
Proportion of studies with low, high or unclear CONCERNS regarding APPLICABILITY

Figure 2

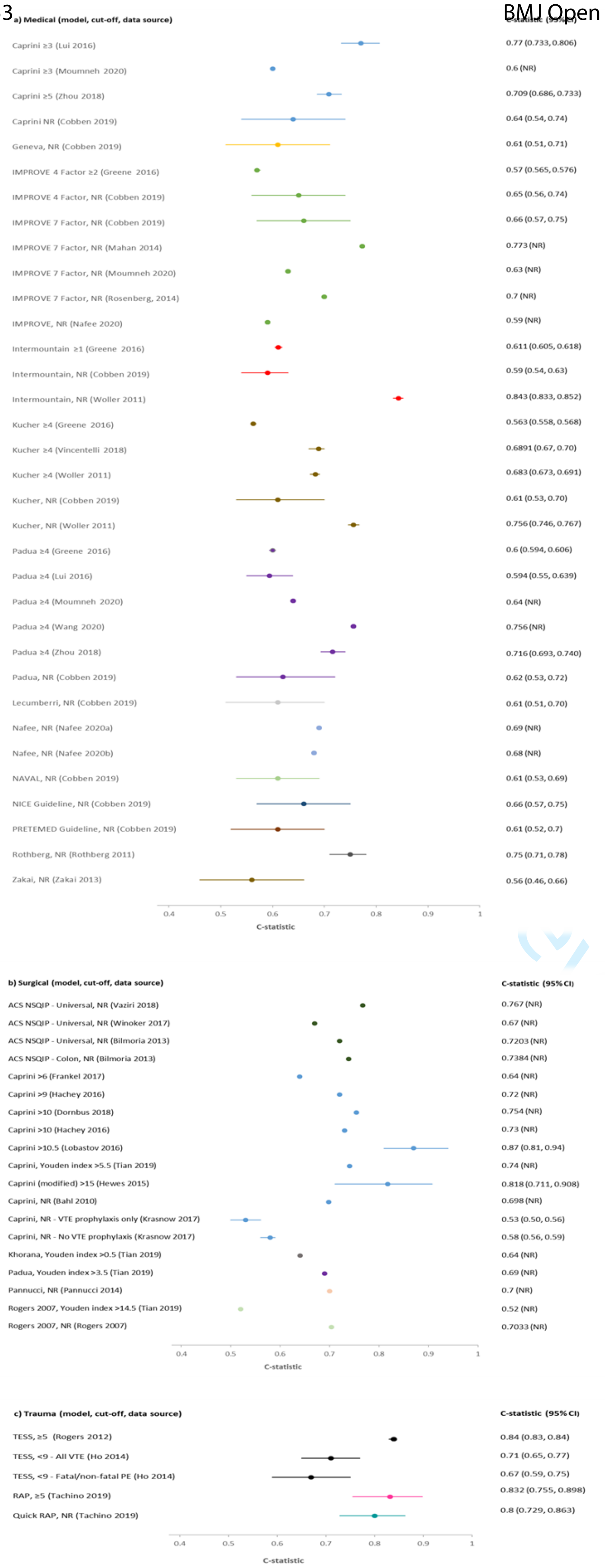

ACS NSQIP, American College of Surgeons National Surgical Quality Improvement Program; CI, confidence interval; DVT, deep vein thrombosis; NR, not reported; PE, pulmonary embolism; RAP, Risk Assessment Profile; TESS, Trauma Embolic Scoring System VTE, venous thromboembolism

Figure 3

ONLINE SUPPLEMENTARY

APPENDIX S1: LITERATURE SEARCH STRATEGY

Table S1 **Literature search strategy for the review of RAMs for VTE in hospital inpatients**

**Database searched:**	**Ovid MEDLINE(R) Epub Ahead of Print, In-Process & Other Non-Indexed Citations, Ovid MEDLINE(R) Daily, Ovid MEDLINE and Versions(R)**
**Platform or provider used:**	**Ovid SP**
**Date of coverage:**	**1946 to February 2021**
**Search undertaken:**	**February 2021**

1 pulmonary embolism/ or thromboembolism/ or venous thromboembolism/ or venous thrombosis/ or upper extremity deep vein thrombosis/
2 (((venous or vein) adj (thrombosis or thromboses or thrombus or thromboembolism)) or (dvt or vte) or ((pulmonary or lung) adj3 (embolism or emboli or embolus or emboliz* or thromboembolism))).ti,ab.
3 1 or 2
4 letter/ or editorial/ or news/ or exp historical article/ or anecdotes as topic/ or comment/ or case report/ or (letter or comment).ti.
5 randomized controlled trial/ or random*.ti,ab.
6 4 not 5
7 animals/ not humans/
8 exp animals, laboratory/
9 exp animal experimentation/
10 exp models, animal/
11 exp rodentia/
12 (rat or rats or mouse or mice).ti.
13 6 or 7 or 8 or 9 or 10 or 11 or 12
14 3 not 13
15 (risk* adj2 assess*).ti,ab.
16 ((score* or scoring) adj2 (tool* or system*)).ti,ab.
17 ((risk* or predict* or prognos*) adj4 (tool* or rule* or index* or indices or score* or scoring or scale* or model* or system* or algorithm* or stratif* or criteria or calculat*)).ti,ab.
18 (vienna adj5 cats).ti,ab.
19 (vienna cancer and thrombosis study).ti,ab.
20 trauma embolic scoring.ti,ab.
21 tess.ti,ab.
22 (roger* or caprini* or kucher* or cohen* or padua* or khorana* or autar).ti,ab.
23 (well* adj2 (score* or scoring)).ti,ab.
24 department of health.ti,ab,au.
25 or/15-24
26 14 and 25
27 limit 26 to yr="2017 -Current"

For peer review only

**Databases searched:**	**EMBASE**
**Platform or provider used:**	**Ovid SP**
**Date of coverage:**	**1974 to February 2021**
**Search undertaken:**	**February 2021**

1 thromboembolism/ or venous thromboembolism/ or vein thrombosis/ or deep veinthrombosis/ or leg thrombosis/ or lower extremity deep vein thrombosis/ or postoperativethrombosis/ or lung embolism/ or upper extremity deep vein thrombosis/
2 (((venous or vein) adj (thrombosis or thromboses or thrombus or thromboembolism)) or (dvt or vte) or ((pulmonary or lung) adj3 (embolism or emboli or embolus or emboliz* or thromboembolism))).ti,ab.
3 1 or 2
4 letter.pt. or letter/ or note.pt. or editorial.pt. or case report/ or case study/ or (letter or comment*).ti.
5 randomized controlled trial/ or random*.ti,ab.
6 4 not 5
7 animal/ not human/
8 nonhuman/
9 exp animal experiment/ or exp experimental animal/
10 animal model/
11 exp rodent/
12 (rat or rats or mouse or mice).ti.
13 6 or 7 or 8 or 9 or 10 or 11 or 12
14 3 not 13
15 (risk* adj2 assess*).ti,ab.
16 ((score* or scoring) adj2 (tool* or system*)).ti,ab.
17 ((risk* or predict* or prognos*) adj4 (tool* or rule* or index* or indices or score* or scoring or scale* or model* or system* or algorithm* or stratif* or criteria or calculat*)).ti,ab.
18 (vienna adj5 cats).ti,ab.
19 (vienna cancer and thrombosis study).ti,ab.
20 trauma embolic scoring.ti,ab.
21 tess.ti,ab.
22 (roger* or caprini* or kucher* or cohen* or padua* or khorana* or autar).ti,ab.
23 (well* adj2 (score* or scoring)).ti,ab.
24 department of health.ti,ab,au.
25 or/15-24
26 14 and 25
27 limit 26 to yr="2017 -Current"

For peer review only

**Databases searched:**	**Cochrane CENTRAL Register of Randomised Controlled Trials & Cochrane Database of Systematic Reviews**
**Platform or provider used:**	**www.thecochranelibrary.com**
**Date of coverage:**	**Inception to February 2021**
**Search undertaken:**	**February 2021**

#1. MeSH descriptor: [venous thromboembolism] this term only

#2. MeSH descriptor: [pulmonary embolism] this term only

#3. MeSH descriptor: [venous thrombosis] this term only

#4. MeSH descriptor: [thromboembolism] this term only

#5. MeSH descriptor: [upper extremity deep vein thrombosis] this term only

#6. ((*venous or *vein) next (thrombosis or thromboses or thrombus or thromboembolism) or dvt or vte or (pulmonary or lung) near/3 (embolism or emboli or embolus or emboliz* or thromboembolism)):ti,ab

#7. #1 or #2 or #3 or #4 or #6

#8. (risk* near/2 assess*):ti,ab

#9. ((score* or scoring) near/2 (tool* or system*)):ti,ab

#10. ((risk* or predict* or prognos*) near/4 (tool* or rule* or index* or indices or score* or scoring or scale* or model* or system* or algorithm* or stratif* or criteria or calculat*)):ti,ab

#11. (vienna near/5 cats):ti,ab

#12. (vienna cancer and thrombosis study):ti,ab

#13. trauma embolic scoring:ti,ab

#14. tess:ti,ab

#15. (roger* or caprini* or kucher* or cohen* or padua* or khorana* or autar):ti,ab

#16. (well* near/2 (score* or scoring)):ti,ab

#17. (department of health):ti,ab

#18. (or #8-#17)

#19. #7 and #18 with Publication Year from 2017 to 21

For peer review only

APPENDIX S2: LIST OF EXCLUDED STUDIES WITH RATIONALE

	Authors, year	Reason for exclusion
1.	Alikhan et al., 2004 [1]	Derivation only
2.	Alper et al., 2018 [2]	Not a RAM for predicting the risk of developing VTE in hospital inpatients
3.	Arcelus et al., 1991 [3]	No relevant/useable outcome data
4.	Arpaia et al., 2020 [4]	Not a RAM for predicting the risk of developing VTE in hospital inpatients
5.	Bagot et al., 2010 [5]	No relevant/useable outcome data
6.	Bellizzi et al., 2018 [6]	No relevant/useable outcome data
7.	Blondon et al., 2020 [7]	Duplicate of included study (Blondon et al., 2019a)
8.	Blondon et al., 2017 [8]	Abstract of an included full-text study
9.	Caprini et al., 2001 [9]	Review
10.	Chen et al., 2018 [10]	Not available (foreign language)
11.	Chopard et al., 2006 [11]	No relevant/useable outcome data
12.	Coelho et al., 2020 [12]	Not a RAM for predicting the risk of developing VTE in hospital inpatients
13.	Dang et al., 2019 [13]	Derivation only
14.	Davis and Intagliata, 2018 [14]	No relevant/useable outcome data
15.	Depietri et al., 2018 [15]	No relevant/useable outcome data
16.	Ellis et al., 2019 [16]	No relevant/useable outcome data
17.	Fadoi Foundation, 2020 [17]	Trial protocol (no results)
18.	Ferreira et al., 2018 [18]	No relevant/useable outcome data
19.	Ferreira et al., 2017 [19]	No relevant/useable outcome data
20.	Fritz et al., 2021 [20]	No relevant/useable outcome data
21.	Gibson et al., 2017 [21]	RAM involves diagnostic testing
22.	Girardi et al., 2018 [22]	Not a RAM for predicting the risk of developing VTE in hospital inpatients
23.	Grzelak et al., 2019 [23]	Not a RAM for predicting the risk of developing VTE in hospital inpatients
24.	Hostler et al., 2016 [24]	No relevant/useable outcome data
25.	Hu, 2018 [25]	No relevant/useable outcome data
26.	Koren et al., 2017 [26]	Not a RAM for predicting the risk of developing VTE in hospital inpatients
27.	Kucher et al., 2005 [27]	No relevant/useable outcome data
28.	Lecumberri et al., 2008 [28]	No relevant/useable outcome data
29.	Luo and Zhang, 2017 [29]	Not available (foreign language)
30.	Maynard et al., 2010 [30]	No relevant/useable outcome data
31.	McCaffrey et al., 2007 [31]	No relevant/useable outcome data

For peer review only

32.	Meizoso et al., 2017 [32]	Derivation only
33.	Monti et al., 2019 [33]	Abstract of included full text paper
34.	Mull et al., 2017 [34]	Review
35.	Nafee et al., 2018 [35]	Abstract of included full text paper
36.	Nnadi et al., 2017 [36]	No relevant/useable outcome data
37.	Obi et al., 2015 [37]	Critical care patients
38.	Pannucci et al., 2017 [38]	Review
39.	Rafizadeh et al., 2016 [39]	No relevant/useable outcome data
40.	Rastogi et al., 2020 [40]	No relevant/useable outcome data
41.	Razak et al., 2019 [41]	Not a RAM for predicting the risk of developing VTE in hospital inpatients
42.	Robert-Ebadi et al., 2017 [42]	Not a RAM for predicting the risk of developing VTE in hospital inpatients
43.	Salim et al., 2018 [43]	No relevant/useable outcome data
44.	Samama et al., 2006 [44]	Expert opinion
45.	Shrotriya et al., 2018 [45]	Not a RAM for predicting the risk of developing VTE in hospital inpatients
46.	Smilg Nicolas et al., 2018 [46]	No relevant/useable outcome data
47.	Spirk et al., 2017 [47]	No relevant/useable outcome data
48.	Spyropoulos et al., 2020 [48]	RAM involves diagnostic testing
49.	Spyropoulos et al., 2011 [49]	Derivation only
50.	Stuck et al., 2017 [50]	Review
51.	Tadesse et al., 2020 [51]	No relevant/useable outcome data
52.	Taha et al., 2020 [52]	No relevant/useable outcome data
53.	Tung et al., 2020 [53]	No relevant/useable outcome data
54.	Veith et al., 2019 [54]	No relevant/useable outcome data
55.	Winoker et al., 2017 [55]	Abstract of an included full-text study
56.	Yale et al., 2005 [56]	Derivation only
57.	Ye et al., 2017 [57]	Review
58.	Zakai et al., 2013 [58]	Derivation only
59.	Zambelli et al., 2020 [59]	Not a RAM for predicting the risk of developing VTE in hospital inpatients
60.	Zhou et al., 2012 [60]	Not a RAM for predicting the risk of developing VTE in hospital inpatients

For peer review only

REFERENCES (APPENDIX S2)

1. Alikhan R, Cohen AT, Combe S, et al. Risk Factors for Venous Thromboembolism in Hospitalized Patients With Acute Medical Illness: Analysis of the MEDENOX Study. *JAMA Internal Medicine* 2004;164(9):963-68. doi: 10.1001/archinte.164.9.963
2. Alper EC, Ip IK, Balthazar P, et al. Risk Stratification Model: Lower-Extremity Ultrasonography for Hospitalized Patients with Suspected Deep Vein Thrombosis. *Journal of General Internal Medicine* 2018;33(1):21-25. doi: https://dx.doi.org/10.1007/s11606-017-4170-3
3. Arcelus JI, Candocia S, Traverso CI, et al. Venous thromboembolism prophylaxis and risk assessment in medical patients. *Semin Thromb Hemost* 1991;17:3313-8.
4. Arpaia GG, Caleffi A, Marano G, et al. Padua prediction score and IMPROVE score do predict in-hospital mortality in Internal Medicine patients. *Internal and Emergency Medicine* 2020;15(6):997-1003. doi: https://dx.doi.org/10.1007/s11739-019-02264-4
5. Bagot C, Gohil S, Perrott R, et al. The use of an exclusion-based risk-assessment model for venous thrombosis improves uptake of appropriate thromboprophylaxis in hospitalized medical patients. *QJM* 2010;103:597-605.
6. Bellizzi A, Palombi G, Bardi G, et al. Score Padua and IMPROVE: What changes in management of venous thromboembolism prophylaxis in Internal Medicine? *Italian Journal of Medicine* 2018;12(2 Supplement 1):2. doi: http://dx.doi.org/10.4081/itjm.2018.s2
7. Blondon M, Righini M, Nendaz M, et al. External validation of the simplified Geneva risk assessment model for hospital-associated venous thromboembolism in the Padua cohort. *Journal of Thrombosis and Haemostasis* 2020;18(3):676-80. doi: https://dx.doi.org/10.1111/jth.14688
8. Blondon M, Spirk D, Kucher N, et al. External validation and comparison of the improve risk assessment model with the Geneva risk assessment model in the estimate cohort. *Research and Practice in Thrombosis and Haemostasis* 2017;1(Supplement 1):180. doi: http://dx.doi.org/10.1002/rth2.12012
9. Caprini JA, Arcelus JI, Reyna J. Effective risk stratification of surgical and nonsurgical patients for venous thromboembolic disease. *Seminars in Hematology* 2001;38:12-19. doi: https://doi.org/10.1016/S0037-1963(01)90094-0
10. Chen XL, Pan L, Wang Y. [Validity of Padua risk assessment scale for assessing the risk of deep venous thrombosis in hospitalized patients]. *Zhonghua nei ke za zhi* 2018;57(7):514-17. doi: https://dx.doi.org/10.3760/cma.j.issn.0578-1426.2018.07.009
11. Chopard P, Spirk D, Bounameaux H, et al. Identifying acutely ill medical patients requiring thromboprophylaxis. *J Thromb Haemost* 2006;4(4):915-6.
12. Coelho J, Divernet-Queriaud M, Roy P-M, et al. Comparison of the Wells score and the revised Geneva score as a tool to predict pulmonary embolism in outpatients over age 65. *Thrombosis Research* 2020;196:120-26. doi: https://dx.doi.org/10.1016/j.thromres.2020.07.026

For peer review only

13. Dang JT, Switzer N, Delisle M, et al. Predicting venous thromboembolism following laparoscopic bariatric surgery: development of the BariClot tool using the MBSAQIP database. *Surgical Endoscopy* 2019;33(3):821-31. doi: https://dx.doi.org/10.1007/s00464-018-6348-0

14. Davis J, Intagliata NM. Practice patterns in application of venous thromboembolism prophylaxis to a hepatology inpatient service: A quality improvement project. *Hepatology* 2018;68(Supplement 1):296A. doi: http://dx.doi.org/10.1002/hep.30257

15. Depietri L, Marietta M, Scarlini S, et al. Clinical impact of application of risk assessment models (Padua Prediction Score and Improve Bleeding Score) on venous thromboembolism, major hemorrhage and health expenditure associated with pharmacologic VTE prophylaxis: a "real life" prospective and retrospective observational study on patients hospitalized in a Single Internal Medicine Unit (the STIME study). *Internal and Emergency Medicine* 2018;13(4):527-34. doi: https://dx.doi.org/10.1007/s11739-018-1808-z

16. Ellis N, Grubb C-M, Mustoe S, et al. Venous thromboembolism risk in psychiatric in-patients: a multicentre cross-sectional study. *BJPsych Bulletin* 2019:1-5. doi: https://dx.doi.org/10.1192/bjb.2019.25

17. Fadoi Foundation. Efficacy of the Use of Risk Scores in Reducing Important Clinical Outcomes in Hospitalized Medical Ill Patients, 2020. Available *https://clinicaltrialsgov/show/NCT04267718* [Accessed 2 March 2021]

18. Ferreira C, Avelino Mancini R, Monique Lana Diniz M, et al. Evaluation of inter-rater agreement of a risk assessment model for thromboprophylaxis in medical hospitalised patients: Preliminary results of a cohort study. *Research and Practice in Thrombosis and Haemostasis* 2018;2(Supplement 1):323-24. doi: http://dx.doi.org/10.1002/rth2.12125

19. Ferreira D, de Sousa JA, Felicissimo P, et al. Venous thromboembolism risk and prophylaxis in the Portuguese hospital care setting: The ARTE study. *Revista portuguesa de cardiologia : orgao oficial da Sociedade Portuguesa de Cardiologia (Portuguese Journal of Cardiology)* 2017;36(11):823-30. doi: https://dx.doi.org/10.1016/j.repc.2017.01.009

20. Fritz MK, Kincaid SE, Sargent CG, et al. Venous thromboembolism (VTE) risk stratification in general medical patients at an academic medical center. *Journal of Thrombosis and Thrombolysis* 2021;51(1):67-73. doi: https://dx.doi.org/10.1007/s11239-020-02144-1

21. Gibson CM, Spyropoulos AC, Cohen AT, et al. The IMPROVEDD VTE Risk Score: Incorporation of D-Dimer into the IMPROVE Score to Improve Venous Thromboembolism Risk Stratification. *TH Open* 2017;1(1):e56-e65. doi: https://dx.doi.org/10.1055/s-0037-1603929

22. Girardi AM, Bettiol RS, Garcia TS, et al. Wells and Geneva Scores Are Not Reliable Predictors of Pulmonary Embolism in Critically Ill Patients: A Retrospective Study. *Journal of Intensive Care Medicine* 2018:885066618816280. doi: https://dx.doi.org/10.1177/0885066618816280

For peer review only

23. Grzelak M, Javia V, Cox C, et al. Incidence and patient characteristics for the development of venous thromboembolic events within the adult burn center population and an evaluation of the caprini risk assessment model. *Journal of Burn Care and Research* 2019;40(Supplement 1):S57. doi: http://dx.doi.org/10.1093/jbcr/irz013.088

24. Hostler DC, Marx ES, Moores LK, et al. Validation of the international medical prevention registry on venous thromboembolism bleeding risk score. *Chest* 2016;149(2):372-79. doi: http://dx.doi.org/10.1378/chest.14-2842

25. Hu Y. Comparison between caprini and Khorana risk assessment models for hospitalized medical patients with cancer at high risk for venous thromboembolism: a retrospective study. *American Journal of Respiratory and Critical Care Medicine* 2018;197(MeetingAbstracts)

26. Koren O, Hourri A, Elias M, et al. Failure to validate padua score's ability to predict the risk of venous thromboembolism in medical patients. *Clinical Therapeutics* 2017;39(8 Supplement 1):e59.

27. Kucher N, Koo S, Quiroz R, et al. Electronic alerts to prevent venous thromboembolism among hospitalized patients. *N Engl J Med* 2005;352(10):969-77.

28. Lecumberri R, Marqués M, Díaz-Navarlaz MT, et al. Maintained effectiveness of an electronic alert system to prevent venous thromboembolism among hospitalized patients. *Thromb Haemost* 2008;100(4):699-704.

29. Luo X, Zhang F. Validation of the Caprini risk assessment model for venous thromboembolism in Chinese hospitalized patients in a general hospital. *National Medical Journal of China* 2017;97(24):1875-77. doi: http://dx.doi.org/10.3760/cma.j.issn.0376-2491.2017.24.007

30. Maynard GA, Morris TA, Jenkins IH, et al. Optimizing prevention of hospital-acquired venous thromboembolism (VTE): prospective validation of a VTE risk assessment model. *J Hosp Med* 2010;5(1):10-18.

31. McCaffrey R, Bishop M, Adonis-Rizzo M, et al. Development and Testing of a DVT Risk Assessment Tool: Providing Evidence of Validity and Reliability. *Worldviews on Evidence-Based Nursing* 2007;4(1):14-20. doi: 10.1111/j.1741-6787.2007.00074.x

32. Meizoso JP, Karcutskie CAt, Ray JJ, et al. A simplified stratification system for venous thromboembolism risk in severely injured trauma patients. *The Journal of Surgical Research* 2017;207:138-44. doi: https://dx.doi.org/10.1016/j.jss.2016.08.072

33. Monti M, Timpone S, Murdolo G, et al. A new risk assessment model for the stratification of the thromboembolism risk in medical patients: The TEVere Score. *Italian Journal of Medicine* 2019;13(Supplement 2):7-8. doi: http://dx.doi.org/10.4081/itjm.2019.s2

34. Mull N, Hecht T, Umscheid C, et al. Clinical risk prediction scores for venous thromboembolism in hospitalized patients. *International Journal of Technology Assessment in Health Care* 2017;33(Supplement 1):75-76. doi: http://dx.doi.org/10.1017/S0266462317002094

For peer review only

35. Nafee T, Gibson CM, Travis R, et al. Performance of a machine learning model vs. IMPROVE score for VTE prediction in acute medically ill patients: Insights from the APEX trial. *European Heart Journal* 2018;39(Supplement 1):431. doi: http://dx.doi.org/10.1093/eurheartj/ehy565.2160
36. Nnadi C, Soltani SJ, Brice HG, et al. Evaluation of a new thrombo-embolism scoring system (TESS) for spinal surgery. *Spine Journal* 2017;17(3):S21-S22. doi: http://dx.doi.org/10.1016/j.spinee.2016.12.068
37. Obi AT, Pannucci CJ, Nackashi A, et al. Validation of the Caprini Venous Thromboembolism Risk Assessment Model in Critically Ill Surgical Patients. *JAMA Surgery* 2015;150(10):941-48. doi: 10.1001/jamasurg.2015.1841
38. Pannucci CJ, Swistun L, MacDonald JK, et al. Individualized Venous Thromboembolism Risk Stratification Using the 2005 Caprini Score to Identify the Benefits and Harms of Chemoprophylaxis in Surgical Patients: A Meta-analysis. *Annals of Surgery* 2017;265(6):1094-103. doi: https://dx.doi.org/10.1097/SLA.0000000000002126
39. Rafizadeh R, Turgeon RD, Batterink J, et al. Characterization of venous thromboembolism risk in medical inpatients using different clinical risk assessment models. *Canadian Journal of Hospital Pharmacy* 2016;69(6):454-59.
40. Rastogi R, Mehaffey JH, Turrentine BE, et al. Implementation of an Electronic Medical Record Risk Stratification Tool to Reduce Venous Thromboembolism Events. *Journal of the American College of Surgeons* 2020;231(4 Supplement 1):S141-S42. doi: http://dx.doi.org/10.1016/j.jamcollsurg.2020.07.273
41. Razak MKA, Rasheed JI, Hamad AA. A comparison between wells' score and modified doppler ultrasound in the diagnosis of deep venous thrombosis of the lower limb. *International Journal of Pharmaceutical Sciences and Research* 2019;10(5):2369-74. doi: http://dx.doi.org/10.13040/IJPSR.0975-8232.10%285%29.2369-74
42. Robert-Ebadi H, Mostaguir K, Hovens MM, et al. Assessing clinical probability of pulmonary embolism: prospective validation of the simplified Geneva score. *Journal of Thrombosis and Haemostasis* 2017;15(9):1764-69. doi: https://dx.doi.org/10.1111/jth.13770
43. Salim S, Onida S, Davies A. Variability in venous thromboembolism (VTE) risk assessment tools. *Phlebology* 2018;33(1 Supplement 1):36-37. doi: http://dx.doi.org/10.1177/0268355518817748
44. Samama MM, Dahl OE, Mismetti P, et al. An electronic tool for venous thromboembolism prevention in medical and surgical patients. *Haematologica* 2006;91(1):64.
45. Shrotriya S, Dhakal P, Sharma M, et al. Predictors of venous thromboembolism among cancer inpatients: A predictive model. *Journal of Clinical Oncology* 2018;36(15 Supplement 1) doi: http://dx.doi.org/10.1200/JCO.2018.36.15_suppl.e18824

For peer review only

46. Smilg Nicolas C, Tornel Sanchez G, Trujillo Santos J. Concordance among venous thromboembolism risk assessment models. *Concordancia entre las escalas de prediccion de riesgo de tromboembolia venosa* 2018;150(2):61-63. doi: https://dx.doi.org/10.1016/j.medcli.2017.06.021

47. Spirk D, Stuck AK, Hager A, et al. Electronic alert system for improving appropriate thromboprophylaxis in hospitalized medical patients: a randomized controlled trial. *Journal of Thrombosis and Haemostasis* 2017;15(11):2138-46. doi: https://dx.doi.org/10.1111/jth.13812

48. Spyropoulos AC, Lipardi C, Xu J, et al. Modified IMPROVE VTE Risk Score and Elevated D-Dimer Identify a High Venous Thromboembolism Risk in Acutely Ill Medical Population for Extended Thromboprophylaxis. *TH Open* 2020;4(1):e59-e65. doi: https://dx.doi.org/10.1055/s-0040-1705137

49. Spyropoulos AC, Anderson FA, FitzGerald G, et al. Predictive and Associative Models to Identify Hospitalized Medical Patients at Risk for VTE. *Chest* 2011;140(3):706-14. doi: https://doi.org/10.1378/chest.10-1944

50. Stuck AK, Spirk D, Schaudt J, et al. Risk assessment models for venous thromboembolism in acutely ill medical patients. A systematic review. *Thrombosis and Haemostasis* 2017;117(4):801-08. doi: https://dx.doi.org/10.1160/TH16-08-0631

51. Tadesse TA, Kedir HM, Fentie AM, et al. Venous Thromboembolism Risk and Thromboprophylaxis Assessment in Surgical Patients Based on Caprini Risk Assessment Model. *Risk Management and Healthcare Policy* 2020;13:2545-52. doi: https://dx.doi.org/10.2147/RMHP.S272852

52. Taha H, Govindraj E, Jaber F, et al. Improving venous thromboembolism prophylaxis through critical thinking and health informatics. *BMJ Open Quality* 2020;9(2) doi: https://dx.doi.org/10.1136/bmjoq-2019-000885

53. Tung EC, Yu S-Y, Shah K, et al. Reassessment of venous thromboembolism and bleeding risk in medical patients receiving VTE prophylaxis. *Journal of Evaluation in Clinical Practice* 2020;26(1):18-25. doi: https://dx.doi.org/10.1111/jep.13213

54. Veith J, Collier W, Rockwell WB, et al. Direct Comparison of Patient-completed and Physician-completed Caprini Scores for Plastic Surgery Patients. *Plastic and Reconstructive Surgery Global Open* 2019;7(8):e2363. doi: https://dx.doi.org/10.1097/GOX.0000000000002363

55. Winoker JS, Paulucci DJ, Anastos H, et al. MP76-07 Predicting complications following robot-assisted partial nephrectomy with the ACS-NSQIP universal surgical risk calculator. *Journal of Urology* 2017;197(4S):e1015.

56. Yale S, Medlin S, Liang H, et al. Risk assessment model for venothromboembolism in post-hospitalized patients. *International Angiology* 2005;24(3):250.

For peer review only

57. Ye F, Stalvey C, Khuddus MA, et al. A systematic review of mobility/immobility in thromboembolism risk assessment models for hospitalized patients. *Journal of Thrombosis and Thrombolysis* 2017;44(1):94-103. doi: https://dx.doi.org/10.1007/s11239-017-1501-5

58. Zakai NA, Callas PW, Repp AB, et al. Venous thrombosis risk assessment in medical inpatients: the medical inpatients and thrombosis (MITH) study. *J Thromb Haemost* 2013;11(4):634-41.

59. Zambelli R, Nemeth B, Touw C, et al. Is the caprini score a reliable tool to predict the risk of venous thrombosis in orthopedic patients. *Research and Practice in Thrombosis and Haemostasis* 2020;4(SUPPL 1):1194-95. doi: http://dx.doi.org/10.1002/rth2.12393

60. Zhou H-X, Peng L-Q, Yan Y, et al. Validation of the Caprini risk assessment model in Chinese hospitalized patients with venous thromboembolism. *Thrombosis Research* 2012;130(5):735-40. doi: https://doi.org/10.1016/j.thromres.2012.08.001

For peer review only

APPENDIX S3: WIDELY EVALUATED GENERIC RAMS, THEIR ASSOCIATED CHARACTERISTICS AND COMPOSITE CLINICAL VARIABLES

Characteristics	Name of VTE risk assessment model					
	**Caprini Score**	**Padua Prediction Score**	**IMPROVE Predictive score**	**IMPROVE Associative score**	**Geneva Risk Score**	**Kucher Score**
**General**						
Author, year	Caprini 2005[1]	Barbar 2010[2]	Tapson 2007[3]	Spyropoulos 2011[4]	Chopard 2006[5]	Kucher 2005[6]
Applicable cohort	Surgical and medical	Medical	Medical	Medical	Medical	Surgical and medical
Design	Ordinal with cumulative score	Dichotomous variables with cumulative score	Dichotomous variables with VTE probability estimate	Dichotomous variables with VTE probability estimate	Dichotomous variables with cumulative score	Dichotomous variables with cumulative score
Number of VTE risk variables	31	11	4	7	19	8
C-statistic (range) across medical, surgical and trauma cohorts	0.53 - 0.87 (12 studies)	0.594 - 0.756 (7 studies)	0.57 – 0.65 (2 studies)	0.63 – 0.7731 (4 studies)	0.61 (1 study)	0.563-0.756 (4 studies)
When is pharmacological thromboprophylaxis recommended?	Score ≥5	Score ≥4	No specific threshold Identified	No specific threshold identified	Score ≥3	Score ≥4
**Clinical Variables**						
Patient related						
Active cancer	Yes	Yes	Yes	Yes	Yes	Yes (Major risk)
Age	Yes	Yes (≥70)	Yes (≥60)	Yes (≥60)	Yes (≥60)	Yes (≥70 Minor risk)
Dehydration	No	No	No	No	Yes	No
Thrombophilia	Yes (generic and named conditions)	Yes (generic)	Yes (generic)	Yes (generic)	Yes (generic)	Yes (Major risk)
Obesity	Yes (≥25kg/m^2)	Yes (≥30kg/m^2)	No	No	Yes (≥30kg/m^2)	Yes (≥30kg/m^2 Minor risk)
Comorbidity	Yes (1 to 5 points for individual comorbidities)	Yes (1 point each for several individual comorbidities)	No	No	Yes (2 points each for several individual comorbidities)	No
Prior VTE	Yes	Yes	Yes	Yes	Yes	Yes (Major risk)
Family history of VTE	Yes	No	No	No	No	No
Use of HRT	Yes	Yes	No	No	Yes	Yes (Minor risk)
Use of oestrogen containing contraceptive therapy	Yes	Yes	No	No	Yes	Yes (Minor risk)
Varicose veins	Yes	No	No	No	No	No
Pregnancy or postpartum period	No	No	No	No	Yes	No
Unexplained stillbirth or spontaneous abortions	Yes (≥3 spontaneous abortions)	No	No	No	No	No
Current swollen legs	Yes	No	No	No	Yes	No
Current central venous access	Yes	No	No	No	No	No
Recent major surgery	Yes (<1 month)	Yes (<1 month)	No	No	No	No
Recent use of plaster cast immobilisation	Yes (<1 month)	No	No	No	No	No

For peer review only

Lower limb paralysis	Yes	No	No	Yes	No	No
Travel related	No	No	No	No	Yes (>6hours)	No
Admission related						
Reduced mobility	Yes (variable points)	Yes	No	Yes (≥7days)	Yes (≥3 days)	Yes (Minor risk)
Arthroplasty surgery	Yes	No	No	No	No	No
Hip fracture	Yes	No	No	No	No	No
Pelvic or lower limb surgery	Yes (arthroscopic)	No	No	No	No	No
Total anaesthetic and surgical time	Yes (≥45mins)	No	No	No	No	Yes (≥60mins intermediate risk)
Acute surgical admission	No	No	No	No	No	No
Acute infection	No	Yes	No	No	Yes	No
Acute rheumatologic disorder	No	Yes	No	No	Yes	No
Critical care admission	No	No	No	Yes	No	No
Surgery leading to reduced mobility	Yes	No	No	No	No	No
Other						
'Other risk factors'	Yes	No	No	No	No	No

HRT, hormone replacement therapy; VTE, venous thromboembolism

REFERENCES (APPENDIX S3)

1. Caprini JA, Arcelus JI, Hasty JH, et al. Clinical assessment of venous thromboembolic risk in surgical patients. *Semin Thromb Hemost* 1991;17:304-12.

2. Barbar S, Noventa F, Rossetto V, et al. A risk assessment model for the identification of hospitalized medical patients at risk for venous thromboembolism: the Padua Prediction Score. *Journal of Thrombosis and Haemostasis* 2010;8(11):2450-57. doi: 10.1111/j.1538-7836.2010.04044.x

3. Tapson VF, Decousus H, Pini M, et al. Venous thromboembolism prophylaxis in acutely ill hospitalized medical patients: findings from the International Medical Prevention Registry on Venous Thromboembolism. *Chest* 2007;132(3):936-45.

4. Spyropoulos AC, Anderson FA, FitzGerald G, et al. Predictive and Associative Models to Identify Hospitalized Medical Patients at Risk for VTE. *Chest* 2011;140(3):706-14. doi: https://doi.org/10.1378/chest.10-1944

5. Chopard P, Spirk D, Bounameaux H, et al. Identifying acutely ill medical patients requiring thromboprophylaxis. . *J Thromb Haemost* 2006;4(4):915-6.

6. Kucher N, Koo S, Quiroz R, et al. Electronic alerts to prevent venous thromboembolism among hospitalized patients. *N Engl J Med* 2005;352(10):969-77.

For peer review only

APPENDIX S4: SUMMARY OF PREDICTIVE PERFORMANCE FOR STUDIES INVOLVING HOSPITAL INPATIENTS WHO REQUIRED CARE FOR CANCER, STROKE, BURN INJURIES AND SEPSIS OR WERE A MIXED MEDICAL/SURGICAL COHORT

**Data source**	**Endpoint**	**Incidence**	**Risk assessment model**	**Threshold or cut-off**	**Predictive performance**		
					**C-statistic (95% CI)**	**Sensitivity (95% CI)**	**Specificity (95% CI)**
**MIXED INPATIENTS**							
Autar, 2003[22]	DVT	18.9%	•Novel (Autar, 2003)	Risk score ≤6	NR	100% (NR)	100% (NR)
			•Novel (Autar, 2003)	Risk score 7-10	NR	86.0% (NR)	68.0% (NR)
			•Novel (Autar, 2003)	Risk score 11-14	NR	68.0% (NR)	31.0% (NR)
			•Novel (Autar, 2003)	Risk score ≥15	NR	25.0% (NR)	10.0% (NR)
Elias, 2017[34]	VTE	0.8%	•Padua (automated)	Risk score ≥5	0.81 (0.79, 0.83)	85.4% (NR)	53.3% (NR)
Chen, 2018[30]	DVT	NA	•Caprini	Risk score ≥4	NR	73.8% (NR)	64.7% (NR)
			•Caprini	Risk score ≥5	NR	62.8% (NR)	82.6% (NR)
			•Padua	Risk score ≥3	NR	53.5% (NR)	82.1% (NR)
			•Padua	Risk score ≥4	NR	42.1% (NR)	92.5% (NR)
Bo, 2020[29]	DVT	0.9%	•Caprini	Risk score ≥3.5	0.74 (0.71, 0.77)	75.0% (NR)	62.0% (NR)
**CANCER INPATIENTS**							
Abdel-Razeq, 2010[21]	VTE	3.5%	•Caprini (modified)	Risk score ≥3	NR	100% (NR)	9.2% (NR)
			•Caprini (modified)	Risk score ≥5	NR	57.1% (NR)	53.2% (NR)
Patell, 2017[54]	VTE	3.8%	•Khorana	Risk score ≥3	NR	18.9% (NR)	87.2% (NR)
Hu, 2020[42]	VTE	NA	•Caprini	Risk score ≥5	0.71 (0.66, 0.75)	82.4% (NR)	46.2% (NR)
			•Khorana	Risk score ≥2	0.58 (0.53, 0.63)	35.3% (NR)	78.7% (NR)
Shang, 2020[60]	VTE	NA	•Caprini 2009	Risk score ≥3	0.72 (0.70, 0.74)	83.5% (NR)	52.7% (NR)
			•Caprini 2013	Risk score ≥5	0.80 (0.78, 0.82)	80.9% (NR)	65.9% (NR)
**BURNS INPATIENTS**							
Pannucci, 2012[53]	VTE	1.0%	•Novel (Pannucci 2012)	NR	0.75	NR	NR
**POST-STROKE INPATIENTS**							
Liu, 2014[44]	DVT	10.5%	•Post-stroke DVT Prediction System	NR	0.65 (0.59, 0.70)	NR	NR
**SEPSIS INPATIENTS**							
Vardi, 2013[64]	VTE	1.3%	•Padua	NR	•All patients<br>0.58 (0.43, 0.73)	•All patients<br>NR	•All patients<br>NR
			•Padua	NR	•No prophylaxis<br>0.54 (0.37, 0.71)	•No prophylaxis<br>NR	•No prophylaxis<br>NR

CI, confidence interval; DVT, deep vein thrombosis; NA, not applicable; NR, not reported; VTE, venous thromboembolism

For peer review only - http://bmjopen.bmj.com/site/about/guidelines.xhtml

PRISMA 2009 Checklist

Section/topic	#	Checklist item	Reported on page #
**TITLE**			
Title	1	Identify the report as a systematic review, meta-analysis, or both.	1
**ABSTRACT**			
Structured summary	2	Provide a structured summary including, as applicable: background; objectives; data sources; study eligibility criteria, participants, and interventions; study appraisal and synthesis methods; results; limitations; conclusions and implications of key findings; systematic review registration number.	2-3
**INTRODUCTION**			
Rationale	3	Describe the rationale for the review in the context of what is already known.	5
Objectives	4	Provide an explicit statement of questions being addressed with reference to participants, interventions, comparisons, outcomes, and study design (PICOS).	5
**METHODS**			
Protocol and registration	5	Indicate if a review protocol exists, if and where it can be accessed (e.g., Web address), and, if available, provide registration information including registration number.	3, 6
Eligibility criteria	6	Specify study characteristics (e.g., PICOS, length of follow-up) and report characteristics (e.g., years considered, language, publication status) used as criteria for eligibility, giving rationale.	6
Information sources	7	Describe all information sources (e.g., databases with dates of coverage, contact with study authors to identify additional studies) in the search and date last searched.	6-7
Search	8	Present full electronic search strategy for at least one database, including any limits used, such that it could be repeated.	Online supplementary - Appendix S1
Study selection	9	State the process for selecting studies (i.e., screening, eligibility, included in systematic review, and, if applicable, included in the meta-analysis).	7
Data collection process	10	Describe method of data extraction from reports (e.g., piloted forms, independently, in duplicate) and any processes for obtaining and confirming data from investigators.	7
Data items	11	List and define all variables for which data were sought (e.g., PICOS, funding sources) and any assumptions and simplifications made.	7
Risk of bias in individual studies	12	Describe methods used for assessing risk of bias of individual studies (including specification of whether this was done at the study or outcome level), and how this information is to be used in any data synthesis.	7-8
Summary measures	13	State the principal summary measures (e.g., risk ratio, difference in means).	8
Synthesis of results	14	Describe the methods of handling data and combining results of studies, if done, including measures of consistency (e.g., I^2) for each meta-analysis.	8

For peer review only - http://bmjopen.bmj.com/site/about/guidelines.xhtml

PRISMA 2009 Checklist

Section/topic	#	Checklist item	Reported on page #
Risk of bias across studies	15	Specify any assessment of risk of bias that may affect the cumulative evidence (e.g., publication bias, selective reporting within studies).	7-8
Additional analyses	16	Describe methods of additional analyses (e.g., sensitivity or subgroup analyses, meta-regression), if done, indicating which were pre-specified.	N/A
**RESULTS**			
Study selection	17	Give numbers of studies screened, assessed for eligibility, and included in the review, with reasons for exclusions at each stage, ideally with a flow diagram.	8; Figure 1; Online supplementary - Appendix S2
Study characteristics	18	For each study, present characteristics for which data were extracted (e.g., study size, PICOS, follow-up period) and provide the citations.	9-15
Risk of bias within studies	19	Present data on risk of bias of each study and, if available, any outcome level assessment (see item 12).	15-17
Results of individual studies	20	For all outcomes considered (benefits or harms), present, for each study: (a) simple summary data for each intervention group (b) effect estimates and confidence intervals, ideally with a forest plot.	18-20; Figure 3; Online supplementary – Appendix S4
Synthesis of results	21	Present results of each meta-analysis done, including confidence intervals and measures of consistency.	N/A
Risk of bias across studies	22	Present results of any assessment of risk of bias across studies (see Item 15).	18, Figure 2
Additional analysis	23	Give results of additional analyses, if done (e.g., sensitivity or subgroup analyses, meta-regression [see Item 16]).	N/A
**DISCUSSION**			
Summary of evidence	24	Summarize the main findings including the strength of evidence for each main outcome; consider their relevance to key groups (e.g., healthcare providers, users, and policy makers).	21-23
Limitations	25	Discuss limitations at study and outcome level (e.g., risk of bias), and at review-level (e.g., incomplete retrieval of identified research, reporting bias).	22
Conclusions	26	Provide a general interpretation of the results in the context of other evidence, and implications for future research.	23
**FUNDING**			
Funding	27	Describe sources of funding for the systematic review and other support (e.g., supply of data); role of funders for the systematic review.	24

From: Moher D, Liberati A, Tetzlaff J, Altman DG, The PRISMA Group (2009). Preferred Reporting Items for Systematic Reviews and Meta-Analyses: The PRISMA Statement. PLoS Med 6(6): e1000097. doi:10.1371/journal.pmed1000097

For peer review only

For peer review only - http://bmjopen.bmj.com/site/about/guidelines.xhtml

PRISMA 2009 Checklist

For more information, visit: **www.prisma-statement.org**.

For peer review only
